# The "Martian" flora: new collections of vascular plants, lichens, fungi, algae, and cyanobacteria from the Mars Desert Research Station, Utah

**DOI:** 10.3897/BDJ.4.e8176

**Published:** 2016-06-09

**Authors:** Paul C. Sokoloff, Colin E. Freebury, Paul B. Hamilton, Jeffery M. Saarela

**Affiliations:** ‡ Canadian Museum of Nature, Ottawa, Canada

**Keywords:** Analog Research, Floristics, Astrobiology

## Abstract

The Mars Desert Research Station is a Mars analog research site located in the desert outside of Hanksville, Utah, U.S.A. Here we present a preliminary checklist of the vascular plant and lichen flora for the station, based on collections made primarily during a two-week simulated Mars mission in November, 2014. Additionally, we present notes on the endolithic chlorophytes and cyanobacteria, and the identification of a fungal genus also based on these collections. Altogether, we recorded 38 vascular plant species from 14 families, 13 lichen species from seven families, six algae taxa including both chlorophytes and cyanobacteria, and one fungal genus from the station and surrounding area. We discuss this floristic diversity in the context of the ecology of the nearby San Rafael Swell and the desert areas of Wayne and Emery counties in southeastern Utah.

## Introduction

"*Hell yeah I'm a botanist! Fear my botany powers!" - Mark Watney (The Martian, by Andy Weir)*.

**Figure 1.**
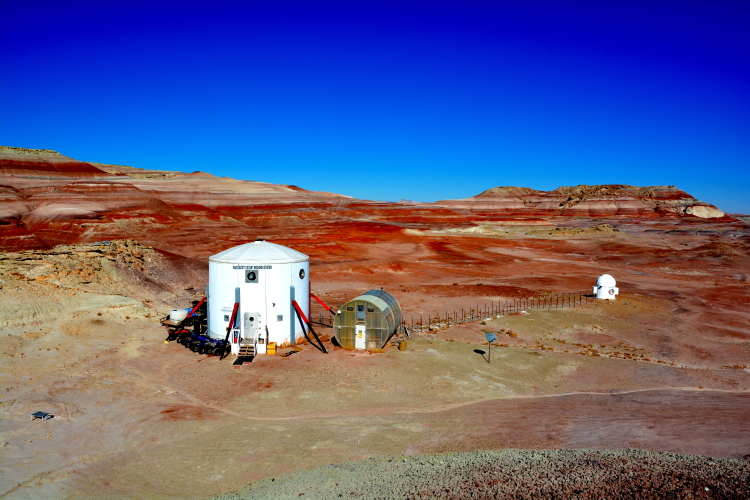
The Mars Desert Research Station near Hanksville, Utah. The large white building on the left is the primary living and working structure (commonly known as the "hab"). To the right of the hab is the station's greenhouse (the "GreenHab"). This photo shows the original GreenHab, burned down on December 29th, 2014, and has since been replaced. The white building to the right is the Musk Observatory. Photo by P.C. Sokoloff. Photo taken on November 16th, 2014.

**Figure 2.**
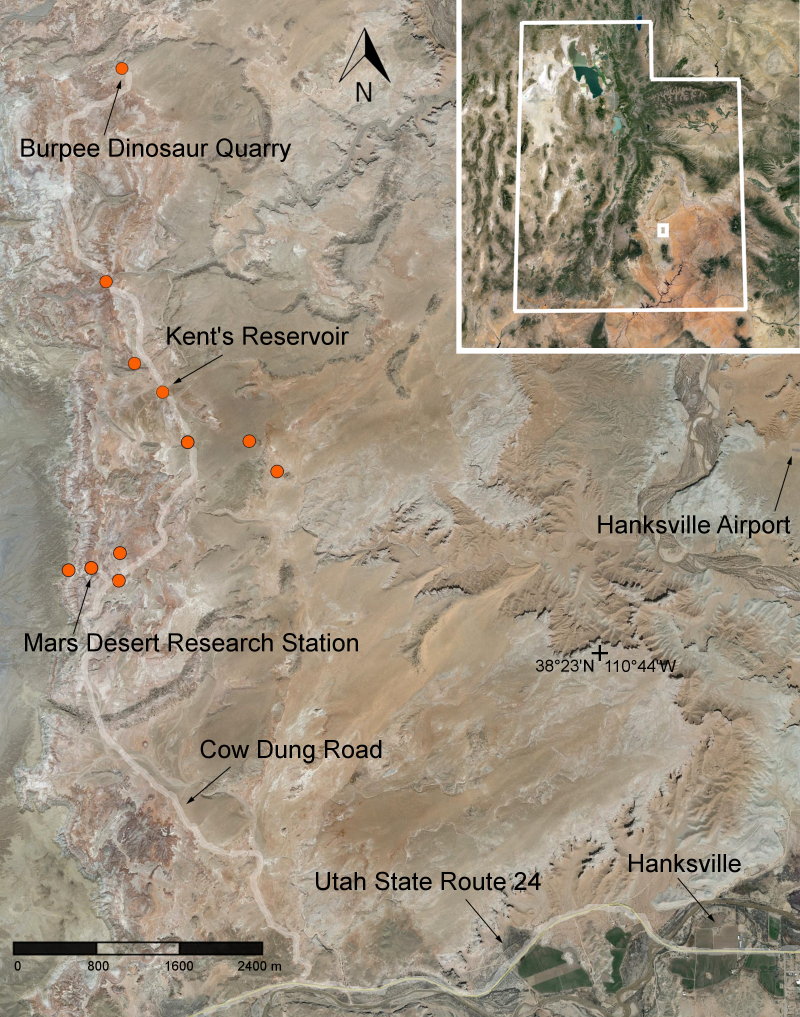
Location of the Mars Desert Research Station habitat and our study area, including 11 collection sites (orange dots, ten are from 2014, one is from 2015), northwest of Hanksville, Utah, U.S.A. Inset map of Utah at top right indicates extent of map (smaller white outlined area). Map data courtesy of Google, SIO, NOAA, U.S. Navy, NGA, GEBCO, and Landsat.

The Mars Desert Research Station (MDRS) (http://mdrs.marssociety.org/) is a Mars analog research site located in the desert approximately 9 km outside of Hanksville, in Wayne County, Utah, U.S.A. at 38°24'23.12"N, 110°47'30.94"W (Figs 1, 2). The station is located 5 km north of Utah State Route 24 along Cow Dung Road, which continues north for another 5 km to the Burpee Dinosaur Quarry, a recently described bone bed from the Jurassic Morrison Formation (Mathews et al. 2009).

Constructed by the Mars Society (http://www.marssociety.org/) in 2002 and operated continuously ever since, MDRS, and its Arctic counterpart, the Flashline Mars Arctic Research Station (FMARS) (http://fmars.marssociety.org/), on Devon Island, Nunavut, Canada, are designed as testbeds for future manned missions to Mars (Bamsey et al. 2009). Visiting crews from multiple scientific and engineering disciplines conduct research at the stations on how to live and work on Mars without having to leave Earth.

Astrobiology, the study of the evolution and distribution of life throughout the universe (including Earth), is a field increasingly represented at MDRS. Professionals in the field seek to refine techniques to detect life on other worlds, and to answer outstanding questions about the origins of life here on Earth (Thiel et al. 2011). At MDRS, astrobiologists have conducted studies ranging from the detection of biomarkers (Martins et al. 2011) and extremophiles (Direito et al. 2011) in the local soils, to the automated detection of life using computer algorithms (Gross et al. 2009), and more. This search for life in extreme environments on Earth is an excellent analog to the search for life on Mars. During their studies, astrobiologists, soil specialists, geologists and other scientists working at MDRS frequently come across, or seek out, vascular plants, lichens, algae, cyanobacteria, and fungi while conducting field research.

Utah is a floristically diverse state, with some 2995 vascular plant species and infrataxa distributed in diverse ecosystems (Welsh et al. 1993). The cool deserts of southeastern Utah, where MDRS is located, possess diverse vascular plant (Andersen 1996) and biological soil crust communities (Belnap 2002). The distribution of these communities is determined primarily by the underlying geology, elevation, and moisture, all of which vary across the deserts of the region (Coles et al. 2009).

There is a long history of floristic work in southeastern Utah. Complete vascular plant inventories exist for two ecologically similar areas close to MDRS: Capitol Reef National Park (Heil et al. 1993, Fertig 2009) and Glen Canyon National Recreation Area (Hill and Ayers 2009). The latter location contains the Orange Cliffs, which have a well-studied vascular plant flora (Shultz et al. 1987). Fertig (2009) presents a comprehensive overview of previous vascular plant collection efforts throughout the region. The closest and best studied flora near MDRS is that of the San Rafael Swell, approximately 23 km northwest of the station. Harris (1983) compiled a vascular plant inventory, including 478 species, for this large geological feature that dominates southeastern Utah.

The inventories of vascular plants from the San Rafael Swell and Capitol Reef National Park provide a useful comparison to our own study region at MDRS. All three sites share common geological characteristics, notably the Mancos Shale and Morrison Formation (Coles et al. 2009, Direito et al. 2011, Gilluly 1929). These locations also share many common vascular plant communities based on this underlying geology.

For example, the vascular plant communities of the desert flats immediately surrounding MDRS correspond well to the description of the "Salt Desert Shrub Zone" in Harris (1983), which is characteristic of the Mancos Shale (Coles et al. 2009). The vegetation found on nearby sandstone outcrops and plateaus relate to the "Mixed Desert Shrub Zone" (Harris 1983). The alkaline clay soils of the station area are dominated by *Achnatherum hymenoides*, *Atriplex* sp., *Eriogonum inflatum*, and *Kali tragus*, while the sandstone outcrops support communities of *Artemisia* sp., *Ephedra viridis*, and *Dasyochloa pulchella*. Where these two ecological zones meet north of MDRS near Kent's Reservoir (a small pond immediately west of Cow Dung Road at 38°25'27.70"N, 110°47'17.01"W), there is an ecological gradient, including water-intensive stands of *Tamarix ramosissima* on wet alkaline soils of the Salt Desert Zone, mixed with *Artemesia* and *Ericameria nauseosa*, both characteristic of the Mixed Desert zone (Harris 1983). Further north along Cow Dung Road, the floodplains of a seasonally wet creek support healthy stands of *Sarcobatus vermiculatus* and other species that are known from the floodplains of the Salt Desert Zone (Harris 1983). This wide variety of habitat types, all differentiated by water availability and geological substrate, support a diverse vascular plant flora around MDRS.

The lichen flora of eastern Wayne County, in which MDRS is located, and adjacent eastern Emery County includes approximately 61 species. This is an estimate based on a catalogue of the lichens of Utah (St. Clair et al. 1991), notes on the state's lichen flora (Newberry and St. Clair 1991, St. Clair et al. 1995), and a flora of the terricolous lichens of the San Rafael Swell (Rajvanshi et al. 1998). A large part of this regional flora was found on soil (28) or rock (26). Of the total, seven species from Capitol Reef National Park in Wayne Co. were collected on either *Juniperus*, which was not observed in the study area, or on wood (e.g., dead shrubs), which was not examined for lichens during this study. The number of 61 reported lichen species for the reference region is dated and therefore likely a conservative estimate of the potential number of species to be found in the study area.

Given the diverse local flora, and the increasing prevalance of biological studies at MDRS, our primary objective for this study was to collect and identify the vascular plants, lichens, fungi, and endolithic cyanobacteria and algae (sub-surface photosynthetic life) at MDRS. These endolithic taxa are of particular interest to astrobiologists as model systems in the search for life on Mars (Direito et al. 2011). A secondary objective of our fieldwork was to practice and evaluate the techniques needed to collect biological samples during simulated Martian fieldwork.

## Materials and methods

### Fieldwork

**Figure 3.**
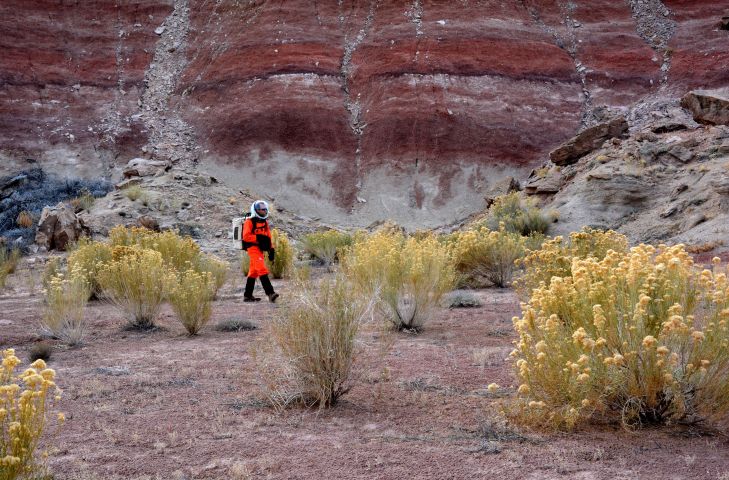
MDRS Expedition 143 Commander Paul Knightly walking through stands of *Ericameria nauseosa* (*Sokoloff 284*, large yellow shrubs) and *Ephedra viridis* (wiry green shrub at centre of photo) at Kent's Reservoir while wearing a simulated spacesuit. Photo by P.C. Sokoloff.

From November 15–30, 2014, Paul Sokoloff participated in MDRS Expedition 143 (http://mdrs.marssociety.org/home/crew-143) as Crew Biologist, and was part of a six-person crew staying at MDRS for an 11-day simulated mission to Mars. Over the course of five extra-vehicular activities (EVAs: all activities that take place outside the "hab" while in simulation), representing approximately 20 hours of collecting time, all vascular plant, lichen, fungi, and endolithic cyanobacteria and algae species encountered (except for completely dead, unidentifiable vascular plants), were collected by Paul Sokoloff with the assistance of other crew members (typically one other crew member per trip). In each area visited, as many microhabitats as possible were explored for unique plants and lichens. EVA teams wore simulated spacesuits, consisting of overalls, hiking boots with gaiters, thick gloves, a backpack containing a fan (the simulated oxygen supply), and a clear bubble helmet. These simulated suits let field teams practice standard field work activities while having to cope with restricted vision and movement, both obstacles that would have to be overcome on a real mission to Mars (Fig. 3).

While on EVAs we used standard collection techniques: plant samples were dug out at the roots or clipped from larger trunks, crustose lichens and cyanobacteria were chiseled away from rocky substrates, material was placed in numbered sample bags, coordinates were logged using a standard GPS receiver, and collection site notes were recorded. Owing to their fragile nature and the requirement for special collecting techniques, no effort was made to collect any of the soil crust lichens that might be found at MDRS. Specimens were pressed and processed as appropriate inside the lab at MDRS (where simulated spacesuits were not required).

**Figure 10.**
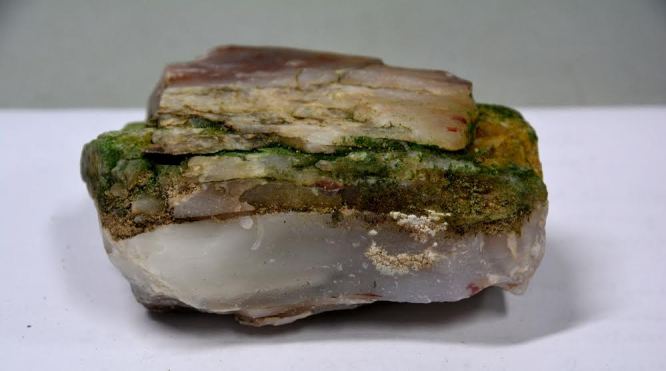
*Gloeocapsa* sp (*Sokoloff 301*). A blue-green layer of cyanobacteria (and an unknown chorophyte) growing on the underside of quartzite stone (stone has been flipped and the surface previously exposed is at the bottom of this photograph). Photo by P.C. Sokoloff.

Where possible, photographs of the specimen *in situ* and of the habitat were taken at the time of collection. These photos are included in the species accounts below. Photos by C.E. Freebury, P.B. Hamilton, and those in Figures 8 and 10 by P.C. Sokoloff were taken under laboratory conditions in Ottawa. All other photographs were taken in the field. Photographs of vouchered lichen and vascular plant specimens include the collection number, while photographs of plants that were not collected include the location at which the photo was taken and the date.

In total, we collected 46 vascular plant specimens, 18 lichen samples (some of which were subdivided upon return to Ottawa), three rock samples containing endolithic cyanobacteria and algae, and one fungus from 10 collection sites during Expedition 143 (Fig. 2). These collection sites were chosen based on satellite images and topographical maps as representatives of the common habitat types around MDRS, so as to maximize the number of different species collected. The Mars Desert Research Station is situated in the southwest corner of this study area: our furthest north (five km from the "hab") and east (two km from the "hab") collection sites delimit an approximately 10 km2 area covered by this study.

A follow-up visit was made to the study area from September 19–20, 2015, during which Paul Sokoloff collected three additional vascular plant specimens from one existing and one new collection site, and photo-documented one vascular plant species. These activities were not carried out under simulated Martian conditions.

### Vascular Plant and Lichen Identification

With the exception of the fungus specimen, which was sent to the National Mycological Herbarium at Agriculture and Agri-Food Canada (DAOM) in Ottawa, the entire set of 71 specimens was deposited at the National Herbarium of Canada (CAN - vascular plants, CANL - lichens, CANA - algae), Canadian Museum of Nature. A duplicate set of 50 specimens was deposited at the Intermountain Herbarium at Utah State University (UTC).

All authors identified a subset of the collections reported here, as noted in the collection data for each specimen. Where appropriate, species accounts include analysis and discussion for each taxon. Vascular plant species were identified using the Flora of North America (chapters cited in species accounts), A Utah Flora (Welsh et al. 1993), and various primary literature sources cited in the species accounts below. For each vascular plant species we report whether or not the species is recorded in the nearby, well-studied San Rafael Swell (Harris 1983).

Lichen species were identified using the methods and keys provided in Lichens of North America Brodo et al. (2001) and Brodo et al. (2016). One lichen specimen (*Sokoloff 307*) was not identifiable, and is not treated here. All mounted and processed vascular plant and lichen specimens at CAN and CANL were scanned and/or photographed, and are presented here as supplemental files, as cited in the species descriptions.

### Algae and Cyanobacteria Identification

Selected fractured rock samples (*Sokoloff 249, 290, 301*) were examined at the Canadian Museum of Nature lab with an Olympus SZX12 dissecting microscope. Observed zones of endolithic algae and cyanobacteria were sampled by selectively isolating the algae from associated granular stone particles. Stone fragments with algae and/or cyanobacteria were picked and either cultured or placed on a microscope slide and squashed for direct examination. Culturing was conducted using 5 ml of sterile BBM Medium in 50 ml falcon tubes held at 22°C under natural and ambient light during the day. After two weeks the cultures were examined. Compound microscope examinations of original and cultured squash samples were initiated after stone fragments were removed from the slides before a semi-permanent slide mount was prepared. A Leica DMR HC microscope with differential interference contrast (DIC), phase contrast and bright field optics was used with a 100x Plan Apo (NA 1.35) objective for all microscopic examinations. Identifications were primarily based on the taxonomic treatments of Komárek and Anagnostidis (1999) and Ettl and Gärtner (2014).

## Checklists

### Algae and Cyanobacteria Collections

#### Chlorophyta

##### Notes

Six taxa of endolithic and endophytic algae and cyanobacteria were identified in this study. Two taxa independently (*Trebouxia* sp. 1 & *Gloeocapsa* sp.) formed a mixed layer (predominantly *Gloeocapsa* sp.) 1–4 mm below the surface within one sandstone sample (*Sokoloff 290*, Figs 4, 5). In a quartz sample (*Sokoloff 301*), *Gloeocapsa* sp. was the dominant taxon growing on the underside of a quartz rock found embedded in desert sand, along with an unknown chlorophyte (Chaetophorales) within crevices of the rock. In another sample, *Trebouxia* sp. 1 was also observed as a distinct layer 1–4 mm below the sandstone surface (*Sokoloff 249*). On average, there was 1–6 sand grains of varying shapes, sizes and orientation between the outer surface and the endolith. The algae layer varied from 0.5 to 1.5 mm in thickness and ranged from disrupted to continuous. In the three samples collected, the expanse of these layers ranged from 0.5 cm^2^ to 2.5 cm^2^. In two samples (*Sokoloff 249, 290*) lichens (Lecanora cf. garovaglii, *Acarospora strigata*) were scattered across the surface with subsurface expansions of the fungi into the sandstone (Fig. 6d, Fig. 7). Scattered fungi were also observed within the endolithic algae layers (Fig. 5). In two lichen samples examined for photobionts, *Heteroplacidium compactum* (*Sokoloff 296*) and *Placidium acarosporoides* (*Sokoloff 305*), the alga *Myrmecia* sp. was observed within the lichens.

#### Chlorophyta

#### Trebouxia sp. 1

Trebouxia sp. 1 [T. cf. anticipata Ahm./ T. cf. gelatinosa Ahm./ T. cf. aggregata (Arch.) Gärtner]

##### Materials

- **Type status:** Other material. **Occurrence:** recordNumber: 249; recordedBy: Sokoloff, Paul C.; **Taxon:** kingdom: Plantae; phylum: Chlorophyta; class: Trebouxiophyceae; order: Trebouxiales; family: Trebouxiaceae; genus: Trebouxia; **Location:** continent: North America; country: United States of America; countryCode: USA; stateProvince: Utah; county: Wayne County; municipality: Hanksville; locality: Mars Desert Research Station; verbatimLocality: Vicinity of the Mars Desert Research Station, Hanksville, Utah, 500 m radius of "hab"; verbatimElevation: 1371 m; verbatimLatitude: 38°24'23.2"N; verbatimLongitude: 110°47'31.1"W; coordinateUncertaintyInMeters: 50; **Identification:** identifiedBy: Hamilton, Paul B.; dateIdentified: 2016; **Event:** verbatimEventDate: November 17, 2014; habitat: Sandstone; **Record Level:** institutionID: CMN; collectionID: CANA 117864; collectionCode: CANA, UTC; basisOfRecord: Dried Specimen

##### Notes

Cells spherical to weakly elliptical, 8–15.0 μm in diameter (Fig. 4 a-c; Fig. 6 a). In culture cells were spherical, up to 19 μm in diameter, the cell wall sheath <0.5 μm. Chloroplast plate-like, sometimes lobed, covering most of the cell. One pyrenoid present, at times difficult to distinguish. In the natural population the cell wall sheath was thick, up to 1.5 μm. Colonies of daughter cells tightly packed, forming wedge-shaped colonies in spherical to elliptical clusters. Endolithic, forming a fine linear layer (flake-like) 0.1–0.4 mm below surface of sandstone.

#### Trebouxia sp. 2

Trebouxia sp. 2 [T. cf. gelatinosa Ahm./ T. cf. aggregata (Arch.) Gärtner]

##### Materials

- **Type status:** Other material. **Occurrence:** recordNumber: 249; recordedBy: Sokoloff, Paul C.; **Taxon:** kingdom: Plantae; phylum: Chlorophyta; class: Trebouxiophyceae; order: Trebouxiales; family: Trebouxiaceae; genus: Trebouxia; **Location:** continent: North America; country: United States of America; countryCode: USA; stateProvince: Utah; county: Wayne County; municipality: Hanksville; locality: Mars Desert Research Station; verbatimLocality: Vicinity of the Mars Desert Research Station, Hanksville, Utah 500 m, radius of "hab"; verbatimElevation: 1371 m; verbatimLatitude: 38°24'23.2"N; verbatimLongitude: 110°47'31.1"W; coordinateUncertaintyInMeters: 50; **Identification:** identifiedBy: Hamilton, Paul B.; dateIdentified: 2016; **Event:** verbatimEventDate: November 17, 2014; habitat: Sandstone; **Record Level:** institutionID: CMN; collectionID: CANA 117864; collectionCode: CANA, UTC; basisOfRecord: Dried Specimen
- **Type status:** Other material. **Occurrence:** recordNumber: 290; recordedBy: Sokoloff, Paul C.; **Taxon:** kingdom: Plantae; phylum: Chlorophyta; class: Trebouxiophyceae; order: Trebouxiales; family: Trebouxiaceae; genus: Trebouxia; **Location:** continent: North America; country: United States of America; countryCode: USA; stateProvince: Utah; county: Wayne County; municipality: Hanksville; locality: Mars Desert Research Station; verbatimLocality: "Comm check" hill, 1.7 km north of Mars Desert Research Station, just west of Cow Dung Road; verbatimElevation: 1371 m; verbatimLatitude: 38°25'3.15"N; verbatimLongitude: 110°46'54.59"W; coordinateUncertaintyInMeters: 50; **Identification:** identifiedBy: Hamilton, Paul B.; dateIdentified: 2016; **Event:** verbatimEventDate: November 22, 2014; habitat: Sandstone at crest of *Artemisia* and *Ephedra* dominated hilltop; **Record Level:** institutionID: CMN; collectionID: CANA 117865; collectionCode: CANA; basisOfRecord: Dried Specimen

##### Notes

Cells elliptical to spherical, 7–21.0 μm in diameter (Fig. 7 a-f). In culture cells were spherical, up to 19–22 μm in diameter and the cell wall sheath was <0.5 μm. Chloroplast large, plate-like to lobed, covering most of the cell. One pyrenoid present, at times difficult to distinguish. In the natural population, the cell wall sheath was <1.5 μm. Colonies of daughter cells tightly packed, forming wedge-shaped colonies in spherical to elliptical clusters. This was found in two lichen species (*Acarospora strigata* and Lecanora cf. garovaglii) forming a fine linear layer 5–100 μm thick, approximately 75–100 μm below the lichen surface.

#### Trebouxia sp. 3

Trebouxia sp. 3 [T. cf. gelatinosa Ahm./ T. cf. aggregata (Arch.) Gärtner]

##### Materials

- **Type status:** Other material. **Occurrence:** recordNumber: 290; recordedBy: Sokoloff, Paul C.; **Taxon:** kingdom: Plantae; phylum: Chlorophyta; class: Trebouxiophyceae; order: Trebouxiales; family: Trebouxiaceae; genus: Trebouxia; **Location:** continent: North America; country: United States of America; countryCode: USA; stateProvince: Utah; county: Wayne County; municipality: Hanksville; locality: Mars Desert Research Station; verbatimLocality: "Comm check" hill, 1.7 km north of Mars Desert Research Station, just west of Cow Dung Road; verbatimElevation: 1371 m; verbatimLatitude: 38°25'3.15"N; verbatimLongitude: 110°46'54.59"W; coordinateUncertaintyInMeters: 50; **Identification:** identifiedBy: Hamilton, Paul B.; dateIdentified: 2016; **Event:** verbatimEventDate: November 22, 2014; habitat: Sandstone at crest of *Artemisia* and *Ephedra* dominated hilltop; **Record Level:** institutionID: CMN; collectionID: CANA 117865; collectionCode: CANA; basisOfRecord: Dried Specimen

##### Notes

Cells spherical to weakly elliptical, 7.0–15.0 μm in diameter (Fig. 8 a-c). Chloroplast plate-like, covering most of the cell. Culturing of this taxon was not successful. Pyrenoid difficult to distinguish. Cell wall sheath 1–2+ μm thick. Small clusters of cells, or colonies of daughter cells. Endophytic within an unidentified lichen (brown-black spheres) from the Verrucariaceae family (Fig. 8.

#### Trebouxia sp. 4

Trebouxia sp. 4 [*T*. cf. *usneae* (Hildreth & Ahm.) Gärtner/ *T*. cf. *potteri* Ahm. ex. Gärtner]

##### Materials

- **Type status:** Other material. **Occurrence:** recordNumber: 290; recordedBy: Sokoloff, Paul C.; **Taxon:** kingdom: Plantae; phylum: Chlorophyta; class: Trebouxiophyceae; order: Trebouxiales; family: Trebouxiaceae; genus: Trebouxia; **Location:** continent: North America; country: United States of America; countryCode: USA; stateProvince: Utah; county: Wayne County; municipality: Hanksville; locality: Mars Desert Research Station; verbatimLocality: "Comm check" hill, 1.7 km north of Mars Desert Research Station, just west of Cow Dung Road; verbatimElevation: 1371 m; verbatimLatitude: 38°25'3.15"N; verbatimLongitude: 110°46'54.59"W; coordinateUncertaintyInMeters: 50; **Identification:** identifiedBy: Hamilton, Paul B.; dateIdentified: 2016; **Event:** verbatimEventDate: November 22, 2014; habitat: Sandstone at crest of *Artemisia* and *Ephedra* dominated hilltop; **Record Level:** institutionID: CMN; collectionID: CANA 117865; collectionCode: CANA; basisOfRecord: Dried Specimen

##### Notes

Cells spherical to weakly elliptical, 12–15.0 μm in diameter (Fig. 4 e-f). Chloroplast lobed, covering most of the cell. One to many pyrenoids present, at times difficult to distinguish. In the natural population the cell wall sheath was thin <0.8 μm. Small colonies of daughter cells tightly packed in forming broad wedge-shaped colonies in spherical to elliptical clusters. Endolithic, scattered with *Gloeocapsa* sp. 0.1–0.4 mm below the sandstone surface.

#### Myrmecia sp.

##### Materials

- **Type status:** Other material. **Occurrence:** recordNumber: 296; recordedBy: Sokoloff Paul C.; **Taxon:** kingdom: Plantae; phylum: Chlorophyta; class: Trebouxiophyceae; order: Trebouxiales; family: Trebouxiaceae; genus: Myrmecia; taxonRank: Species; **Location:** continent: North America; country: United States of America; countryCode: USA; stateProvince: Utah; county: Wayne County; municipality: Hanksville; locality: Mars Desert Research Station; verbatimLocality: Roadside along ATV trail 2 km east of Mars Desert Research Station; verbatimElevation: 1348 m; verbatimLatitude: 38°24'53.8"N; verbatimLongitude: 110°46'18"W; coordinateUncertaintyInMeters: 50; **Identification:** identifiedBy: Hamilton Paul B.; dateIdentified: 2015; **Event:** verbatimEventDate: November 23 2014; habitat: Sandstone rubble and sandy plains.; **Record Level:** institutionID: CMN; collectionID: CANL 127964; collectionCode: CANL, UTC; basisOfRecord: Dried Specimen
- **Type status:** Other material. **Occurrence:** recordNumber: 305; recordedBy: Sokoloff Paul C.; **Taxon:** kingdom: Plantae; phylum: Chlorophyta; class: Trebouxiophyceae; order: Trebouxiales; family: Trebouxiaceae; genus: Myrmecia; taxonRank: Species; **Location:** continent: North America; country: United States of America; countryCode: USA; stateProvince: Utah; county: Wayne County; municipality: Hanksville; locality: Mars Desert Research Station; verbatimLocality: Alluvial plain and dry creekbed directly opposite turnoff to Mars Desert Research Station on Cow Dung Road; verbatimElevation: 1357 m; verbatimLatitude: 38°24'19.2"N; verbatimLongitude: 110°47'20"W; coordinateUncertaintyInMeters: 50; **Identification:** identifiedBy: Hamilton Paul B; dateIdentified: 2016; **Event:** verbatimEventDate: November 24 2014; habitat: Calcareous sandstone; **Record Level:** institutionID: CMN; collectionID: CANL 127965; collectionCode: CANL, UTC; basisOfRecord: Preserved Specimen

##### Notes

Cells elliptical, length 6.5–15.5 μm, width 5–12 μm (Fig. 9). Chloroplast plate-like (1-many), covering most of the cell. Pyrenoid absent. Cell mucilage 0.5–1.5 μm thick. Endophytic, within two species of lichen in family Verrucariaceae (Fig. 9). Earlier descriptions of *M. biatorellae* indicated a broad range in cell size, however a more recent treatment of the genus (Ettl and Gärtner 2014) suggests that cells of *M. biatorellae* are larger than the cells observed here.

#### Cyanobacteria

#### Gloeocapsa sp.

Gloeocapsa sp. [*G*. cf. *coracina* Kütz.]

##### Materials

- **Type status:** Other material. **Occurrence:** recordNumber: 290; recordedBy: Sokoloff, Paul C.; **Taxon:** kingdom: Eubacteria; phylum: Cyanobacteria; class: Cyanophyceae; order: Chroococcales; family: Microcystaceae; genus: Gloeocapsa; **Location:** continent: North America; country: United States of America; countryCode: USA; stateProvince: Utah; county: Wayne County; municipality: Hanksville; locality: Mars Desert Research Station; verbatimLocality: "Comm check" hill, 1.7 km north of Mars Desert Research Station, just west of Cow Dung Road; verbatimElevation: 1371 m; verbatimLatitude: 38°25'3.15"N; verbatimLongitude: 110°46'54.59"W; coordinateUncertaintyInMeters: 50; **Identification:** identifiedBy: Hamilton, Paul B.; dateIdentified: 2016; **Event:** verbatimEventDate: November 22, 2014; habitat: Sandstone at crest of *Artemisia* and *Ephedra* dominated hilltop; **Record Level:** institutionID: CMN; collectionID: CANA 117865; collectionCode: CANA; basisOfRecord: Dried Specimen
- **Type status:** Other material. **Occurrence:** recordNumber: 301; recordedBy: Sokoloff, Paul C.; **Taxon:** kingdom: Eubacteria; phylum: Cyanobacteria; class: Cyanophyceae; order: Chroococcales; family: Microcystaceae; genus: Gloeocapsa; **Location:** continent: North America; country: United States of America; countryCode: USA; stateProvince: Utah; county: Wayne County; municipality: Hanksville; locality: Mars Desert Research Station; verbatimLocality: Alluvial plain and dry creekbed directly opposite turnoff to Mars Desert Research Station on Cow Dung Road; verbatimElevation: 1357 m; verbatimLatitude: 38°24'19.2"N; verbatimLongitude: 110°47'20"W; coordinateUncertaintyInMeters: 50; **Identification:** identifiedBy: Hamilton, Paul B.; dateIdentified: 2016; **Event:** verbatimEventDate: November 24 2014; habitat: Sandstone rubble and sandy plains; **Record Level:** institutionID: CMN; collectionID: CANA 117866; collectionCode: CANA; basisOfRecord: Dried Specimen

##### Notes

Cells small ovals, found as single cells, two cell clusters or in small clusters of daughter cells within thin firm colourless sheaths. Length 2.5–5(6) μm by 1.5–3 μm wide, cell wall sheath thickness 0.8–1.6 μm. Mats of cells form as a collection of single cells or clusters of daughter cells closely packed together. Layer occurring 0.1–0.4 mm below the sandstone surface, or on the underside of quartzite rocks (Fig. 10).

### Fungus Collections

#### Agaricaceae

#### Tulostoma sp.

##### Materials

- **Type status:** Other material. **Occurrence:** recordNumber: 308; recordedBy: Sokoloff, Paul C.; **Taxon:** kingdom: Fungi; phylum: Basidiomycota; class: Agaricomycetes; order: Agaricales; family: Agaricaceae; genus: Tulostoma; taxonRank: Genus; **Location:** continent: North America; country: United States of America; countryCode: USA; stateProvince: Utah; county: Wayne County; municipality: Hanksville; locality: Mars Desert Research Station; verbatimLocality: Sandstone plateau immediately southwest of Mars Desert Research Station, alongside ATV trail; verbatimElevation: 1412 m; verbatimLatitude: 38°24'22.4"N; verbatimLongitude: 110°47'40.3"W; coordinateUncertaintyInMeters: 50; **Identification:** identifiedBy: Redhead, Scott; dateIdentified: 2015; **Event:** verbatimEventDate: November 24, 2014; habitat: Dry conglomerate sandstone; **Record Level:** institutionID: AAFC; collectionCode: DAOM; basisOfRecord: Preserved Specimen

##### Notes

**Figure 11.**
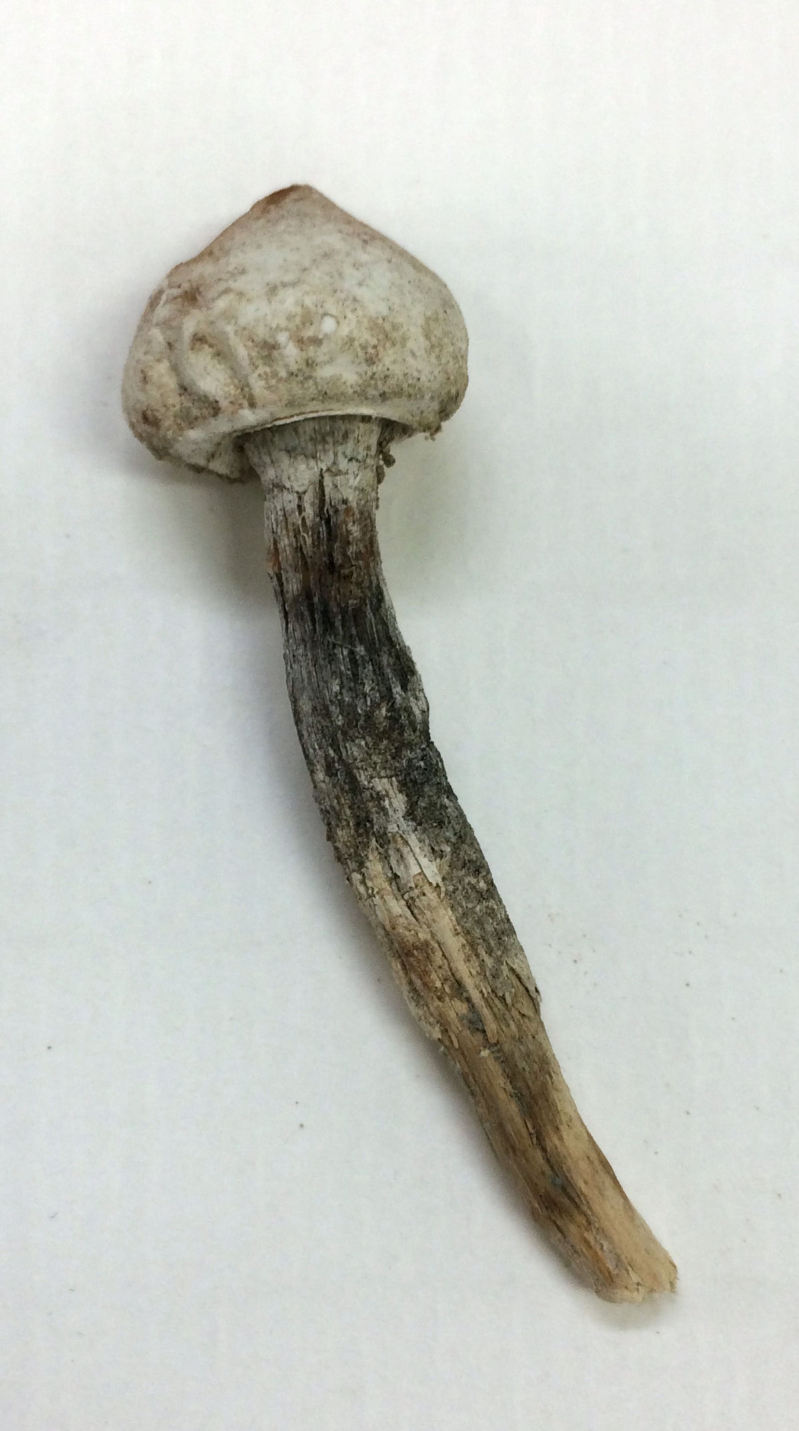
*Tulostoma* sp. (*Sokoloff 308*). Photo by P.C. Sokoloff.

This is a diverse woody mushroom genus common in deserts of the southwestern United States (Hernandez Caffot et al. 2011). This specimen was found growing on sandy soils on top of the plateau immediately southwest of MDRS. Unfortunately, the specimen was too old and degraded for anything other than a generic determination (Scott A. Redhead, personal communication) (Fig. 11).

### Lichen Collections

#### Acarosporaceae

#### Acarospora peliscypha

Th. Fr.

##### Materials

- **Type status:** Other material. **Occurrence:** recordNumber: 286; recordedBy: Sokoloff, Paul C.; **Taxon:** scientificName: *Acarospora peliscypha* Th. Fr.; kingdom: Fungi; phylum: Ascomycota; class: Lecanoromycetes; order: Acarosporales; family: Acarosporaceae; genus: Acarospora; specificEpithet: peliscypha; taxonRank: Species; scientificNameAuthorship: Th. Fr.; **Location:** continent: North America; country: United States of America; countryCode: USA; stateProvince: Utah; county: Wayne County; municipality: Hanksville; locality: Mars Desert Research Station; verbatimLocality: "Comm check" hill, 1.7 km north of Mars Desert Research Station, just west of Cow Dung Road; verbatimElevation: 1371 m; verbatimLatitude: 38°25'3.15"N; verbatimLongitude: 110°46'54.59"W; coordinateUncertaintyInMeters: 50; **Identification:** identifiedBy: Freebury, Colin E.; dateIdentified: 2015; identificationRemarks: Additional specimens examined: Utah: Grand County, Sharnoff & Sharnoff 1635.01 (CANL), on partially calcareous sandstone. Colorado: Boulder County, Shushan & Weber S3363 (CANL), on acidic sandstone.; **Event:** verbatimEventDate: November 22, 2014; habitat: Conglomerate sandstone hilltop dominated by *Artemisia* and *Ephedra*; **Record Level:** institutionID: CMN; collectionID: CANL 127960; collectionCode: CANL, UTC; basisOfRecord: Preserved Specimen

##### Notes

**Figure 12.**
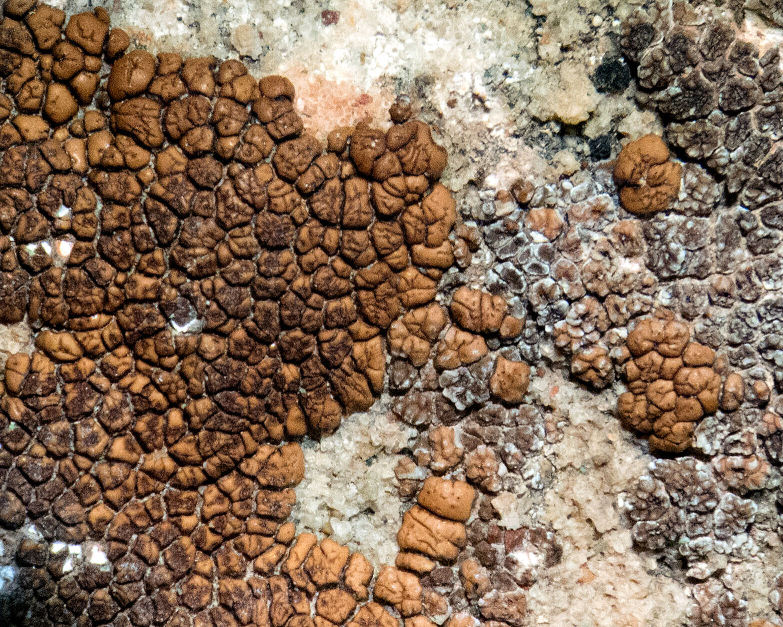
*Acarospora peliscypha* (*Sokoloff 286*). Habit. On partially calcareous sandstone. Photo by C.E. Freebury.

*Acarospora peliscypha* is an epruinose species; the whitish material on this specimen, as shown on the right side of Fig. 12, is not pruina but represents an accumulation of necrotic material. Although we were unable to find previous published reports of this species from Utah, we did examine one previous collection from that state. This species has been described from the Sonoran Desert as growing on granite only (Knudsen 2007), however we found the species on partially calcareous sandstone. 

**Supplemental File**: CANL 127960 (Suppl. material 1).

#### Acarospora rosulata

(Th. Fr.) H. Magn.

##### Materials

- **Type status:** Other material. **Occurrence:** recordNumber: 303; recordedBy: Sokoloff, Paul C.; **Taxon:** scientificName: *Acarospora rosulata* (Th. Fr.) H. Magn.; kingdom: Fungi; phylum: Ascomycota; class: Lecanoromycetes; order: Acarosporales; family: Acarosporaceae; genus: Acarospora; specificEpithet: rosulata; taxonRank: Species; scientificNameAuthorship: (Th. Fr.) H. Magn.; **Location:** continent: North America; country: United States of America; countryCode: USA; stateProvince: Utah; county: Wayne County; municipality: Hanksville; locality: Mars Desert Research Station; verbatimLocality: Alluvial plain and dry creekbed directly opposite turnoff to Mars Desert Research Station on Cow Dung Road; verbatimElevation: 1357 m; verbatimLatitude: 38°24'19.2"N; verbatimLongitude: 110°47'20"W; coordinateUncertaintyInMeters: 50; **Identification:** identifiedBy: Freebury, Colin E.; dateIdentified: 2015; identificationReferences: Knudsen et al. (2010); **Event:** verbatimEventDate: November 24, 2014; habitat: Sandstone rubble on sandy plain; **Record Level:** institutionID: CMN; collectionID: CANL 127968; collectionCode: CANL; basisOfRecord: Preserved Specimen

##### Notes

Medulla KC+ pink, C+ pink (gyrophoric acid); on limestone. *Acarospora rosulata* has been previously reported from Utah as *Acarospora bullata* by Newberry and St. Clair (1991) (Sevier County), Leavitt and St. Clair (2008) (Wayne County) and Knudsen et al. (2010) (San Juan County and Sevier County). 

**Supplemental File**: CANL 127968 (Suppl. material 2).

#### Acarospora stapfiana

(Müll. Arg.) Hue

##### Materials

- **Type status:** Other material. **Occurrence:** recordNumber: 270; recordedBy: Sokoloff, Paul C.; **Taxon:** scientificName: *Acarospora stapfiana* (Müll. Arg.) Hue; kingdom: Fungi; phylum: Ascomycota; class: Lecanoromycetes; order: Acarosporales; family: Acarosporaceae; genus: Acarospora; specificEpithet: stapfiana; taxonRank: Species; scientificNameAuthorship: (Müll. Arg.) Hue; **Location:** continent: North America; country: United States of America; countryCode: USA; stateProvince: Utah; county: Wayne County; municipality: Hanksville; locality: Mars Desert Research Station; verbatimLocality: Dry streambed approx 500 m northeast of Mars Desert Research Station "hab"; verbatimElevation: 1371 m; verbatimLatitude: 38°24'27.7"N; verbatimLongitude: 110°47'20"W; coordinateUncertaintyInMeters: 50; **Identification:** identifiedBy: Freebury, Colin E.; dateIdentified: 2015; identificationRemarks: Parasitic on *C. trachyphylla*; **Event:** verbatimEventDate: November 20, 2014; habitat: Rock-strewn hill with bird-perch boulder; **Record Level:** institutionID: CMN; collectionID: CANL 127958; collectionCode: CANL, UTC; basisOfRecord: Preserved Specimen

##### Notes

This lichen was encountered growing on sandstone and parasitically on *Caloplaca trachyphylla* (Fig. 13) in the shade of a large rock due northeast of MDRS. *Acarospora stapfiana* has been previously reported from Capitol Reef National Park (St. Clair et al. 1995).

**Supplemental File**: CANL 127958 (Suppl. material 3).

#### Acarospora strigata

(Nyl.) Jatta

##### Materials

- **Type status:** Other material. **Occurrence:** recordNumber: 248; recordedBy: Sokoloff, Paul C.; **Taxon:** scientificName: *Acarospora strigata* (Nyl.) Jatta; kingdom: Fungi; phylum: Ascomycota; class: Lecanoromycetes; order: Acarosporales; family: Acarosporaceae; genus: Acarospora; specificEpithet: strigata; taxonRank: Species; scientificNameAuthorship: (Nyl.) Jatta; **Location:** continent: North America; country: United States of America; countryCode: USA; stateProvince: Utah; county: Wayne County; municipality: Hanksville; locality: Mars Desert Research Station; verbatimLocality: Vicinity of the Mars Desert Research Station, Hanksville, Utah, 500 m radius of "hab"; verbatimElevation: 1371 m; verbatimLatitude: 38°24'23.2"N; verbatimLongitude: 110°47'31.1"W; coordinateUncertaintyInMeters: 50; **Identification:** identifiedBy: Freebury, Colin E.; dateIdentified: 2015; **Event:** verbatimEventDate: November 17, 2014; habitat: Conglomerate sandstone hilltop; **Record Level:** institutionID: CMN; collectionID: CANL 127953; collectionCode: CANL, UTC; basisOfRecord: Preserved Specimen
- **Type status:** Other material. **Occurrence:** recordNumber: 288; recordedBy: Sokoloff, Paul C.; **Taxon:** scientificName: *Acarospora strigata* (Nyl.) Jatta; kingdom: Fungi; phylum: Ascomycota; class: Lecanoromycetes; order: Acarosporales; family: Acarosporaceae; genus: Acarospora; specificEpithet: strigata; taxonRank: Species; scientificNameAuthorship: (Nyl.) Jatta; **Location:** continent: North America; country: United States of America; countryCode: USA; stateProvince: Utah; county: Wayne County; municipality: Hanksville; locality: Mars Desert Research Station; verbatimLocality: "Comm check" hill, 1.7 km north of Mars Desert Research Station, just west of Cow Dung Road; verbatimElevation: 1371 m; verbatimLatitude: 38°25'3.15"N; verbatimLongitude: 110°46'54.59"W; coordinateUncertaintyInMeters: 50; **Identification:** identifiedBy: Freebury, Colin E.; dateIdentified: 2015; **Event:** verbatimEventDate: November 22, 2014; habitat: Conglomerate sandstone hilltop dominated by *Artemisia* and *Ephedra*; **Record Level:** institutionID: CMN; institutionCode: CANL 127962; collectionCode: CANL; basisOfRecord: Preserved Specimen
- **Type status:** Other material. **Occurrence:** recordNumber: 304; recordedBy: Sokoloff, Paul C.; **Taxon:** scientificName: *Acarospora strigata* (Nyl.) Jatta; kingdom: Fungi; phylum: Ascomycota; class: Lecanoromycetes; order: Acarosporales; family: Acarosporaceae; genus: Acarospora; specificEpithet: strigata; taxonRank: Species; scientificNameAuthorship: (Nyl.) Jatta; **Location:** continent: North America; country: United States of America; countryCode: USA; stateProvince: Utah; county: Wayne County; municipality: Hanksville; locality: Mars Desert Research Station; verbatimLocality: Alluvial plain and dry creekbed directly opposite turnoff to Mars Desert Research Station on Cow Dung Road; verbatimElevation: 1357 m; verbatimLatitude: 38°24'19.2"N; verbatimLongitude: 110°47'20"W; coordinateUncertaintyInMeters: 50; **Identification:** identifiedBy: Freebury, Colin E.; dateIdentified: 2015; **Event:** verbatimEventDate: November 24, 2014; habitat: Sandstone rubble on sandy plain; **Record Level:** institutionID: CMN; collectionID: CANL 127969; collectionCode: CANL, UTC; basisOfRecord: Preserved Specimen
- **Type status:** Other material. **Occurrence:** recordNumber: 306; recordedBy: Sokoloff, Paul C.; **Taxon:** scientificName: *Acarospora strigata* (Nyl.) Jatta; kingdom: Fungi; phylum: Ascomycota; class: Lecanoromycetes; order: Acarosporales; family: Acarosporaceae; genus: Acarospora; specificEpithet: strigata; taxonRank: Species; scientificNameAuthorship: (Nyl.) Jatta; **Location:** continent: North America; country: United States of America; countryCode: USA; stateProvince: Utah; county: Wayne County; municipality: Hanksville; locality: Mars Desert Research Station; verbatimLocality: Sandstone plateau immediately southwest of Mars Desert Research Station, alongside ATV trail; verbatimElevation: 1412 m; verbatimLatitude: 38°24'22.4"N; verbatimLongitude: 110°47'40.3"W; coordinateUncertaintyInMeters: 50; **Identification:** identifiedBy: Freebury, Colin E.; dateIdentified: 2015; **Event:** verbatimEventDate: November 24, 2014; habitat: Rock-strewn hill; **Record Level:** institutionID: CMN; collectionID: CANL 127952; collectionCode: CANL, UTC; basisOfRecord: Preserved Specimen

##### Notes

This greyish-white crustose lichen was commonly encountered growing on sandstone rocks surrounding MDRS (Fig. 14). The species was previously reported from eastern Wayne County by St. Clair et al. (1991).

**Supplemental Files**: CANL 127953 (Suppl. material 4), CANL 127962 (Suppl. material 5), CANL 127969 (Suppl. material 6), CANL 127966 (Suppl. material 7).

#### Polysporina gyrocarpa

(H. Magn.) N. S. Golubk.

##### Materials

- **Type status:** Other material. **Occurrence:** recordNumber: 247; recordedBy: Sokoloff, Paul C.; **Taxon:** scientificName: *Polysporina gyrocarpa* (H. Magn.) N. S. Golubk.; kingdom: Fungi; phylum: Ascomycota; class: Lecanoromycetes; order: Acarosporales; family: Acarosporaceae; genus: Polysporina; specificEpithet: gyrocarpa; taxonRank: Species; scientificNameAuthorship: (H. Magn.) N. S. Golubk.; **Location:** continent: North America; country: United States of America; countryCode: USA; stateProvince: Utah; county: Wayne County; municipality: Hanksville; locality: Mars Desert Research Station; verbatimLocality: Vicinity of the Mars Desert Research Station, Hanksville, Utah, 500 m radius of "hab".; verbatimElevation: 1371 m; verbatimLatitude: 38°24'23.2"N; verbatimLongitude: 110°47'31.1"W; coordinateUncertaintyInMeters: 50; **Identification:** identifiedBy: Freebury, Colin E.; dateIdentified: 2015; identificationReferences: Knudsen and Kocourková (2009); **Event:** verbatimEventDate: November 17, 2014; habitat: Sandstone rubble on sandy plain; **Record Level:** institutionID: CMN; collectionID: CANL 127952; collectionCode: CANL, UTC; basisOfRecord: Preserved Specimen
- **Type status:** Other material. **Occurrence:** recordNumber: 250; recordedBy: Sokoloff, Paul C.; **Taxon:** scientificName: *Polysporina gyrocarpa* (H. Magn.) N. S. Golubk.; kingdom: Fungi; phylum: Ascomycota; class: Lecanoromycetes; order: Acarosporales; family: Acarosporaceae; genus: Polysporina; specificEpithet: gyrocarpa; taxonRank: Species; scientificNameAuthorship: (H. Magn.) N. S. Golubk.; **Location:** continent: North America; country: United States of America; countryCode: USA; stateProvince: Utah; county: Wayne County; municipality: Hanksville; locality: Mars Desert Research Station; verbatimLocality: Vicinity of the Mars Desert Research Station, Hanksville, Utah, 500 m radius of "hab"; verbatimElevation: 1371 m; verbatimLatitude: 38°24'23.2"N; verbatimLongitude: 110°47'31.1"W; coordinateUncertaintyInMeters: 50; **Identification:** identifiedBy: Freebury, Colin E.; dateIdentified: 2015; **Event:** verbatimEventDate: November 17, 2014; habitat: Sandstone rubble on sandy plain; **Record Level:** institutionID: CMN; collectionID: CANL 127954; collectionCode: CANL; basisOfRecord: Preserved Specimen
- **Type status:** Other material. **Occurrence:** recordNumber: 286b; recordedBy: Sokoloff, Paul C.; **Taxon:** scientificName: *Polysporina gyrocarpa* (H. Magn.) N. S. Golubk.; kingdom: Fungi; phylum: Ascomycota; class: Lecanoromycetes; order: Acarosporales; family: Acarosporaceae; genus: Polysporina; specificEpithet: gyrocarpa; taxonRank: Species; scientificNameAuthorship: (H. Magn.) N. S. Golubk.; **Location:** continent: North America; country: United States of America; countryCode: USA; stateProvince: Utah; county: Wayne County; municipality: Hanksville; locality: Mars Desert Research Station; verbatimLocality: "Comm check" hill, 1.7 km northeast of Mars Desert Research Station, just west of Cow Dung Road; verbatimElevation: 1371 m; verbatimLatitude: 38°25'3.15"N; verbatimLongitude: 110°46'54.59"W; coordinateUncertaintyInMeters: 50; **Identification:** identifiedBy: Freebury, Colin E.; dateIdentified: 2015; **Event:** verbatimEventDate: November 22, 2014; habitat: Conglomerate sandstone hilltop dominated by *Artemisia* and *Ephedra*; **Record Level:** institutionID: CMN; collectionID: CANL 127970; collectionCode: CANL; basisOfRecord: Preserved Specimen
- **Type status:** Other material. **Occurrence:** recordNumber: 289; recordedBy: Sokoloff, Paul C.; **Taxon:** scientificName: *Polysporina gyrocarpa* (H. Magn.) N. S. Golubk.; kingdom: Fungi; phylum: Ascomycota; class: Lecanoromycetes; order: Acarosporales; family: Acarosporaceae; genus: Polysporina; specificEpithet: gyrocarpa; taxonRank: Species; scientificNameAuthorship: (H. Magn.) N. S. Golubk.; **Location:** continent: North America; country: United States of America; countryCode: USA; stateProvince: Utah; county: Wayne County; municipality: Hanksville; locality: Mars Desert Research Station; verbatimLocality: Along Cow Dung Road north of the Mars Desert Research Station; verbatimElevation: 1371 m; verbatimLatitude: 38°25'19.35"N; verbatimLongitude: 110°47'5.4"W; coordinateUncertaintyInMeters: 50; **Identification:** identifiedBy: Freebury, Colin E.; dateIdentified: 2015; **Event:** verbatimEventDate: November 22, 2014; habitat: Moist desert flats; **Record Level:** institutionID: CMN; collectionID: CANL 127963; collectionCode: CANL, UTC; basisOfRecord: Preserved Specimen

##### Notes

**Figure 15.**
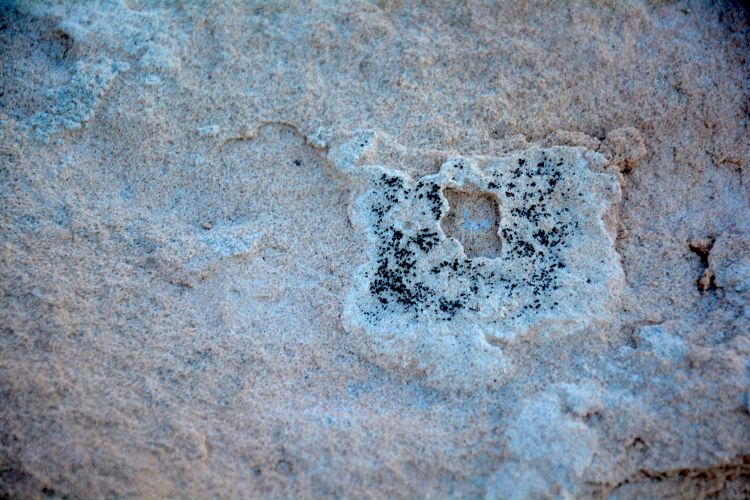
*Polysporina gyrocarpa* (*Sokoloff 247*). Habit. Photo by P.C. Sokoloff.

This species was commonly encountered growing on sandstone rocks and outcrops surrounding MDRS; the black, dot-like apothecia stand out well on tan-coloured sandstone (Fig. 15). *Polysporina gyrocarpa* was previously reported from Wayne County as *Sarcogyne oligospora* H. Magn. (St. Clair et al. 1991) and as *Polysporina oligospora* (H. Magn.) K. Knudsen (Knudsen and Lendemer 2005), and from the San Rafael Swell (Knudsen and Kocourková 2009).

**Supplemental Files**: CANL 127952 (Suppl. material 8), CANL 127954 (Suppl. material 9), CANL 127963 (Suppl. material 10), CANL 127970 (Suppl. material 11).

#### Candelariaceae

#### Candelariella rosulans

(Müll. Arg.) Zahlbr.

##### Materials

- **Type status:** Other material. **Occurrence:** recordNumber: 251; recordedBy: Sokoloff, Paul C.; reproductiveCondition: without fruiting bodies; **Taxon:** scientificName: Candelariella cf. rosulans (Müll. Arg.) Zahlbr.; kingdom: Fungi; phylum: Ascomycota; class: Lecanoromycetes; order: Candelariales; family: Candelariaceae; genus: Candelariella; specificEpithet: rosulans; taxonRank: Species; scientificNameAuthorship: (Mull. Arg.) Zahlbr.; **Location:** continent: North America; country: United States of America; countryCode: USA; stateProvince: Utah; county: Wayne County; municipality: Hanksville; locality: Mars Desert Research Station; verbatimLocality: Vicinity of the Mars Desert Research Station, Hanksville, Utah, 500 m radius of "hab"; verbatimElevation: 1371 m; verbatimLatitude: 38°24'23.2"N; verbatimLongitude: 110°47'31.1"W; coordinateUncertaintyInMeters: 50; **Identification:** identifiedBy: Freebury, Colin E.; dateIdentified: 2015; identificationQualifier: cf.; **Event:** verbatimEventDate: November 17, 2014; habitat: Rocky soil; **Record Level:** institutionID: CMN; collectionID: CANL 127955; collectionCode: CANL, UTC; basisOfRecord: Preserved Specimen
- **Type status:** Other material. **Occurrence:** recordNumber: 288b; recordedBy: Sokoloff, Paul C.; **Taxon:** scientificName: *Candelariella rosulans* (Müll. Arg.) Zahlbr.; kingdom: Fungi; phylum: Ascomycota; class: Lecanoromycetes; order: Candelariales; family: Candelariaceae; genus: Candelariella; specificEpithet: rosulans; taxonRank: Species; scientificNameAuthorship: (Mull. Arg.) Zahlbr.; **Location:** continent: North America; country: United States of America; countryCode: USA; stateProvince: Utah; county: Wayne County; municipality: Hanksville; locality: Mars Desert Research Station; verbatimLocality: "Comm check" hill, 1.7 km northeast of Mars Desert Research Station, just west of Cow Dung Road; verbatimElevation: 1371 m; verbatimLatitude: 38°25'3.15"N; verbatimLongitude: 110°46'54.59"W; coordinateUncertaintyInMeters: 50; **Identification:** identifiedBy: Freebury, Colin E.; dateIdentified: 2015; identificationReferences: Westberg (2007); **Event:** verbatimEventDate: November 22, 2014; habitat: Conglomerate sandstone hilltop dominated by *Artemisia* and *Ephedra*; **Record Level:** institutionID: CMN; collectionID: CANL 127971; collectionCode: CANL; basisOfRecord: Preserved Specimen

##### Notes

**Figure 16.**
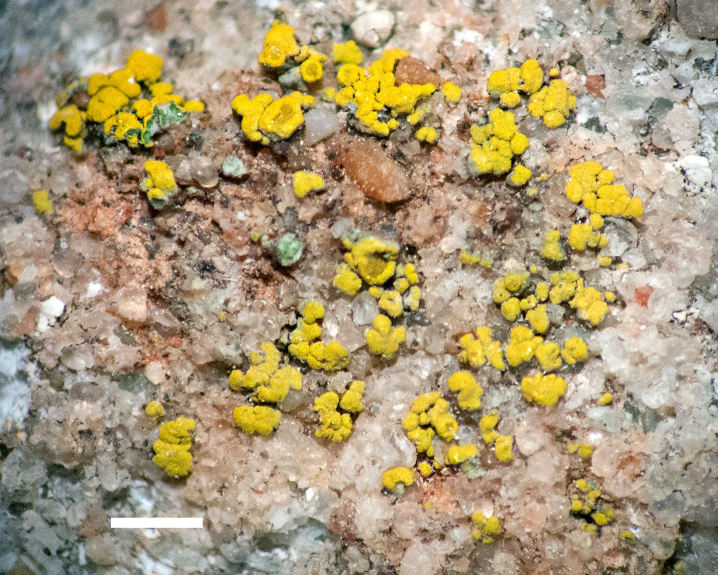
*Candelariella rosulans* (*Sokoloff 288b*). Habit. Scale bar = 1 mm. Photo by C.E. Freebury.

Asci 8-spored, ascospores narrowly ellipsoid to oblong, 13-15 × (4-)5(-6) µm. This yellow lichen species (Fig. 16) is known from throughout much of western North America (Westberg 2007). It was previously reported from Wayne County by Westberg (2007) and Leavitt and St. Clair (2008).

**Supplemental Files**: CANL 127955 (Suppl. material 12), CANL 127971 (Suppl. material 13).

#### Collemataceae

#### Enchylium tenax

(Sw.) Gray

##### Materials

- **Type status:** Other material. **Occurrence:** recordNumber: 305b; recordedBy: Sokoloff Paul C.; reproductiveCondition: Apothecia absent; **Taxon:** scientificName: *Enchylium tenax* (Sw.) Gray; kingdom: Fungi; phylum: Ascomycota; class: Lecanoromycetes; order: Lecanorales; family: Collemataceae; genus: Enchylium; specificEpithet: tenax; taxonRank: Species; scientificNameAuthorship: (Sw.) Gray; vernacularName: Soil jelly lichen; **Location:** continent: North America; country: United States of America; countryCode: USA; stateProvince: Utah; county: Wayne County; municipality: Hanksville; locality: Mars Desert Research Station; verbatimLocality: Alluvial plain and dry creekbed directly opposite turnoff to Mars Desert Research Station on Cow Dung Road; verbatimElevation: 1357 m; verbatimLatitude: 38°24'19.2"N; verbatimLongitude: 110°47'20"W; geodeticDatum: WGS84; coordinateUncertaintyInMeters: 50; **Identification:** identifiedBy: Brodo, I. M.; dateIdentified: 2016; identificationRemarks: Additional specimen examined: Utah: Emery County, San Rafael Swell; on soil. S. & S.D. Sharnoff 1181.21 (CANL).; **Event:** verbatimEventDate: November 24, 2014; habitat: Sandstone rubble on sandy plain; **Record Level:** institutionID: CMN; collectionID: CANL 127973; collectionCode: CANL; basisOfRecord: Preserved Specimen

##### Notes

**Figure 17.**
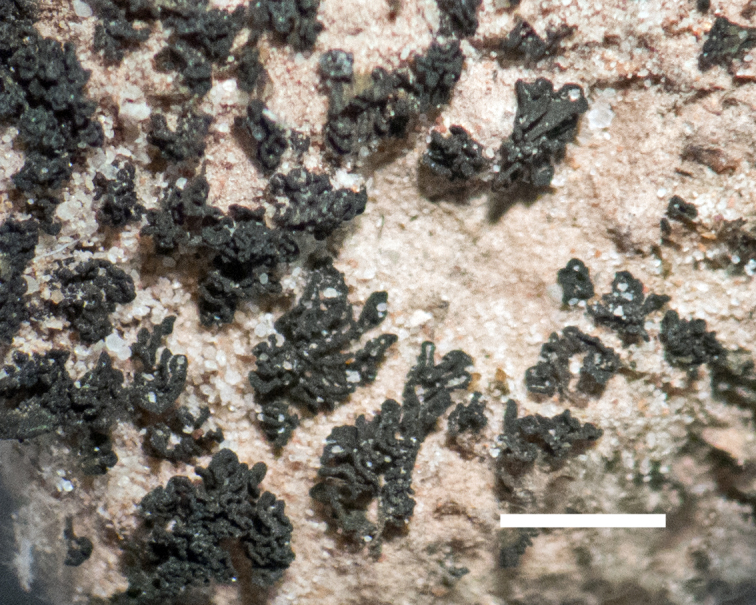
*Enchylium tenax* (*Sokoloff 305b*). Habit. Scale bar = 3 mm. Photo by C.E. Freebury.

Small sample, ca. 2 cm diam.; on sandy soil. *Enchylium tenax* (syn. *Collema tenax* (Sw.) Ach.) (Otálora et al. 2013) is a cosmopolitan species that is widely variable in terms of habit, color and isidia (Schultz et al. 2004). This particular specimen (Fig. 17) has somewhat ascending lobes and lacks apothecia. The species was previously reported as *Collema tenax* from the San Rafael Swell by Rajvanshi et al. (1998).

**Supplemental File**: CANL 127937 (Suppl. material 14.

#### Lecanoraceae

#### Lecanora garovaglii

(Körber) Zahlbr.

##### Materials

- **Type status:** Other material. **Occurrence:** recordNumber: 287; recordedBy: Sokoloff, Paul C.; **Taxon:** scientificName: *Lecanora garovaglii* (Körber) Zahlbr.; kingdom: Fungi; phylum: Ascomycota; class: Lecanoromycetes; order: Lecanorales; family: Lecanoraceae; genus: Lecanora; specificEpithet: garovaglii; taxonRank: Species; scientificNameAuthorship: (Körber) Zahlbr.; **Location:** continent: North America; country: United States of America; countryCode: USA; stateProvince: Utah; county: Wayne County; municipality: Hanksville; locality: Mars Desert Research Station; verbatimLocality: "Comm check" hill, 1.7 km north of Mars Desert Research Station, just west of Cow Dung Road; verbatimElevation: 1371 m; verbatimLatitude: 38°25'3.15"N; verbatimLongitude: 110°46'54.59"W; coordinateUncertaintyInMeters: 50; **Identification:** identifiedBy: Freebury, Colin E.; dateIdentified: 2015; identificationRemarks: Additional specimen examined: Utah: Carbon County, B. D. Ryan 18077, on sandstone (CANL).; **Event:** verbatimEventDate: November 22, 2014; habitat: Conglomerate sandstone hilltop dominated by *Artemisia* and *Ephedra*; **Record Level:** institutionID: CMN; collectionID: CANL 127961; collectionCode: CANL, UTC; basisOfRecord: Preserved Specimen

##### Notes

**Figure 18.**
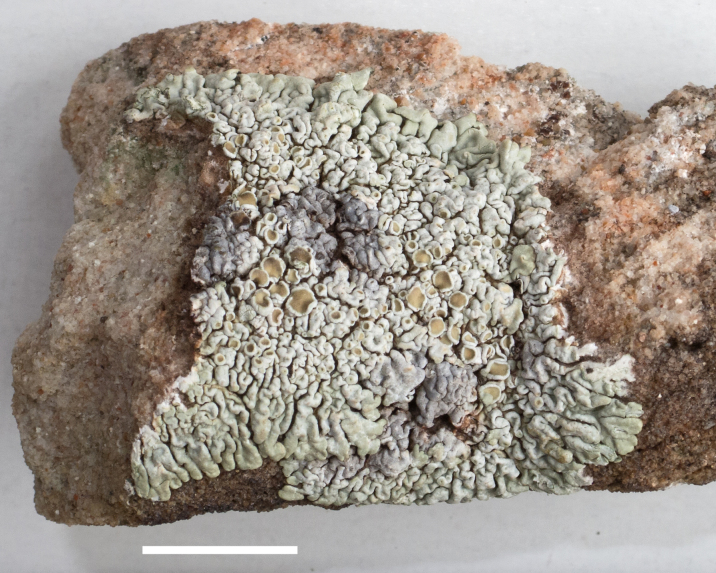
*Lecanora garovaglii* (*Sokoloff 287*). Habit. Scale bar = 1 cm. Photo by C.E. Freebury.

Cortex KC+ gold (usnic & isousnic acids). *Lecanora garovaglii* (Fig. 18) is common throughout the semi-arid regions of central North America (Brodo et al. 2001). It has been previously reported from Boulder Mt. Plateau, Wayne County by Leavitt and St. Clair (2008).

**Supplemental File**: CANL 127961 (Suppl. material 15).

#### Physicaceae

#### Buellia abstracta

(Nyl.) H. Olivier

##### Materials

- **Type status:** Other material. **Occurrence:** recordNumber: 271; recordedBy: Sokoloff, Paul C.; **Taxon:** scientificName: *Buellia abstracta* (Nyl.) H. Olivier; kingdom: Fungi; phylum: Ascomycota; class: Lecanoromycetes; order: Teloschistales; family: Physciaceae; genus: Buellia; specificEpithet: abstracta; taxonRank: Species; scientificNameAuthorship: (Nyl.) H. Olivier; **Location:** continent: North America; country: United States of America; countryCode: USA; stateProvince: Utah; county: Wayne County; municipality: Hanksville; locality: Mars Desert Research Station; verbatimLocality: Dry streambed approx 500 m northeast of Mars Desert Research Station "hab"; verbatimElevation: 1371 m; verbatimLatitude: 38°24'27.7"N; verbatimLongitude: 110°47'20"W; coordinateUncertaintyInMeters: 50; **Identification:** identifiedBy: Freebury, Colin E.; dateIdentified: 2015; **Event:** verbatimEventDate: November 20, 2014; habitat: Hills with scattered sandstone boulders; **Record Level:** institutionID: CMN; collectionID: CANL 127959; collectionCode: CANL, UTC; basisOfRecord: Preserved Specimen

##### Notes

*Buellia abstracta* has sometimes been treated incorrectly as *Buellia sequax* (Nyl.) Zahlbr. (Bungartz et al. 2007, Giralt et al. 2011), and it has been reported as such from southwestern Utah (Bungartz et al. 2004). We have been unable to find reports of the species from the San Rafael Swell or other nearby areas.

**Supplemental File**: CANL 127959 (Suppl. material 16).

#### Teloschistaceae

#### Caloplaca trachyphylla

(Tuck.) Zahlbr.

##### Materials

- **Type status:** Other material. **Occurrence:** recordNumber: 252; recordedBy: Sokoloff, Paul C.; **Taxon:** scientificName: *Caloplaca trachyphylla* (Tuck.) Zahlbr.; kingdom: Fungi; phylum: Ascomycota; class: Lecanoromycetes; order: Teloschistales; family: Teloschistaceae; genus: Caloplaca; specificEpithet: trachyphylla; taxonRank: Species; scientificNameAuthorship: (Tuck.) Zahlbr.; **Location:** continent: North America; country: United States of America; countryCode: USA; stateProvince: Utah; county: Wayne County; municipality: Hanksville; locality: Mars Desert Research Station; verbatimLocality: Vicinity of the Mars Desert Research Station, Hanksville, Utah, 500 m radius of "hab"; verbatimElevation: 1371 m; verbatimLatitude: 38°24'23.2"N; verbatimLongitude: 110°47'31.1"W; coordinateUncertaintyInMeters: 50; **Identification:** identifiedBy: Freebury, Colin E.; dateIdentified: 2015; **Event:** verbatimEventDate: November 17, 2014; habitat: Rocks on soil within a covered passageway; **Record Level:** institutionID: CMN; collectionID: CANL 127956; collectionCode: CANL, UTC; basisOfRecord: Preserved Specimen
- **Type status:** Other material. **Occurrence:** recordNumber: 269; recordedBy: Sokoloff, Paul C.; **Taxon:** scientificName: *Caloplaca trachyphylla* (Tuck.) Zahlbr.; kingdom: Fungi; phylum: Ascomycota; class: Lecanoromycetes; order: Teloschistales; family: Teloschistaceae; genus: Caloplaca; specificEpithet: trachyphylla; taxonRank: Species; scientificNameAuthorship: (Tuck.) Zahlbr.; **Location:** continent: North America; country: United States of America; countryCode: USA; stateProvince: Utah; county: Wayne County; municipality: Hanksville; locality: Mars Desert Research Station; verbatimLocality: Dry streambed approx. 500 m northeast of Mars Desert Research Station "hab"; verbatimElevation: 1371 m; verbatimLatitude: 38°24'27.7"N; verbatimLongitude: 110°47'20"W; coordinateUncertaintyInMeters: 50; **Identification:** identifiedBy: Freebury, Colin E.; dateIdentified: 2015; **Event:** verbatimEventDate: November 20, 2014; habitat: Hilltop sandstone boulder, a bird perch; **Record Level:** institutionID: CMN; collectionID: CANL 127957; collectionCode: CANL, UTC; basisOfRecord: Preserved Specimen

##### Notes

This species was one of the most conspicuous lichens encountered on EVAs (Fig. 19). One specimen (*Sokoloff 252*) was collected on a rock inside the passageway connecting the MDRS "hab" with the Musk Observatory. Though we follow Brodo et al. (2001) in treating this species as a member of *Caloplaca*, some authors treat this species as *Xanthomendoza trachyphylla* (Tuck.) Frödén, Arup & Søchting (Arup et al. 2013). *Caloplaca trachyphylla* is common in western North America (Brodo et al. 2001).

**Supplemental Files**: CANL 127956 (Suppl. material 17), CANL 127957 (Suppl. material 18).

#### Verrucariaceae

#### Heteroplacidium compactum

(A. Massal.) Gueidan & Cl. Roux

##### Materials

- **Type status:** Other material. **Occurrence:** recordNumber: 296; recordedBy: Sokoloff, Paul C.; **Taxon:** scientificName: *Heteroplacidium compactum* (A. Massal.) Gueidan & Cl. Roux; kingdom: Fungi; phylum: Ascomycota; class: Eurotiomycetes; order: Verrucariales; family: Verrucariaceae; genus: Heteroplacidium; specificEpithet: compactum; taxonRank: Species; scientificNameAuthorship: (A. Massal.) Gueidan & Cl. Roux; **Location:** continent: North America; country: United States of America; countryCode: USA; stateProvince: Utah; county: Wayne County; municipality: Hanksville; locality: Mars Desert Research Station; verbatimLocality: Roadside along ATV trail 2 km east of Mars Desert Research Station; verbatimElevation: 1348 m; verbatimLatitude: 38°24'53.8"N; verbatimLongitude: 110°46'18"W; coordinateUncertaintyInMeters: 50; **Identification:** identifiedBy: Freebury, Colin E.; dateIdentified: 2015; identificationReferences: Breuss, O. (2007); identificationRemarks: Additional specimen examined: Saskatchewan: Grasslands National Park: Freebury 1335, independent on sandstone, parasitically on *Verrucaria inficiens* (CANL).; **Event:** verbatimEventDate: November 23, 2014; habitat: Sandstone rubble on sandy plain; **Record Level:** institutionID: CMN; collectionID: CANL 127964; collectionCode: CANL, UTC; basisOfRecord: Preserved Specimen

##### Notes

**Figure 20.**
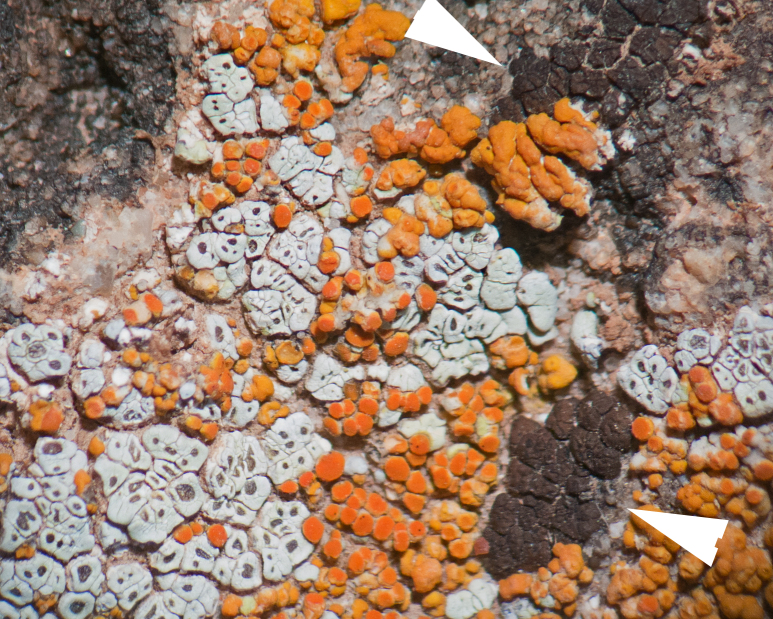
*Heteroplacidium compactum* (arrows) (*Sokoloff 296*), growing among *Caloplaca trachyphylla* (orange). The whitish (pruinose) lichen is *Acarospora stapfiana*, which is also a facultative parasite. Photo by C.E. Freebury.

*Heteroplacidium compactum* is widely distributed worldwide. It was previously reported from Utah as *Catapyrenium compactum* (A. Massal.) R. Sant. by St. Clair et al. (1991). This lichen begins as a parasite on other lichens, and then grows independently. Fig. 20 shows our specimen growing partly scattered among and likely parasitic on *Caloplaca trachyphylla*. 

**Supplemental File**: CANL 127964 (Suppl. material 19).

#### Placidium acarosporoides

(Zahlbr.) Breuss

##### Materials

- **Type status:** Other material. **Occurrence:** recordNumber: 305; recordedBy: Sokoloff, Paul C.; **Taxon:** scientificName: *Placidium acarosporoides* (Zahlbr.) Breuss; kingdom: Fungi; phylum: Ascomycota; class: Eurotiomycetes; order: Verrucariales; family: Verrucariaceae; genus: Placidium; specificEpithet: acarosporoides; taxonRank: Species; scientificNameAuthorship: (Zahlbr.) Breuss; **Location:** continent: North America; country: United States of America; countryCode: USA; stateProvince: Utah; county: Wayne County; municipality: Hanksville; locality: Mars Desert Research Station; verbatimLocality: Alluvial plain and dry creekbed directly opposite turnoff to Mars Desert Research Station on Cow Dung Road; verbatimElevation: 1357 m; verbatimLatitude: 38°24'19.2"N; verbatimLongitude: 110°47'20"W; coordinateUncertaintyInMeters: 50; **Identification:** identifiedBy: Freebury, C.E.; dateIdentified: 2015; identificationReferences: Breuss (2002), Thomson (1987); identificationRemarks: Additional specimen examined: California: Riverside Co., Mojave Desert: S. & S.D. Sharnoff 1598.25, on granitic rock (CANL).; **Event:** verbatimEventDate: November 24, 2014; habitat: Sandstone rubble on sandy plain; **Record Level:** institutionID: CMN; collectionID: CANL 127965; collectionCode: CANL, UTC; basisOfRecord: Preserved Specimen

##### Notes

*Placidium acarosporoides* was found growing on calcareous sandstone in the vicinity of MDRS (Fig. 21). It has been reported previously from eastern Wayne County by Thomson (1987) as *Catapyrenium acarosporoides* (Zahlbr.) J.W. Thomson.

**Supplemental File**: CANL 127965 (Suppl. material 20).

#### Placidium lachneum

(Ach.) Breuss

##### Materials

- **Type status:** Other material. **Occurrence:** recordNumber: 305c; recordedBy: Sokoloff, Paul C.; **Taxon:** scientificName: *Placidium lachneum* (Ach.) Breuss; kingdom: Fungi; phylum: Ascomycota; class: Eurotiomycetes; order: Verrucariales; family: Verrucariaceae; genus: Placidium; specificEpithet: lachneum; taxonRank: Species; scientificNameAuthorship: (Ach.) Breuss; **Location:** continent: North America; country: United States of America; countryCode: USA; stateProvince: Utah; county: Wayne County; municipality: Hanksville; locality: Mars Desert Research Station; verbatimLocality: Alluvial plain and dry creekbed directly opposite turnoff to Mars Desert Research Station on Cow Dung Road; verbatimElevation: 1357 m; verbatimLatitude: 38°24'19.2"N; verbatimLongitude: 110°47'20"W; geodeticDatum: WGS84; coordinateUncertaintyInMeters: 50; **Identification:** identifiedBy: Freebury, Colin E.; dateIdentified: 2015; **Event:** verbatimEventDate: November 24, 2014; habitat: Sandstone rubble on sandy plain; **Record Level:** institutionID: CMN; collectionID: CANL 127972; collectionCode: CANL; basisOfRecord: Preserved Specimen

##### Notes

This soil crust lichen is common in the Great Basin desert shrub lands and on the Colorado Plateau (St. Clair et al. 1991). The lower cortex is comprised of a distinct layer of globular cells, 20-70 μm high, with the lowermost cells brown to black. Breuss (2002) describes the lower cortex with angular cells in distinct vertical columns. McCune and Rosentreter (2007) provide a photo that shows +/- globular cells in a non-aligned pattern. Brodo et al. (2016) describe the cells of the lower cortex as spherical and sometimes in vertical columns, which corresponds well with our specimen (Fig. 20b). Other key characteristics of *Placidium lachneum* include the presence of marginal pycnidia and hyphal wefts that help to attach the lichen to the soil, as shown in Fig. 22c.

**Supplemental File**: CANL 127972 (Suppl. material 21).

### Vascular Plant Collections

#### Amaranthaceae

#### Atriplex confertifolia

(Torr. & Frém.) S. Watson

##### Materials

- **Type status:** Other material. **Occurrence:** recordNumber: 313; recordedBy: Sokoloff, Paul C.; preparations: Silica gel collection; **Taxon:** scientificName: *Atriplex confertifolia* (Torr. & Frém.) S. Watson; kingdom: Plantae; phylum: Angiosperms; class: Eudicots; order: Caryophyllales; family: Amaranthaceae; genus: Atriplex; specificEpithet: confertifolia; taxonRank: Species; scientificNameAuthorship: (Torr. & Frém.) S. Watson; **Location:** continent: North America; country: United States of America; countryCode: USA; stateProvince: Utah; county: Wayne County; municipality: Hanksville; locality: Mars Desert Research Station; verbatimLocality: Sandstone plateau immediately southwest of Mars Desert Research Station, alongside ATV trail; verbatimElevation: 1412 m; verbatimLatitude: 38°24'22.4"N; verbatimLongitude: 110°47'40.3"W; coordinateUncertaintyInMeters: 50; **Identification:** identifiedBy: Sokoloff, Paul C.; dateIdentified: 2015; **Event:** verbatimEventDate: November 24, 2014; habitat: Dry conglomerate sandstone; **Record Level:** institutionID: CMN; collectionID: CAN 607477; collectionCode: CAN, UTC; basisOfRecord: Preserved Specimen

##### Notes

Common on dry saline soils (Welsh 2003), *Atriplex confertifolia* was abundant along a seasonally wet streambed north of MDRS. This widely distributed species readily hybridizes with other congeneric taxa, including *Atriplex corrugata* and *A. gardneri* (Stutz 1978), both of which are found in the study area. This species is known from the nearby San Rafael Swell (Harris 1983​).

**Supplemental File**: CAN 607477 (Suppl. material 22).

#### Atriplex corrugata

S. Watson

##### Materials

- **Type status:** Other material. **Occurrence:** recordNumber: 273; recordedBy: Sokoloff, Paul C.; preparations: Silica gel collection; **Taxon:** scientificName: *Atriplex corrugata* S. Watson; kingdom: Plantae; phylum: Angiosperms; class: Eudicots; order: Caryophyllales; family: Amaranthaceae; genus: Atriplex; specificEpithet: corrugata; taxonRank: Species; scientificNameAuthorship: S. Watson; **Location:** continent: North America; country: United States of America; countryCode: USA; stateProvince: Utah; county: Wayne County; municipality: Hanksville; locality: Mars Desert Research Station; verbatimLocality: Seasonally wet stream crossing on Cow Dung Road, 1.6 km northeast of Mars Desert Research Station; verbatimElevation: 1371 m; verbatimLatitude: 38°25'55.39"N; verbatimLongitude: 110°47'30.2"W; coordinateUncertaintyInMeters: 50; **Identification:** identifiedBy: Sokoloff, Paul C.; dateIdentified: 2015; **Event:** verbatimEventDate: November 22, 2014; habitat: Desert slopes; **Record Level:** institutionID: CMN; collectionID: CAN 607503; collectionCode: CAN, UTC; basisOfRecord: Preserved Specimen

##### Notes

Common on the Mancos shale formation of eastern Utah and western Colorado (Welsh 2003), *Atriplex corrugata* was abundant at a seasonally wet streambed north of MDRS. This species was previously reported in the nearby San Rafael Swell (Harris 1983).

**Supplemental File**: CAN 607503 (Suppl. material 23).

#### Atriplex gardnerivar.cuneata

(A. Nelson) S.L. Welsh

##### Materials

- **Type status:** Other material. **Occurrence:** recordNumber: 256; recordedBy: Sokoloff, Paul C.; preparations: Silica gel collection; **Taxon:** scientificName: Atriplex gardneri (Moq.) D. Dietr. var. cuneata (A. Nelson) S.L. Welsh; kingdom: Plantae; phylum: Angiosperms; class: Eudicots; order: Caryophyllales; family: Amaranthaceae; genus: Atriplex; specificEpithet: gardneri; infraspecificEpithet: cuneata; taxonRank: Variety; scientificNameAuthorship: (A. Nelson) S.L. Welsh; **Location:** continent: North America; country: United States of America; countryCode: USA; stateProvince: Utah; county: Wayne County; municipality: Hanksville; locality: Mars Desert Research Station; verbatimLocality: Vicinity of the Mars Desert Research Station, Hanksville, Utah, 500 m radius of "hab"; verbatimElevation: 1371 m; verbatimLatitude: 38°24'23.2"N; verbatimLongitude: 110°47'31.1"W; coordinateUncertaintyInMeters: 50; **Identification:** identifiedBy: Sokoloff, Paul C.; dateIdentified: 2015; **Event:** verbatimEventDate: November 17, 2014; habitat: Sandy washes and outcrops surrounding MDRS; **Record Level:** institutionID: CMN; collectionID: CAN 607507; collectionCode: CAN, UTC; basisOfRecord: Preserved Specimen
- **Type status:** Other material. **Occurrence:** recordNumber: 259; recordedBy: Sokoloff, Paul C.; preparations: Silica gel collection; **Taxon:** scientificName: Atriplex gardneri (Moq.) D. Dietr. var. cuneata (A. Nelson) S.L. Welsh; kingdom: Plantae; phylum: Angiosperms; class: Eudicots; order: Caryophyllales; family: Amaranthaceae; genus: Atriplex; specificEpithet: gardneri; infraspecificEpithet: cuneata; taxonRank: Variety; scientificNameAuthorship: (A. Nelson) S.L. Welsh; **Location:** continent: North America; country: United States of America; countryCode: USA; stateProvince: Utah; county: Wayne County; municipality: Hanksville; locality: Mars Desert Research Station; verbatimLocality: Vicinity of the Mars Desert Research Station, Hanksville, Utah, 500 m radius of "hab"; verbatimElevation: 1371 m; verbatimLatitude: 38°24'23.2"N; verbatimLongitude: 110°47'31.1"W; coordinateUncertaintyInMeters: 50; **Identification:** identifiedBy: Sokoloff, Paul C.; dateIdentified: 2015; **Event:** verbatimEventDate: November 17, 2014; habitat: Sandy washes and outcrops surrounding MDRS; **Record Level:** institutionID: CMN; collectionID: CAN 607505; collectionCode: CAN, UTC; basisOfRecord: Preserved Specimen
- **Type status:** Other material. **Occurrence:** recordNumber: 266; recordedBy: Sokoloff, Paul C.; preparations: Silica gel collection; **Taxon:** scientificName: Atriplex gardneri (Moq.) D. Dietr. var. cuneata (A. Nelson) S.L. Welsh; kingdom: Plantae; phylum: Angiosperms; class: Eudicots; order: Caryophyllales; family: Amaranthaceae; genus: Atriplex; specificEpithet: gardneri; infraspecificEpithet: cuneata; taxonRank: Variety; scientificNameAuthorship: (A. Nelson) S.L. Welsh; **Location:** continent: North America; country: United States of America; countryCode: USA; stateProvince: Utah; county: Wayne County; municipality: Hanksville; locality: Mars Desert Research Station; verbatimLocality: Dry streambed approx 500 m northeast of Mars Desert Research Station "hab"; verbatimElevation: 1371 m; verbatimLatitude: 38°24'27.7"N; verbatimLongitude: 110°47'20"W; coordinateUncertaintyInMeters: 50; **Identification:** identifiedBy: Sokoloff, Paul C.; dateIdentified: 2015; **Event:** verbatimEventDate: November 20, 2014; habitat: Silty dry streambed; **Record Level:** institutionID: CMN; collectionID: CAN 607506; collectionCode: CAN, UTC; basisOfRecord: Preserved Specimen

##### Notes

This was one of the most commonly encountered species in the vicinity of MDRS (Fig. 23), and was seen on sandy desert flats throughout the study area. This species displays a great deal of phenotypic plasticity throughout its range, and hybridizes readily wth other sympatric species of *Atriplex*, complicating taxonomic delimitation (Stutz 1978). Previously recorded in the nearby San Rafael Swell as *Atriplex cuneata* A. Nelson (Harris 1983), here we follow Welsh (2003) in treating this taxon at the subspecies level.

**Supplemental Files**: CAN 607507 (Suppl. material 24), CAN 607505 (Suppl. material 25), CAN 607506 (Suppl. material 26).

#### Halogeton glomeratus

(M. Bieb.) C.A. Mey.

##### Materials

- **Type status:** Other material. **Occurrence:** recordNumber: 254; recordedBy: Sokoloff, Paul C.; **Taxon:** scientificName: *Halogeton glomeratus* (M. Bieb.) C.A. Mey.; kingdom: Plantae; phylum: Angiosperms; class: Eudicots; order: Caryophyllales; family: Amaranthaceae; genus: Halogeton; specificEpithet: glomeratus; taxonRank: Species; scientificNameAuthorship: (M. Bieb.) C.A. Mey.; **Location:** continent: North America; country: United States of America; countryCode: USA; stateProvince: Utah; county: Wayne County; municipality: Hanksville; locality: Mars Desert Research Station; verbatimLocality: Vicinity of the Mars Desert Research Station, Hanksville, Utah, 500 m radius of "hab"; verbatimElevation: 1371 m; verbatimLatitude: 38°24'23.2"N; verbatimLongitude: 110°47'31.1"W; coordinateUncertaintyInMeters: 50; **Identification:** identifiedBy: Sokoloff, Paul C.; dateIdentified: 2015; **Event:** verbatimEventDate: November 17, 2014; habitat: Sandy washes and outcrops surrounding MDRS; **Record Level:** institutionID: CMN; collectionID: CAN 607484; collectionCode: CAN, UTC; basisOfRecord: Preserved Specimen
- **Type status:** Other material. **Occurrence:** recordNumber: 267; recordedBy: Sokoloff, Paul C.; **Taxon:** scientificName: *Halogeton glomeratus* (M. Bieb.) C.A. Mey.; kingdom: Plantae; phylum: Angiosperms; class: Eudicots; order: Caryophyllales; family: Amaranthaceae; genus: Halogeton; specificEpithet: glomeratus; taxonRank: Species; scientificNameAuthorship: (M. Bieb.) C.A. Mey.; **Location:** continent: North America; country: United States of America; countryCode: USA; stateProvince: Utah; county: Wayne County; municipality: Hanksville; locality: Mars Desert Research Station; verbatimLocality: Dry streambed approx 500 m northeast of Mars Desert Research Station "hab"; verbatimElevation: 1371 m; verbatimLatitude: 38°24'27.7"N; verbatimLongitude: 110°47'20"W; coordinateUncertaintyInMeters: 50; **Identification:** identifiedBy: Sokoloff, Paul C.; dateIdentified: 2015; **Event:** verbatimEventDate: November 20, 2014; habitat: Rocky sandstone desert; **Record Level:** institutionID: CMN; collectionID: CAN 607485; collectionCode: CAN, UTC; basisOfRecord: Preserved Specimen

##### Notes

This introduced species is highly invasive in the western United States, flourishing in disturbed habitats and alkaline soils (Holmgren 2003). It was commonly encountered along Cow Dung Road, and has flourished in the disturbed areas immediately surrounding MDRS (Fig. 24). This species was previously recorded for the nearby San Rafael Swell (Harris 1983).

**Supplemental Files**: CAN 607484 (Suppl. material 27), CAN 607485 (Suppl. material 28).

#### Kali tragus

(L.) Scop.

##### Notes

This species was observed and photographed on a sandstone outcrop approximately 1 km northeast of MDRS on September 20, 2015 (Fig. 25), however this plant was not collected due to time and equipment constraints. Common on sandy plateaus and *Guterriezia*-*Bromus* dominated scrub near MDRS, this species is a noxious weed found throughout the southwestern United States (Welsh et al. 1993). This species was previously reported for the nearby San Rafael Swell (Harris 1983) as *Salsola iberica* Sennen & Pau, which was later synonymized under *Salsola tragus* L. (Mosyakin 2003). Further phylogenetic work has transferred this species to the genus *Kali* (Akhani et al. 2007), now placed in the expanded Amaranthaceae (APG III 2009)

#### Asteraceae

#### Artemisia filifolia

Torr.

##### Materials

- **Type status:** Other material. **Occurrence:** recordNumber: 282; recordedBy: Sokoloff, Paul C.; preparations: Silica gel collection; **Taxon:** scientificName: *Artemisia filifolia* Torr.; kingdom: Plantae; phylum: Angiosperms; class: Eudicots; order: Asterales; family: Asteraceae; genus: Artemisia; specificEpithet: filifolia; taxonRank: Species; scientificNameAuthorship: Torr.; **Location:** continent: North America; country: United States of America; countryCode: USA; stateProvince: Utah; county: Wayne County; municipality: Hanksville; locality: Mars Desert Research Station; verbatimLocality: "Comm check" hill, 1.7 km northeast of Mars Desert Research Station, just west of Cow Dung Road; verbatimElevation: 1371 m; verbatimLatitude: 38°25'3.15"N; verbatimLongitude: 110°46'54.59"W; coordinateUncertaintyInMeters: 50; **Identification:** identifiedBy: Sokoloff, Paul C.; dateIdentified: 2015; **Event:** verbatimEventDate: November 22, 2014; habitat: Conglomerate sandstone hilltop dominated by *Artemisia* and *Ephedra*; **Record Level:** institutionID: CMN; collectionID: CAN 607478; collectionCode: CAN, UTC; basisOfRecord: Preserved Specimen

##### Notes

**Figure 26.**
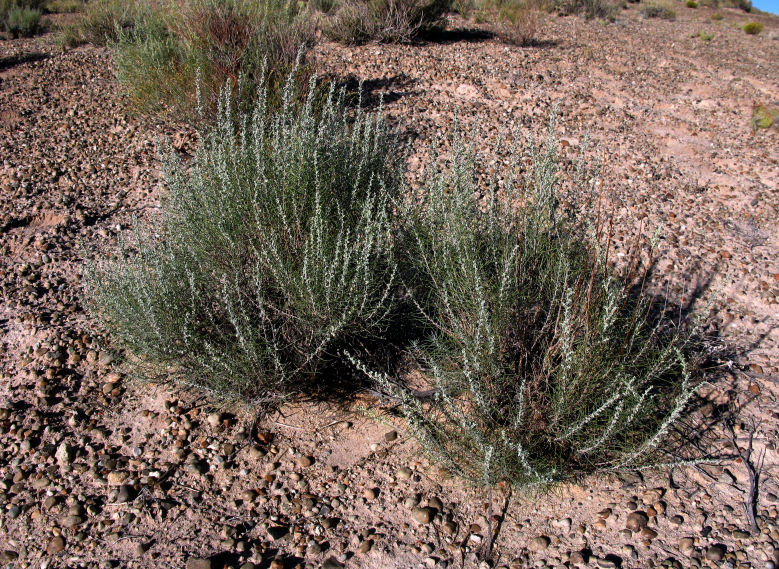
*Artemisia filifolia*. Habit. Vicinity of Mars Desert Research Station, Utah, September 19, 2015. Photo by P.C. Sokoloff.

Common on the plateau just west of the MDRS (Fig. 26) this species is abundant on sandy substrates of the region (Shultz 2006), and was previously reported for the nearby San Rafael Swell (Harris 1983).

**Supplemental File**: CAN 607478 (Suppl. material 29).

#### Chaenactis douglasiivar.douglasii

(Hook.) Hook. & Arn.

##### Materials

- **Type status:** Other material. **Occurrence:** recordNumber: 291; recordedBy: Sokoloff, Paul C.; preparations: Silica gel collection; **Taxon:** scientificName: Chaenactis douglasii (Hook.) Hook. & Arn. var. douglasii; kingdom: Plantae; phylum: Angiosperms; class: Eudicots; order: Asterales; family: Asteraceae; genus: Chaenactis; specificEpithet: douglasii; infraspecificEpithet: douglasii; taxonRank: Variety; scientificNameAuthorship: (Hook.) Hook. & Arn.; **Location:** continent: North America; country: United States of America; countryCode: USA; stateProvince: Utah; county: Wayne County; municipality: Hanksville; locality: Mars Desert Research Station; verbatimLocality: Approx. 200 m past fork on Cow Dung Road, down eastern fork, 1.9 km northeast of Mars Desert Research Station; verbatimElevation: 1381 m; verbatimLatitude: 38°25'3.2"N; verbatimLongitude: 110°46'29.7"W; coordinateUncertaintyInMeters: 50; **Identification:** identifiedBy: Sokoloff, Paul C.; dateIdentified: 2015; **Event:** verbatimEventDate: November 23, 2014; habitat: *Artemisia*-dominated desert scrub; **Record Level:** institutionID: CMN; collectionID: CAN 607472; collectionCode: CAN; basisOfRecord: Preserved Specimen

##### Notes

Found in desert shrub communities alongside the ATV trails north of MDRS, this widespread species was not recorded previously for the nearby San Rafael Swell (Harris 1983), though it is known to occur in nearby Capitol Reef National Park (Coles et al. 2009), and in the Four Corners region further southeast (Heil and O'Kane 2003). Though Welsh et al. (1993) did not recognize varieties, our specimen would be considered C. douglasii var. douglasii following Morefield (2006).

**Supplemental File**: CAN 607472 (Suppl. material 30).

#### Dieteria canescensvar.canescens

(Pursh) Nutt.

##### Materials

- **Type status:** Other material. **Occurrence:** recordNumber: 366; recordedBy: Sokoloff, Paul C.; **Taxon:** scientificName: Dieteria canescens (Pursh) Nutt. var. canescens; kingdom: Plantae; phylum: Angiosperms; class: Eudicots; order: Asterales; family: Asteraceae; genus: Dieteria; specificEpithet: canescens; infraspecificEpithet: canescens; taxonRank: Variety; scientificNameAuthorship: (Pursh) Nutt.; **Location:** continent: North America; country: United States of America; countryCode: USA; stateProvince: Utah; county: Wayne County; municipality: Hanksville; locality: Mars Desert Research Station; verbatimLocality: Parking lot of Burpee Dinosaur Quarry, end of Cow Dung Road; verbatimElevation: 1377 m; verbatimLatitude: 38°27'8.24"N; verbatimLongitude: 110°47'27.384"W; coordinateUncertaintyInMeters: 50; **Identification:** identifiedBy: Sokoloff, Paul C.; dateIdentified: 2015; **Event:** verbatimEventDate: September 19, 2015; habitat: Dry sandstone bluffs; **Record Level:** institutionID: CMN; collectionID: CAN 607522; basisOfRecord: Preserved Specimen

##### Notes

This species was recorded for the nearby San Rafael Swell as *Machaeranthera canescens* (Pursh) Gray (Harris 1983); the currently accepted name for this taxon is *Dieteria canecens* (Morgan 2006) based on molecular analysis and reclassification of the polyphyletic genus *Machaeranthera* (Morgan et al. 2009​).

**Supplemental File**: CAN 607552 (Suppl. material 31).

#### Ericameria nauseosa

(Pall. ex Pursh) G.L. Nesom & G.I. Baird

##### Materials

- **Type status:** Other material. **Occurrence:** recordNumber: 284; recordedBy: Sokoloff, Paul C.; preparations: Silica gel collection; **Taxon:** scientificName: *Ericameria nauseosa* (Pall. ex Pursh) G.L. Nesom & G.I. Baird; kingdom: Plantae; phylum: Angiosperms; class: Eudicots; order: Asterales; family: Asteraceae; genus: Ericameria; specificEpithet: nauseosa; taxonRank: Species; scientificNameAuthorship: (Pall. ex Pursh) G.L. Nesom & G.I. Baird; **Location:** continent: North America; country: United States of America; countryCode: USA; stateProvince: Utah; county: Wayne County; municipality: Hanksville; locality: Mars Desert Research Station; verbatimLocality: Kent's Reservoir, 1.14 km north of Mars Desert Research Station, just west of Cow Dung Road; verbatimElevation: 1371 m; verbatimLatitude: 38°25'28.4"N; verbatimLongitude: 110°47'17.29"W; coordinateUncertaintyInMeters: 50; **Identification:** identifiedBy: Sokoloff, Paul C.; dateIdentified: 2015; identificationRemarks: Unable to determine to variety as no stigmatic appendages present; **Event:** verbatimEventDate: November 22, 2014; habitat: Moist desert flats; **Record Level:** institutionID: CMN; collectionID: CAN 607481; collectionCode: CAN, UTC; basisOfRecord: Preserved Specimen

##### Notes

*Ericameria nauseosa* was abundant along Cow Dung Road due north of MDRS, in the lowlands between rocky outcrops between MDRS and the Burpee Dinosaur Quarry (Fig. 27). Welsh et al. (1993) treats this species as *Chrysothamnus nauseous* (Pallas ex Pursh) Britton, with 14 varieties in Utah. Four of these varieties (C. nauseous var. consimilis (Greene) Hall, C. nauseous var. gnaphaloides (Greene) Hall, C. nauseous var. junceus (Greene) Hall, and C. nauseous var. leiospermus (Gray) Hall) were previously reported for the nearby San Rafael Swell (Harris 1983). Urbatshc et al. (2006), whose treatment we follow here, accept 21 varieties in North America; however we were unable to identify our specimen to variety as the diagnostic phyllaries were missing.

**Supplemental File**: CAN 607481 (Suppl. material 32).

#### Gaillardia spathulata

A. Gray

##### Materials

- **Type status:** Other material. **Occurrence:** recordNumber: 367; recordedBy: Sokoloff, Paul C.; **Taxon:** scientificName: *Gaillardia spathulata* A. Gray; kingdom: Plantae; phylum: Angiosperms; class: Eudicots; order: Asterales; family: Asteraceae; genus: Gaillardia; specificEpithet: spathulata; scientificNameAuthorship: A. Gray; **Location:** continent: North America; country: United States of America; countryCode: USA; stateProvince: Utah; county: Wayne County; municipality: Hanksville; locality: Mars Desert Research Station; verbatimLocality: Sandstone plateau immediately southwest of Mars Desert Research Station, alongside ATV trail; verbatimElevation: 1412 m; verbatimLatitude: 38°24'22.4"N; verbatimLongitude: 110°47'40.3"W; coordinateUncertaintyInMeters: 50; **Identification:** identifiedBy: Sokoloff, Paul C.; dateIdentified: 2015; **Event:** verbatimEventDate: September 19, 2015; habitat: Dry sandy soil; **Record Level:** institutionID: CMN; collectionID: CAN 607524; collectionCode: CAN; basisOfRecord: PreservedSpecimen

##### Notes

A common plant of sandy desert soils (Strother 2006a), this species was abundant on the plateau immediately southwest of MDRS. This species was previously recorded for the nearby San Rafael Swell (Harris 1983).

**Supplemental File**: CAN 607524 (Suppl. material 33).

#### Gutierrezia sarothrae

(Pursh) Britton & Rusby

##### Materials

- **Type status:** Other material. **Occurrence:** recordNumber: 253; recordedBy: Sokoloff, Paul C.; preparations: Silica gel collection; **Taxon:** scientificName: *Gutierrezia sarothrae* (Pursh) Britton & Rusby; kingdom: Plantae; phylum: Angiosperms; class: Eudicots; order: Asterales; family: Asteraceae; genus: Gutierrezia; specificEpithet: sarothrae; taxonRank: Species; scientificNameAuthorship: (Pursh) Britton & Rusby; **Location:** continent: North America; country: United States of America; countryCode: USA; stateProvince: Utah; county: Wayne County; municipality: Hanksville; locality: Mars Desert Research Station; verbatimLocality: Vicinity of the Mars Desert Research Station, Hanksville, Utah, 500 m radius of "hab"; verbatimElevation: 1371 m; verbatimLatitude: 38°24'23.2"N; verbatimLongitude: 110°47'31.1"W; coordinateUncertaintyInMeters: 50; **Identification:** identifiedBy: Sokoloff, Paul C.; dateIdentified: 2015; **Event:** verbatimEventDate: November 17, 2014; habitat: Sandy washes and outcrops surrounding MDRS; **Record Level:** institutionID: CMN; collectionID: CAN 607462; collectionCode: CAN, UTC; basisOfRecord: Preserved Specimen
- **Type status:** Other material. **Occurrence:** recordNumber: 257; recordedBy: Sokoloff, Paul C.; preparations: Silica gel collection; **Taxon:** scientificName: *Gutierrezia sarothrae* (Pursh) Britton & Rusby; kingdom: Plantae; phylum: Angiosperms; class: Eudicots; order: Asterales; family: Asteraceae; genus: Gutierrezia; specificEpithet: sarothrae; taxonRank: Species; scientificNameAuthorship: (Pursh) Britton & Rusby; **Location:** continent: North America; country: United States of America; countryCode: USA; stateProvince: Utah; county: Wayne County; municipality: Hanksville; locality: Mars Desert Research Station; verbatimLocality: Vicinity of the Mars Desert Research Station, Hanksville, Utah, 500 m radius of "hab"; verbatimElevation: 1371 m; verbatimLatitude: 38°24'23.2"N; verbatimLongitude: 110°47'31.1"W; coordinateUncertaintyInMeters: 50; **Identification:** identifiedBy: Sokoloff, Paul C.; dateIdentified: 2015; **Event:** verbatimEventDate: November 17, 2014; habitat: Sandy washes and outcrops surrounding MDRS; **Record Level:** institutionID: CMN; collectionID: CAN 607469; collectionCode: CAN, UTC; basisOfRecord: Preserved Specimen
- **Type status:** Other material. **Occurrence:** recordNumber: 311; recordedBy: Sokoloff, Paul C.; preparations: Silica gel collection; **Taxon:** scientificName: *Gutierrezia sarothrae* (Pursh) Britton & Rusby; kingdom: Plantae; phylum: Angiosperms; class: Eudicots; order: Asterales; family: Asteraceae; genus: Gutierrezia; specificEpithet: sarothrae; taxonRank: Species; scientificNameAuthorship: (Pursh) Britton & Rusby; **Location:** continent: North America; country: United States of America; countryCode: USA; stateProvince: Utah; county: Wayne County; municipality: Hanksville; locality: Mars Desert Research Station; verbatimLocality: Sandstone plateau immediately southwest of Mars Desert Research Station, alongside ATV trail; verbatimElevation: 1412 m; verbatimLatitude: 38°24'22.4"N; verbatimLongitude: 110°47'40.3"W; coordinateUncertaintyInMeters: 50; **Identification:** identifiedBy: Sokoloff, Paul C.; dateIdentified: 2015; **Event:** verbatimEventDate: November 24, 2014; habitat: Dry conglomerate sandstone; **Record Level:** institutionID: CMN; collectionID: CAN 607463; collectionCode: CAN; basisOfRecord: Preserved Specimen

##### Notes

**Figure 28.**
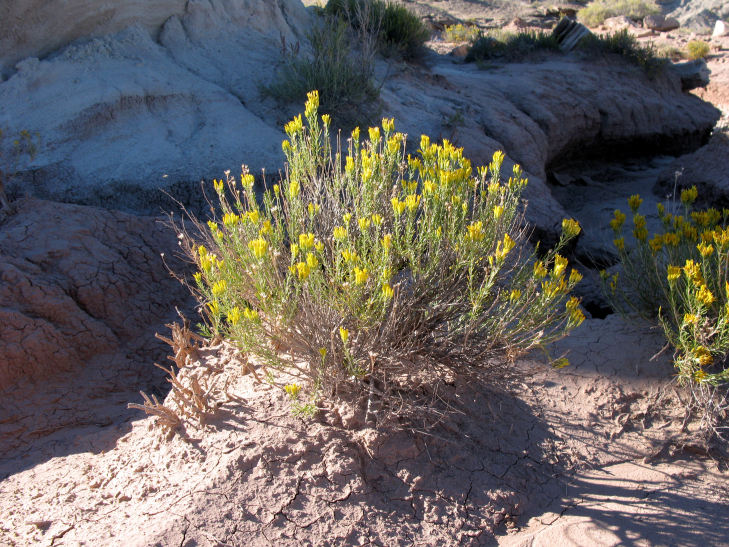
*Gutierrezia sarothrae*. Habit. Vicinity of Mars Desert Research Station, Utah, September 19, 2015. Photo by P.C. Sokoloff.

Abundant on the rocky outcrops, desert shrub communities (Fig. 28), and plateaus surrounding MDRS, this species is well adapted to disturbance in Utah's desert rangelands (Welsh et al. 1993). *Gutierrezia sarothrae* was previously reported from the nearby San Rafael Swell as *Xanthocephalum sarothrae* (Pursh) Shinners (Harris 1983). *Gutierrezia* is now segregated into a distinct genus (Nesom 2006).

**Supplemental Files**: CAN 607462 (Suppl. material 34), CAN 607469 (Suppl. material 35), CAN 607463 (Suppl. material 36).

#### Hymenoxys cooperi

(A. Gray) Cockerell

##### Materials

- **Type status:** Other material. **Occurrence:** recordNumber: 312; recordedBy: Sokoloff, Paul C.; preparations: Silica gel collection; **Taxon:** scientificName: *Hymenoxys cooperi* (A. Gray) Cockerell; kingdom: Plantae; phylum: Angiosperms; class: Eudicots; order: Asterales; family: Asteraceae; genus: Hymenoxys; specificEpithet: cooperi; taxonRank: Species; scientificNameAuthorship: (A. Gray) Cockerell; **Location:** continent: North America; country: United States of America; countryCode: USA; stateProvince: Utah; county: Wayne County; municipality: Hanksville; locality: Mars Desert Research Station; verbatimLocality: Sandstone plateau immediately southwest of Mars Desert Research Station, alongside ATV trail; verbatimElevation: 1412 m; verbatimLatitude: 38°24'22.4"N; verbatimLongitude: 110°47'40.3"W; coordinateUncertaintyInMeters: 50; **Identification:** identifiedBy: Sokoloff, Paul C.; dateIdentified: 2015; **Event:** verbatimEventDate: November 24, 2014; habitat: Dry conglomerate sandstone; **Record Level:** institutionID: CMN; collectionID: CAN 607483; collectionCode: CAN; basisOfRecord: Preserved Specimen

##### Notes

This species, collected on the plateau west of MDRS, was not recorded in the previous floristic survey of the nearby San Rafael Swell Harris (1983) but there is a record of this species occurring in the Glen Canyon Recreational Area to the south (Hill and Ayers 2009). A shorter form with fewer heads is sometimes recognized as H. cooperi var. canescens (D.C. Eaton) K. F. Parker; however Welsh et al. (1993) did not recognize varieties in Utah, and Bierner (2006) did not recognize varieties in this species whatsoever.

**Supplemental File**: CAN 607483 (Suppl. material 37).

#### Scabrethia scabra

(Hook.) W.A. Weber

##### Materials

- **Type status:** Other material. **Occurrence:** recordNumber: 280; recordedBy: Sokoloff, Paul C.; **Taxon:** scientificName: *Scabrethia scabra* (Hook.) W.A. Weber; kingdom: Plantae; phylum: Angiosperms; class: Eudicots; order: Asterales; family: Asteraceae; genus: Scabrethia; specificEpithet: scabra; taxonRank: Species; scientificNameAuthorship: (Hook.) W.A. Weber; **Location:** continent: North America; country: United States of America; countryCode: USA; stateProvince: Utah; county: Wayne County; municipality: Hanksville; locality: Mars Desert Research Station; verbatimLocality: "Comm check" hill, 1.7 km northeast of Mars Desert Research Station, just west of Cow Dung Road; verbatimElevation: 1371 m; verbatimLatitude: 38°25'3.15"N; verbatimLongitude: 110°46'54.59"W; coordinateUncertaintyInMeters: 50; **Identification:** identifiedBy: Sokoloff, Paul C.; dateIdentified: 2015; identificationRemarks: Unable to determine to subspecies, as no phyllaries present; **Event:** verbatimEventDate: November 22, 2014; habitat: Conglomerate sandstone hilltop dominated by *Artemisia* and *Ephedra*; **Record Level:** institutionID: CMN; collectionID: CAN 607479; collectionCode: CAN, UTC; basisOfRecord: Preserved Specimen

##### Notes

 Previously reported for the nearby San Rafael Swell as *Wyethia scabra* Hook. (Harris 1983), we follow Weber (2006), who had previously placed this species in *Scabrethia* (Weber 1998). We were unable to identify this specimen to subspecies as the diagnostic phyllaries were missing (Fig. 29)​.

**Supplemental File**: CAN 607479 (Suppl. material 38).

#### Thelesperma subnudum

A. Gray

##### Materials

- **Type status:** Other material. **Occurrence:** recordNumber: 281; recordedBy: Sokoloff, Paul C.; preparations: Silica gel collection; **Taxon:** scientificName: *Thelesperma subnudum* A. Gray; kingdom: Plantae; phylum: Angiosperms; class: Eudicots; order: Asterales; family: Asteraceae; genus: Thelesperma; specificEpithet: subnudum; taxonRank: Species; scientificNameAuthorship: A. Gray; **Location:** continent: North America; country: United States of America; countryCode: USA; stateProvince: Utah; county: Wayne County; municipality: Hanksville; locality: Mars Desert Research Station; verbatimLocality: "Comm check" hill, 1.7 km northeast of Mars Desert Research Station, just west of Cow Dung Road; verbatimElevation: 1371 m; verbatimLatitude: 38°25'3.15"N; verbatimLongitude: 110°46'54.59"W; coordinateUncertaintyInMeters: 50; **Identification:** identifiedBy: Sokoloff, Paul C.; dateIdentified: 2015; **Event:** verbatimEventDate: November 22, 2014; habitat: Conglomerate sandstone hilltop dominated by *Artemisia* and *Ephedra*; **Record Level:** institutionID: CMN; collectionID: CAN 607470; collectionCode: CAN, UTC; basisOfRecord: Preserved Specimen

##### Notes

**Figure 30.**
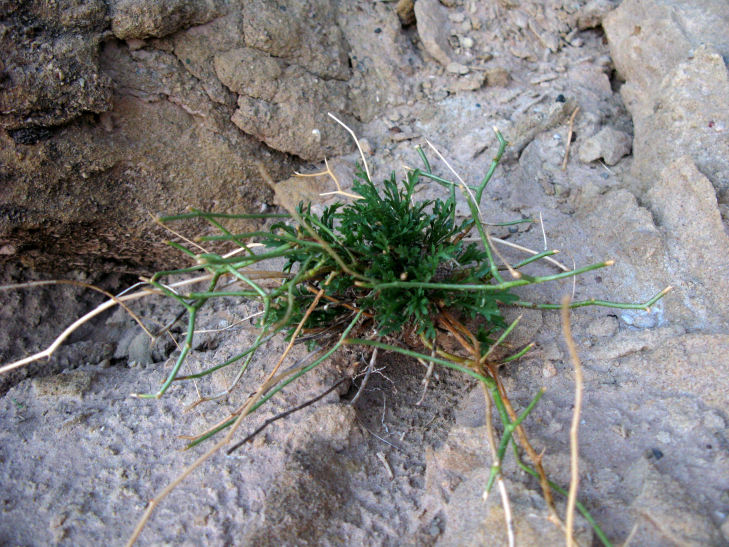
*Thelesperma subnudum*. Basal leaves. Vicinity of Mars Desert Research Station, Utah, September 20, 2015. Photo by P.C. Sokoloff.

Common on sandstone bluffs in the region (Fig. 30) this species was previously reported for the nearby San Rafael Swell (Harris 1983​). Welsh et al. (1993) described several varieties of this species in Utah, all of which have been subsumed under the broadly circumscribed *T. subnudum* of Strother (2006b).

**Supplemental File**: CAN 607470 (Suppl. material 39).

#### Boraginaceae

#### Cryptantha humilis

(Greene) Payson

##### Materials

- **Type status:** Other material. **Occurrence:** recordNumber: 314; recordedBy: Sokoloff, Paul C.; preparations: Silica gel collection; **Taxon:** scientificName: *Cryptantha humilis* (Greene) Payson; kingdom: Plantae; phylum: Angiosperms; class: Eudicots; family: Boraginaceae; genus: Cryptantha; specificEpithet: humilis; taxonRank: Species; scientificNameAuthorship: (Greene) Payson; **Location:** continent: North America; country: United States of America; countryCode: USA; stateProvince: Utah; county: Wayne County; municipality: Hanksville; locality: Mars Desert Research Station; verbatimLocality: Sandstone plateau immediately southwest of Mars Desert Research Station, alongside ATV trail; verbatimElevation: 1412 m; verbatimLatitude: 38°24'22.4"N; verbatimLongitude: 110°47'40.3"W; coordinateUncertaintyInMeters: 50; **Identification:** identifiedBy: Sokoloff, Paul C.; dateIdentified: 2015; **Event:** verbatimEventDate: November 24, 2014; habitat: Dry conglomerate sandstone; **Record Level:** institutionID: CMN; collectionID: CAN 607504; collectionCode: CAN; basisOfRecord: Preserved Specimen

##### Notes

This species was previously recorded for the nearby San Rafael Swell as Cryptantha humilis var. nana (Eastw.) L.C. Higgins (Harris 1983). Welsh et al. (1993) suggests that while var. *humilisnana* might be applied to plants from the Uinta Basin (north of the study area), however they assert that variation of intraspecific diagnostic characters from across the range of *C. humilis* does not support the treatment of varieties.

**Supplemental File**: CAN 607504 (Suppl. material 40).

#### Brassicaceae

#### Lepidium montanum

Nutt.

##### Materials

- **Type status:** Other material. **Occurrence:** recordNumber: 261; recordedBy: Sokoloff, Paul C.; preparations: Silica gel collection; **Taxon:** scientificName: *Lepidium montanum* Nutt.; kingdom: Plantae; phylum: Angiosperms; class: Eudicots; order: Brassicales; family: Brassicaceae; genus: Lepidium; specificEpithet: montanum; taxonRank: Species; scientificNameAuthorship: Nutt.; **Location:** continent: North America; country: United States of America; countryCode: USA; stateProvince: Utah; county: Wayne County; municipality: Hanksville; locality: Mars Desert Research Station; verbatimLocality: Vicinity of the Mars Desert Research Station, Hanksville, Utah, 500 m radius of "hab"; verbatimElevation: 1371 m; verbatimLatitude: 38°24'23.2"N; verbatimLongitude: 110°47'31.1"W; coordinateUncertaintyInMeters: 50; **Identification:** identifiedBy: Sokoloff, Paul C.; dateIdentified: 2015; **Event:** verbatimEventDate: November 17, 2014; habitat: Sandy washes and outcrops surrounding MDRS; **Record Level:** institutionID: CMN; collectionID: CAN 607467; collectionCode: CAN, UTC; basisOfRecord: Preserved Specimen
- **Type status:** Other material. **Occurrence:** recordNumber: 263; recordedBy: Sokoloff, Paul C.; preparations: Silica gel collection; **Taxon:** scientificName: *Lepidium montanum* Nutt.; kingdom: Plantae; phylum: Angiosperms; class: Eudicots; order: Brassicales; family: Brassicaceae; genus: Lepidium; specificEpithet: montanum; taxonRank: Species; scientificNameAuthorship: Nutt.; **Location:** continent: North America; country: United States of America; countryCode: USA; stateProvince: Utah; county: Wayne County; municipality: Hanksville; locality: Mars Desert Research Station; verbatimLocality: Dry streambed approx 500 m northeast of Mars Desert Research Station "hab"; verbatimElevation: 1371 m; verbatimLatitude: 38°24'27.7"N; verbatimLongitude: 110°47'20"W; coordinateUncertaintyInMeters: 50; **Identification:** identifiedBy: Sokoloff, Paul C.; dateIdentified: 2015; **Event:** verbatimEventDate: November 20, 2014; habitat: Rocky sandstone desert; **Record Level:** institutionID: CMN; collectionID: CAN 607471; collectionCode: CAN, UTC; basisOfRecord: Preserved Specimen

##### Notes

This species was commonly encountered in the sandy washes immediately surrounding MDRS (Fig. 31). Widely variable across its range, Welsh et al. (1993) report nine named varieties in Utah, and Harris (1983) reports L. montanum var. jonesii (Rydb.) C.L. Hitch. from the nearby San Rafael Swell. Al-Shebaz and Gaskin (2010) subsume these varieties into *L. montanum* *sensu lato* pending a thorough study of infraspecific delimitation; we follow their treatment here.

**Supplemental Files**: CAN 607467 (Suppl. material 41), CAN 607471 (Suppl. material 42).

#### Cactaceae

#### Opuntia basilarisvar.basilaris

Engelm. & J.M. Bigelow

##### Materials

- **Type status:** Other material. **Occurrence:** recordNumber: 272; recordedBy: Sokoloff, Paul C.; preparations: Silica gel collection; **Taxon:** scientificName: Opuntia basilaris Engelm. & J.M. Bigelow var. basilaris; kingdom: Plantae; phylum: Angiosperms; class: Eudicots; order: Caryophyllales; family: Cactaceae; genus: Opuntia; specificEpithet: basilaris; infraspecificEpithet: basilaris; taxonRank: Variety; scientificNameAuthorship: Engelm. & J.M. Bigelow; **Location:** continent: North America; country: United States of America; countryCode: USA; stateProvince: Utah; county: Wayne County; municipality: Hanksville; locality: Mars Desert Research Station; verbatimLocality: Seasonally wet stream crossing on Cow Dung Road, 1.6 km northeast of Mars Desert Research Station; verbatimElevation: 1371 m; verbatimLatitude: 38°25'55.39"N; verbatimLongitude: 110°47'30.2"W; coordinateUncertaintyInMeters: 50; **Identification:** identifiedBy: Sokoloff, Paul C.; dateIdentified: 2015; **Event:** verbatimEventDate: November 22, 2014; habitat: Desert slopes; **Record Level:** institutionID: CMN; collectionID: CAN 607488; collectionCode: CAN, UTC; basisOfRecord: Preserved Specimen

##### Notes

**Figure 32.**
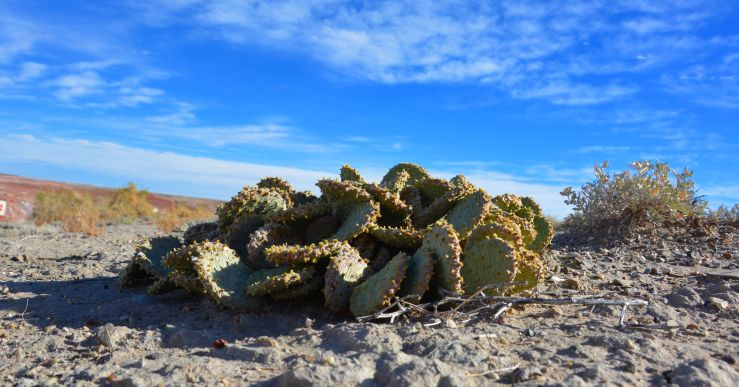
Opuntia basilaris var. basiliaris (*Sokoloff 272*). Habit. Photo by P.C. Sokoloff.

​ This species was common along the banks of a seasonally wet stream crossing due northeast of MDRS (Fig. 32). This species was previously recorded from the nearby San Rafael Swell as *Opuntia basilaris* (without infraspecific rank) (Harris 1983). Morphological variability and hybridization have hindered infraspecific delineation in this species, and many named varieties appear in the literature, including four in Welsh et al. (1993), and a different set of four in Pinkava (2003); here we follow the latter treatment.

**Supplemental File**: CAN 607488 (Suppl. material 43).

#### Opuntia polyacanthavar.polyacantha

Haw.

##### Materials

- **Type status:** Other material. **Occurrence:** recordNumber: 293; recordedBy: Sokoloff, Paul C.; preparations: Silica gel collection; **Taxon:** scientificName: *Opuntia polyacantha* Haw. var. polyacantha; kingdom: Plantae; phylum: Angiosperms; class: Eudicots; order: Caryophyllales; family: Cactaceae; genus: Opuntia; specificEpithet: polyacantha; infraspecificEpithet: polyacantha; taxonRank: Variety; scientificNameAuthorship: Haw.; **Location:** continent: North America; country: United States of America; countryCode: USA; stateProvince: Utah; county: Wayne County; municipality: Hanksville; locality: Mars Desert Research Station; verbatimLocality: Approx. 200 m past fork on Cow Dung Road, down eastern fork, 1.9 km northeast of Mars Desert Research Station; verbatimElevation: 1381 m; verbatimLatitude: 38°25'3.2"N; verbatimLongitude: 110°46'29.7"W; coordinateUncertaintyInMeters: 50; **Identification:** identifiedBy: Sokoloff, Paul C.; dateIdentified: 2015; **Event:** verbatimEventDate: November 23, 2014; habitat: *Artemisia*-dominated desert scrub; **Record Level:** institutionID: CMN; collectionID: CAN 607489; collectionCode: CAN, UTC; basisOfRecord: Preserved Specimen

##### Notes

**Figure 33.**
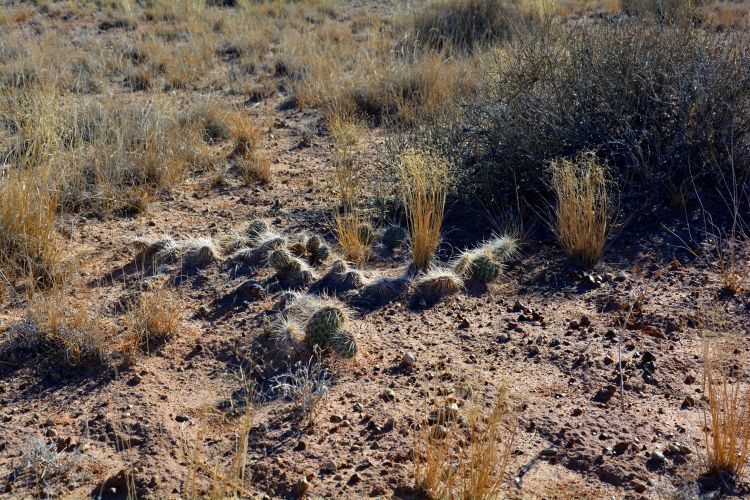
Opuntia polyacantha var. polyacantha (*Sokoloff 293*). Habitat. Photo by P.C. Sokoloff.

*Opuntia polyacantha* was the more common of the two *Opuntia* species recorded near the station, and was frequently encountered in the *Ephedra-Atriplex-Achnatherum* shrubland deserts north of MDRS (Fig. 33). Like *O. basilaris*, *O. polyacantha* is morphologically variable and hybridizes readily. This has resulted in a proliferation of variety names in *O. polyacantha*, including four in Welsh et al. (1993). We follow Pinkava (2003) here, who delineates *O. polyacantha* into five varieties across its range. This species was previously recorded for the nearby San Rafael Swell as *Opuntia polyacantha* (without infraspecific rank) (Harris 1983).

**Supplemental File**: CAN 607489 (Suppl. material 44).

#### Ephedraceae

#### Ephedra viridis

Colville

##### Materials

- **Type status:** Other material. **Occurrence:** recordNumber: 275; recordedBy: Sokoloff, Paul C.; preparations: Silica gel collection; **Taxon:** scientificName: *Ephedra viridis* Colville; kingdom: Plantae; phylum: Gnetophyta; class: Gnetopsida; order: Ephedrales; family: Ephedraceae; genus: Ephedra; specificEpithet: viridis; taxonRank: Species; scientificNameAuthorship: Colville; **Location:** continent: North America; country: United States of America; countryCode: USA; stateProvince: Utah; county: Wayne County; municipality: Hanksville; locality: Mars Desert Research Station; verbatimLocality: "Comm check" hill, 1.7 km northeast of Mars Desert Research Station, just west of Cow Dung Road; verbatimElevation: 1371 m; verbatimLatitude: 38°25'3.15"N; verbatimLongitude: 110°46'54.59"W; coordinateUncertaintyInMeters: 50; **Identification:** identifiedBy: Sokoloff, Paul C.; dateIdentified: 2015; **Event:** verbatimEventDate: November 22, 2014; habitat: Conglomerate sandstone hilltop dominated by *Artemisia* and *Ephedra*; **Record Level:** institutionID: CMN; collectionID: CAN 607468; collectionCode: CAN, UTC; basisOfRecord: Preserved Specimen
- **Type status:** Other material. **Occurrence:** recordNumber: 365; recordedBy: Sokoloff, Paul C.; **Taxon:** scientificName: *Ephedra viridis* Colville; kingdom: Plantae; phylum: Gnetophyta; class: Gnetopsida; order: Ephedrales; family: Ephedraceae; genus: Ephedra; specificEpithet: viridis; scientificNameAuthorship: Colville; **Location:** continent: North America; country: United States of America; countryCode: USA; stateProvince: Utah; county: Wayne County; municipality: Hanksville; locality: Mars Desert Research Station; verbatimLocality: Parking lot of Burpee Dinosaur Quarry, end of Cow Dung Road; verbatimElevation: 1377 m; verbatimLatitude: 38°27'8.24"N; verbatimLongitude: 110°47'27.384"W; coordinateUncertaintyInMeters: 50; **Identification:** identifiedBy: Sokoloff, Paul C.; dateIdentified: 2015; **Event:** verbatimEventDate: September 19, 2015; habitat: Dry sandstone bluffs; **Record Level:** institutionID: CMN; collectionID: CAN 607523; collectionCode: CAN; basisOfRecord: Preserved Specimen

##### Notes

**Figure 34.**
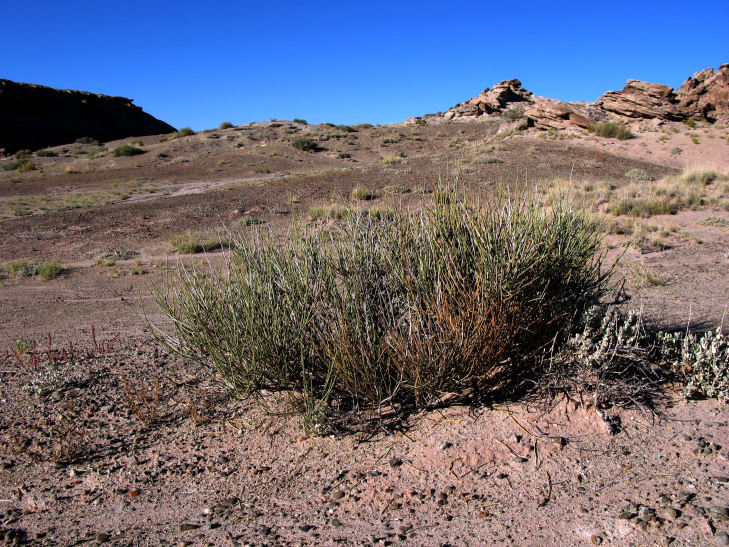
*Ephedra viridis*. Habit. Vicinity of Mars Desert Research Station, Utah, September 19, 2015. Photo by P.C. Sokoloff.

A common species of dry hills and desert slopes (Stevensen 1993), we collected this species along mesa tops and desert scrub communities north of MDRS along Cow Dung Road (Fig. 34). This species was previously reported for the nearby San Rafael Swell (Harris 1983​).

**Supplemental File**: CAN 607468 (Suppl. material 45), CAN 607523 (Suppl. material 46).

#### Euphorbiaceae

#### Euphorbia fendleri

Torr. & A. Gray

##### Materials

- **Type status:** Other material. **Occurrence:** recordNumber: 279; recordedBy: Sokoloff, Paul C.; **Taxon:** scientificName: *Euphorbia fendleri* Torr. & A. Gray; kingdom: Plantae; phylum: Angiosperms; class: Eudicots; order: Malpighiales; family: Euphorbiaceae; genus: Euphorbia; specificEpithet: fendleri; taxonRank: Species; scientificNameAuthorship: Torr. & A. Gray; **Location:** continent: North America; country: United States of America; countryCode: USA; stateProvince: Utah; county: Wayne County; municipality: Hanksville; locality: Mars Desert Research Station; verbatimLocality: "Comm check" hill, 1.7 km northeast of Mars Desert Research Station, just west of Cow Dung Road; verbatimElevation: 1371 m; verbatimLatitude: 38°25'3.15"N; verbatimLongitude: 110°46'54.59"W; coordinateUncertaintyInMeters: 50; **Identification:** identifiedBy: Sokoloff, Paul C.; dateIdentified: 2015; **Event:** verbatimEventDate: November 22, 2014; habitat: Conglomerate sandstone hilltop dominated by *Artemisia* and *Ephedra*; **Record Level:** institutionID: CMN; collectionID: CAN 607464; collectionCode: CAN; basisOfRecord: Preserved Specimen

##### Notes

Widespread throughout Utah (Welsh et al. 1993); a sometimes-used combination exists for this species in *Chamaesyce* (*Chamaesyce fendleri* (Torr. & A. Gray) Small), however molecular evidence firmly places this species within *Euphorbia* (Yang and Berry 2011). This species was previously reported for the nearby San Rafael Swell (Harris 1983).

**Supplemental File**: CAN 607464 (Suppl. material 47).

#### Fabaceae

#### Astragalus amphioxys

A. Gray

##### Materials

- **Type status:** Other material. **Occurrence:** recordNumber: 276; recordedBy: Sokoloff, Paul C.; preparations: Silica gel collection; **Taxon:** scientificName: *Astragalus amphioxys* A. Gray; kingdom: Plantae; phylum: Angiosperms; class: Eudicots; order: Fabales; family: Fabaceae; genus: Astragalus; specificEpithet: amphioxys; taxonRank: Species; scientificNameAuthorship: A. Gray; **Location:** continent: North America; country: United States of America; countryCode: USA; stateProvince: Utah; county: Wayne County; municipality: Hanksville; locality: Mars Desert Research Station; verbatimLocality: "Comm check" hill, 1.7 km northeast of Mars Desert Research Station, just west of Cow Dung Road; verbatimElevation: 1371 m; verbatimLatitude: 38°25'3.15"N; verbatimLongitude: 110°46'54.59"W; coordinateUncertaintyInMeters: 50; **Identification:** identifiedBy: Sokoloff, Paul C.; dateIdentified: 2015; **Event:** verbatimEventDate: November 22, 2014; habitat: Conglomerate sandstone hilltop dominated by *Artemisia* and *Ephedra*; **Record Level:** institutionID: CMN; collectionID: CAN 607473; collectionCode: CAN, UTC; basisOfRecord: Preserved Specimen
- **Type status:** Other material. **Occurrence:** recordNumber: 292; recordedBy: Sokoloff, Paul C.; preparations: Silica gel collection; **Taxon:** scientificName: *Astragalus amphioxys* A. Gray; kingdom: Plantae; phylum: Angiosperms; class: Eudicots; order: Fabales; family: Fabaceae; genus: Astragalus; specificEpithet: amphioxys; taxonRank: Species; scientificNameAuthorship: A. Gray; **Location:** continent: North America; country: United States of America; countryCode: USA; stateProvince: Utah; county: Wayne County; municipality: Hanksville; locality: Mars Desert Research Station; verbatimLocality: Approx. 200 m past fork on Cow Dung Road, down eastern fork, 1.9 km northeast of Mars Desert Research Station; verbatimElevation: 1381 m; verbatimLatitude: 38°25'3.2"N; verbatimLongitude: 110°46'29.7"W; coordinateUncertaintyInMeters: 50; **Identification:** identifiedBy: Sokoloff, Paul C.; dateIdentified: 2015; **Event:** verbatimEventDate: November 23, 2014; habitat: *Artemisia*-dominated desert scrub; **Record Level:** institutionID: CMN; collectionID: CAN 607474; collectionCode: CAN, UTC; basisOfRecord: Preserved Specimen

##### Notes

**Figure 35.**
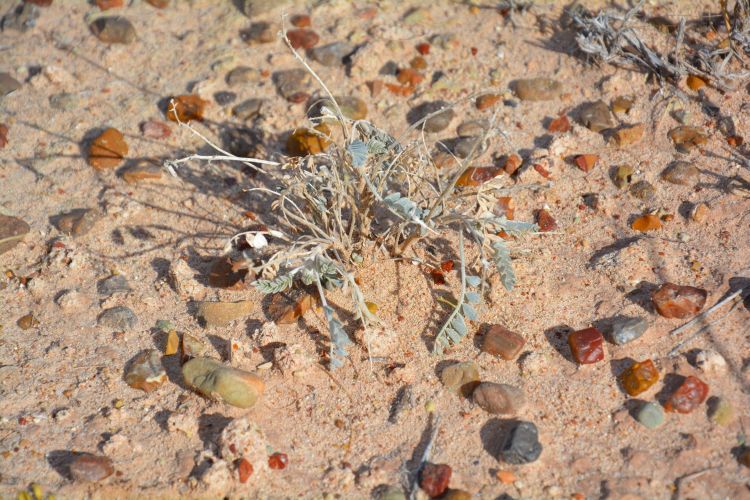
*Astragalus amphioxys* (*Sokoloff 276*). Habit. Photo by P.C. Sokoloff.

This species was common on sandy soil in *Atriplex-Ephedra* communities due north of MDRS (Fig. 35). Harris (1983) reported two varieties of this species from the nearby San Rafael Swell: Astragalus amphioxys var. amphioxys and Astragalus amphioxys var. vespertinus (E. Sheld.) M.E. Jones. Both varieties are recognized in Barneby (1964) and Welsh (2006), which follow a nearly identical taxonomy, however we were unable to determine these collections to variety as the plants were neither flowering nor fruiting.

**Supplemental Files**: CAN 607473 (Suppl. material 48), CAN 607474 (Suppl. material 49).

#### Astragalus desperatus

M.E. Jones

##### Materials

- **Type status:** Other material. **Occurrence:** recordNumber: 295; recordedBy: Sokoloff, Paul C.; preparations: Silica gel collection; **Taxon:** scientificName: *Astragalus desperatus* M.E. Jones; kingdom: Plantae; phylum: Angiosperms; class: Eudicots; order: Fabales; family: Fabaceae; genus: Astragalus; specificEpithet: desperatus; taxonRank: Species; scientificNameAuthorship: M.E. Jones; **Location:** continent: North America; country: United States of America; countryCode: USA; stateProvince: Utah; county: Wayne County; municipality: Hanksville; locality: Mars Desert Research Station; verbatimLocality: Roadside along ATV trail 2 km northeast of Mars Desert Research Station; verbatimElevation: 1348 m; verbatimLatitude: 38°24'53.8"N; verbatimLongitude: 110°46'18"W; coordinateUncertaintyInMeters: 50; **Identification:** identifiedBy: Sokoloff, Paul C.; dateIdentified: 2015; **Event:** verbatimEventDate: November 23, 2014; habitat: Sandstone rubble and sandy plains; **Record Level:** institutionID: CMN; collectionID: CAN 607476; collectionCode: CAN, UTC; basisOfRecord: Preserved Specimen

##### Notes

Harris (1983) reported two varieties of this species from the nearby San Rafael Swell: Astragalus desperatus var. desperatus and Astragalus desperatus var. petrophilus M.E. Jones. Both varieties are accepted in Welsh (2006). Barneby (1964) treats this species as containing var. *desperatus* and var. *conspectus* Barneby, the later of which is synonymous with Welsh's *Astragalus barnebyi* S.L. Welsh & N.D. Atwood (Welsh 2006). As our collection was vegetative, we were unable to identify it to variety.

**Supplemental File**: CAN 607476 (Suppl. material 50).

#### Astragalus lentiginosus

Douglas

##### Materials

- **Type status:** Other material. **Occurrence:** recordNumber: 299; recordedBy: Sokoloff, Paul C.; preparations: Silica gel collection; **Taxon:** scientificName: *Astragalus lentiginosus* Douglas; kingdom: Plantae; phylum: Angiosperms; class: Eudicots; order: Fabales; family: Fabaceae; genus: Astragalus; specificEpithet: lentiginosus; taxonRank: Species; scientificNameAuthorship: Douglas; **Location:** continent: North America; country: United States of America; countryCode: USA; stateProvince: Utah; county: Wayne County; municipality: Hanksville; locality: Mars Desert Research Station; verbatimLocality: Roadside along ATV trail 2 km northeast of Mars Desert Research Station; verbatimElevation: 1348 m; verbatimLatitude: 38°24'53.8"N; verbatimLongitude: 110°46'18"W; coordinateUncertaintyInMeters: 50; **Identification:** identifiedBy: Sokoloff, Paul C.; dateIdentified: 2015; **Event:** verbatimEventDate: November 23, 2014; habitat: Dry sandy streambed, grass-dominated community; **Record Level:** institutionID: CMN; collectionID: CAN 607475; collectionCode: CAN; basisOfRecord: Preserved Specimen

##### Notes

**Figure 36.**
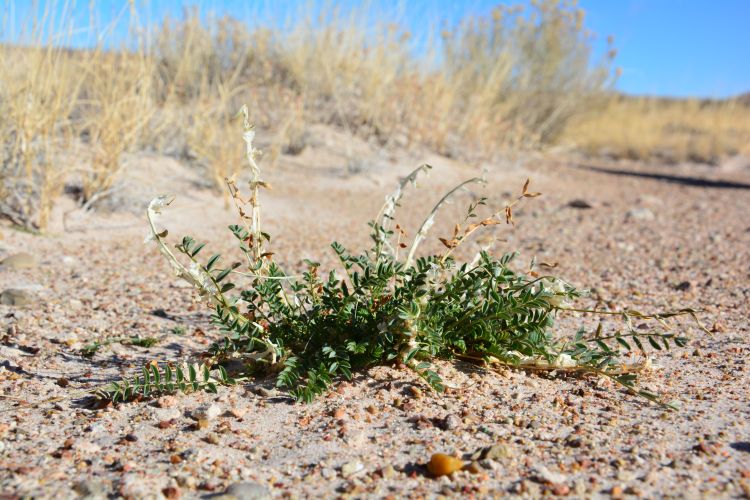
*Astragalus lentiginosus* (*Sokoloff 299*). Habit. Photo by P.C. Sokoloff

This species was only encountered once in the MDRS vicinity, due northwest of MDRS (Fig. 36). Harris (1983) reported two varieties of this species from the nearby San Rafael Swell: Astragalus lentiginosus var. araneosus (E. Sheld.) Barneby and Astragalus lentiginosus var. palans (M.E. Jones) M.E. Jones. Both of these varieties are accepted in both Barneby (1964) and Welsh (2006) however, we were unable to determine our collection to variety as this specimen was vegetative.

**Supplemental File**: CAN 607475 (Suppl. material 51).

#### Juncaceae

#### Juncus bufonius

L.

##### Materials

- **Type status:** Other material. **Occurrence:** recordNumber: 265; recordedBy: Sokoloff, Paul C.; **Taxon:** scientificName: *Juncus bufonius* L.; kingdom: Plantae; phylum: Angiosperms; class: Monocots; order: Poales; family: Juncaceae; genus: Juncus; specificEpithet: bufonius; taxonRank: Species; scientificNameAuthorship: L.; **Location:** continent: North America; country: United States of America; countryCode: USA; stateProvince: Utah; county: Wayne County; municipality: Hanksville; locality: Mars Desert Research Station; verbatimLocality: Dry streambed approx 500 m northeast of Mars Desert Research Station "hab"; verbatimElevation: 1371 m; verbatimLatitude: 38°24'27.7"N; verbatimLongitude: 110°47'20"W; coordinateUncertaintyInMeters: 50; **Identification:** identifiedBy: Sokoloff, Paul C.; dateIdentified: 2015; **Event:** verbatimEventDate: November 20, 2014; habitat: Summit of rocky hill; **Record Level:** institutionID: CMN; collectionID: CAN 607487; collectionCode: CAN, UTC; basisOfRecord: Preserved Specimen

##### Notes

Harris (1983) did not record this species within the nearby San Rafael Swell, but it is reported for both the Glen Canyon National Recreation Area (Hill and Ayers 2009), and Capitol Reef National Park (Fertig 2009). Though common throughout North America (Brooks and Clemants 2000), it is seemingly uncommon in the vicinity of MDRS; this species was only encountered on one low dune directly northeast of the station.

**Supplemental File**: CAN 607487 (Suppl. material 52).

#### Malvaceae

#### Sphaeralcea coccinea

(Nutt.) Rydb.

##### Materials

- **Type status:** Other material. **Occurrence:** recordNumber: 260; recordedBy: Sokoloff, Paul C.; preparations: Silica gel collection; **Taxon:** scientificName: *Sphaeralcea coccinea* (Nutt.) Rydb.; kingdom: Plantae; phylum: Angiosperms; class: Eudicots; order: Malvales; family: Malvaceae; genus: Sphaeralcea; specificEpithet: coccinea; taxonRank: Species; scientificNameAuthorship: (Nutt.) Rydb.; **Location:** continent: North America; country: United States of America; countryCode: USA; stateProvince: Utah; county: Wayne County; municipality: Hanksville; locality: Mars Desert Research Station; verbatimLocality: Vicinity of the Mars Desert Research Station, Hanksville, Utah, 500 m radius of "hab"; verbatimElevation: 1371 m; verbatimLatitude: 38°24'23.2"N; verbatimLongitude: 110°47'31.1"W; coordinateUncertaintyInMeters: 50; **Identification:** identifiedBy: Sokoloff, Paul C.; dateIdentified: 2015; **Event:** verbatimEventDate: November 17, 2014; habitat: Sandy washes and outcrops surrounding MDRS; **Record Level:** institutionID: CMN; collectionID: CAN 607480; collectionCode: CAN, UTC; basisOfRecord: Preserved Specimen

##### Notes

Common in *Atriplex-Ephedra* communities (Welsh et al. 1993), this species was found growing in the disturbed sandy areas immediately surrounding MDRS (Fig. 37). This species was previously recorded in the nearby San Rafael Swell (Harris 1983).

**Supplemental File**: CAN 607480 (Suppl. material 53).

#### Sphaeralcea parviflora

A. Nelson

##### Materials

- **Type status:** Other material. **Occurrence:** recordNumber: 294; recordedBy: Sokoloff, Paul C.; preparations: Silica gel collection; **Taxon:** scientificName: *Sphaeralcea parviflora* A. Nelson; kingdom: Plantae; phylum: Angiosperms; class: Eudicots; order: Malvales; family: Malvaceae; genus: Sphaeralcea; specificEpithet: parviflora; taxonRank: Species; scientificNameAuthorship: A. Nelson; **Location:** continent: North America; country: United States of America; countryCode: USA; stateProvince: Utah; county: Wayne County; municipality: Hanksville; locality: Mars Desert Research Station; verbatimLocality: Approx. 200 m past fork on Cow Dung Road, down eastern fork, 1.9 km northeast of Mars Desert Research Station; verbatimElevation: 1381 m; verbatimLatitude: 38°25'3.2"N; verbatimLongitude: 110°46'29.7"W; coordinateUncertaintyInMeters: 50; **Identification:** identifiedBy: Sokoloff, Paul C.; dateIdentified: 2015; **Event:** verbatimEventDate: November 23, 2014; habitat: *Artemisia*-dominated desert scrub; **Record Level:** institutionID: CMN; collectionID: CAN 607482; collectionCode: CAN; basisOfRecord: Preserved Specimen

##### Notes

This species was previously reported for the nearby San Rafael Swell (Harris 1983​), and is common in desert shrub communities (Welsh et al. 1993).

**Supplemental File**: CAN 607482 (Suppl. material 54).

#### Onagraceae

#### Oenothera cespitosavar.navajoensis

(W.L. Wagner, Stockh. & W.M. Klein) Cronquist

##### Materials

- **Type status:** Other material. **Occurrence:** recordNumber: 258; recordedBy: Sokoloff, Paul C.; preparations: Silica gel collection; **Taxon:** scientificName: Oenothera cespitosa Nutt. var. navajoensis (W.L. Wagner, Stockh. & W.M. Klein) Cronquist; kingdom: Plantae; phylum: Angiosperms; class: Eudicots; order: Myrtales; family: Onagracaeae; genus: Oenothera; specificEpithet: cespitosa; infraspecificEpithet: navajoensis; taxonRank: Variety; scientificNameAuthorship: (W.L. Wagner, Stockh. & W.M. Klein) Cronquist; **Location:** continent: North America; country: United States of America; countryCode: USA; stateProvince: Utah; county: Wayne County; municipality: Hanksville; locality: Mars Desert Research Station; verbatimLocality: Vicinity of the Mars Desert Research Station, Hanksville, Utah, 500 m radius of "hab"; verbatimElevation: 1371 m; verbatimLatitude: 38°24'23.2"N; verbatimLongitude: 110°47'31.1"W; coordinateUncertaintyInMeters: 50; **Identification:** identifiedBy: Sokoloff, Paul C.; dateIdentified: 2015; **Event:** verbatimEventDate: November 17, 2014; habitat: Sandy washes and outcrops surrounding MDRS; **Record Level:** institutionID: CMN; collectionID: CAN 607493; collectionCode: CAN, UTC; basisOfRecord: Preserved Specimen
- **Type status:** Other material. **Occurrence:** recordNumber: 283; recordedBy: Sokoloff, Paul C.; preparations: Silica gel collection; **Taxon:** scientificName: Oenothera cespitosa Nutt. var. navajoensis (W.L. Wagner, Stockh. & W.M. Klein) Cronquist; kingdom: Plantae; phylum: Angiosperms; class: Eudicots; order: Myrtales; family: Onagracaeae; genus: Oenothera; specificEpithet: cespitosa; infraspecificEpithet: navajoensis; taxonRank: Variety; scientificNameAuthorship: (W.L. Wagner, Stockh. & W.M. Klein) Cronquist; **Location:** continent: North America; country: United States of America; countryCode: USA; stateProvince: Utah; county: Wayne County; municipality: Hanksville; locality: Mars Desert Research Station; verbatimLocality: "Comm check" hill, 1.7 km northeast of Mars Desert Research Station, just west of Cow Dung Road; verbatimElevation: 1371 m; verbatimLatitude: 38°25'3.15"N; verbatimLongitude: 110°46'54.59"W; coordinateUncertaintyInMeters: 50; **Identification:** identifiedBy: Sokoloff, Paul C.; dateIdentified: 2015; **Event:** verbatimEventDate: November 22, 2014; habitat: Desert plains; **Record Level:** institutionID: CMN; collectionID: CAN 607499; collectionCode: CAN, UTC; basisOfRecord: Preserved Specimen

##### Notes

**Figure 38.**
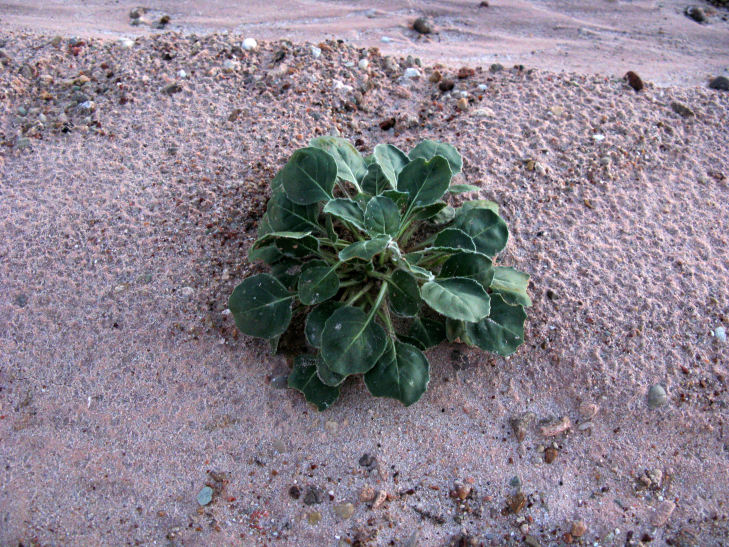
Oenothera cespitosa var. navajoensis. Habit. Vicinity of Mars Desert Research Station, Utah, September 20, 2015. Photo by P.C. Sokoloff.

Common on disturbed sands and desert shrub communities in the vicinity of MDRS (Fig. 38), this species was previously recorded in the nearby San Rafael Swell as *Oenothera caespitosa* Nutt. (Harris 1983). Here we follow Welsh et al. (1993) and treat these specimens as var. *navajoensis*, based on the characteristic fringe of trichomes on the leaf margin.

**Supplemental Files**: CAN 607493 (Suppl. material 55), CAN 607499 (Suppl. material 56).

#### Poaceae

#### Achnatherum hymenoides

(Roem. & Schult.) Barkworth

##### Materials

- **Type status:** Other material. **Occurrence:** recordNumber: 277; recordedBy: Sokoloff, Paul C.; **Taxon:** scientificName: *Achnatherum hymenoides* (Roem. & Schult.) Barkworth; kingdom: Plantae; phylum: Angiosperms; class: Monocots; order: Poales; family: Poaceae; genus: Achnatherum; specificEpithet: hymenoides; taxonRank: Species; scientificNameAuthorship: (Roem. & Schult.) Barkworth; **Location:** continent: North America; country: United States of America; countryCode: USA; stateProvince: Utah; county: Wayne County; municipality: Hanksville; locality: Mars Desert Research Station; verbatimLocality: "Comm check" hill, 1.7 km northeast of Mars Desert Research Station, just west of Cow Dung Road.; verbatimElevation: 1381 m; verbatimLatitude: 38°25'3.15"N; verbatimLongitude: 110°46'54.59"W; coordinateUncertaintyInMeters: 50; **Identification:** identifiedBy: Saarela, Jeffery M.; dateIdentified: 2015; **Event:** verbatimEventDate: November 22, 2014; habitat: Desert plains; **Record Level:** institutionID: CMN; collectionID: CAN 607491; collectionCode: CAN, UTC; basisOfRecord: Preserved Specimen

##### Notes

Recorded from the nearby San Rafael Swell as *Oryzopsis hymenoides* (Roem. & Schult.) Ricker ex Piper (Harris 1983), and treated as *Stipa hymenoides* Roem. & Schult in Welsh et al. (1993) here we follow Barkworth (2007) who recognize the taxon in *Achnatherum*. Common across our study area, we encountered this species throughout the deserts surrounding MDRS (Fig. 39).

**Supplemental File**: CAN 607491 (Suppl. material 57).

#### Aristida purpureavar.longiseta

(Steud.) Vasey

##### Materials

- **Type status:** Other material. **Occurrence:** recordNumber: 310; recordedBy: Sokoloff, Paul C.; **Taxon:** scientificName: Aristida purpurea var. longiseta (Steud.) Vasey; kingdom: Plantae; phylum: Angiosperms; class: Monocots; order: Poales; family: Poaceae; genus: Aristida; specificEpithet: purpurea; infraspecificEpithet: longiseta; taxonRank: Variety; scientificNameAuthorship: (Steud.) Vasey; **Location:** continent: North America; country: United States of America; countryCode: USA; stateProvince: Utah; county: Wayne County; municipality: Hanksville; locality: Mars Desert Research Station; verbatimLocality: Sandstone plateau immediately southwest of Mars Desert Research Station, alongside ATV trail; verbatimElevation: 1412 m; verbatimLatitude: 38°24'22.4"N; verbatimLongitude: 110°47'40.3"W; coordinateUncertaintyInMeters: 50; **Identification:** identifiedBy: Saarela, Jeffery M.; dateIdentified: 2015; **Event:** verbatimEventDate: November 24, 2014; habitat: Dry conglomerate sandstone; **Record Level:** institutionID: CMN; collectionID: CAN 607496; collectionCode: CAN, UTC; basisOfRecord: Preserved Specimen

##### Notes

This species was previously reported for the nearby San Rafael Swell (Harris 1983​). While Welsh et al. (1993) did not recognize subspecies of this taxon in Utah, following Allred (2003) our specimen is identifiable as var. *longiseta*.

**Supplemental File**: CAN 607496 (Suppl. material 58).

#### Bouteloua barbatavar.barbata

Lag.

##### Materials

- **Type status:** Other material. **Occurrence:** recordNumber: 300; recordedBy: Sokoloff, Paul C.; **Taxon:** scientificName: Bouteloua barbata Lag. var. barbata; kingdom: Plantae; phylum: Angiosperms; class: Monocots; order: Poales; family: Poaceae; genus: Bouteloua; specificEpithet: barbata; infraspecificEpithet: barbata; taxonRank: Variety; scientificNameAuthorship: Lag.; **Location:** continent: North America; country: United States of America; countryCode: USA; stateProvince: Utah; county: Wayne County; municipality: Hanksville; locality: Mars Desert Research Station; verbatimLocality: Roadside along ATV trail 2 km northeast of Mars Desert Research Station; verbatimElevation: 1348 m; verbatimLatitude: 38°24'53.8"N; verbatimLongitude: 110°46'18"W; coordinateUncertaintyInMeters: 50; **Identification:** identifiedBy: Saarela, Jeffery M.; dateIdentified: 2015; **Event:** verbatimEventDate: November 23, 2014; habitat: Dry sandy streambed, grass-dominated community; **Record Level:** institutionID: CMN; collectionID: CAN 607492; collectionCode: CAN; basisOfRecord: Preserved Specimen

##### Notes

**Figure 40.**
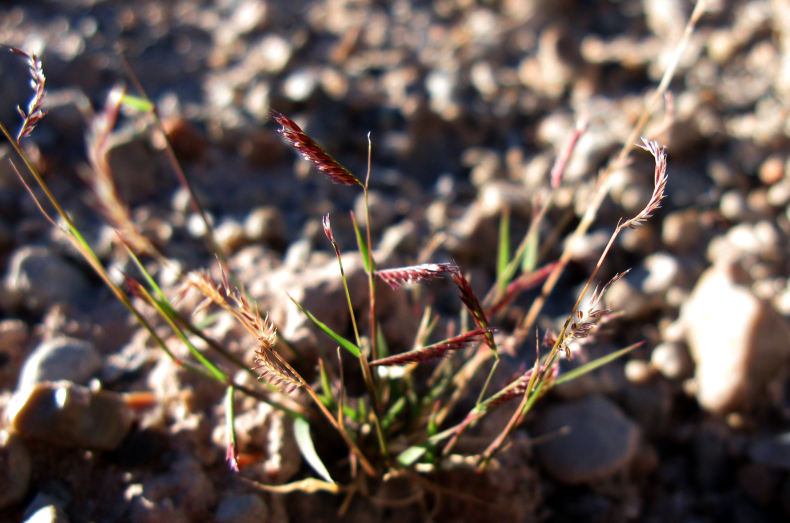
Bouteloua barbata var. barbata. Habit. Vicinity of Mars Desert Research Station, Utah, September 19, 2015. Photo by P.C. Sokoloff.

Though not previously reported in a survey of the nearby San Rafael Swell flora (Harris 1983​), this species was recorded for nearby Capitol Reef National Park (Fertig 2009). This species was collected once in an old, dried wash northeast of MDRS, and photographed in the vicinity of the Burpee Dinosaur quarry at the northern end of Cow Dung Road (Fig. 40). Varieties in *B. barbata* are recognized by both Wipff (2005) and Welsh et al. 1993. The latter treatment asserts that all Utah plants to belong to var. *barbata*.

**Supplemental File**: CAN 607492 (Suppl. material 59).

#### Bromus tectorum

L.

##### Materials

- **Type status:** Other material. **Occurrence:** recordNumber: 297; recordedBy: Sokoloff, Paul C.; **Taxon:** scientificName: *Bromus tectorum* L.; kingdom: Plantae; phylum: Angiosperms; class: Monocots; order: Poales; family: Poaceae; genus: Bromus; specificEpithet: tectorum; taxonRank: Species; scientificNameAuthorship: L.; **Location:** continent: North America; country: United States of America; countryCode: USA; stateProvince: Utah; county: Wayne County; municipality: Hanksville; locality: Mars Desert Research Station; verbatimLocality: Roadside along ATV trail 2 km northeast of Mars Desert Research Station; verbatimElevation: 1348 m; verbatimLatitude: 38°24'53.8"N; verbatimLongitude: 110°46'18"W; coordinateUncertaintyInMeters: 50; **Identification:** identifiedBy: Saarela, Jeffery M.; dateIdentified: 2015; **Event:** verbatimEventDate: November 23, 2014; habitat: Dry sandy streambed, grass-dominated community; **Record Level:** institutionID: CMN; collectionID: CAN 607495; collectionCode: CAN, UTC; basisOfRecord: Preserved Specimen

##### Notes

A noxious weed common throughout the southwestern United States (Pavlick and Anderton 2007), this species was previously reported for the nearby San Rafael Swell (Harris 1983​), and was common in the immediate vicinity of MDRS.

**Supplemental File**: CAN 607495 (Suppl. material 60).

#### Dasyochloa pulchella

(Kunth) Willd. ex Rydb.

##### Materials

- **Type status:** Other material. **Occurrence:** recordNumber: 309; recordedBy: Sokoloff, Paul C.; **Taxon:** scientificName: *Dasyochloa pulchella* (Kunth) Willd. ex Rydb.; kingdom: Plantae; phylum: Angiosperms; class: Monocots; order: Poales; family: Poaceae; genus: Dasyochloa; specificEpithet: pulchella; taxonRank: Species; scientificNameAuthorship: (Kunth) Willd. ex Rydb.; **Location:** continent: North America; country: United States of America; countryCode: USA; stateProvince: Utah; county: Wayne County; municipality: Hanksville; locality: Mars Desert Research Station; verbatimLocality: Sandstone plateau immediately southwest of Mars Desert Research Station, alongside ATV trail; verbatimElevation: 1412 m; verbatimLatitude: 38°24'22.4"N; verbatimLongitude: 110°47'40.3"W; coordinateUncertaintyInMeters: 50; **Identification:** identifiedBy: Saarela, Jeffery M.; dateIdentified: 2015; **Event:** verbatimEventDate: November 24, 2014; habitat: Dry conglomerate sandstone; **Record Level:** institutionID: CMN; collectionID: CAN 607497; collectionCode: CAN, UTC; basisOfRecord: Preserved Specimen

##### Notes

This species was commonly encountered on the plateau immediately southwest of MDRS. Previously reported for the nearby San Rafael Swell as *Erioneuron pulchellum* (Kunth) Tateoka (Harris 1983), recent work has placed this species in the monotypic genus *Dasyochloa* (Caro 1981, Valdés-Reyna 2003).

**Supplemental File**: CAN 607497 (Suppl. material 61).

#### Hilaria jamesii

(Torr.) Benth.

##### Materials

- **Type status:** Other material. **Occurrence:** recordNumber: 255; recordedBy: Sokoloff, Paul C.; **Taxon:** scientificName: *Hilaria jamesii* (Torr.) Benth.; kingdom: Plantae; phylum: Angiosperms; class: Monocots; order: Poales; family: Poaceae; genus: Hilaria; specificEpithet: jamesii; taxonRank: Species; scientificNameAuthorship: (Torr.) Benth.; **Location:** continent: North America; country: United States of America; countryCode: USA; stateProvince: Utah; county: Wayne County; municipality: Hanksville; locality: Mars Desert Research Station; verbatimLocality: Vicinity of the Mars Desert Research Station, Hanksville, Utah, 500 m radius of "hab"; verbatimElevation: 1371 m; verbatimLatitude: 38°24'23.2"N; verbatimLongitude: 110°47'31.1"W; coordinateUncertaintyInMeters: 50; **Identification:** identifiedBy: Saarela, Jeffery M.; dateIdentified: 2015; **Event:** verbatimEventDate: November 17, 2014; habitat: Sandy washes and outcrops surrounding MDRS; **Record Level:** institutionID: CMN; collectionID: CAN 607498; collectionCode: CAN, UTC; basisOfRecord: Preserved Specimen

##### Notes

This desert grass is endemic to the southwestern United States (Barkworth 2005), and was common in the vicinity of MDRS (Fig. 41). This species was previously reported for the nearby San Rafael Swell (Harris 1983).

**Supplemental File**: CAN 607498 (Suppl. material 62).

#### Sporobolus airoides

(Torr.) Torr.

##### Materials

- **Type status:** Other material. **Occurrence:** recordNumber: 298; recordedBy: Sokoloff, Paul C.; **Taxon:** scientificName: *Sporobolus airoides* (Torr.) Torr.; kingdom: Plantae; phylum: Angiosperms; class: Monocots; order: Poales; family: Poaceae; genus: Sporobolus; specificEpithet: airoides; taxonRank: Species; scientificNameAuthorship: (Torr.) Torr.; **Location:** continent: North America; country: United States of America; countryCode: USA; stateProvince: Utah; county: Wayne County; municipality: Hanksville; locality: Mars Desert Research Station; verbatimLocality: Roadside along ATV trail 2 km northeast of Mars Desert Research Station; verbatimElevation: 1348 m; verbatimLatitude: 38°24'53.8"N; verbatimLongitude: 110°46'18"W; coordinateUncertaintyInMeters: 50; **Identification:** identifiedBy: Saarela, Jeffery M.; dateIdentified: 2015; **Event:** verbatimEventDate: November 23, 2014; habitat: Dry sandy streambed, grass-dominated community; **Record Level:** institutionID: CMN; collectionID: CAN 607494; collectionCode: CAN; basisOfRecord: Preserved Specimen

##### Notes

**Figure 42.**
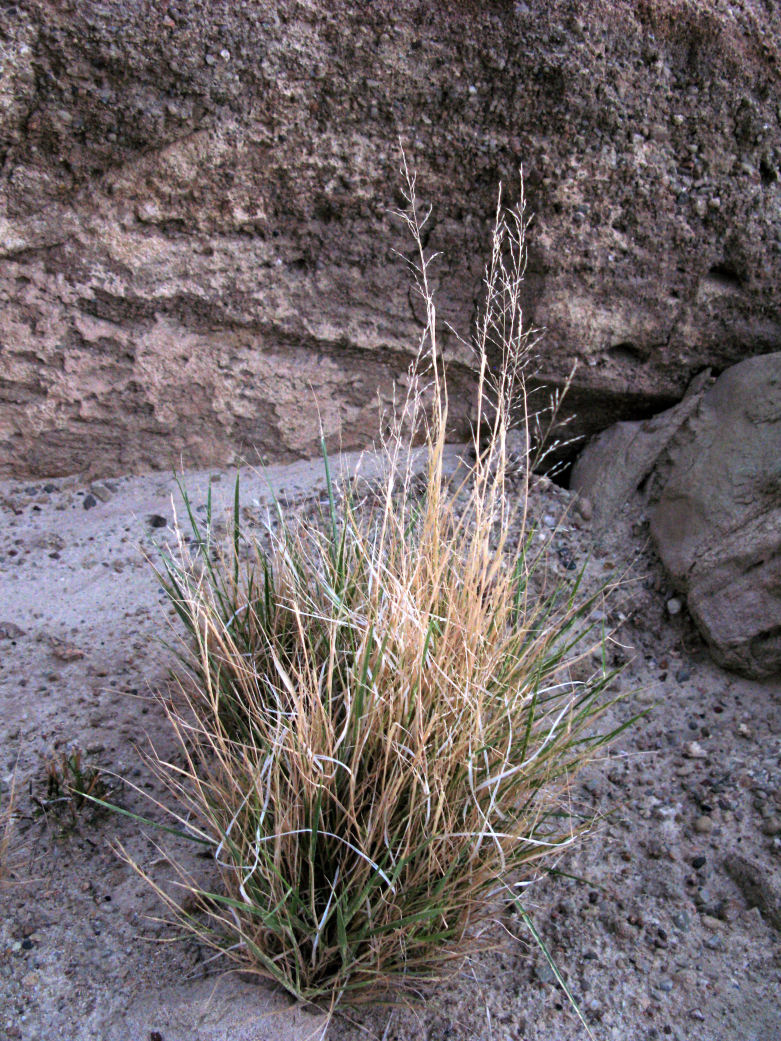
*Sporobolus airoides*. Habit. Vicinity of Mars Desert Research Station, Utah, September 20, 2015. Photo by P.C. Sokoloff.

This species was previously reported for the San Rafael Swell (Harris 1983​), and was encountered on sandy soils north of MDRS (Fig. 42). Welsh et al. (1993) recognize two varieties in *S. airoides*, however Peterson et al. (2003) do not. We follow the latter treatment here.

**Supplemental File**: CAN 607494 (Suppl. material 63).

#### Sporobolus contractus

Hitchc.

##### Materials

- **Type status:** Other material. **Occurrence:** recordNumber: 264; recordedBy: Sokoloff, Paul C.; **Taxon:** scientificName: *Sporobolus contractus* Hitchc.; kingdom: Plantae; phylum: Angiosperms; class: Monocots; order: Poales; family: Poaceae; genus: Sporobolus; specificEpithet: contractus; taxonRank: Species; scientificNameAuthorship: Hitchc.; **Location:** continent: North America; country: United States of America; countryCode: USA; stateProvince: Utah; county: Wayne County; municipality: Hanksville; locality: Mars Desert Research Station; verbatimLocality: Dry streambed approx 500 m northeast of Mars Desert Research Station "hab"; verbatimElevation: 1371 m; verbatimLatitude: 38°24'27.7"N; verbatimCoordinateSystem: 110°47'20"W; coordinateUncertaintyInMeters: 50; **Identification:** identifiedBy: Saarela, Jeffery M.; dateIdentified: 2015; **Event:** verbatimEventDate: November 20, 2014 ; habitat: Rocky sandstone desert; **Record Level:** institutionID: CMN; collectionID: CAN 607501; collectionCode: CAN, UTC; basisOfRecord: Preserved Specimen
- **Type status:** Other material. **Occurrence:** recordNumber: 278; recordedBy: Sokoloff, Paul C.; **Taxon:** scientificName: *Sporobolus contractus* Hitchc.; kingdom: Plantae; phylum: Angiosperms; class: Monocots; order: Poales; family: Poaceae; genus: Sporobolus; specificEpithet: contractus; taxonRank: Species; scientificNameAuthorship: Hitchc.; **Location:** continent: North America; country: United States of America; countryCode: USA; stateProvince: Utah; county: Wayne County; municipality: Hanksville; locality: Mars Desert Research Station; verbatimLocality: "Comm check" hill, 1.7 km northeast of Mars Desert Research Station, just west of Cow Dung Road; verbatimElevation: 1381 m; verbatimLatitude: 38°25'3.15"N; verbatimLongitude: 110°46'54.59"W; coordinateUncertaintyInMeters: 50; **Identification:** identifiedBy: Saarela, Jeffery M.; dateIdentified: 2015; **Event:** verbatimEventDate: November 22, 2014; habitat: Conglomerate sandstone hilltop dominated by *Artemisia* and *Ephedra*; **Record Level:** institutionID: CMN; collectionID: CAN 607500; collectionCode: CAN, UTC; basisOfRecord: Preserved Specimen

##### Notes

Common in the desert shrub communities near MDRS (Fig. 43), and typical of sandy soils and salt deserts (Peterson et al. 2003), this species was previously reported for the nearby San Rafael Swell (Harris 1983​).

**Supplemental Files**: CAN 607501 (Suppl. material 64), CAN 607500 (Suppl. material 65).

#### Polygonaceae

#### Eriogonum inflatum

Torr. & Frém.

##### Materials

- **Type status:** Other material. **Occurrence:** recordNumber: 262; recordedBy: Sokoloff, Paul C.; **Taxon:** scientificName: *Eriogonum inflatum* Torr. & Frém.; kingdom: Plantae; phylum: Angiosperms; class: Eudicots; order: Caryophyllales; family: Polygonaceae; genus: Eriogonum; specificEpithet: inflatum; taxonRank: Species; scientificNameAuthorship: Torr. & Frém.; **Location:** continent: North America; country: United States of America; countryCode: USA; stateProvince: Utah; county: Wayne County; municipality: Hanksville; locality: Mars Desert Research Station; verbatimLocality: Dry streambed approx 500 m northeast of Mars Desert Research Station "hab"; verbatimElevation: 1371 m; verbatimLatitude: 38°24'27.7"N; verbatimLongitude: 110°47'20"W; coordinateUncertaintyInMeters: 50; **Identification:** identifiedBy: Sokoloff, Paul C.; dateIdentified: 2015; **Event:** verbatimEventDate: November 20, 2014; habitat: Eroded elbows of dry streambeds; **Record Level:** institutionID: CMN; collectionID: CAN 607465; collectionCode: CAN, UTC; basisOfRecord: Preserved Specimen
- **Type status:** Other material. **Occurrence:** recordNumber: 268; recordedBy: Sokoloff, Paul C.; **Taxon:** scientificName: Eriogonum inflatum Torr. & Frém.; kingdom: Plantae; phylum: Angiosperms; class: Eudicots; order: Caryophyllales; family: Polygonaceae; genus: Eriogonum; specificEpithet: inflatum; taxonRank: Species; scientificNameAuthorship: Torr. & Frém.; **Location:** continent: North America; country: United States of America; countryCode: USA; stateProvince: Utah; county: Wayne County; municipality: Hanksville; locality: Mars Desert Research Station; verbatimLocality: Dry streambed approx 500 m northeast of Mars Desert Research Station "hab"; verbatimElevation: 1371 m; verbatimLatitude: 38°24'27.7"N; verbatimLongitude: 110°47'20"W; coordinateUncertaintyInMeters: 50; **Identification:** identifiedBy: Sokoloff, Paul C.; dateIdentified: 2015; **Event:** verbatimEventDate: November 20, 2014; habitat: Silty dry streambed; **Record Level:** institutionID: CMN; collectionID: CAN 607486; collectionCode: CAN, UTC; basisOfRecord: Preserved Specimen

##### Notes

**Figure 44.**
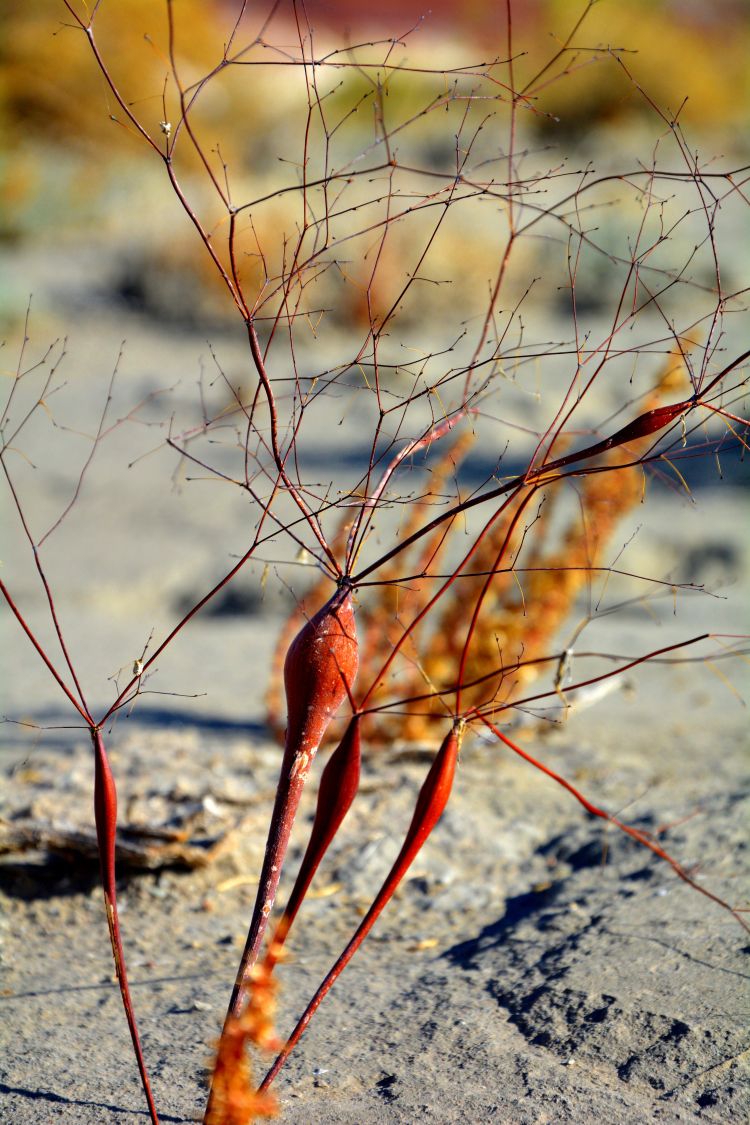
*Eriogonum inflatum* (*Sokoloff 262*). Habit. Photo by P.C. Sokoloff.

This species is known from the nearby San Rafael Swell as two varieties: Eriogonum inflatum var. inflatum and Erigonum inflatum var. fusiforme (Small) Reveal (Harris 1983). These two varieties have been differentiated by substrate (fine textured shales on the Colorado Plateau in var. *fusiforme*, vs. coarse sandstone in var. *inflatum*), root and caudex size, and annual vs. perennial life history (Harris 1983). Reveal (2005), who we follow here, ascribe var. *fusiforme* to an "annual phase", and do not recognize these varieties. *Eriogonum inflatum* was common in the deserts surrounding MDRS (Fig. 44).

**Supplemental Files**: CAN 607465 (Suppl. material 66), CAN 607486 (Suppl. material 67).

#### Eriogonum shockleyi

S. Watson

##### Materials

- **Type status:** Other material. **Occurrence:** recordNumber: 315; recordedBy: Sokoloff, Paul C.; preparations: Silica gel collection; **Taxon:** scientificName: *Eriogonum shockleyi* S. Watson; kingdom: Plantae; phylum: Angiosperms; class: Eudicots; order: Caryophyllales; family: Polygonaceae; genus: Eriogonum; specificEpithet: shockleyi; taxonRank: Species; scientificNameAuthorship: S. Watson; **Location:** continent: North America; country: United States of America; countryCode: USA; stateProvince: Utah; county: Wayne County; municipality: Hanksville; locality: Mars Desert Research Station; verbatimLocality: Sandstone plateau immediately southwest of Mars Desert Research Station, alongside ATV trail; verbatimElevation: 1412 m; verbatimLatitude: 38°24'22.4"N; verbatimLongitude: 110°47'40.3"W; coordinateUncertaintyInMeters: 50; **Identification:** identifiedBy: Sokoloff, Paul C.; dateIdentified: 2015; **Event:** verbatimEventDate: November 24, 2014; habitat: Dry conglomerate sandstone; **Record Level:** institutionID: CMN; collectionID: CAN 607502; collectionCode: CAN, UTC; basisOfRecord: Preserved Specimen

##### Notes

**Figure 45.**
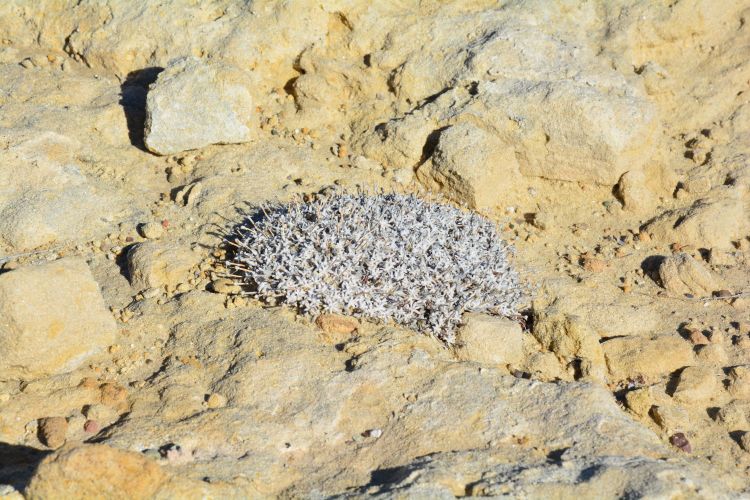
*Eriogonum shockleyi* (*Sokoloff 315*). Habit. Photo by P.C. Sokoloff.

This prostrate plant was encountered on the plateau immediately southwest of MDRS (Fig. 45). This species was previously recorded for the nearby San Rafael Swell as Eriogonum shockleyi var. longilobum (M.E. Jones) S. Stokes (Harris 1983), though varieties are not recognized in later treatments (Welsh et al. 1993, Reveal 2005).

**Supplemental File**: CAN 607502 (Suppl. material 68).

#### Sarcobataceae

#### Sarcobatus vermiculatus

(Hook.) Torr.

##### Materials

- **Type status:** Other material. **Occurrence:** recordNumber: 274; recordedBy: Sokoloff, Paul C.; preparations: Silica gel collection; **Taxon:** scientificName: *Sarcobatus vermiculatus* (Hook.) Torr. ; kingdom: Plantae; phylum: Angiosperms; class: Eudicots; order: Caryophyllales; family: Sarcobataceae; genus: Sarcobatus; specificEpithet: vermiculatus; taxonRank: Species; scientificNameAuthorship: (Hook.) Torr.; **Location:** continent: North America; country: United States of America; countryCode: USA; stateProvince: Utah; county: Wayne County; municipality: Hanksville; locality: Mars Desert Research Station; verbatimLocality: Seasonally wet stream crossing on Cow Dung Road, 1.6 km northeast of Mars Desert Research Station; verbatimElevation: 1371 m; verbatimLatitude: 38°25'55.39"N; verbatimLongitude: 110°47'30.2"W; coordinateUncertaintyInMeters: 50; **Identification:** identifiedBy: Sokoloff, Paul C.; dateIdentified: 2015; **Event:** verbatimEventDate: November 22, 2014; habitat: Desert slopes; **Record Level:** institutionID: CMN; collectionID: CAN 607490; collectionCode: CAN, UTC; basisOfRecord: Preserved Specimen

##### Notes

**Figure 46.**
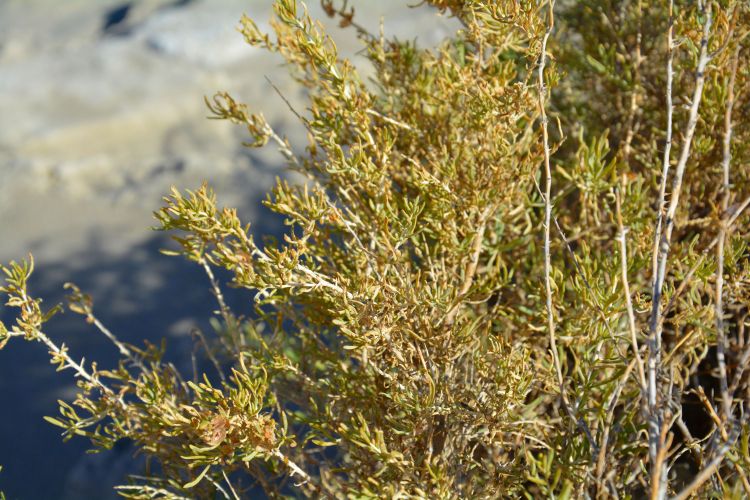
*Sarcobatus vermiculatus* (*Sokoloff 274*). Habit. Photo by P.C. Sokoloff

A common species on alkaline habitats (Hils et al. 1993), *Sarcobatus vermiculatus* was found in greatest abundance along the banks of a seasonally wet stream crossing due northeast of MDRS (Fig. 46). Previously placed within the Chenopodiaceae, *Sarcobatus* is now recognized as the sole genus within the Sarcobataceae (Behnke 1997; APG III 2009). This species was previously recorded for the nearby San Rafael Swell (Harris 1983).

**Supplemental File**: CAN 607490 (Suppl. material 69).

#### Tamaricaceae

#### Tamarix ramosissima

Ledeb.

##### Materials

- **Type status:** Other material. **Occurrence:** recordNumber: 285; recordedBy: Sokoloff, Paul C.; preparations: Silica gel collection; **Taxon:** scientificName: *Tamarix ramosissima* Ledeb.; kingdom: Plantae; phylum: Angiosperms; class: Eudicots; order: Caryophyllales; family: Tamaricaceae; genus: Tamarix; specificEpithet: ramosissima; taxonRank: Species; scientificNameAuthorship: Ledeb.; **Location:** continent: North America; country: United States of America; countryCode: USA; stateProvince: Utah; county: Wayne County; municipality: Hanksville; locality: Mars Desert Research Station; verbatimLocality: Kent's Reservoir, 1.14 km north of Mars Desert Research Station, just west of Cow Dung Road; verbatimElevation: 1371 m; verbatimLatitude: 38°25'28.4"N; verbatimLongitude: 110°47'17.29"W; coordinateUncertaintyInMeters: 50; **Identification:** identifiedBy: Sokoloff, Paul C.; dateIdentified: 2015; **Event:** verbatimEventDate: November 22, 2014; habitat: Moist desert flats; **Record Level:** institutionID: CMN; collectionID: CAN 607466; collectionCode: CAN, UTC; basisOfRecord: Preserved Specimen

##### Notes

*Tamarix chinensis* and *Tamarix ramosissima* are both highly invasive within the western U.S.A. (Gaskin and Kazmer 2009). *Tamarix ramosissima* is often treated as a synonym of *T. chinensis* (Welsh et al. 1993), however genetic evidence supports their treatment as distinct species, albeit with extensive introgression and hybridization across its introduced range in the United States (Gaskin and Kazmer 2009). Plants sampled previously from southeastern Utah have been identified as either back-crossed *T. ramosissima* or F_2_ hybrids (Gaskin and Kazmer 2009); it seems extremely likely that our material would possess a similar genotype.

**Figure 47.**
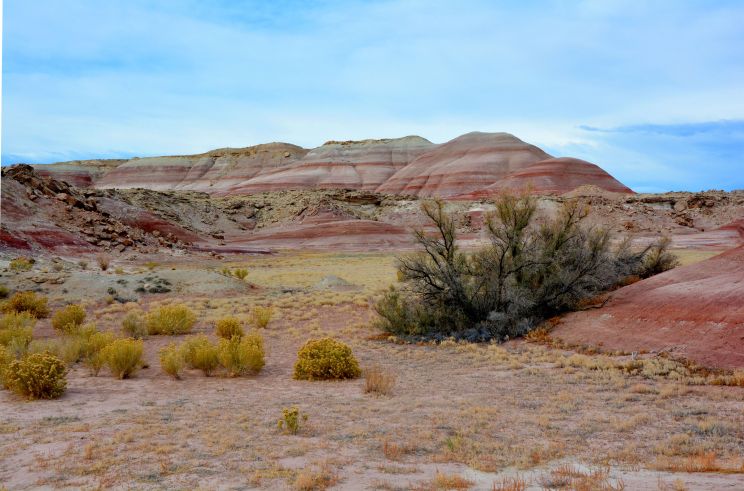
*Tamarix ramosissima* (*Sokoloff 285*). Habit and habitat. The large shub to the centre-right of the photo is *Tamarix ramosissima*, the yellow shrubs to the left are *Ericamerica nauseosa*. Photo by P.C. Sokoloff.

This species has previously been reported for the nearby San Rafael Swell (Harris 1983). Only one population was encountered in the vicinity of MDRS, consisting of three shrubby trees and multiple seedlings, around Kent's Reservoir - a pond on the west side of Cow Dung Road north of MDRS (Fig. 47).

**Supplemental File**: CAN 607466 (Suppl. material 70).

## Analysis

Based on our 2014 and 2015 collections, we recorded 38 vascular plant species from 14 families, 13 lichen species from seven families, five chlorophytes, one cyanobacterium, and one fungus from the MDRS study area (Table 1).

## Discussion

### Vascular Plants

The most species-rich vascular plant families reported from MDRS included the Asteraceae (nine species), and the Poaceae (eight species). These two plant families were also the largest reported for nearby Capitol Reef National Park (Heil et al. 1993). The most species-rich genera in the study area were *Atriplex* and *Astragalus*, both of which are extremely diverse throughout the southwestern states (Stutz 1978, Welsh et al. 1993). We recorded three species in each of these two genera at the site.

In our survey of the vascular plants of MDRS we recorded four species not previously reported for the nearby San Rafael Swell (the best-studied local flora currently available): Chaenactis douglasii var. douglasii, *Hymenoxys cooperi*, *Juncus bufonius*, and Bouteloua barbata var. barbata. These records fill out the known distribution of these plant species in southeastern Utah east of Capitol Reef National Park (for *Chaenactis douglasii* var. *douglasii, Juncus bufonius*, and Bouteloua barbata var. barbata) (Fertig 2009), and north from Glen Canyon Recreation Area (for *Hymenoxys cooperi*) (Hill and Ayers 2009), and contribute to the growing body of knowledge on vascular plant distribution in this region.

Crews at MDRS often must balance multiple research, habitat maintenance, and outreach activities while in simulation. Time spent on EVA is therefore tightly controlled to accomplish these diverse goals, to simulate realistic work pacing, and because radiation exposure will likely limit EVA time on an actual mission to Mars. Therefore, many studies at MDRS take place within close proximity to the hab, and the species present at the hab are likely of primary interest to most investigators. Within the desert flats in the immediate area of the MDRS hab we recorded ten vascular plant species: Atriplex gardneri var. cuneata, *Halogeton glomeratus*, *Gutierrezia sarothrae*, *Juncus bufonius*, *Lepidum montanum*, *Sphaeralcea coccinea*, *Eriogonum inflatum, Juncus bufonius, Sporobolus contractus*, and Oenothera cespitosa var. navajoensis.

On the plateau immediately southwest and on sandstone outcrops immediately north of MDRS, we recorded eight vascular plant species: *Aristida purpurea* var. *longiseta, Atriplex confertifolia, Cryptantha humilis, Dasyochloa pulchella, Eriogonum shockleyi, Gaillardia spathulata, Gutierrezia sarothrae, and Hymenoxys cooperi*. We observed higher vascular plant diversity, and many examples of completely dead, unidentifiable (and therefore not collected) plant species on this plateau than on the desert flats surrounding the station. Therefore researchers at MDRS should take care when identifying vascular plant species from the plateau, as they may not be treated here.

Future floristic work at MDRS should focus on collecting in warmer seasons, when vascular plants are flowering, as this will undoubtedly yield new records for the station. This would also ensure that geophytes, if present, would be recorded. Geophytes are vascular plant species which die back each year and lay dormant underground (Dafni et al. 1981), and are therefore unlikely to be found during a fall/winter survey such as ours. Several geophyte species, notably the sego lily (*Calochortus nuttallii*) have been recorded from both the San Rafael Swell (Harris 1983), and Capitol Reef National Park (Fertig 2009), and thus may be present at MDRS.

### Lichens

Overall, we collected 13 lichen species from nine genera. Given the lichen diversity documented from nearby sites (i.e. Rajvanshi et al. 1998, St. Clair et al. 1991), these 13 species likely represent a small sample of the true lichen diversity at MDRS. Further exploration will be required to generate a more comprehensive local checklist.

The following ten species were collected from the desert flats and outcrops within a 500-meter radius of the research station: *Acarospora rosulata*, *A. stapfiana*, *A. strigata*, *Buellia abstracta*, *Caloplaca trachyphylla*, *Candelariella rosulans*, *Enchylium tenax*, *Placidium acarosporoides*, *P. lachneum*, and *Polysporina gyrocarpa*. *Acarospora strigata* was also collected on the rim of the plateau about 400 meters southwest of, and about 34 meters in altitude above the station. Another three species, *Acarospora peliscypha*, *Heteroplacidium compactum* and *Polysporina gyrocarpa*, were collected along Cow Dung Road between 1.5 and two kilometres north of the station. Ten species were growing on rock, while *Enchylium tenax* and *Placidium lachneum* were collected on sandy soil. Two species, *Acarospora stapfiana* and *Heteroplacidium compactum*, were found growing independently on rock and parasitically on other lichens. We were unable to find previous published reports of *Acarospora peliscypha* for Utah, and the two specimens reported here may represent new records for the state.

The gypsiferous soils of southeastern Utah are well-known habitats for lichen soil crusts (St. Clair et al. 1991) and provide future opportunities to add to the flora of the research area and practice the techniques required to collect delicate species on fragile substrates. As well, lower sagebrush (*Artemisia*) branches on desert flats, and soil in protected, shaded microhabitats should also offer good possibilities of adding to the checklist list of species. Care should be taken to collect good-sized samples with fruiting bodies to aid in making determinations.

### Algae and Cyanobacteria

The rarity of eplithic algae in the Utah desert is not surprising due to the high-to-extreme level of desiccation and light exposure (Rahmonov and Piatek 2007); however the relative abundance of endolithic (cryptoendolithic and chasmoendolithic) algae and photobiont algae was notable from three endolithic samples and two lichen samples. Environmental DNA (eDNA) markers have shown a wide diversity of life forms, from bacteria to eutrophs, in MDRS sub-terrestrial habitats (Direito et al. 2011). This abundance of diversity has also been observed genetically in extreme dry and cold environments (de la Torre et al. 2003, Pointing et al. 2009) and hot thermal environments (Walker et al. 2005). In the Utah desert, endolithic algae layers were close to the substrate surface (<4mm), allowing for adequate light penetration (Matthes et al. 2001). In addition, periods of long dormancy in endolithic microenvironments could further stabilize species-rich communities and niche diversity (Knoll and Grotzinger 2006).

In sandstone microhabitats, the majority of the cells did not show signs of desiccation or stress. *Trebouxia* sp. (Chlorophyceae) had well developed chloroplasts extending across most of the cell. The lobed or plate-like structures of the chloroplasts were often difficult to discern, but occasionally observed. Cultures of these algae from samples *Sokoloff 249* and *Sokoloff 290* also showed well developed chloroplasts that were also difficult to identify (Fig. 4). The central pyrenoid, lobed plate-like chloroplast and thickened cell wall distinguished these *Trebouxia* from other genera, such as *Neochloris* and *Myrmecia*; these latter two genera have similar valve morphologies and genetically similar 18S sequences. Therefore a definitive identification even to the genus is problematic (Komárek and Fott 1983, Friedl and Büdel 2008). Comparable taxa include *N. minuta* Arce & Bold, *N. alveolaris* Bold, *N. pseudoalveolaris* Deason & Bold, *M. astigmatica* Vinatzer, and *M. biatorellae* (Tschermak-Woess. & Plessl) Boye-Pet. 

The number of *Trebouxia* taxa was difficult to discern in this study; four possible taxa were observed based on size, wall sheath thickness, general chloroplast structure and the number of pyrenoids. These taxa likely belong to the *Trebouxia anticipata*/*gelatinosa* ITS rDNA clade (Komárek and Fott 1983, Friedl and Büdel 2008). Lobe and parietal plate-like chloroplast, cell size, wall thickness, and number of pyrenoids are diagnostic for this clade. Zoospores were not observed for any of the chlorophytes.

*Gloeocapsa* sp. (Chroococcales, Microcystaceae) was prominent as a fine layer within the sandstone of *Sokoloff 290* (Fig. 5a, b, c). This cyanobacterium was also dominant as a sub-surface crust (along with a small, prostrate, thallus-forming epiphytic chlorophyte in the Chaetophorales) on the bottom of a quartzite rock found embedded in desert sand near MDRS (*Sokoloff 301*, Fig. 10). Quartzite rocks were infrequently encountered in the deserts surrounding MDRS, and the stone collected likely provided a protected habitat for the cyanobacterial crust while still allowing light transmittance through the translucent mineral of the quartz. Similar stones around MDRS may harbour similar microbial communities.

*Gloeocapsa* colonies form by cell division and binary fusion (Komárek and Anagnostidis 1999). In the two samples collected here (endolithic and epilithic), colonies were densely packed often forming rectangular masses. The multilayered wall sheath characteristic for *Gloeocapsa* was evident in isolated cell clusters (Fig. 5. Cell morphology is very similar to genera within the family Xenococcaceae, but distinguished from them by the absence of multiple fission and baeocyte formation (Komárek and Anagnostidis 1999). Early 16S rDNA results show that *Gloeocapsa* is separated from *Myxosarcina* and *Xenococcus*, which is further separated from *Chroococcidiopsis* (Friedl and Büdel 2008). We did not observe baeocytes (motile or nonmotile), and this combined with the general rectangular-cube formation of the colonies, widely separated cells in the colonies, layers of surrounding sheath and single cells with thick sheaths, leads us to identify this taxon as *Gloeocapsa* sp. This differentiation and identification is subject to challenge with recent DNA studies suggesting that the other genera *Myxosarcina* and *Chroococcidiopsis* are somewhat related (Friedl and Büdel 2008). The DNA results further highlight potential problematic identifications in cultures used for the study, indicating the difficulty in determining differences between taxa in *Gloeocapsa*, *Chroococcidiopsis* and *Myxosarcina*. A number of studies have identified *Chroococcidiopsis* sp. (*sensu lato*) in addition to *Gloeocapsa* sp. as prominent endolithic or epilithic genera (Friedmann 1961, Friedmann 1971, Friedmann et al. 1967, Friedmann and Ocampo-Friedmann 1984, Dor and Danin 1996). In the present study, colony morphology, cell size and form would indicate that we have *Gloeocapsa* sp. Many taxa within *Gloeocapsa* are classified as sub-areal and epilithic. Comparable species include *Gloeocapsa coracina*, *G. decorticans* (A. Braun) Richter, *G. caldariorum* Raben., *G. atrata* Kütz. and *G. bituminosa* (Bory) Kütz..

Two lichen species had hyphae associated with endolithic *Trebouxia* algae layers in our samples (Lecanora cf. garovaglii, *Acarospora strigata*). Two other lichen taxa (*Placidium acarosporoides* and *Heteroplacidium compactum*) had *Myrmecia* sp. as the associated alga with endolithic algae layers and endolithic hyphae in proximity to the surface lichens. The subsurface penetration and sporadic association of fungal threads with the endolithic algae support the observation that biological interactions within the sandstone are part of the ongoing development of lichen taxa (de la Torre et al. 1991). In addition, *Heteroplacidium compactum* is partly parasitic on other crustose lichens, as well as growing independently on rock (Breuss 2007, Prieto et al. 2012).

The primary survival mechanism displayed by endolithic algae in hot conditions is to “hide” from the extreme environment. Like other plant groups, extreme heat causes cellular water loss, which at some point will cause the cell to die. Xeric algae typically have thick sheaths to minimize water loss and environmental abrasion. Many studies have shown the effective tolerance of plant, fungi, and algae cells to desiccation, and the ability to remain viable for long periods of drought in both extreme hot and cold conditions (e.g. Beckett et al. 2008 and references within). Most xeric plants, fungi, and algae have thick cuticles, or protective carbon based coverings for water retention as well as UV protection. The rate of water loss is also critical, if cells are not able to adapt to the environment and their cellular structure (e.g. leaky membranes) cannot control the water loss. In addition to water loss heat can denature functional proteins, while light and heat can induce the cellular production of reactive oxygen species (ROS). Beckett et al. (2008) define many of the ROS as free oxygen radicals. ROS are unstable and a threat to all cellular macromolecules (Fridovich 1999). At least eight ROS species (eg. superoxide O_2_ and hydrogen peroxide H_2_O_2_), are potentially harmful to cellular structure. For an excellent review of general ROS damaging processes to cells refer to Beckett et al. (2008). The endolithic algae observed here do not appear to be stressed and there is no visual evidence of membrane and micro-molecule damage. These algae have either created adapted processes to combat ROH stress or have selected a perfect microhabitat for survival and growth. 

With new developments in taxonomy using DNA sequencing, it would not be surprising to find that endolithic algae communities are more diverse than currently reported. This notion is in contrast to the traditional idea that only a few endolithic algae groups and species are present in the environment (Friedmann et al. 1967). Gerrath et al. (2000) for example, observed 22 endolithic taxa from 180 rock samples along the Niagara escarpment (Ontario, Canada). Already, molecular studies have shown that more than one algae species can be present in one lichen host (Kroken and Taylor 2000), thus more endolithic taxa may be present at MDRS given the number and taxonomic diversity of crustose lichen taxa reported in this study. However, the difficulty in identifying xeric algae based on cell morphology is still problematic without additional culturing and future DNA work, as observed here and in other publications (Friedmann et al. 1967, Thüs et al. 2011). The biological complexity of algae development within the rock, between the rock and surface biota, and the facultative transfer of algae taxa between species illustrates the interwoven connectivity of life in this extreme environment. 

### Biological Sampling at MDRS

While we found it unnecessary to modify standard collection techniques for vascular plant and lichen species, we did note several improvements that would streamline biological sample collection while wearing a simulated spacesuit.

The thick gloves that simulate pressurized material made it difficult to write notes in a fieldbook and operate a handheld GPS receiver. We improvised better collecting workflows during the expedition by using a clipboard and paper datasheets instead of a fieldbook. We kept the GPS receiver on continuously, rather than attempting to operate the touchpad, which was not designed for heavy gloves.

While digital cameras were relatively easy to operate while wearing gloves, it was not possible to use the viewfinder on a DLSR camera while wearing a helmet; we used live view mode on the rear screen of the camera body to compose photographs. Future expeditions may benefit from technological data capture methods, like the voice operated "Mobile Agents" of Clancey et al. (2004), for example.

The presence of an on-site laboratory at MDRS greatly assisted in the processing of plant and lichen samples in a clean, controlled environment, and the availability of microscopes and electronic literature databases allowed us to accurately identify a subset of the vascular plant species during the simulation. The *in situ* facilities allowed us to share preliminary data with mission control, and to decide if follow-up collections at the same site would be required during our mission. This highlights the importance of the laboratory to MDRS, and the utility of including a well-designed laboratory space on a future manned mission to Mars, where efficient execution of a field science program would be of paramount importance.

### Conclusions

In the deserts surrounding MDRS and throughout the southwestern United States of America, the diversity and distribution of vascular plants, lichens, fungi, algae and cyanobacteria are dependent on various factors, including underlying geology (Schenk et al. 2003), the availability of water (Ehleringer et al. 1991) and nutrients (Schlesinger et al. 1996), and the presence of other biological organisms, including vascular plant (Schlesinger and Pilmanis 1998) and soil crust communities (Harper and Belnap 2001). The taxonomic groups treated in this study all possess various adaptations to life in this harsh desert environment, including (but not limited to) thick, moisture retaining cell walls (Holzinger and Karsten 2013) and an endolithic habit (Thielen and Garbary 1999) in algae and cyanobacteria, resistance to UV radiation and dessication in lichens (de Vera et al. 2002), and C4 carbon fixation in vascular plants (Mulroy and Rundel 1977). These adaptations have allowed a diverse floristic comunity to thrive in various microhabitats throughout southeastern Utah, and continued exploration will undoubtedly yield many species not documented here.

Therefore, while our present checklist is not an exhaustive inventory of the MDRS site (greater sampling effort would be necessary to capture all local diversity), it can serve as a first-line reference for identifying vascular plants and lichens at MDRS, and serves as a starting point for future floristic and ecological work at the station. Other useful field references to the MDRS flora include the *Desert Plants of Utah* (Andersen 1996), and *A Field Guide to Biological Soil Crusts of the Western U.S. Drylands* (Rosentreter et al. 2007).

## Supplementary Material

Supplementary material 1 CANL 127960, Acarospora peliscypha (Sokoloff 286) Data type: image
File: oo_60777.JPG PC Sokoloff

Supplementary material 2 CANL 127968, Acarospora rosulata (Sokoloff 303) Data type: image
File: oo_60778.JPG PC Sokoloff

Supplementary material 3 CANL 127958, Acarospora stapfiana (Sokoloff 270) Data type: image
File: oo_60779.JPG PC Sokoloff

Supplementary material 4 CANL 127953, Acarospora strigata (Sokoloff 248) Data type: image
File: oo_60780.JPG PC Sokoloff

Supplementary material 5 CANL 127962, Acarospora strigata (Sokoloff 288) Data type: image
File: oo_60781.JPG PC Sokoloff

Supplementary material 6 CANL 127969, Acarospora strigata (Sokoloff 304) Data type: image
File: oo_60782.JPG PC Sokoloff

Supplementary material 7 CANL 127966, Acarospora strigata (Sokoloff 306) Data type: image
File: oo_60783.JPG PC Sokoloff

Supplementary material 8 CANL 127952, Polysporina gyrocarpa (Sokoloff 247) Data type: image
File: oo_60784.JPG PC Sokoloff

Supplementary material 9 CANL 127954, Polysporina gyrocarpa (Sokoloff 250) Data type: image
File: oo_60785.JPG PC Sokoloff

Supplementary material 10 CANL 127963, Polysporina gyrocarpa (Sokoloff 289) Data type: image
File: oo_60786.JPG PC Sokoloff

Supplementary material 11 CANL 127970, Polysporina gyrocarpa (Sokoloff 286b) Data type: image
File: oo_60787.JPG PC Sokoloff

Supplementary material 12 CANL 127955, Candelariella cf. rosulans (Sokoloff 251) Data type: image
File: oo_60788.JPG PC Sokoloff

Supplementary material 13 CANL 127971, Candelariella rosulans (Sokoloff 288b) Data type: image
File: oo_60789.JPG PC Sokoloff

Supplementary material 14 CANL 127973, Enchylium tenax (Sokoloff 305b) Data type: image
File: oo_60797.JPG PC Sokoloff

Supplementary material 15 CANL 127961, Lecanora garovaglii (Sokoloff 287) Data type: image
File: oo_60790.JPG PC Sokoloff

Supplementary material 16 CANL 127959, Buellia abstracta (Sokoloff 271) Data type: image
File: oo_60792.JPG PC Sokoloff

Supplementary material 17 CANL 127956, Caloplaca trachyphylla (Sokoloff 252) Data type: image
File: oo_60793.JPG PC Sokoloff

Supplementary material 18 CANL 127957, Caloplaca trachyphylla (Sokoloff 269) Data type: image
File: oo_60794.JPG PC Sokoloff

Supplementary material 19 CANL 127964, Heteroplacidium compactum (Sokoloff 296) Data type: image
File: oo_60796.JPG PC Sokoloff

Supplementary material 20 CANL 127965, Placidium acarosporoides (Sokoloff 305) Data type: image
File: oo_60795.JPG PC Sokoloff

Supplementary material 21 CANL 127972, Placidium lachneum (Sokoloff 305c) Data type: image
File: oo_60798.JPG PC Sokoloff

Supplementary material 22 CAN 607477, Atriplex confertifolia (Sokoloff 313) Data type: image
File: oo_60800.jpg PC Sokoloff

Supplementary material 23 CAN 607503, Atriplex corrugata (Sokoloff 273) Data type: image
File: oo_77648.jpg PC Sokoloff

Supplementary material 24 CAN 607507, Atriplex gardneri var. cuneata (Sokoloff 256) Data type: image
File: oo_60802.jpg PC Sokoloff

Supplementary material 25 CAN 607505, Atriplex gardneri var. cuneata (Sokoloff 259) Data type: image
File: oo_60803.jpg PC Sokoloff

Supplementary material 26 CAN 607506, Atriplex gardneri var. cuneata (Sokoloff 266) Data type: image
File: oo_60804.jpg PC Sokoloff

Supplementary material 27 CAN 607484, Halogeton glomeratus (Sokoloff 254) Data type: image
File: oo_60805.jpg PC Sokoloff

Supplementary material 28 CAN 607485, Halogeton glomeratus (Sokoloff 267) Data type: image
File: oo_60806.jpg PC Sokoloff

Supplementary material 29 CAN 607478, Artemisia filifolia (Sokoloff 282) Data type: image
File: oo_77639.jpg PC Sokoloff

Supplementary material 30 CAN 607472, Chaenactis douglasii var. douglasii (Sokoloff 291) Data type: image
File: oo_60808.jpg PC Sokoloff

Supplementary material 31 CAN 607522, Dieteria canescens var. canescens (Sokoloff 366) Data type: image
File: oo_60809.jpg PC Sokoloff

Supplementary material 32 CAN 607481, Ericameria nauseosa (Sokoloff 284) Data type: image
File: oo_77640.jpg PC Sokoloff

Supplementary material 33 CAN 607524, Gaillardia spathulata (Sokoloff 367) Data type: image
File: oo_60811.jpg PC Sokoloff

Supplementary material 34 CAN 607462, Gutierrezia sarothrae (Sokoloff 253) Data type: image
File: oo_60812.jpg PC Sokoloff

Supplementary material 35 CAN 607469, Gutierrezia sarothrae (Sokoloff 257) Data type: image
File: oo_60813.jpg PC Sokoloff

Supplementary material 36 CAN 607463, Gutierrezia sarothrae (Sokoloff 311) Data type: image
File: oo_60814.jpg PC Sokoloff

Supplementary material 37 CAN 607483, Hymenoxys cooperi (Sokoloff 312) Data type: image
File: oo_60815.jpg PC Sokoloff

Supplementary material 38 CAN 607479, Scabrethia scabra (Sokoloff 280) Data type: image
File: oo_77646.jpg PC Sokoloff

Supplementary material 39 CAN 607470, Thelesperma subnudum (Sokoloff 281) Data type: image
File: oo_77647.jpg PC Sokoloff

Supplementary material 40 CAN 607504, Cryptantha humilis (Sokoloff 314) Data type: image
File: oo_60818.jpg PC Sokoloff

Supplementary material 41 CAN 607467, Lepidium montanum (Sokoloff 261) Data type: image
File: oo_60819.jpg PC Sokoloff

Supplementary material 42 CAN 607471, Lepidium montanum (Sokoloff 263) Data type: image
File: oo_60820.jpg PC Sokoloff

Supplementary material 43 CAN 607488, Opuntia basilaris var. basilaris (Sokoloff 272) Data type: image
File: oo_77641.jpg PC Sokoloff

Supplementary material 44 CAN 607489, Opuntia polyacantha var. polyacantha (Sokoloff 293) Data type: image
File: oo_60822.jpg PC Sokoloff

Supplementary material 45 CAN 607468, Ephedra viridis (Sokoloff 275) Data type: image
File: oo_77650.jpg PC Sokoloff

Supplementary material 46 CAN 607523, Ephedra viridis (Sokoloff 365) Data type: image
File: oo_60824.jpg PC Sokoloff

Supplementary material 47 CAN 607464, Euphorbia fendleri (Sokoloff 279) Data type: image
File: oo_77643.jpg PC Sokoloff

Supplementary material 48 CAN 607473, Astragalus amphioxys (Sokoloff 276) Data type: image
File: oo_77653.jpg PC Sokoloff

Supplementary material 49 CAN 607474, Astragalus amphioxys (Sokoloff 292) Data type: image
File: oo_60827.jpg PC Sokoloff

Supplementary material 50 CAN 607476, Astragalus desperatus (Sokoloff 295) Data type: image
File: oo_60828.jpg PC Sokoloff

Supplementary material 51 CAN 607475, Astragalus lentiginosus (Sokoloff 299) Data type: image
File: oo_60829.jpg PC Sokoloff

Supplementary material 52 CAN 607487, Juncus bufonius (Sokoloff 265) Data type: image
File: oo_60830.jpg PC Sokoloff

Supplementary material 53 CAN 607480, Sphaeralcea coccinea (Sokoloff 260) Data type: image
File: oo_60831.jpg PC Sokoloff

Supplementary material 54 CAN 607482, Sphaeralcea parviflora (Sokoloff 294) Data type: image
File: oo_60832.jpg PC Sokoloff

Supplementary material 55 CAN 607493, Oenothera cespitosa var. navajoensis (Sokoloff 258) Data type: image
File: oo_60833.jpg PC Sokoloff

Supplementary material 56 CAN 607499, Oenothera cespitosa var. navajoensis (Sokoloff 283) Data type: image
File: oo_77654.JPG PC Sokoloff

Supplementary material 57 CAN 607491, Achnatherum hymenoides (Sokoloff 277) Data type: image
File: oo_77652.jpg PC Sokoloff

Supplementary material 58 CAN 607496, Aristida purpurea var. longiseta (Sokoloff 310) Data type: image
File: oo_60836.jpg PC Sokoloff

Supplementary material 59 CAN 607492, Bouteloua barbata var. barbata (Sokoloff 300) Data type: image
File: oo_60837.jpg PC Sokoloff

Supplementary material 60 CAN 607495, Bromus tectorum (Sokoloff 297) Data type: image
File: oo_60838.jpg PC Sokoloff

Supplementary material 61 CAN 607497, Dasyochloa pulchella (Sokoloff 309) Data type: image
File: oo_60839.jpg PC Sokoloff

Supplementary material 62 CAN 607498, Hilaria jamesii (Sokoloff 255) Data type: image
File: oo_60840.jpg PC Sokoloff

Supplementary material 63 CAN 607494, Sporobolus airoides (Sokoloff 298) Data type: image
File: oo_60841.jpg PC Sokoloff

Supplementary material 64 CAN 607501, Sporobolus contractus (Sokoloff 264) Data type: image
File: oo_60842.jpg PC Sokoloff

Supplementary material 65 CAN 607500, Sporobolus contractus (Sokoloff 278) Data type: image
File: oo_77655.jpg PC Sokoloff

Supplementary material 66 CAN 607465, Eriogonum inflatum (Sokoloff 262) Data type: image
File: oo_60844.jpg PC Sokoloff

Supplementary material 67 CAN 607486, Eriogonum inflatum (Sokoloff 268) Data type: image
File: oo_60845.jpg PC Sokoloff

Supplementary material 68 CAN 607502, Eriogonum shockleyi (Sokoloff 315) Data type: image
File: oo_60846.jpg PC Sokoloff

Supplementary material 69 CAN 607490, Sarcobatus vermiculatus (Sokoloff 274) Data type: image
File: oo_77651.jpg PC Sokoloff

Supplementary material 70 CAN 607466, Tamarix ramosissima (Sokoloff 285) Data type: image
File: oo_77644.jpg PC Sokoloff

XML Treatment for Chlorophyta

XML Treatment for Chlorophyta

XML Treatment for Trebouxia sp. 1

XML Treatment for Trebouxia sp. 2

XML Treatment for Trebouxia sp. 3

XML Treatment for Trebouxia sp. 4

XML Treatment for Myrmecia sp.

XML Treatment for Cyanobacteria

XML Treatment for Gloeocapsa sp.

XML Treatment for Agaricaceae

XML Treatment for Tulostoma sp.

XML Treatment for Acarosporaceae

XML Treatment for Acarospora peliscypha

XML Treatment for Acarospora rosulata

XML Treatment for Acarospora stapfiana

XML Treatment for Acarospora strigata

XML Treatment for Polysporina gyrocarpa

XML Treatment for Candelariaceae

XML Treatment for Candelariella rosulans

XML Treatment for Collemataceae

XML Treatment for Enchylium tenax

XML Treatment for Lecanoraceae

XML Treatment for Lecanora garovaglii

XML Treatment for Physicaceae

XML Treatment for Buellia abstracta

XML Treatment for Teloschistaceae

XML Treatment for Caloplaca trachyphylla

XML Treatment for Verrucariaceae

XML Treatment for Heteroplacidium compactum

XML Treatment for Placidium acarosporoides

XML Treatment for Placidium lachneum

XML Treatment for Amaranthaceae

XML Treatment for Atriplex confertifolia

XML Treatment for Atriplex corrugata

XML Treatment for Atriplex gardnerivar.cuneata

XML Treatment for Halogeton glomeratus

XML Treatment for Kali tragus

XML Treatment for Asteraceae

XML Treatment for Artemisia filifolia

XML Treatment for Chaenactis douglasiivar.douglasii

XML Treatment for Dieteria canescensvar.canescens

XML Treatment for Ericameria nauseosa

XML Treatment for Gaillardia spathulata

XML Treatment for Gutierrezia sarothrae

XML Treatment for Hymenoxys cooperi

XML Treatment for Scabrethia scabra

XML Treatment for Thelesperma subnudum

XML Treatment for Boraginaceae

XML Treatment for Cryptantha humilis

XML Treatment for Brassicaceae

XML Treatment for Lepidium montanum

XML Treatment for Cactaceae

XML Treatment for Opuntia basilarisvar.basilaris

XML Treatment for Opuntia polyacanthavar.polyacantha

XML Treatment for Ephedraceae

XML Treatment for Ephedra viridis

XML Treatment for Euphorbiaceae

XML Treatment for Euphorbia fendleri

XML Treatment for Fabaceae

XML Treatment for Astragalus amphioxys

XML Treatment for Astragalus desperatus

XML Treatment for Astragalus lentiginosus

XML Treatment for Juncaceae

XML Treatment for Juncus bufonius

XML Treatment for Malvaceae

XML Treatment for Sphaeralcea coccinea

XML Treatment for Sphaeralcea parviflora

XML Treatment for Onagraceae

XML Treatment for Oenothera cespitosavar.navajoensis

XML Treatment for Poaceae

XML Treatment for Achnatherum hymenoides

XML Treatment for Aristida purpureavar.longiseta

XML Treatment for Bouteloua barbatavar.barbata

XML Treatment for Bromus tectorum

XML Treatment for Dasyochloa pulchella

XML Treatment for Hilaria jamesii

XML Treatment for Sporobolus airoides

XML Treatment for Sporobolus contractus

XML Treatment for Polygonaceae

XML Treatment for Eriogonum inflatum

XML Treatment for Eriogonum shockleyi

XML Treatment for Sarcobataceae

XML Treatment for Sarcobatus vermiculatus

XML Treatment for Tamaricaceae

XML Treatment for Tamarix ramosissima

## Figures and Tables

**Figure 4a.**
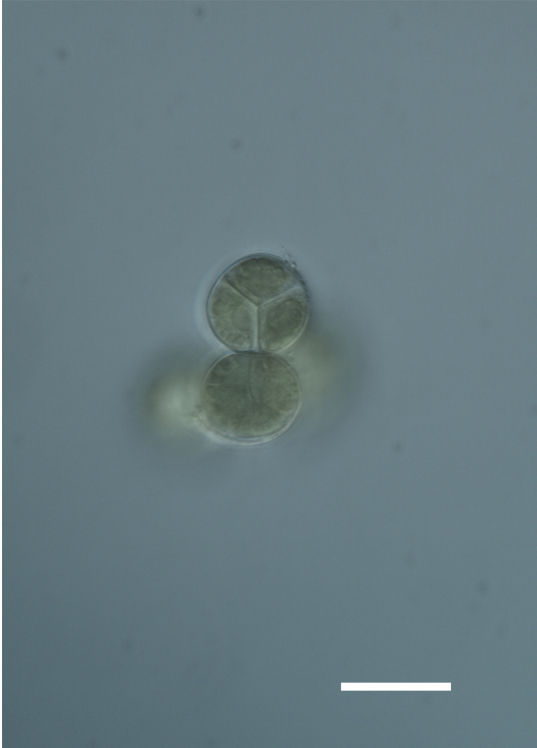
*Trebouxia* sp. 1. Endolithic cells from sandstone.

**Figure 4b.**
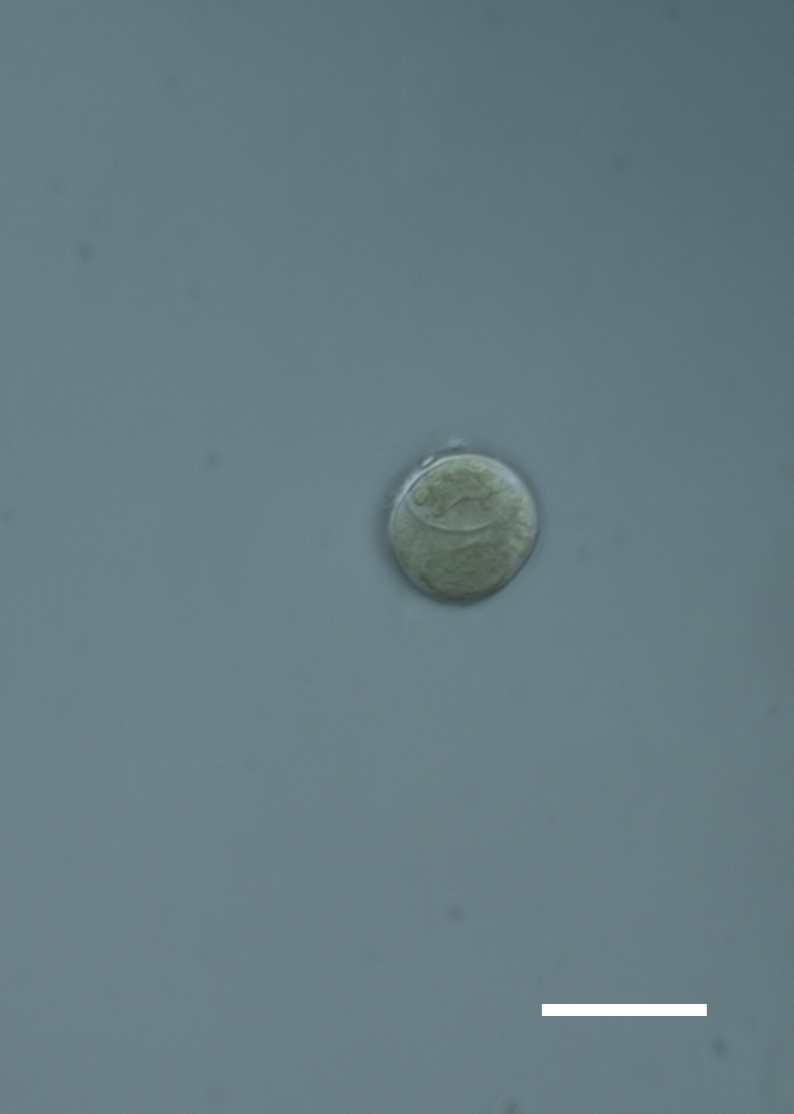
*Trebouxia* sp. 1. Cultured spherical cells showing single and colony forming cells.

**Figure 4c.**
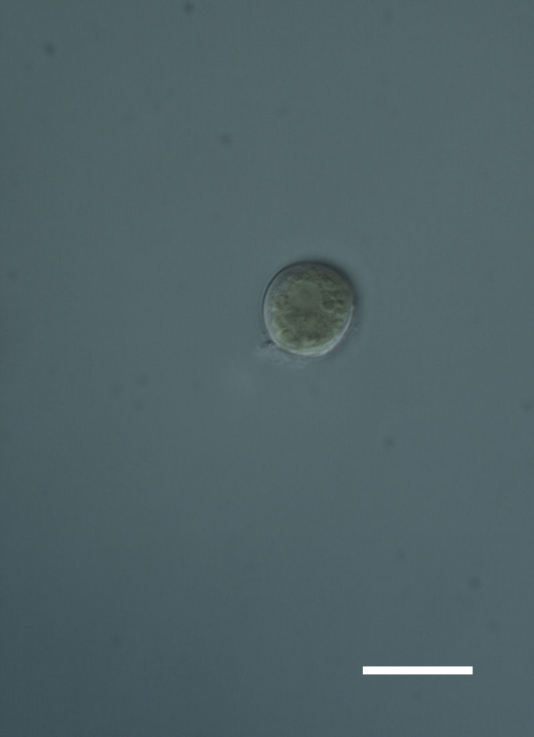
*Trebouxia* sp. 1. Cultured single cell showing lobed chloroplast plate with central pyrenoid.

**Figure 4d.**
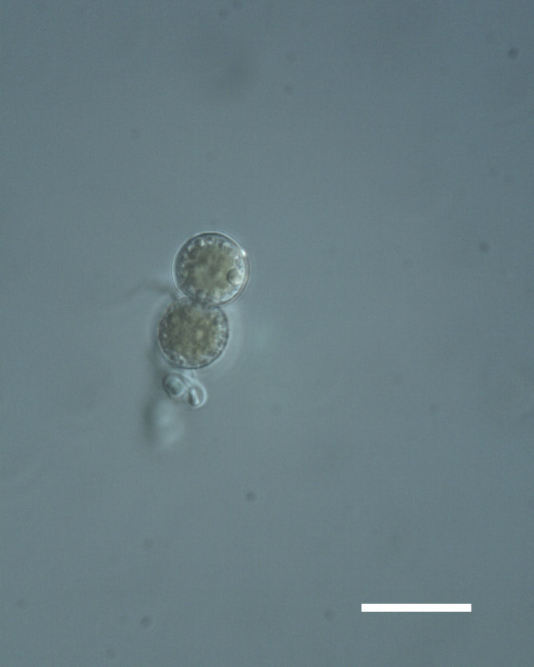
*Trebouxia* sp. 4. Two cells grouped together.

**Figure 4e.**
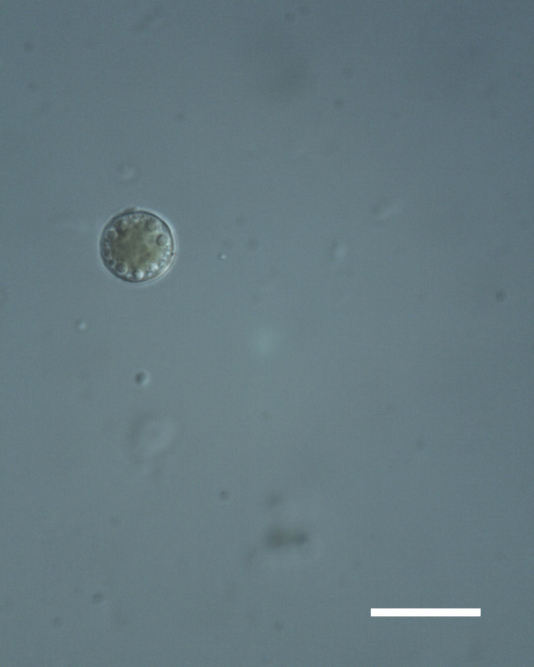
*Trebouxia* sp. 4. Single cell showing lobed chloroplast with one to 5+ pyrenoids.

**Figure 4f.**
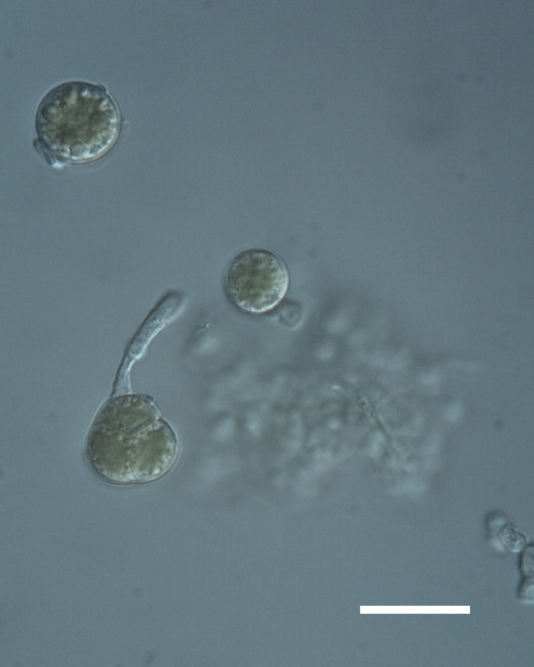
*Trebouxia* sp. 4. Fungal hyphae associated with cells.

**Figure 5a.**
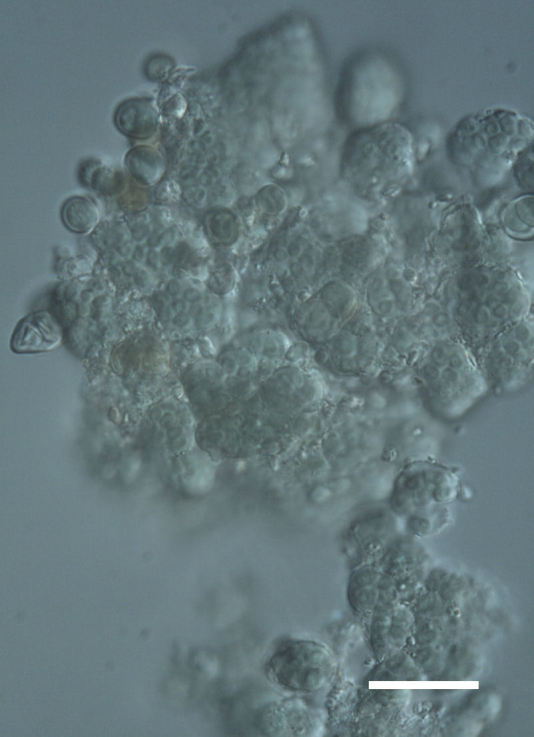
*Gloeocapsa* sp. Compact rectangular to sub-rectangular colonies with scattered single cells.

**Figure 5b.**
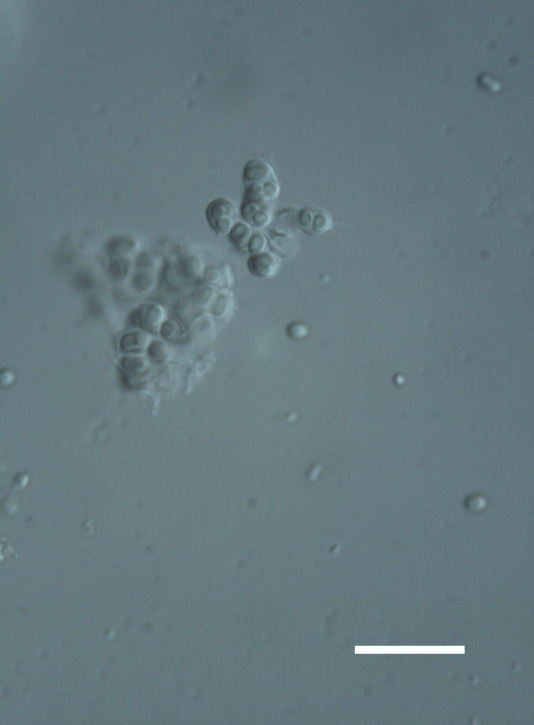
*Gloeocapsa* sp. Small colonies less than or equal to 4 cells. Wall sheath thick.

**Figure 5c.**
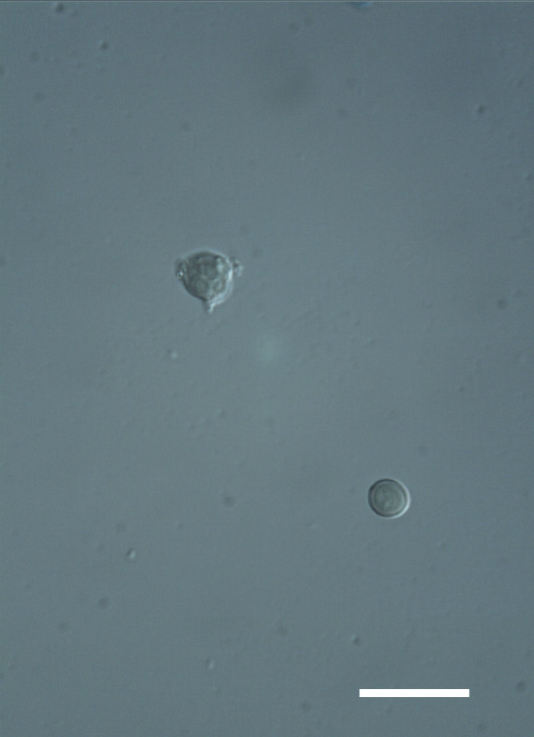
*Gloeocapsa* sp. Single cell and small colony showing thick sheath.

**Figure 6a.**
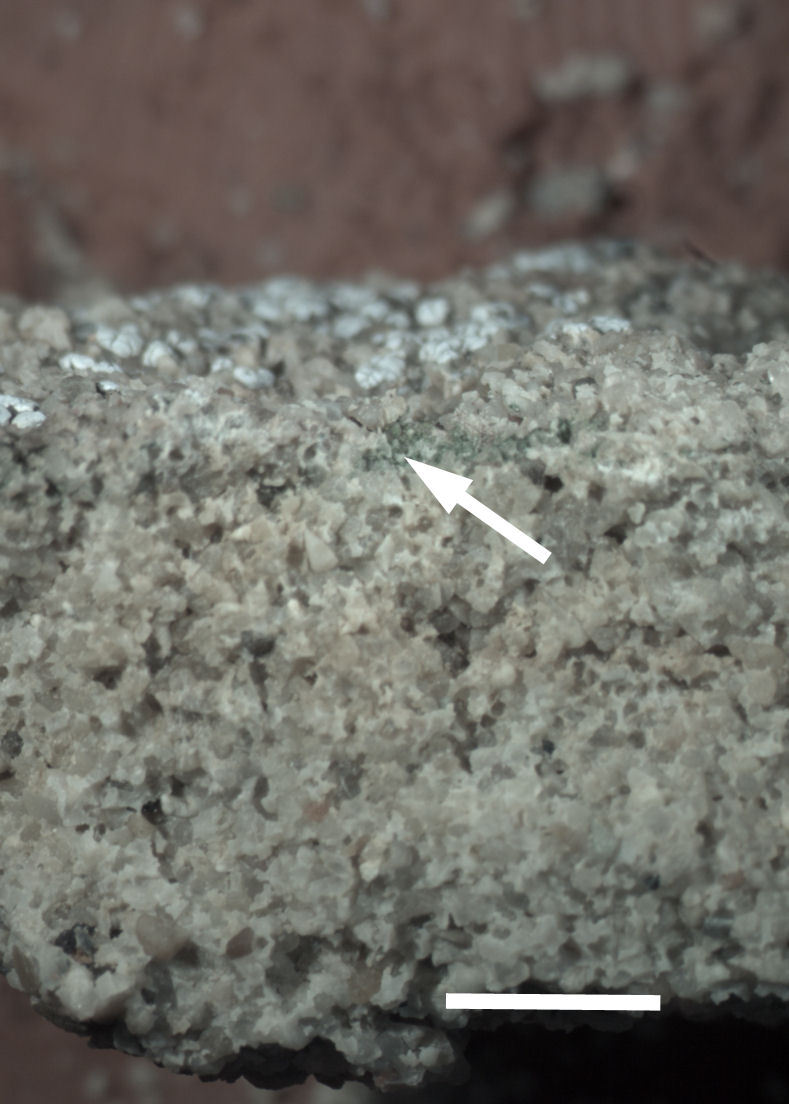
Endolithic subsurface layer of *Trebouxia* sp. 1 (*Sokoloff 249*, arrow). Scale bar = 5 mm.

**Figure 6b.**
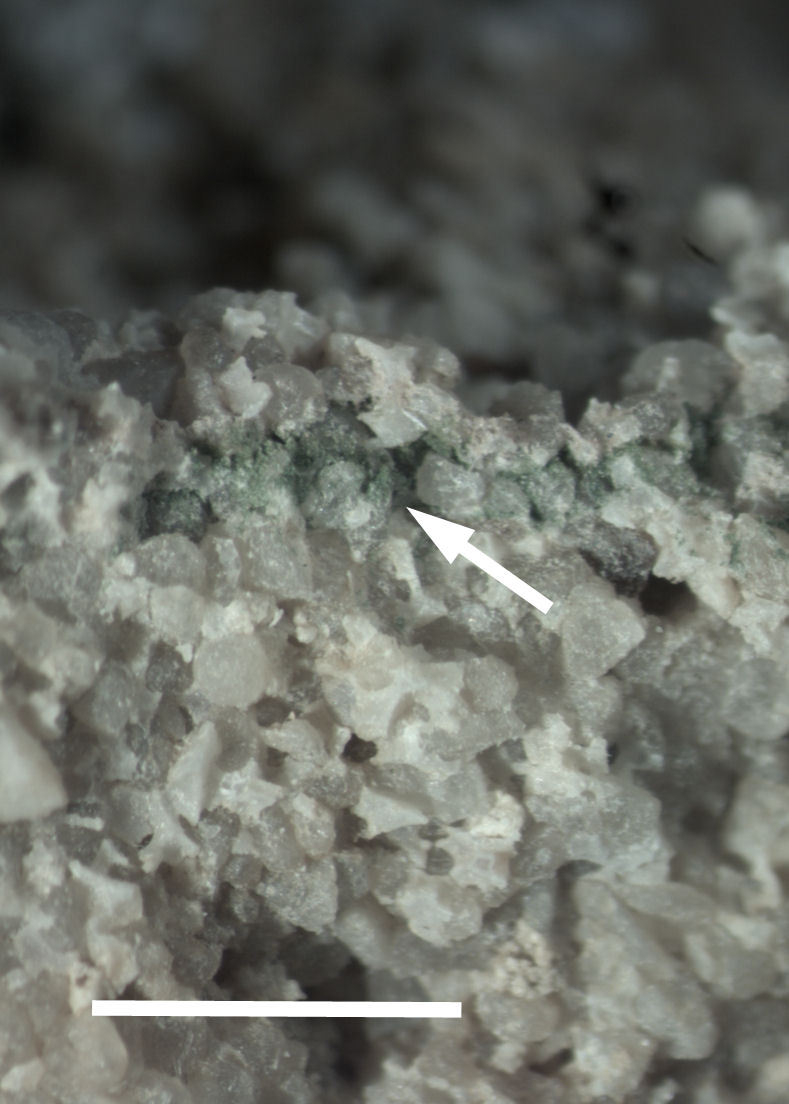
Endolithic subsurface layer of *Gloeocapsa* sp. 1 (*Sokoloff 290*, arrow). Scale bar = 2.5 mm.

**Figure 6c.**
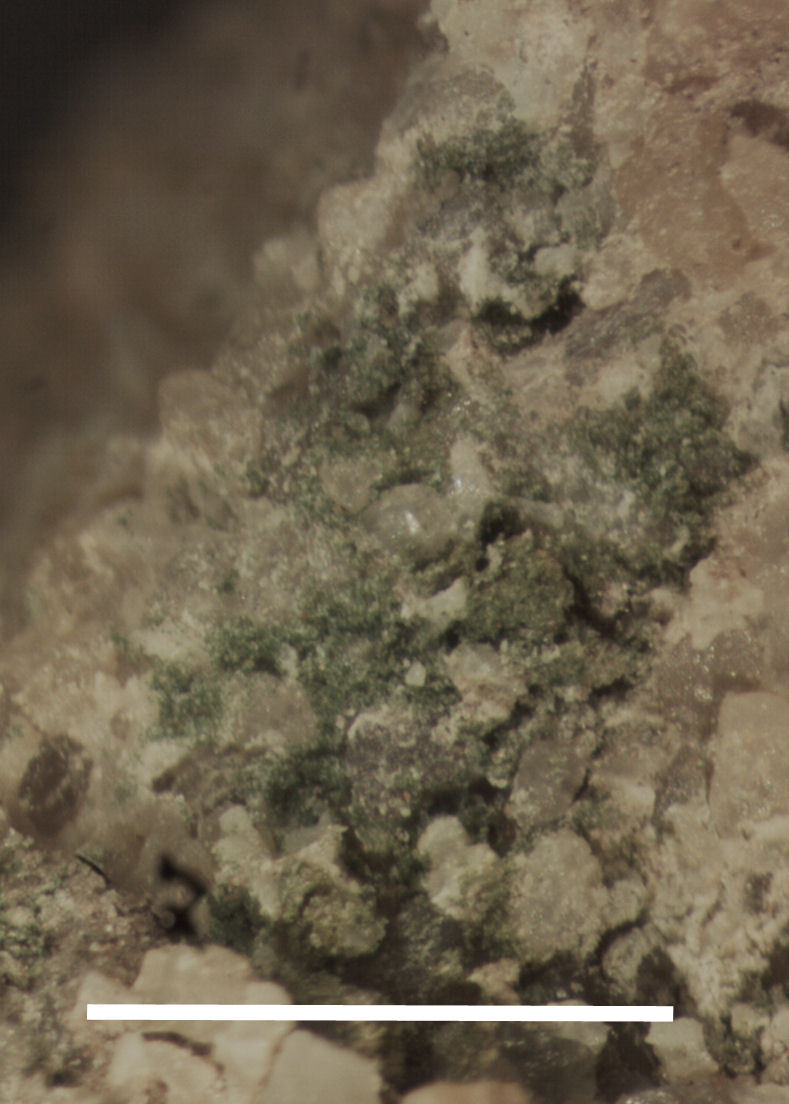
Exposed *Trebouxia* sp. Scale bar = 2.5 mm.

**Figure 6d.**
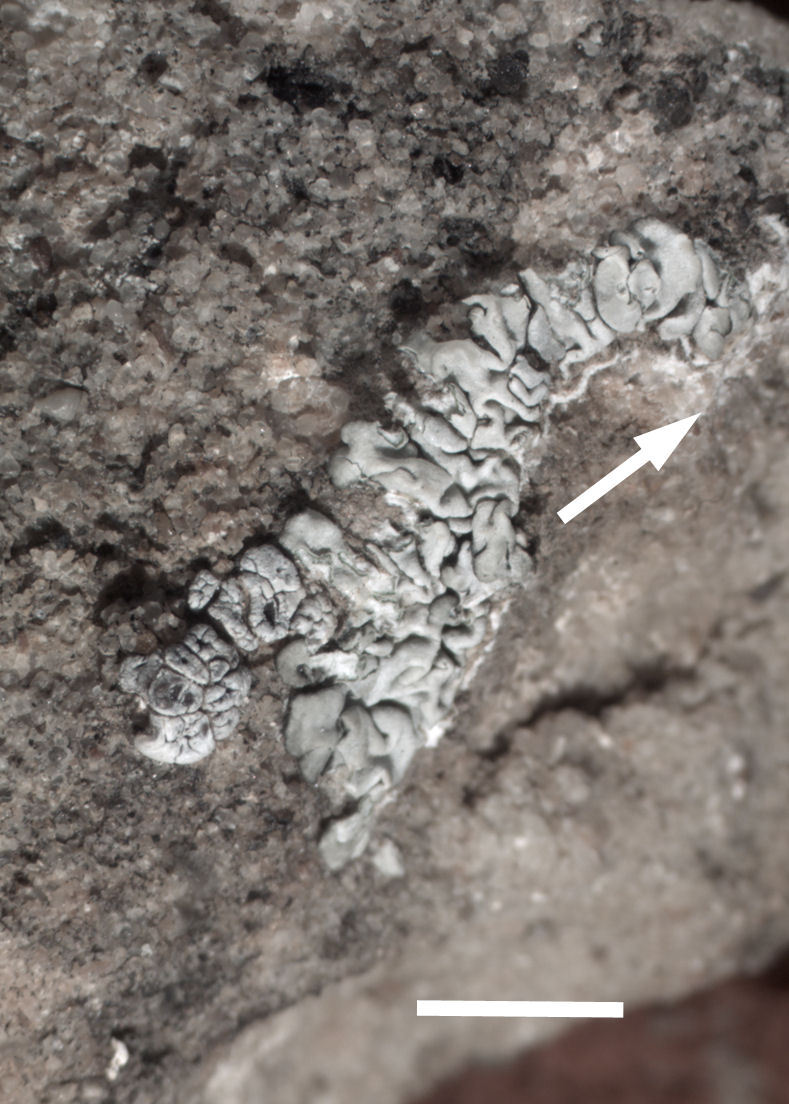
*Lecanora garovaglii* with residual fungal hyphae (arrow). Scale bar = 5 mm.

**Figure 6e.**
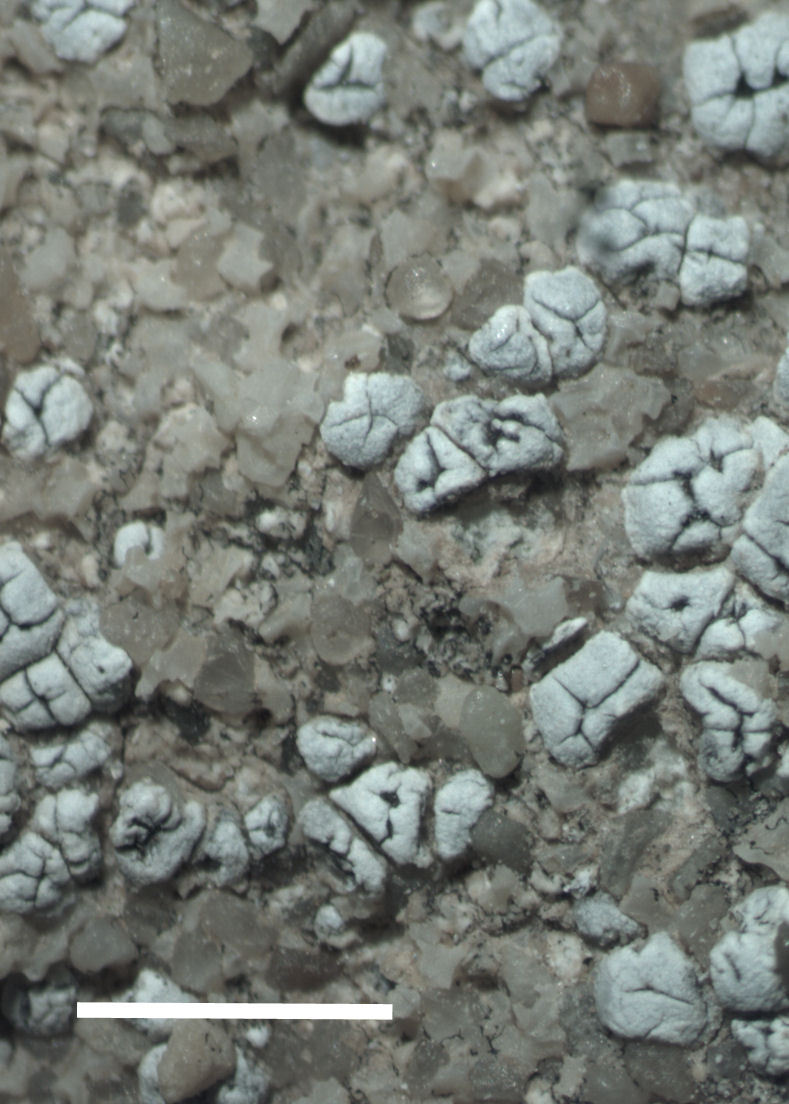
Lichen, Verrucariaceae family, scattered squamules. Scale bar = 2.5 mm.

**Figure 6f.**
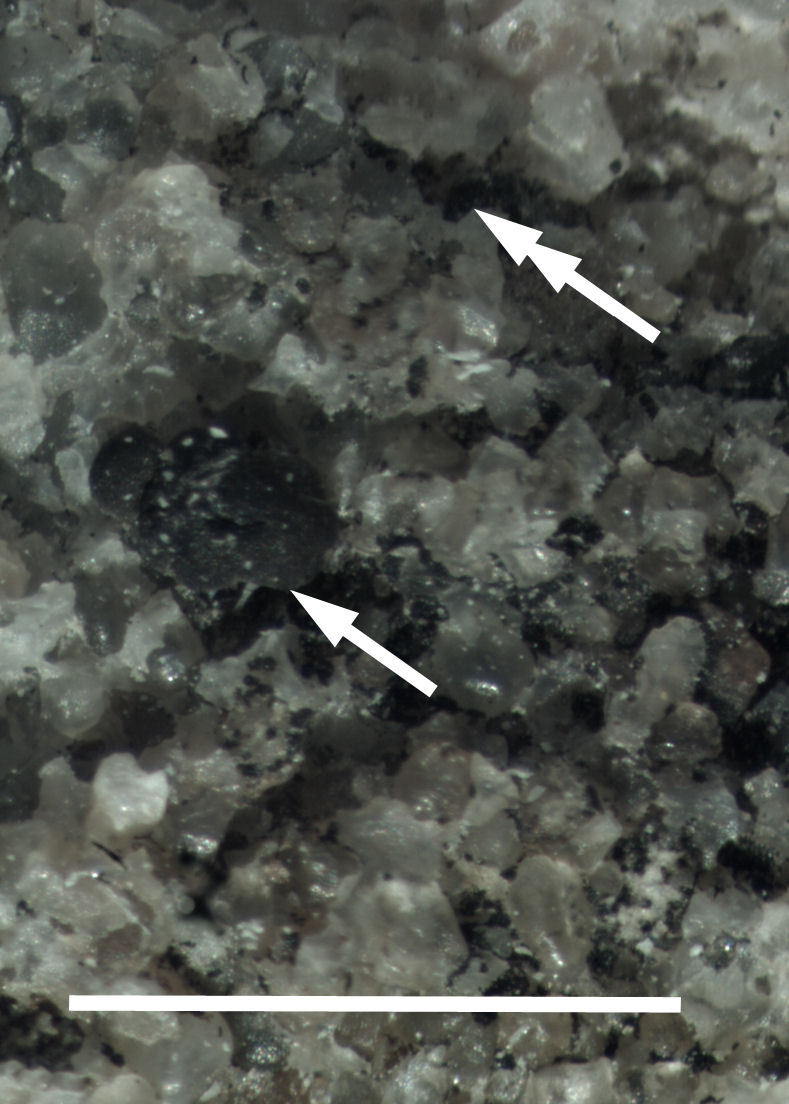
Partial unidentified lichen ball (single arrow). Fine residual hyphae, identity unknown (double arrow). Scale bar = 2.5 mm.

**Figure 7a.**
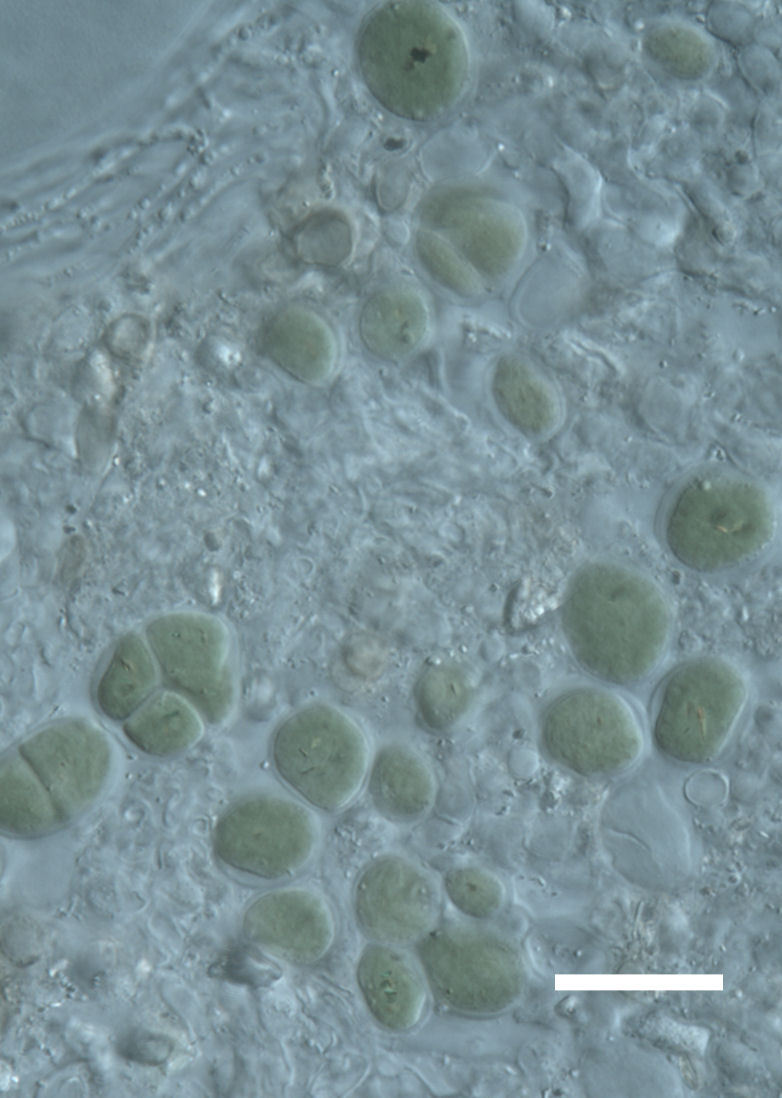
Cell squash highlighting *Trebouxia* sp. 2 with solid flattened chloroplast plates extending across the cell. Pyrenoid present in 6 cells. Cell wall <0.8 μm thick.

**Figure 7b.**
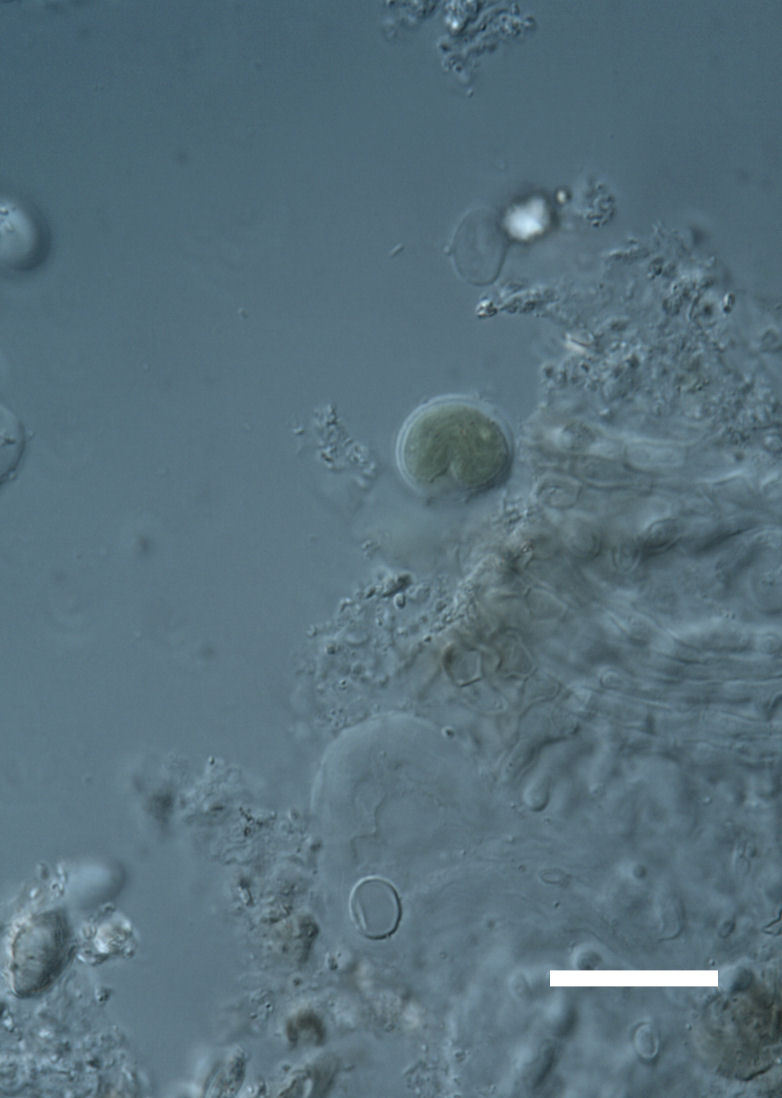
Chloroplast poorly discoid or a flattened plate. Pyrenoid present in a few cells. Cell wall up to 1 μm in thickness.

**Figure 7c.**
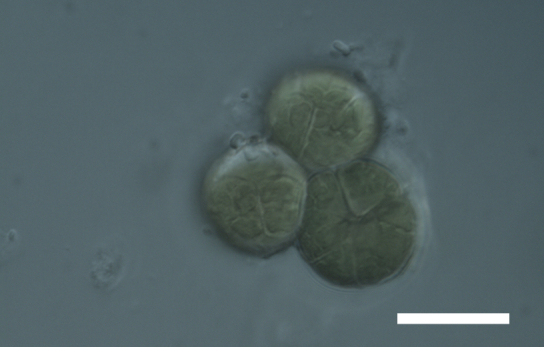
Cultured cells showing larger size, single cells and colony with pie-shaped cells.

**Figure 7d.**
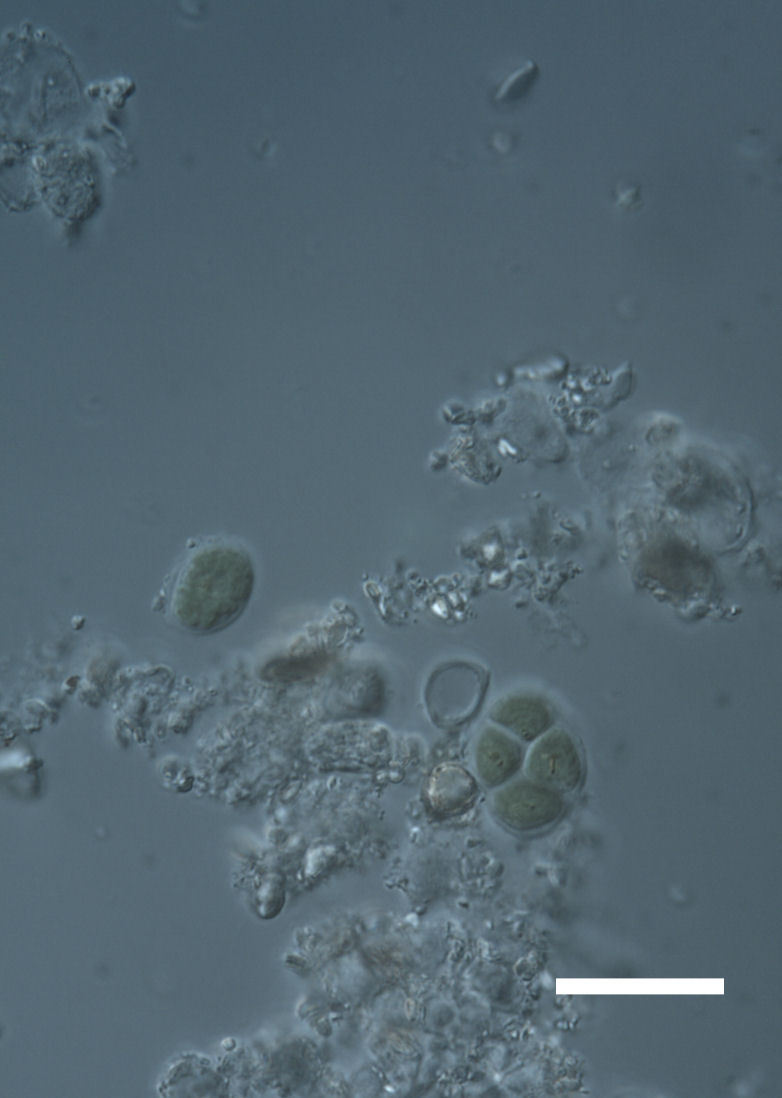
Colonial form within the lichen, cells with flattened plate-like chloroplast and thick cell walls.

**Figure 7e.**
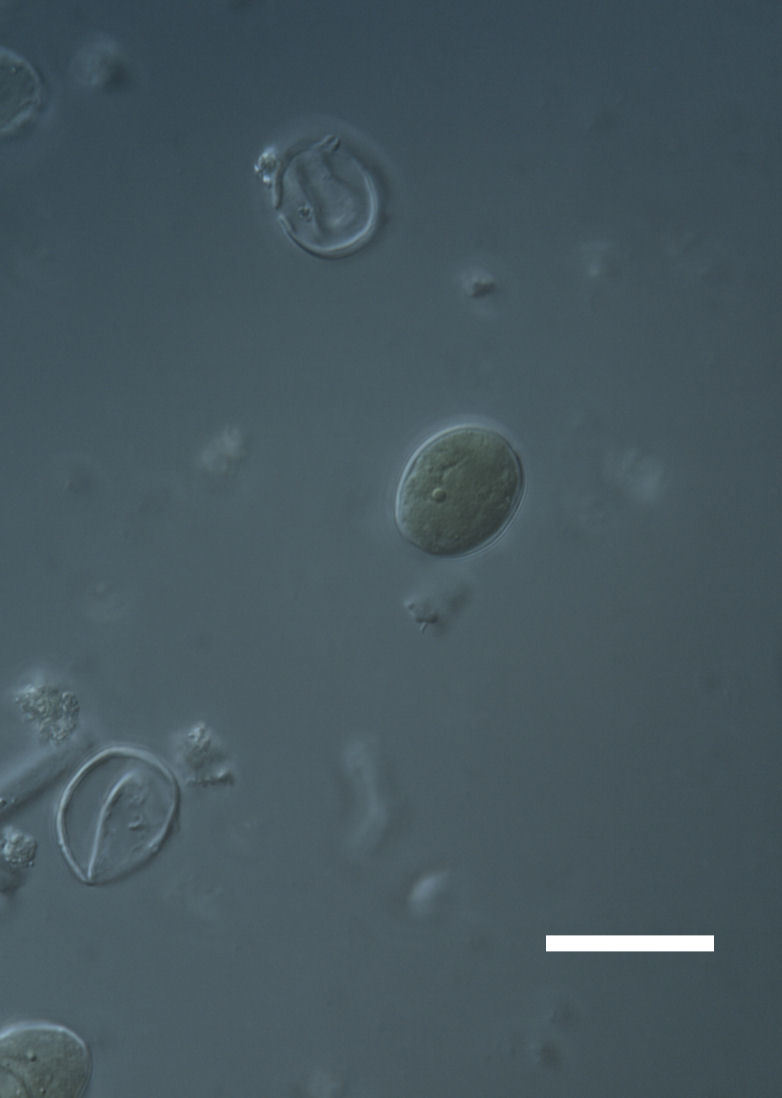
Squash showing single cells with a solid chloroplast and thin cell walls.

**Figure 7f.**
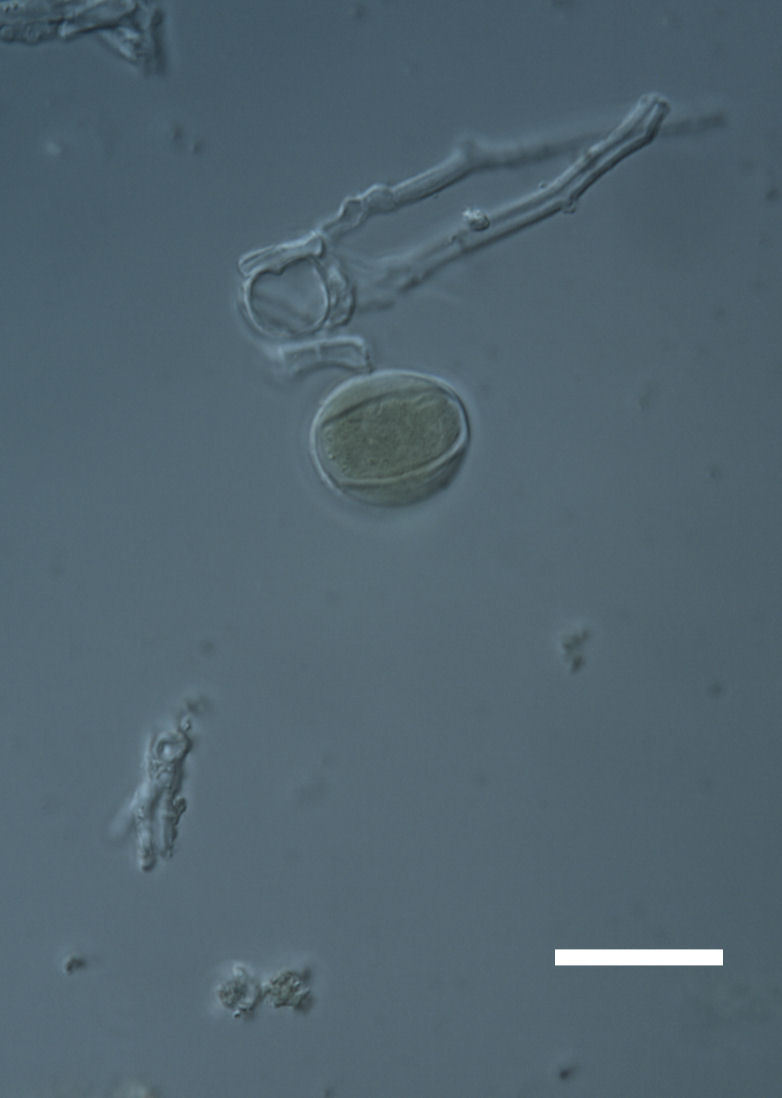
*Trebouxia* sp. 2 with hyphae of Lecanora cf. garovaglii.

**Figure 8a.**
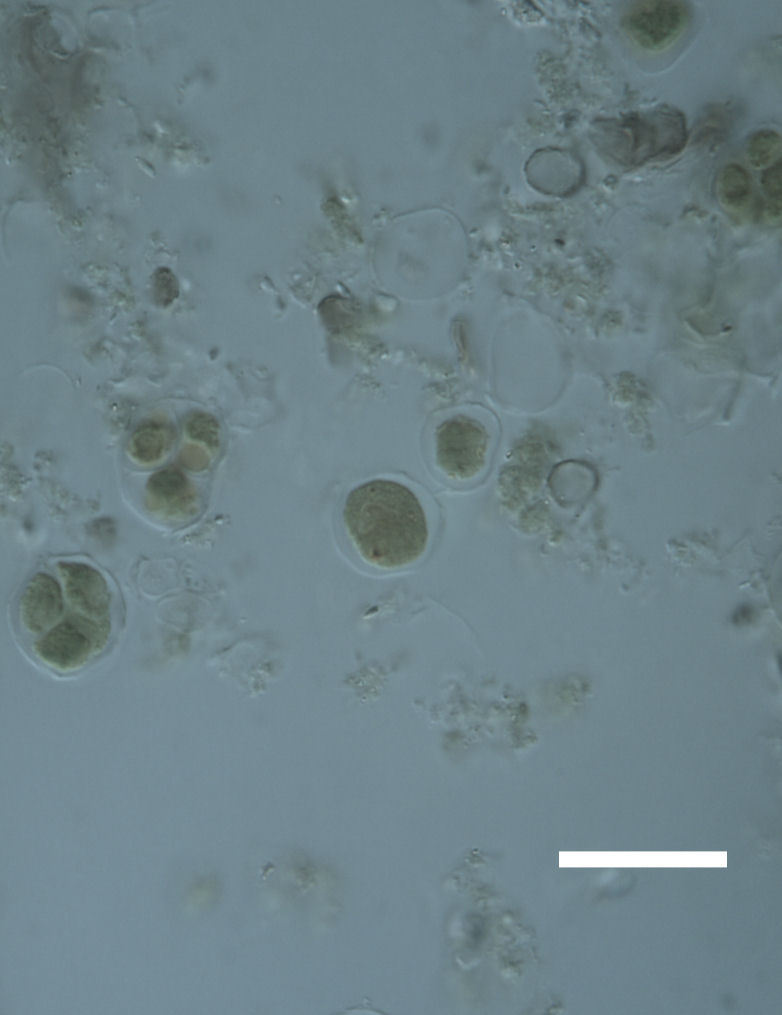
Squash showing single cells of small colonies.

**Figure 8b.**
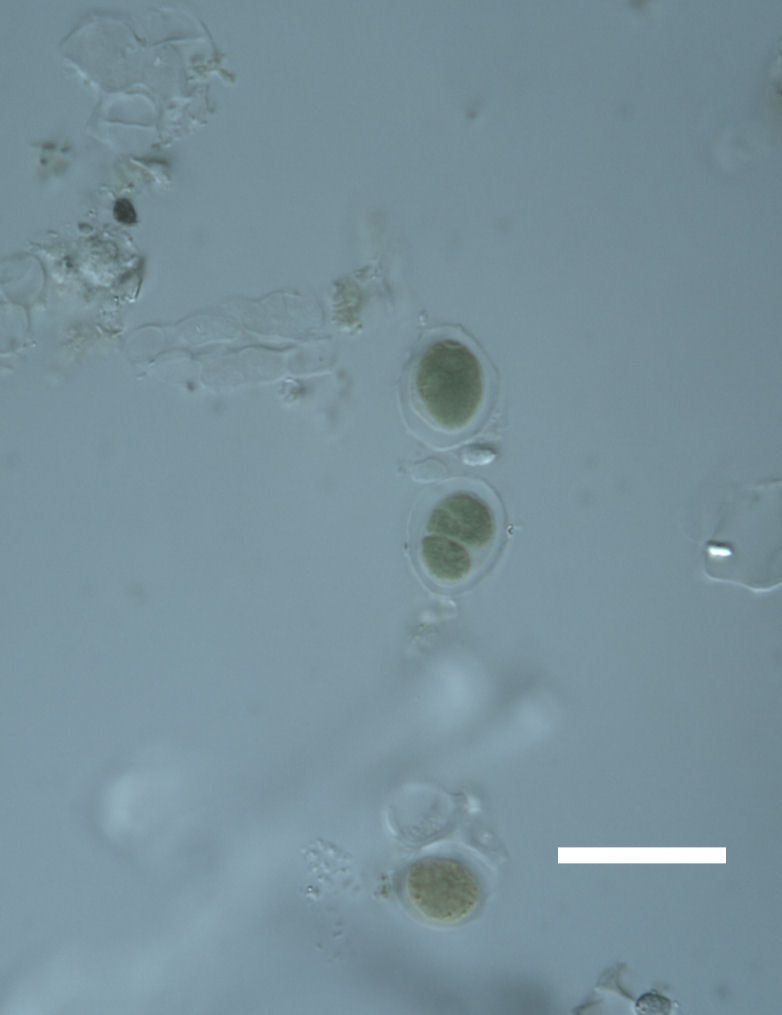
Squash showing single cells of small colonies.

**Figure 8c.**
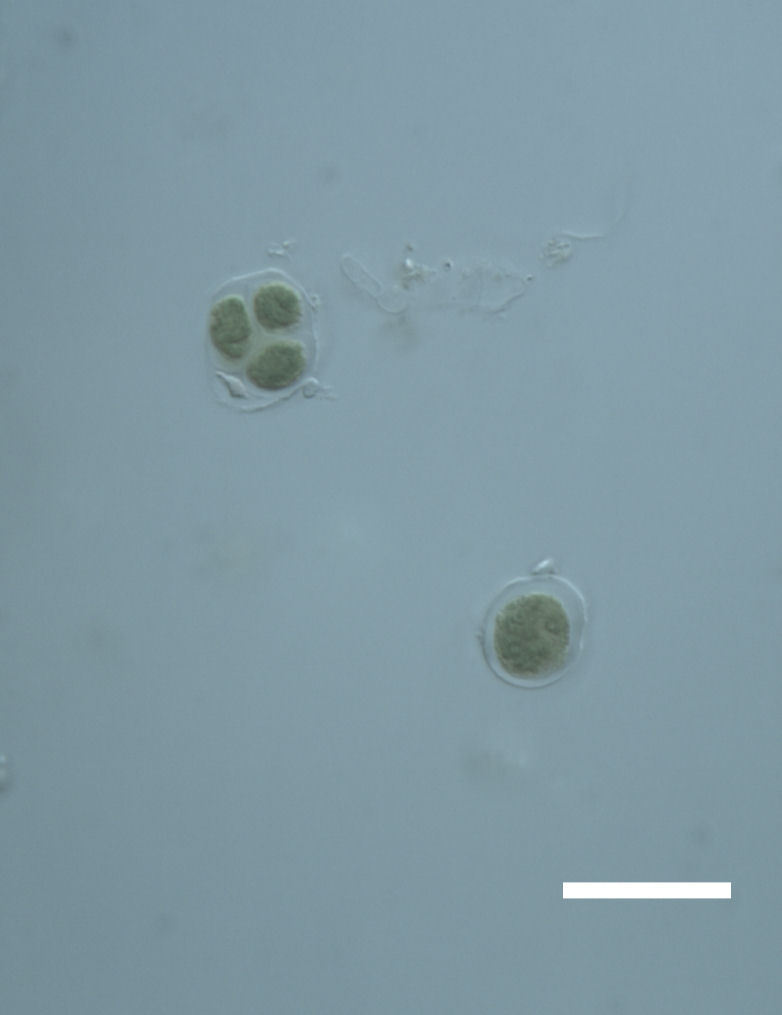
Cells with a broad flattened plate and very thick sheath (>1.5 μm).

**Figure 9a.**
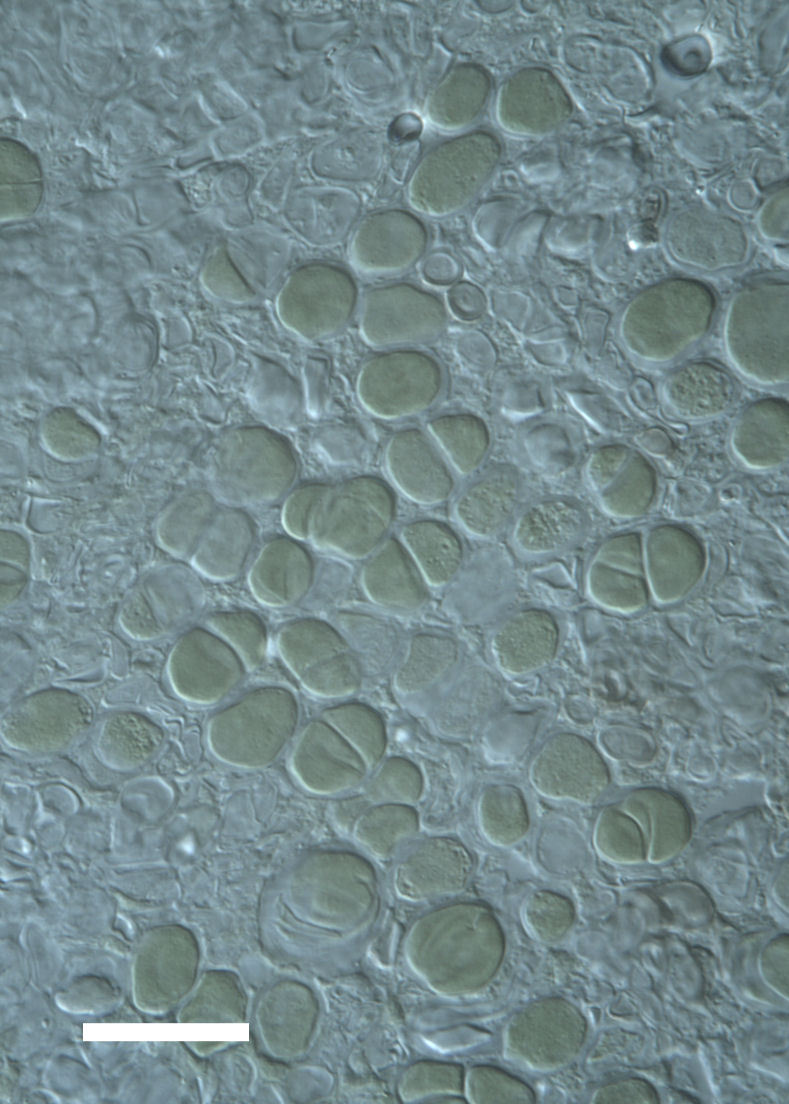
*Myrmecia* sp. in *Heteroplacidium compactum*.

**Figure 9b.**
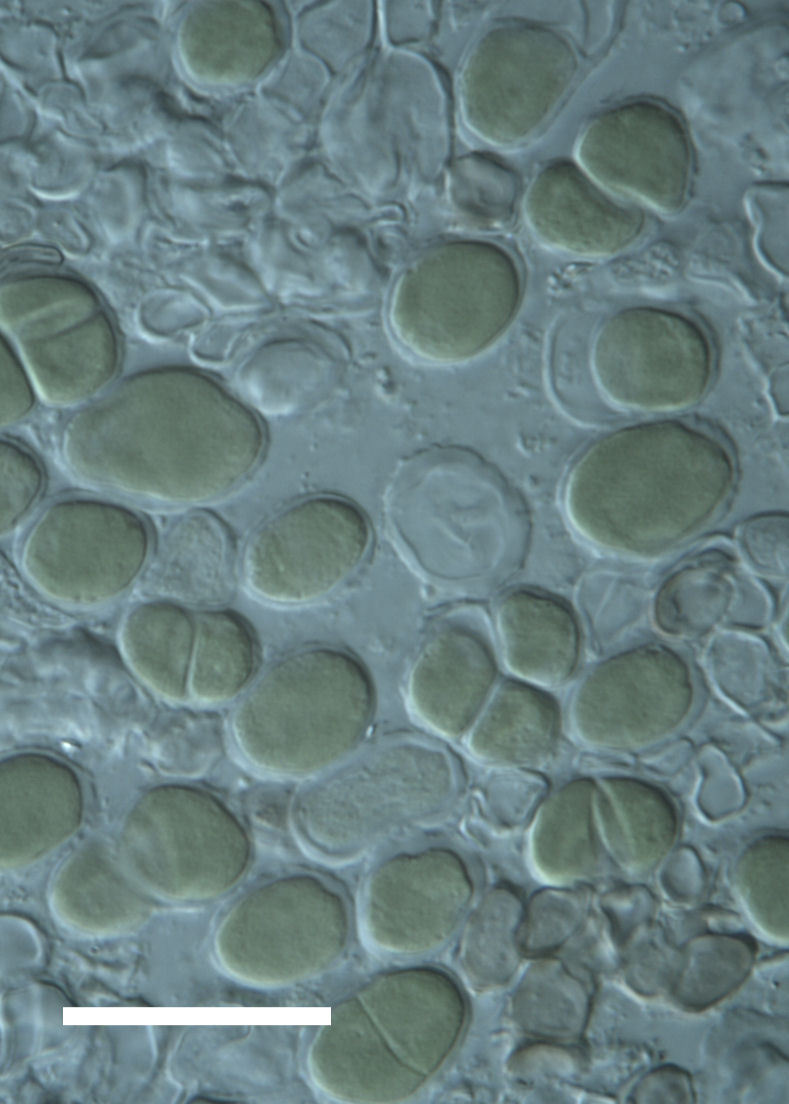
*Myrmecia* sp. in *Heteroplacidium compactum*.

**Figure 9c.**
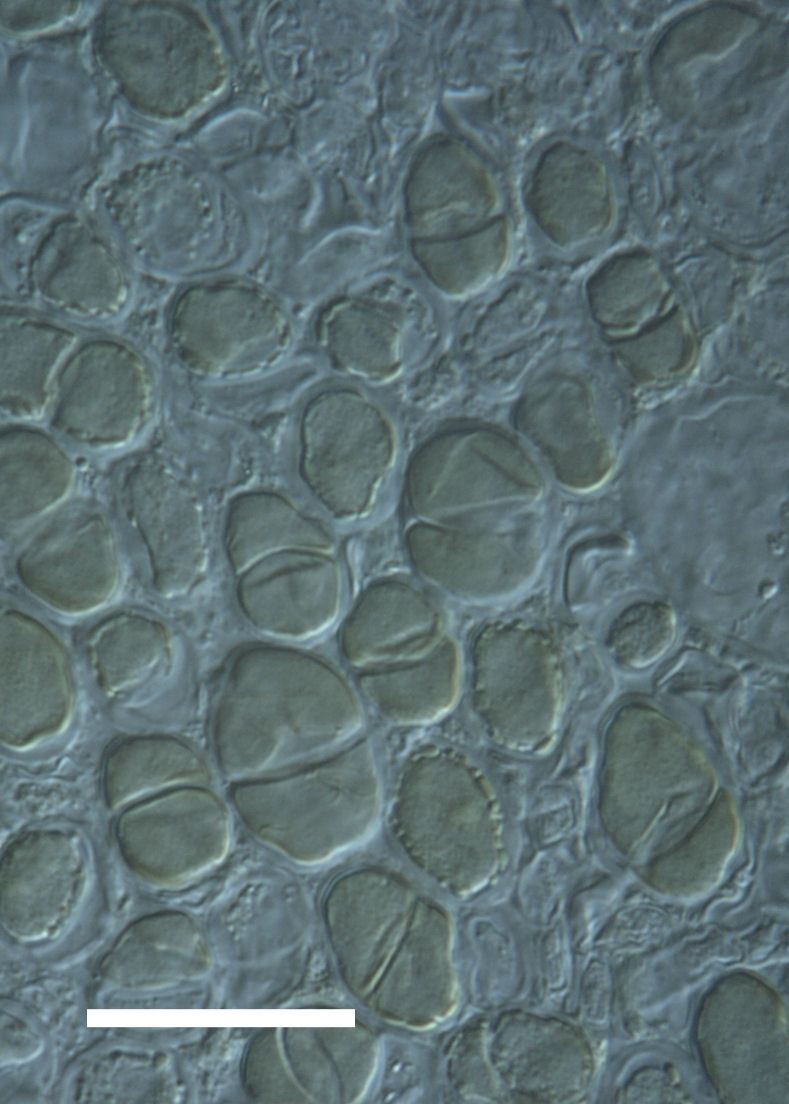
*Myrmecia* sp. in *Heteroplacidium compactum*.

**Figure 9d.**
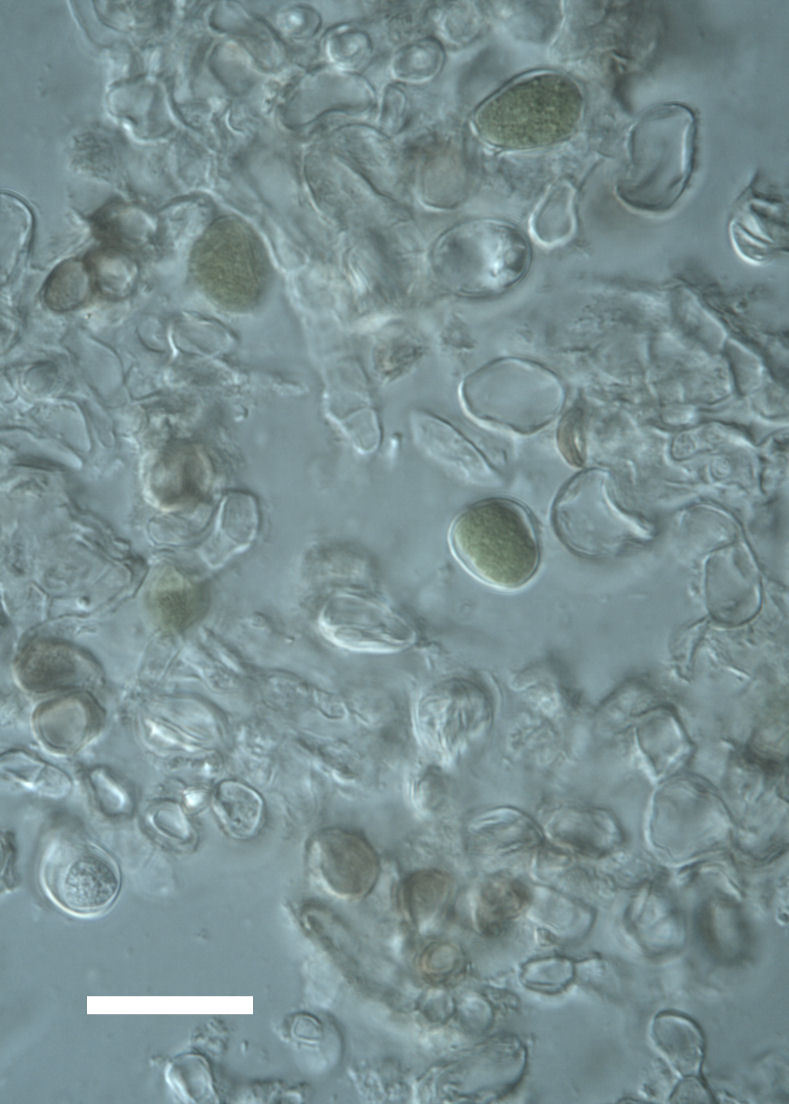
*Myrmecia* sp. in *Placidium acarosporoides*.

**Figure 9e.**
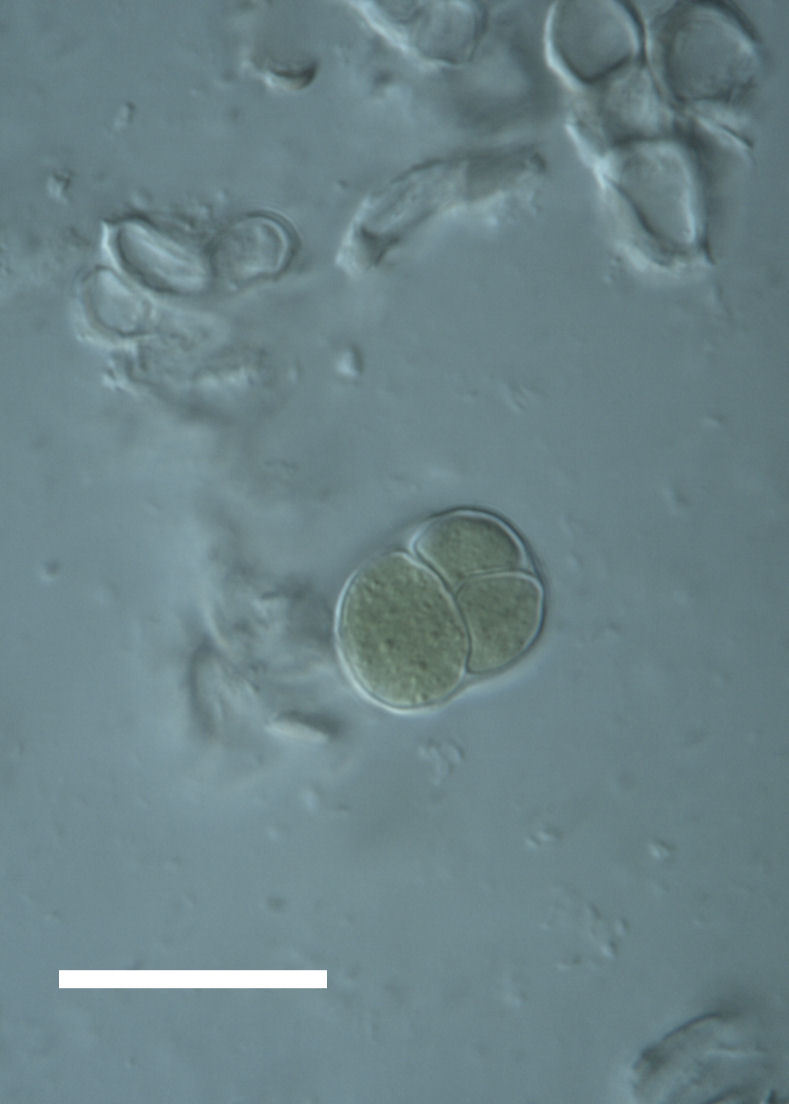
*Myrmecia* sp. in *Placidium acarosporoides*.

**Figure 9f.**
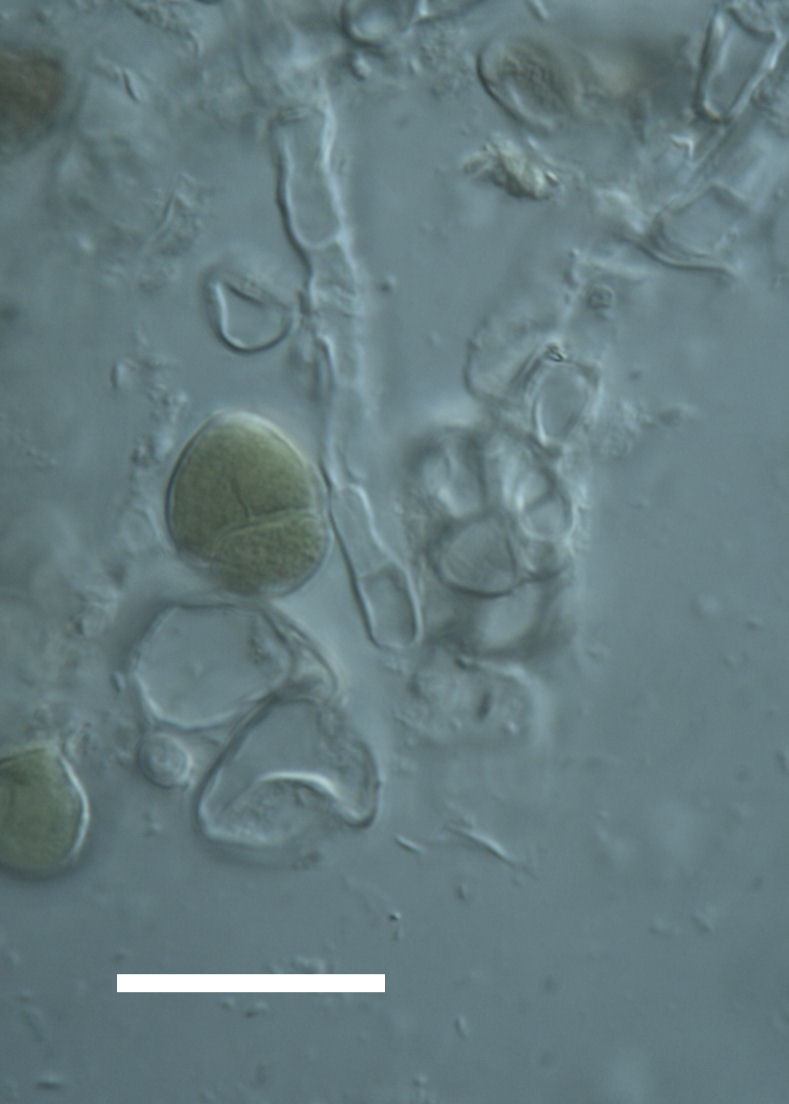
*Myrmecia* sp. in *Placidium acarosporoides*.

**Figure 13a.**
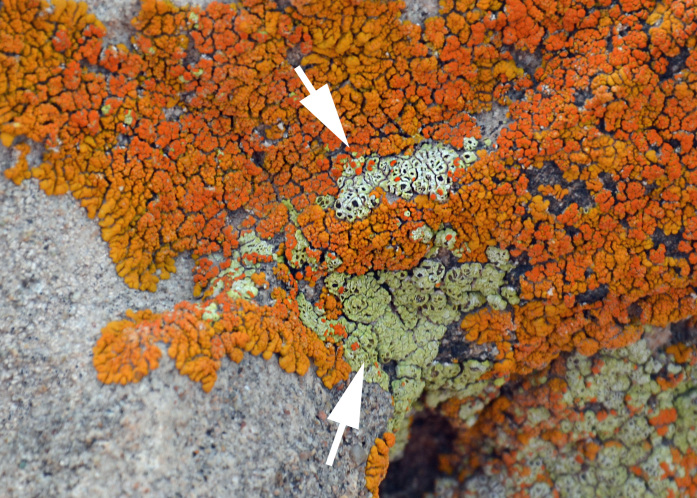
Habit, growing on sandstone and parasitic on *Caloplaca trachyphylla* (orange lichen). Vicinity of Mars Desert Research Station, November 20, 2014.

**Figure 13b.**
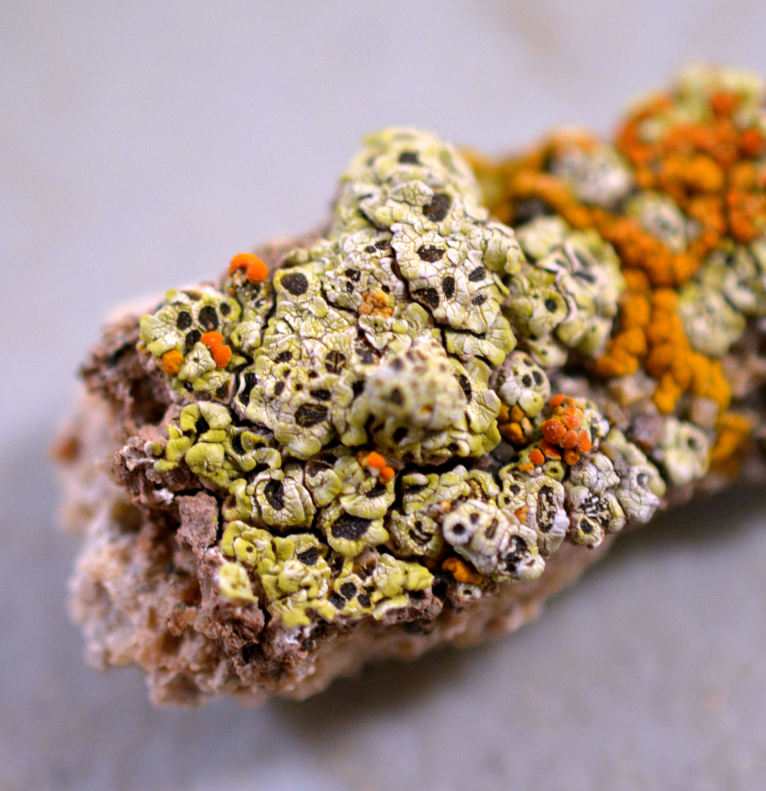
Thallus detail (*Sokoloff 270*).

**Figure 14a.**
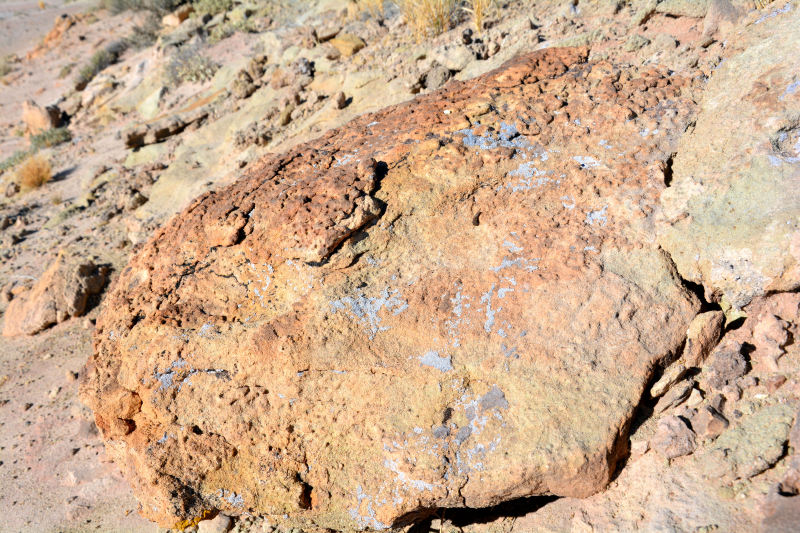
Habitat on sandstone.

**Figure 14b.**
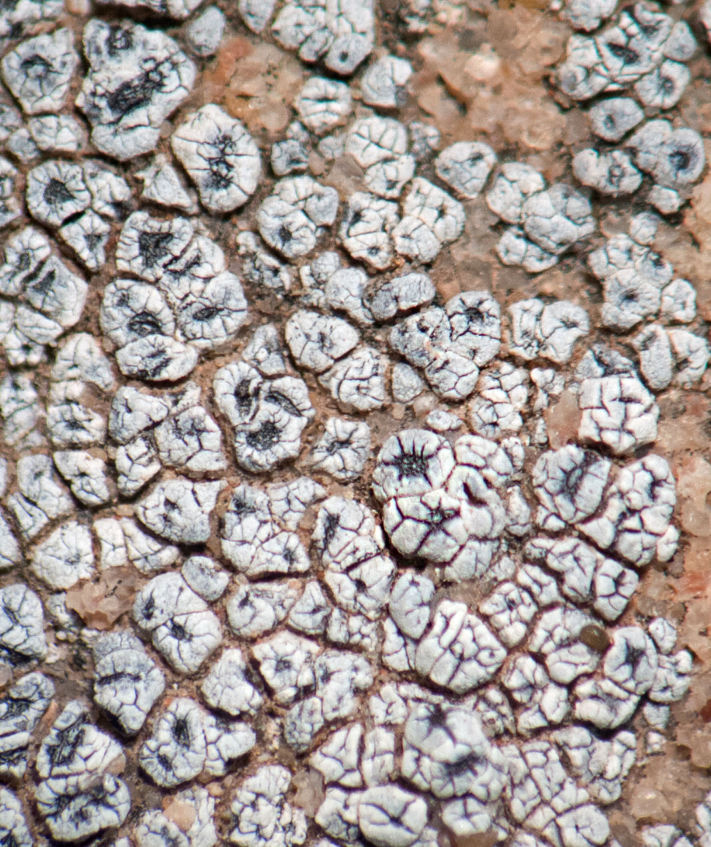
Habit.

**Figure 19a.**
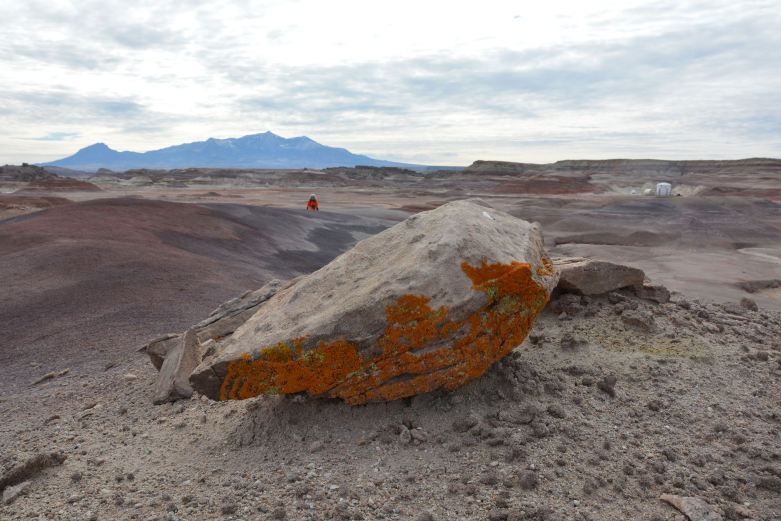
Habitat, rock-strewn hill northeast of the Mars Desert Research Station (visible at right), November 20, 2014.

**Figure 19b.**
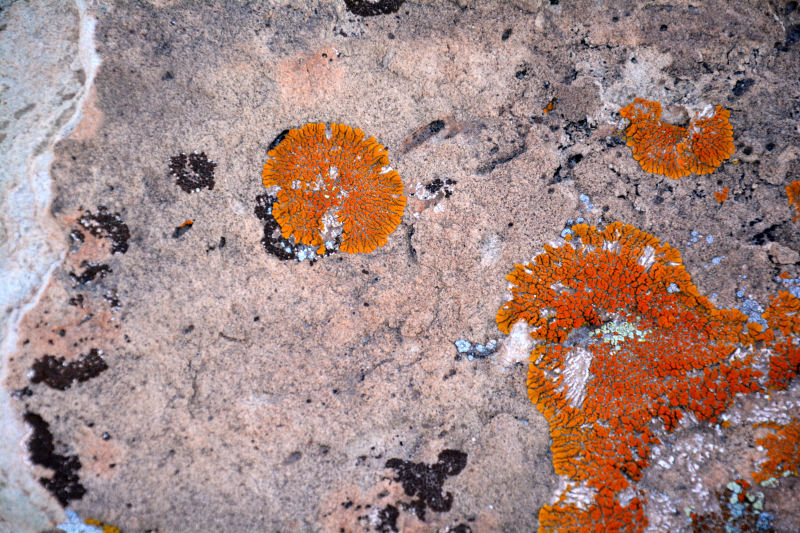
Habit (*Sokoloff 252*).

**Figure 21a.**
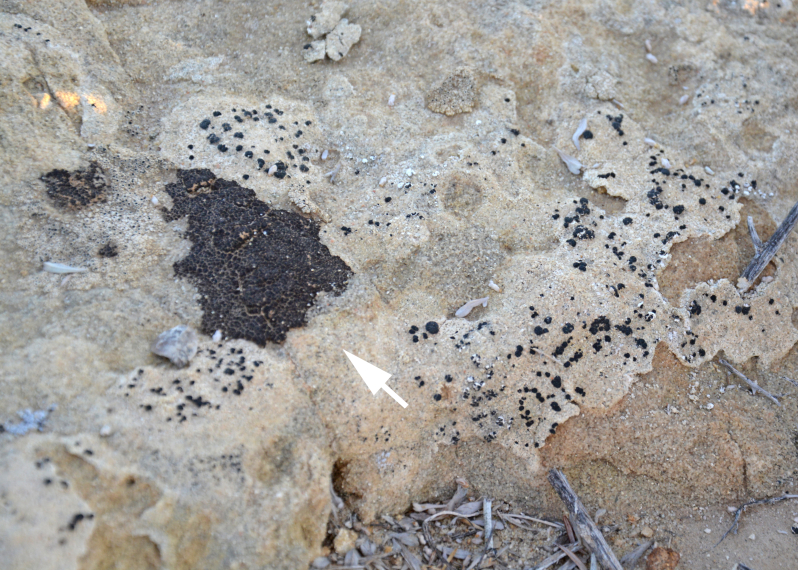
Habit. Vicinity of Mars Desert Research Station, Utah, November 29, 2014.

**Figure 21b.**
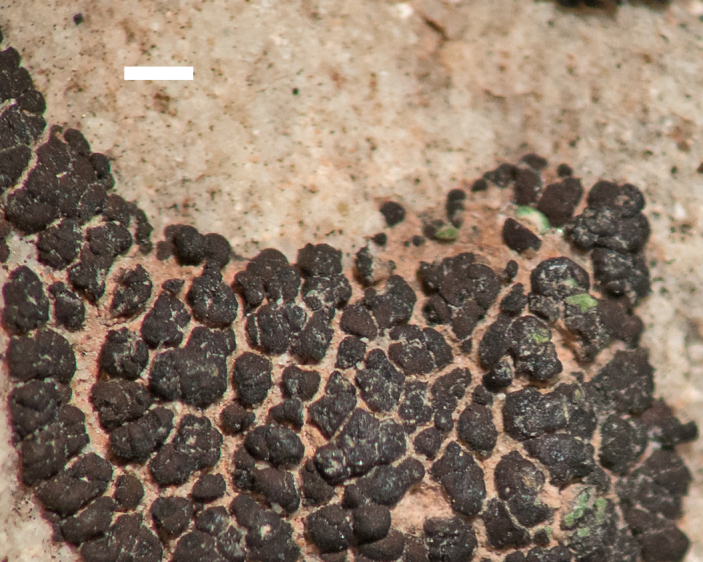
Convex squamules forming an areolate-looking thallus (*Sokoloff 305*). Scale bar = 1 mm.

**Figure 22a.**
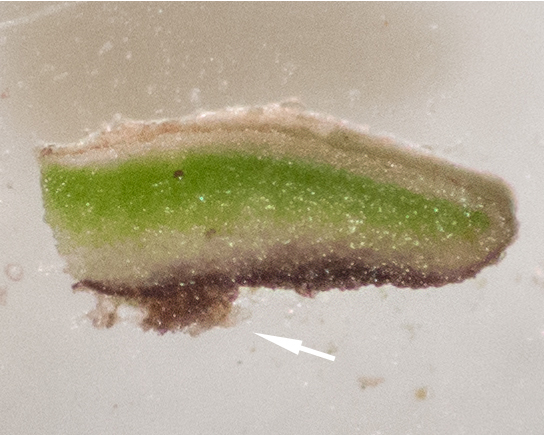
Squamule section showing distinct lower cortex and hyphal weft (arrow).

**Figure 22b.**
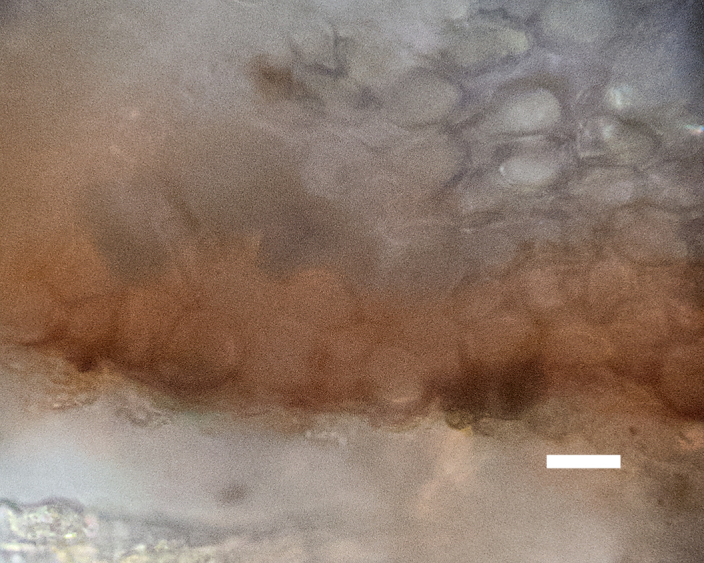
Lower cortex cells in +/- vertical alignment. Scale bar = 10 µm.

**Figure 22c.**
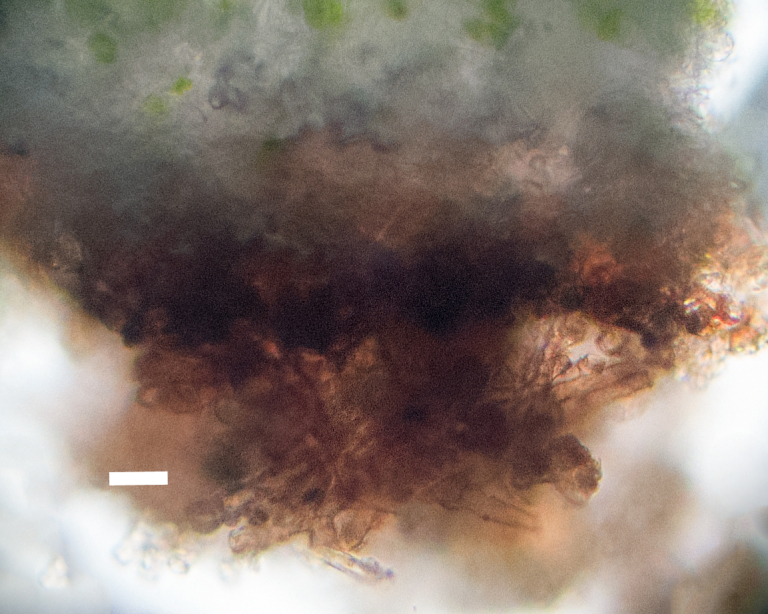
Hyphal weft incorporating soil granules. Scale bar = 20 µm.

**Figure 23a.**
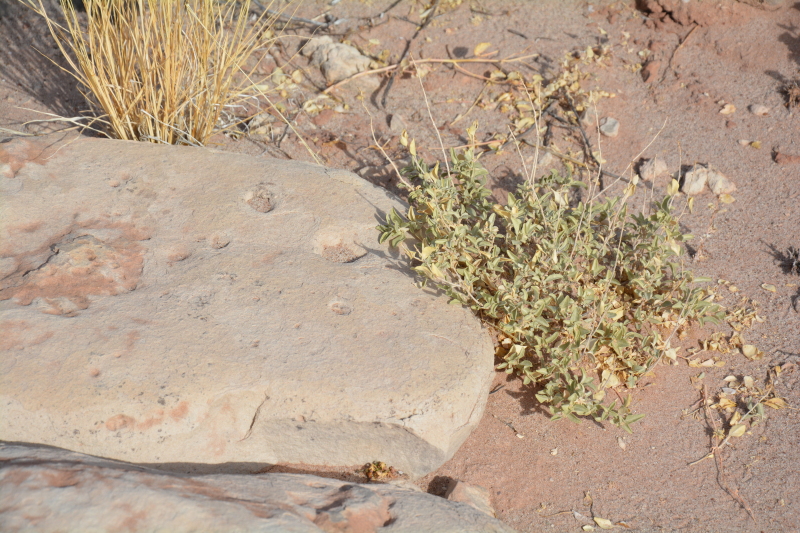
Habit (*Sokoloff 266*).

**Figure 23b.**
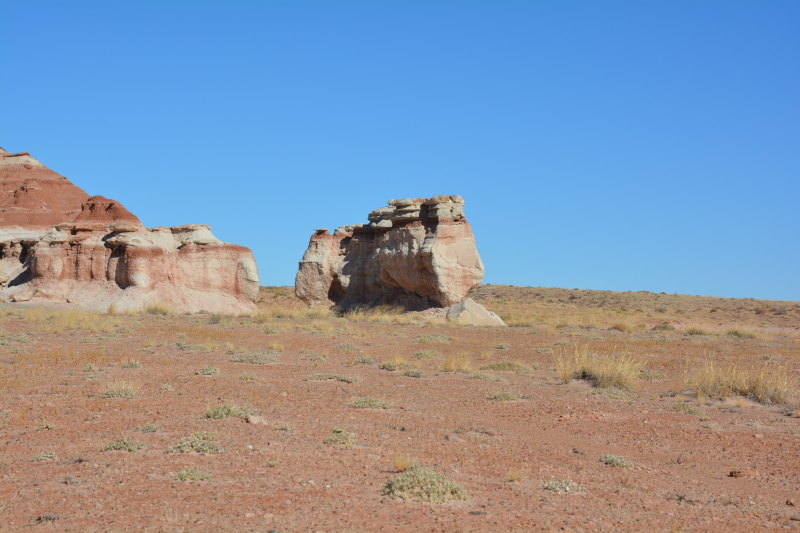
Desert flats dominated by Atriplex gardneri var. cuneata and grasses. Northeast of Mars Desert Research Station, Utah, November 23, 2014.

**Figure 24a.**
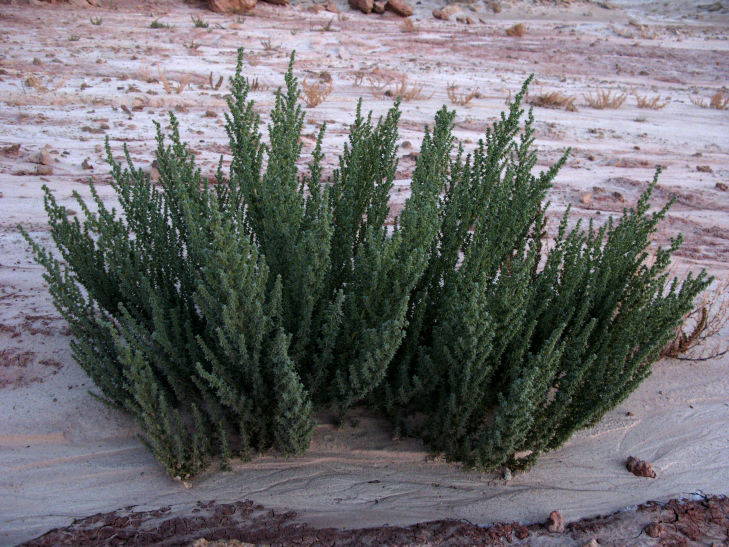
Habit. Vicinity of Mars Desert Research Station, Utah, September 20, 2015.

**Figure 24b.**
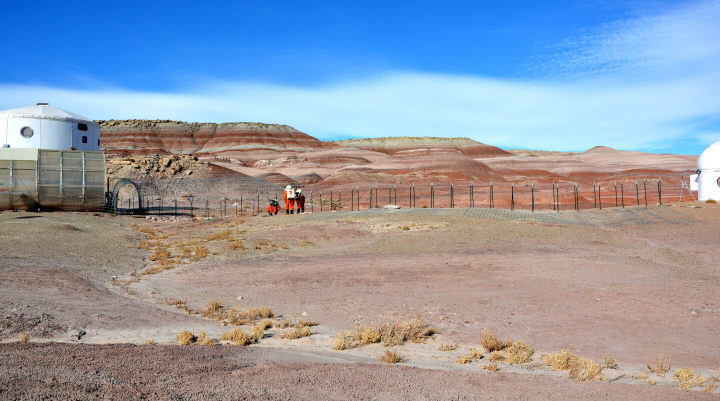
*Halogeton glomeratus* and Atriplex gardneri var. cuneata habitat (foreground) immediately east of the Mars Desert Research Station, Utah, November 26, 2014.

**Figure 25a.**
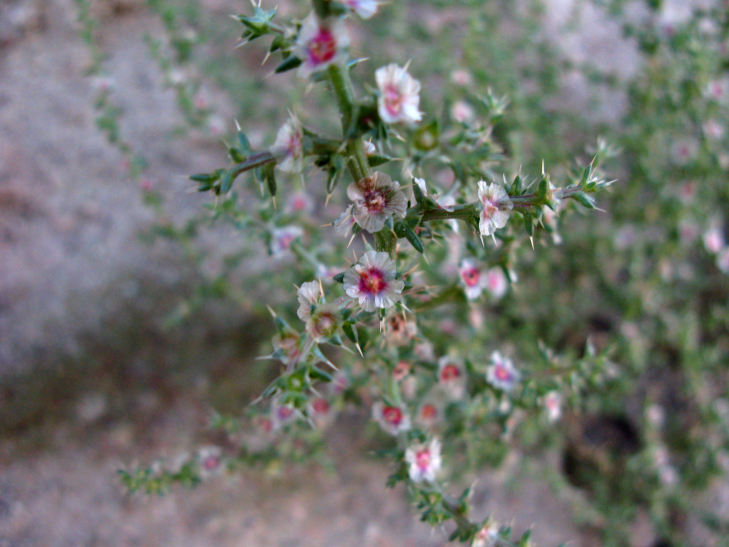
Inflorescence.

**Figure 25b.**
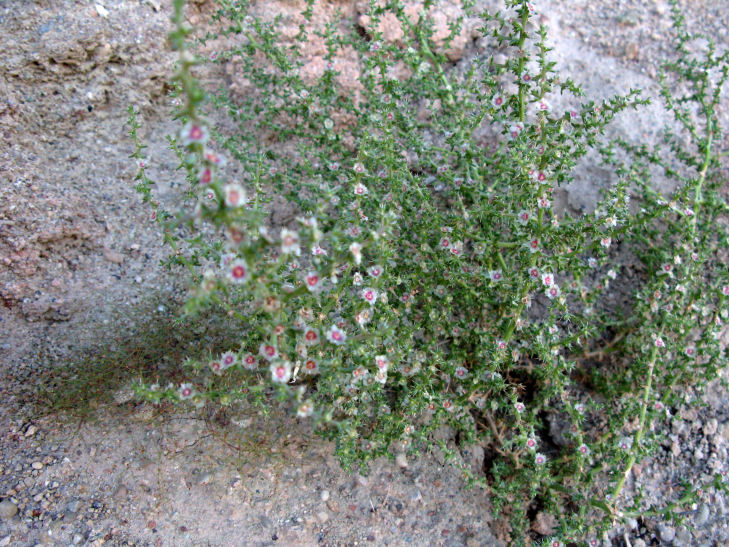
Habit.

**Figure 27a.**
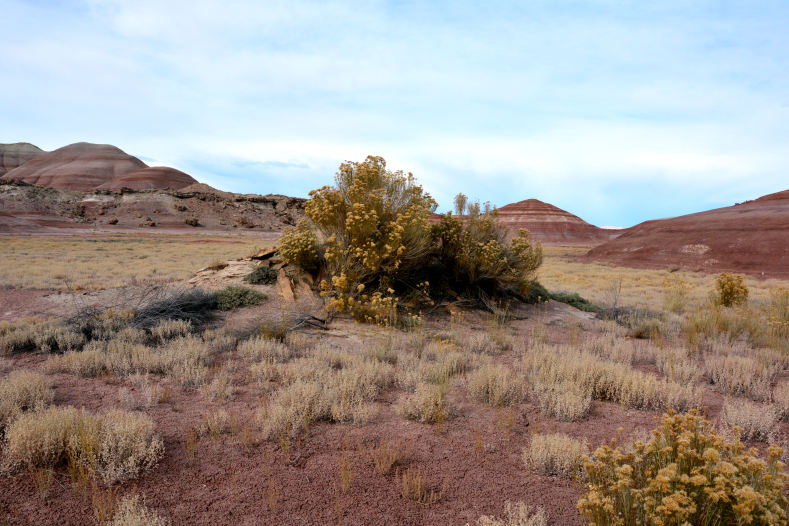
Desert lowland habitat dominated by *Ericameria nauseosa* (large yellow shrub in centre and bottom-right of photo), *Atriplex* sp., *Achnatherum hymenoides*, and *Halogeton glomeratus*.

**Figure 27b.**
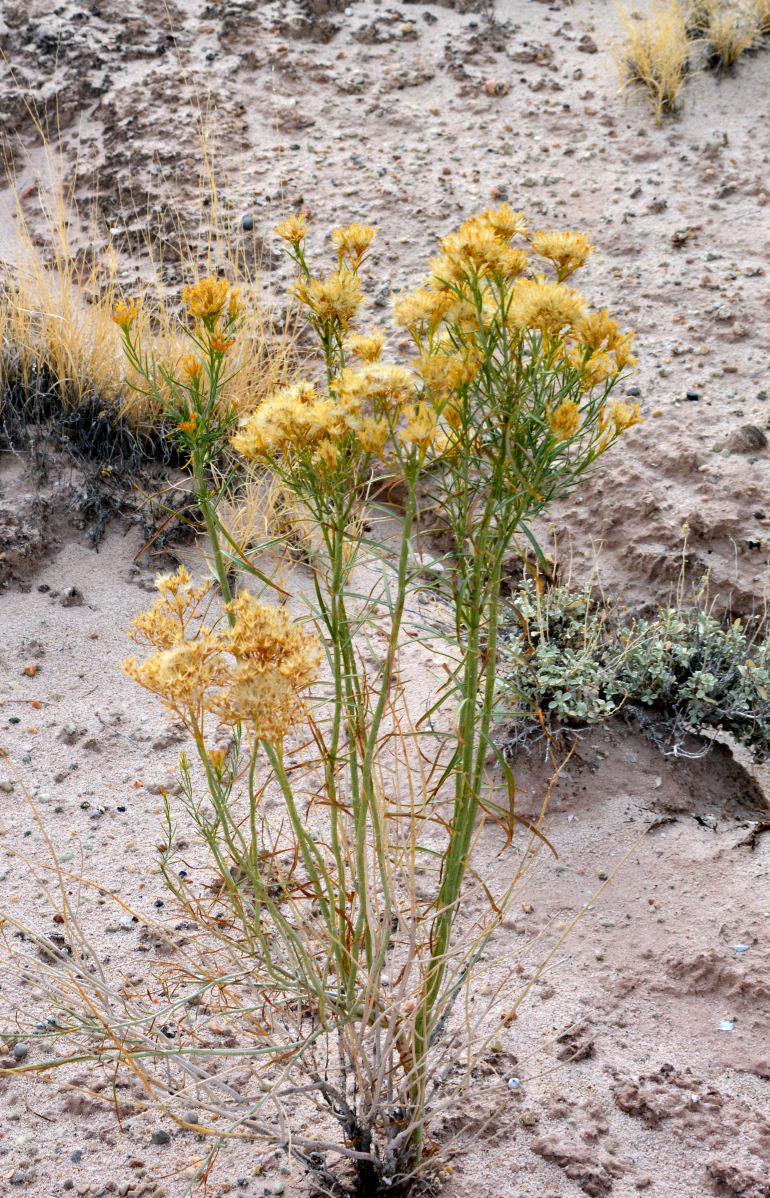
Habit.

**Figure 29a.**
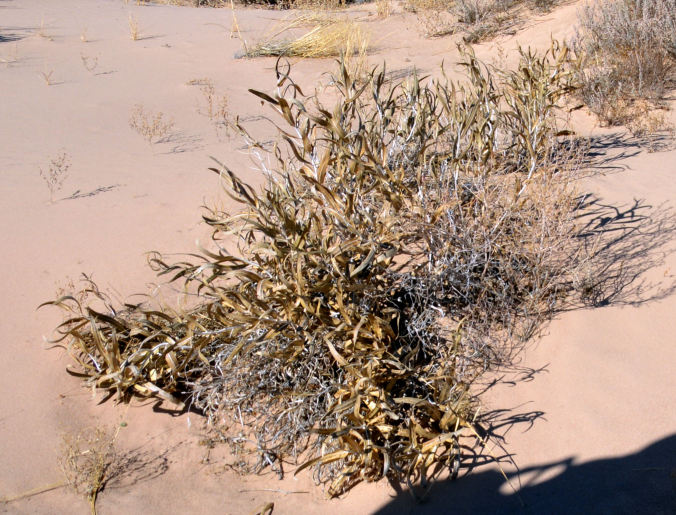
Habit.

**Figure 29b.**
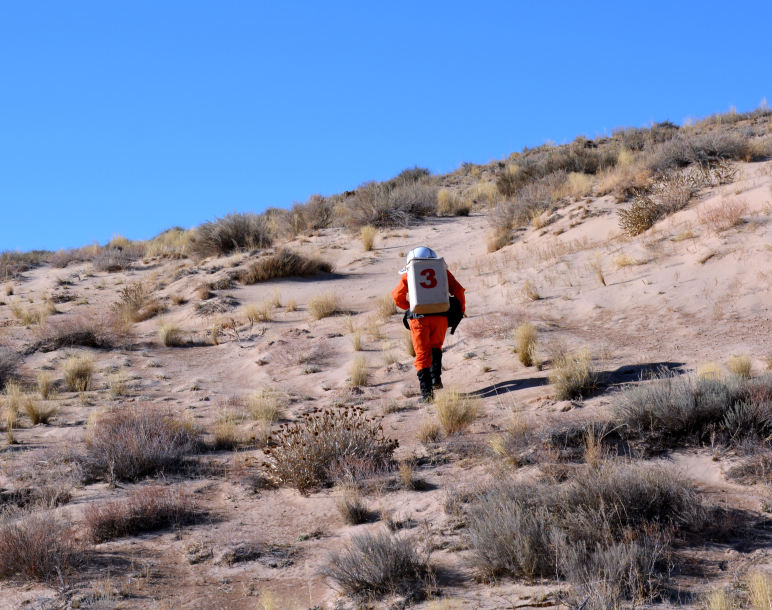
Habitat.

**Figure 31a.**
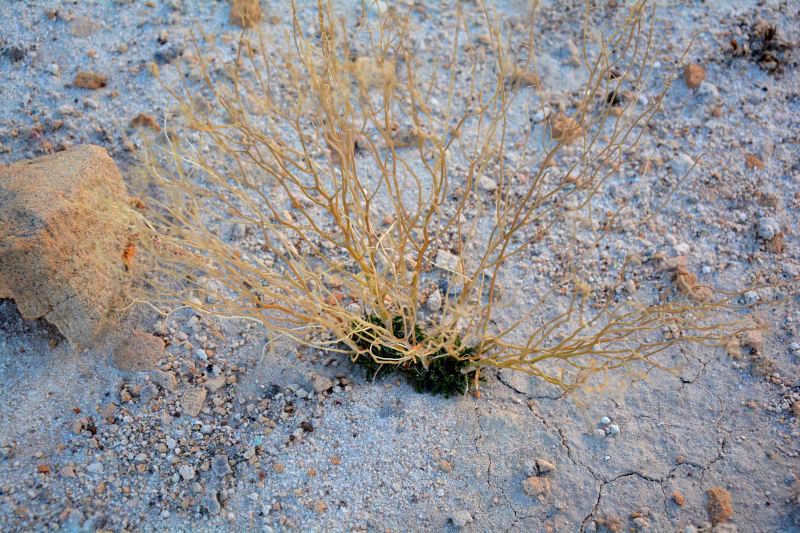
Habit (*Sokoloff 261*).

**Figure 31b.**
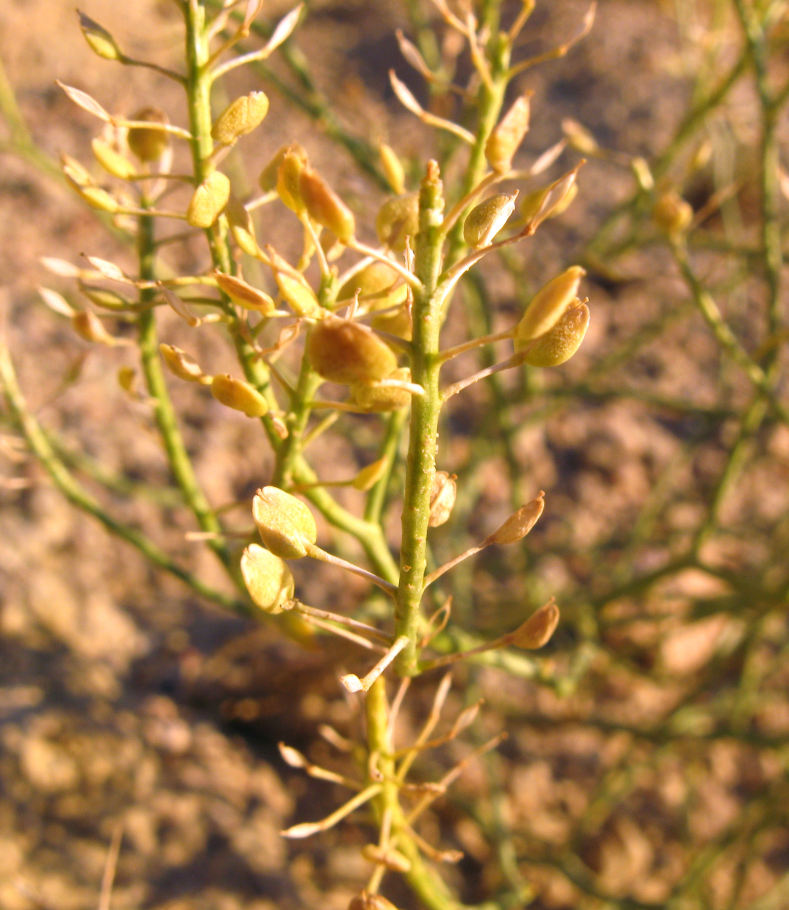
Fruits. Vicinity of Mars Desert Research Station, Utah, September 20, 2015

**Figure 31c.**
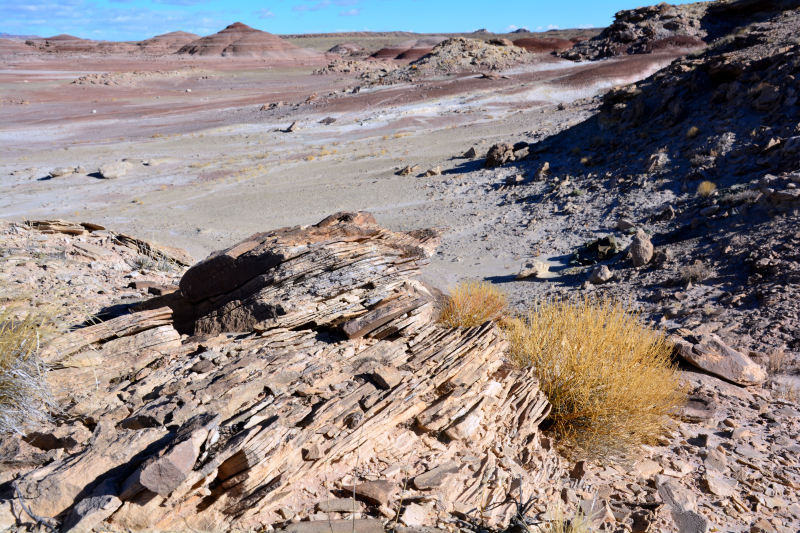
Habitat. Vicinity of Cow Dung Road, Utah, November 24, 2014.

**Figure 37a.**
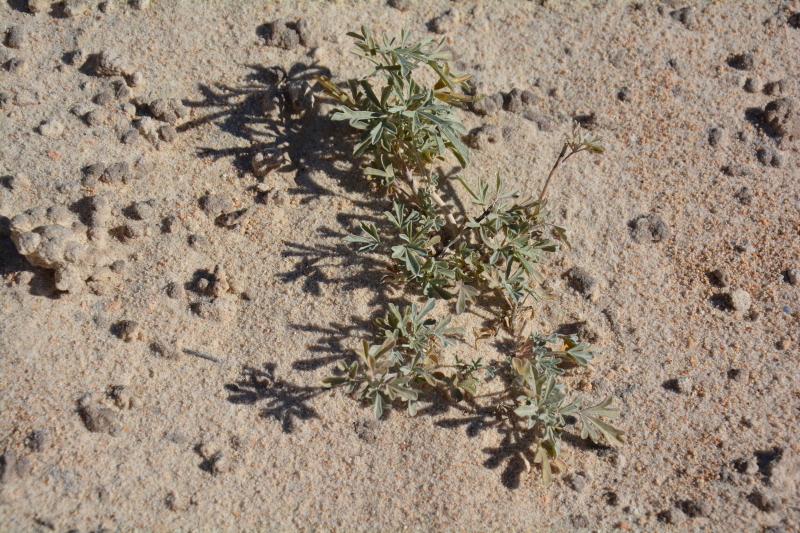
Habit.

**Figure 37b.**
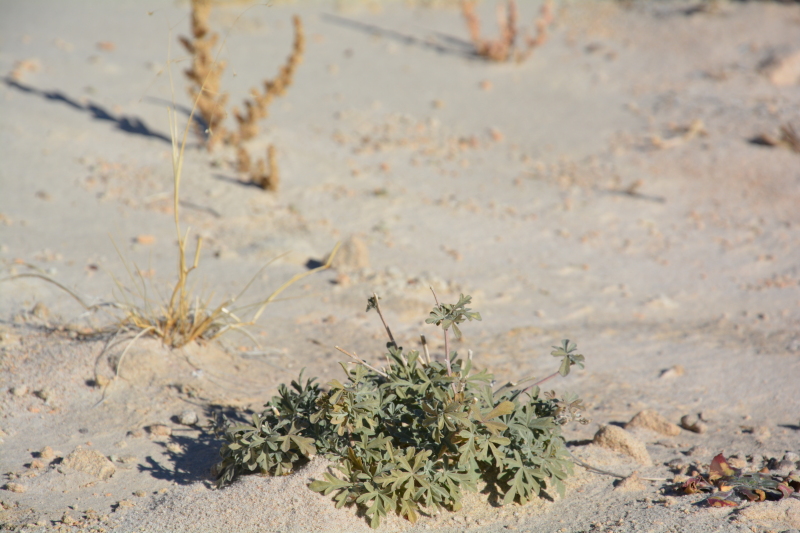
Habitat.

**Figure 39a.**
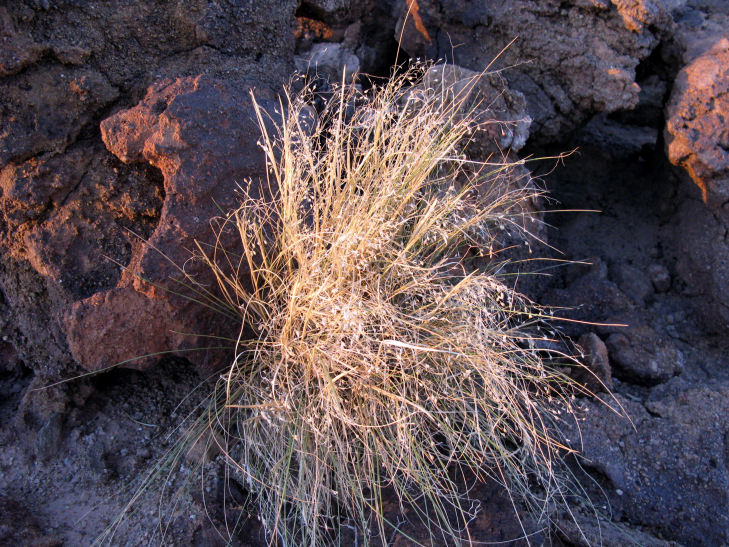
Habit.

**Figure 39b.**
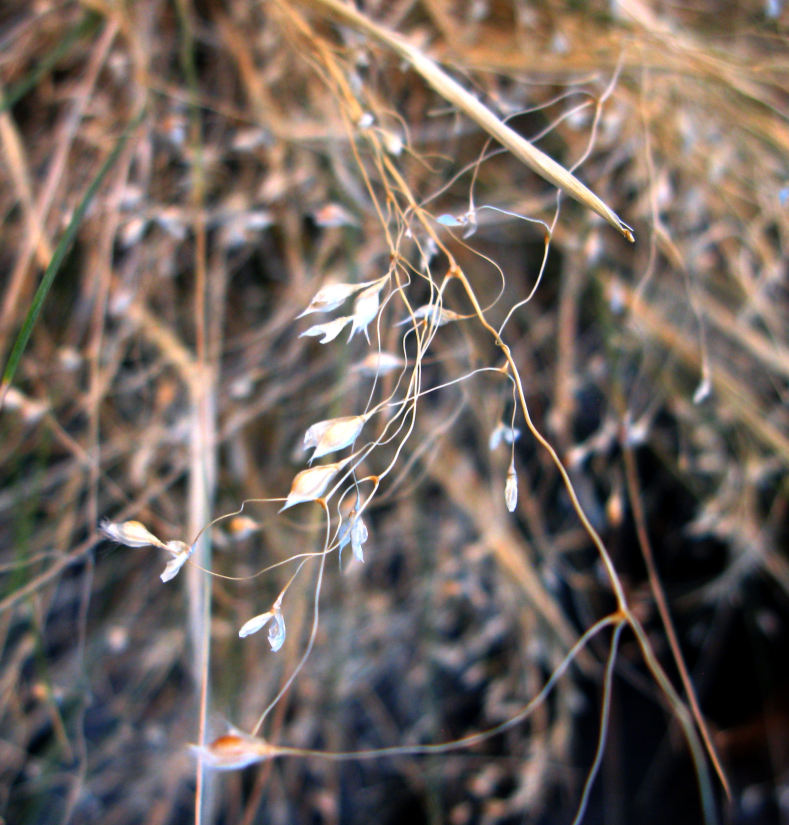
Inflorescence.

**Figure 39c.**
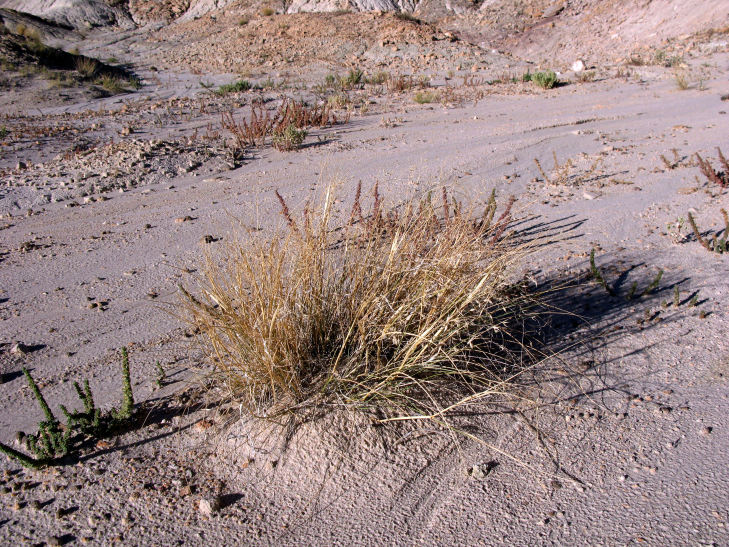
Habitat.

**Figure 41a.**
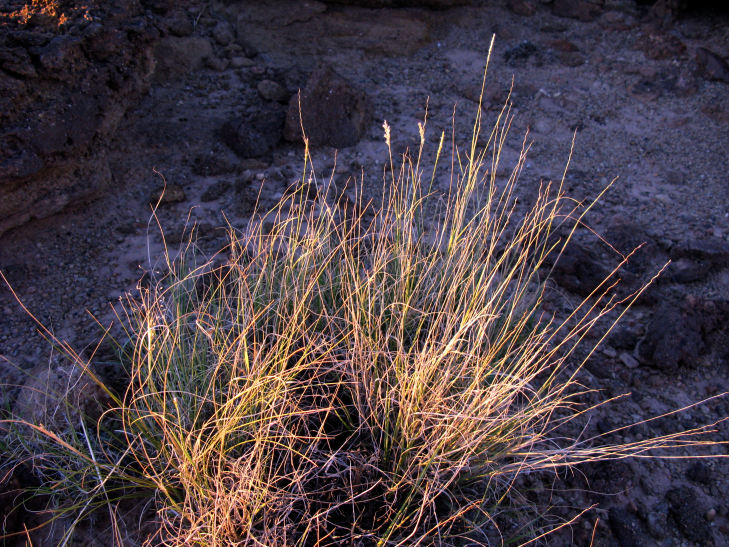
Habit.

**Figure 41b.**
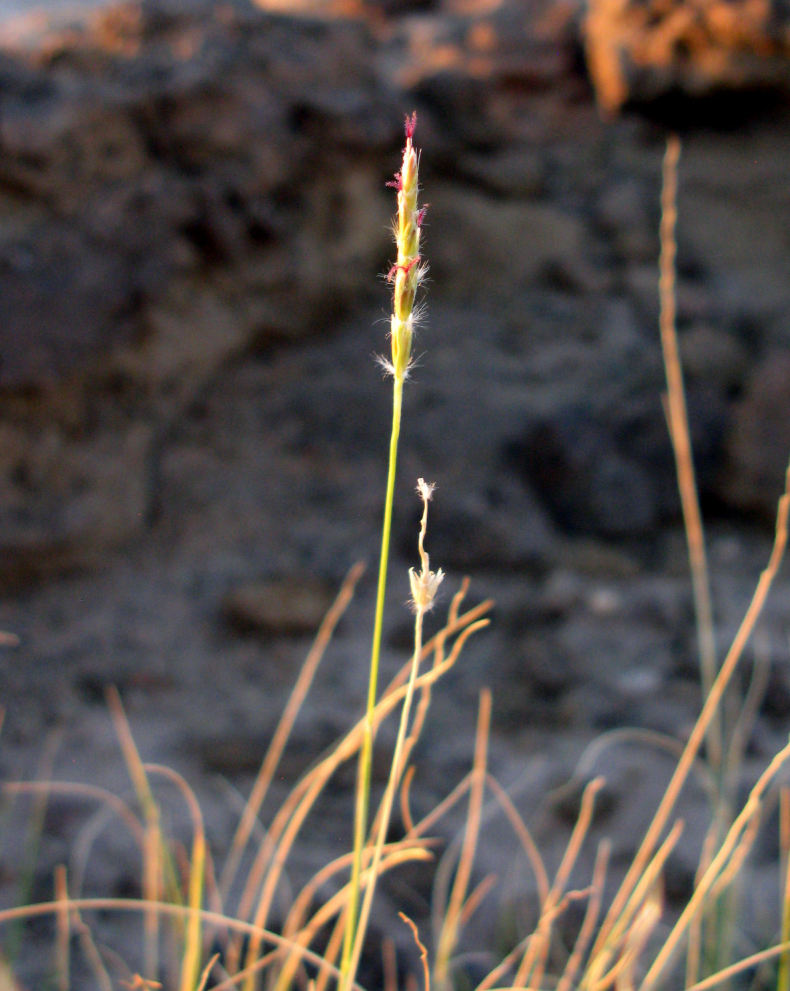
Inflorescence.

**Figure 43a.**
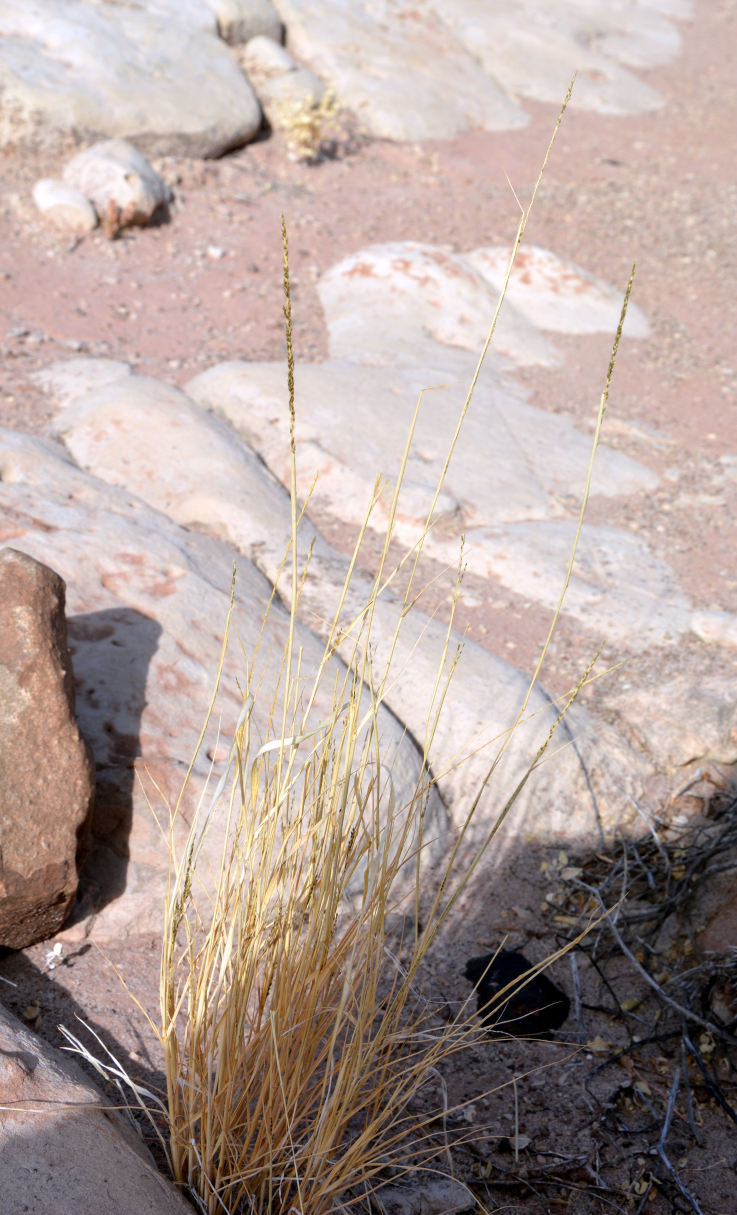
Habit.

**Figure 43b.**
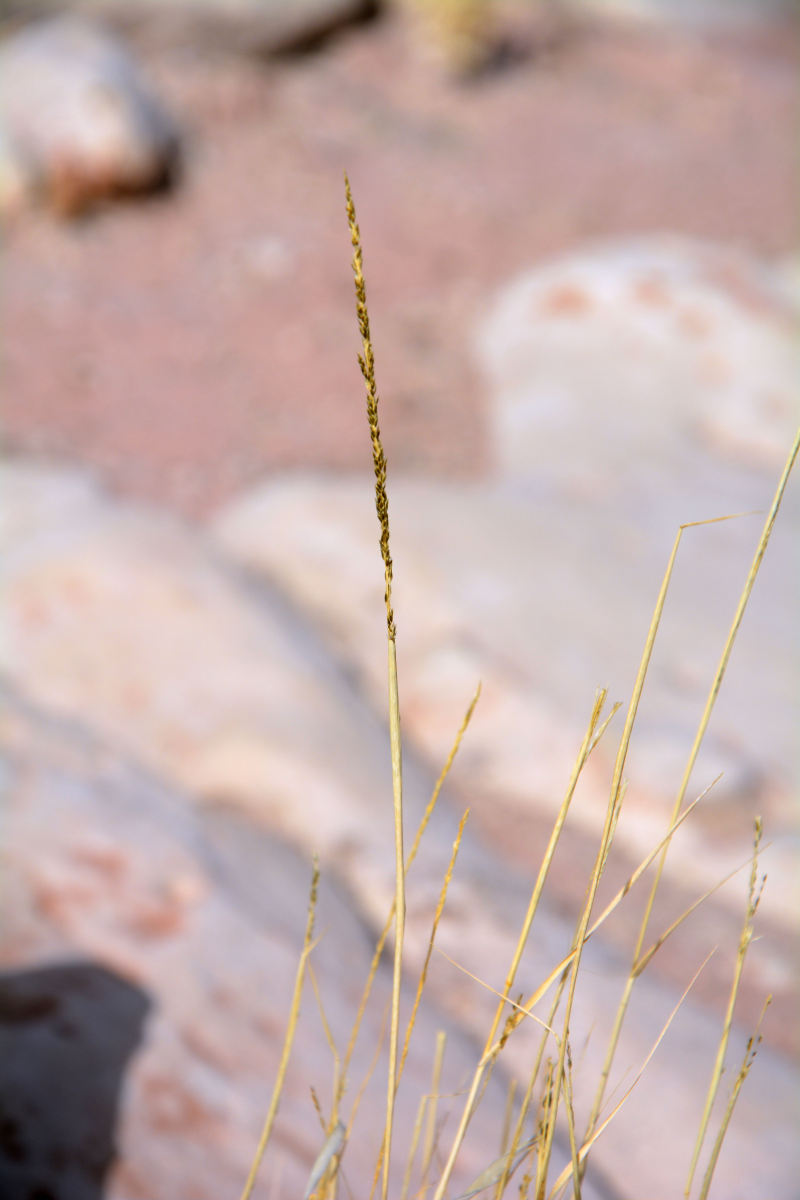
Inflorescence.

**Table 1.** Summary of terrestrial algae (chlorophyta), cyanobacteria, fungi, lichens, and vascular plant species collected at the Mars Desert research Station (MDRS) for the current study. For the vascular plant taxa, presence at one of three nearby, well collected sites (the San Rafael Swell, Capitol Reef National Park, and Glen Canyon Recreational Area) is noted with an x. x^1^: expected to occur within Glen Canyon Recreational Area, but no voucher was located during the study.

**Higher taxon**	**Family**	**Species recorded for the Mars Desert Research Station (MDRS) study area**	**Present in the San Rafael Swell (Harris 1983)**	**Present at Capitol Reef National Park (Fertig 2009)**	**Present at Glen Canyon Recreational Area (Hill et al. 2009)**
Chlorophyta	Trebouxiaceae	*Trebouxia* sp. 1	-	-	-
		*Trebouxia* sp. 2	-	-	-
		*Trebouxia* sp. 3	-	-	-
		*Trebouxia* sp. 4	-	-	-
		*Myrmecia* sp.	-	-	-
Cyanobacteria	Microcystaceae	*Gloeocapsa* sp.	-	-	-
Fungi	Agaricaceae	*Tulostoma* sp.	-	-	-
Lichen	Acarosporaceae	*Acarospora peliscypha* Th. Fr.	-	-	-
		*Acarospora rosulata* (Th. Fr.) H. Magn.	-	-	-
		*Acarospora stapfiana* (Müll. Arg.) Hue	-	-	-
		*Acarospora strigata* (Nyl.) Jatta	-	-	-
		*Polysporina gyrocarpa* (H. Magn.) N. S. Golubk.	-	-	-
	Candelariaceae	*Candelariella rosulans* (Müll. Arg.) Zahlbr.	-	-	-
	Collemataceae	*Enchylium tenax* (Sw.) Gray	-	-	-
	Lecanoraceae	*Lecanora garovaglii* (Körber) Zahlbr.	-	-	-
	Physicaceae	*Buellia abstracta* (Nyl.) H. Olivier	-	-	-
	Teloschistaceae	*Caloplaca trachyphylla* (Tuck.) Zahlbr.	-	-	-
	Verrucariaceae	*Heteroplacidium compactum* (A. Massal.) Gueidan & Cl. Roux	-	-	-
		*Placidium acarosporoides* (Zahlbr.) Breuss	-	-	-
		*Placidium lachneum* (Ach.) Breuss	-	-	-
Vascular Plant	Amaranthaceae	*Atriplex confertifolia* (Torr. & Frém.) S. Watson	x	x	x
		*Atriplex corrugata* S. Watson	x	x	x
		Atriplex gardneri (Moq.) D. Dietr. var. cuneata (A. Nelson) S.L. Welsh	x	x	x
		*Halogeton glomeratus* (M. Bieb.) C.A. Mey.	x	x	x
		*Kali tragus* (L.) Scop.	x	x	x
	Asteraceae	*Artemisia filifolia* Torr.	x	x	x
		Chaenactis douglasii (Hook.) Hook. & Arn. var. douglasii		x	
		Dieteria canescens (Pursh) Nutt. var. canescens	x	x	x
		*Ericameria nauseosa* (Pall. ex Pursh) G.L. Nesom & G.I. Baird	x	x	x
		*Gaillardia spathulata* A. Gray	x	x	x^1^
		*Gutierrezia sarothrae* (Pursh) Britton & Rusby	x	x	x
		*Hymenoxys cooperi* (A. Gray) Cockerell	x		x
		*Scabrethia scabra* (Hook.) W.A. Weber	x	x	x
		*Thelesperma subnudum* A. Gray	x	x	x
	Boraginaceae	*Cryptantha humilis* (Greene) Payson	x	x	x
	Brassicaceae	*Lepidium montanum* Nutt.	x	x	x
	Cactaceae	Opuntia basilaris Engelm. & J.M. Bigelow var. basilaris	x	x	x
		Opuntia polyacantha Haw. var. polyacantha	x	x	x
	Ephedraceae	*Ephedra viridis* Colville	x	x	x
	Euphorbiaceae	*Euphorbia fendleri* Torr. & A. Gray	x	x	x
	Fabaceae	*Astragalus amphioxys* A. Gray	x	x	x
		*Astragalus desperatus* M.E. Jones	x	x	x
		*Astragalus lentiginosus* Douglas	x	x	x
	Juncaceae	*Juncus bufonius* L.		x	x
	Malvaceae	*Sphaeralcea coccinea* (Nutt.) Rydb.	x	x	x
		*Sphaeralcea parviflora* A. Nelson	x	x	x
	Onagraceae	Oenothera cespitosa Nutt. var. navajoensis (W.L. Wagner, Stockh. & W.M. Klein) Cronquist	x	x	x
	Poaceae	*Achnatherum hymenoides* (Roem. & Schult.) Barkworth	x	x	x
		Aristida purpurea var. longiseta (Steud.) Vasey	x	x	x
		Bouteloua barbata Lag. var. barbata		x	x
		*Bromus tectorum* L.	x	x	x
		*Dasyochloa pulchella* (Kunth) Willd. ex Rydb.	x	x	x
		*Hilaria jamesii* (Torr.) Benth.	x	x	x
		*Sporobolus airoides* (Torr.) Torr.	x	x	x
		*Sporobolus contractus* Hitchc.	x	x	x
	Polygonaceae	*Eriogonum inflatum* Torr. & Frém.	x	x	x
		*Eriogonum shockleyi* S. Watson	x	x	x
	Sarcobataceae	*Sarcobatus vermiculatus* (Hook.) Torr.	x	x	x^1^
	Tamaricaceae	*Tamarix ramosissima* Ledeb.	x	x	x
