# Supplementary figures and images for: The "Martian" flora: new collections of vascular plants, lichens, fungi, algae, and cyanobacteria from the Mars Desert Research Station, Utah
Source: Biodivers Data J. 2016 Jun 9;(4):e8176. doi: 10.3897/BDJ.4.e8176 (PMC4911540; doi:10.3897/BDJ.4.e8176)

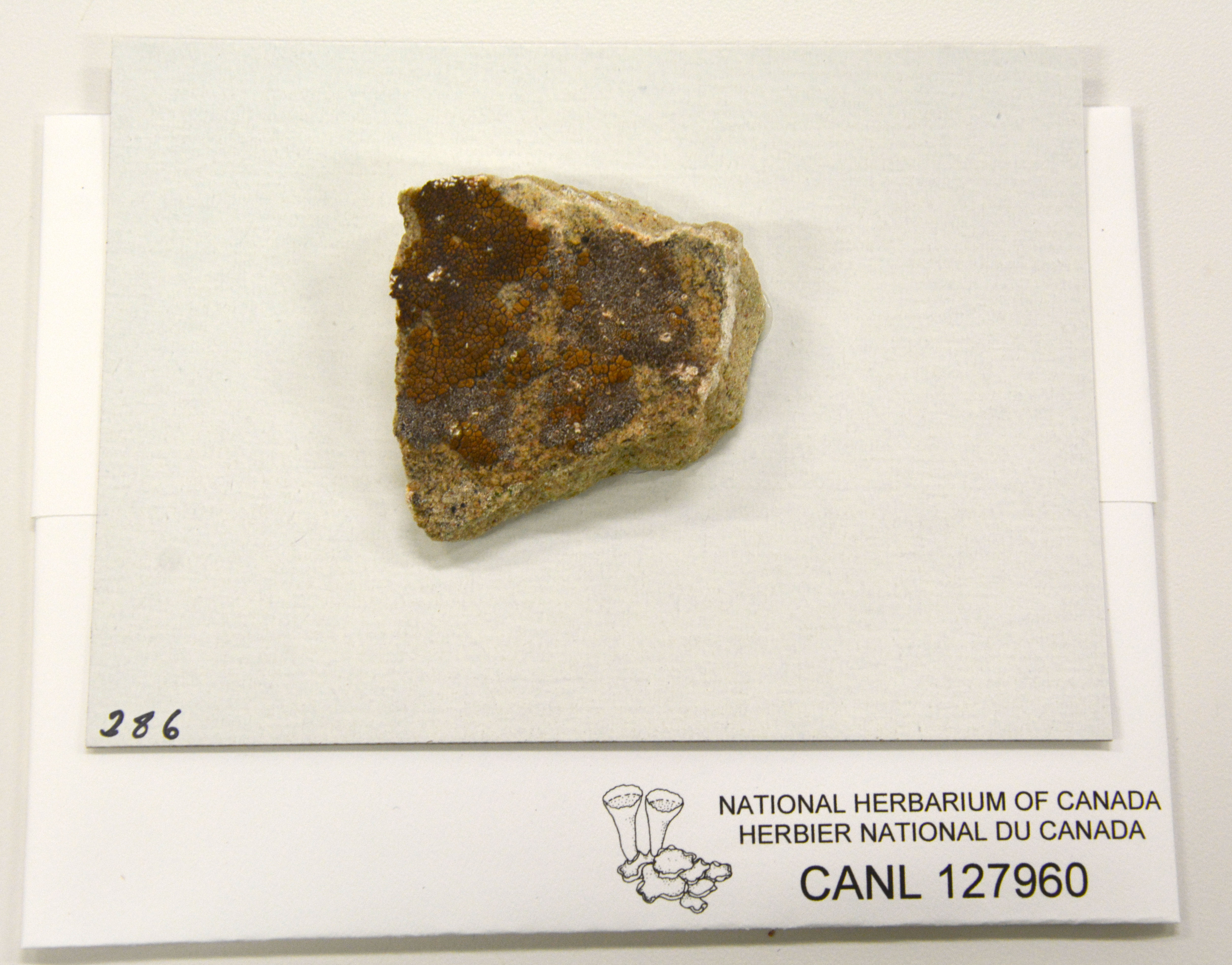

Supplement: Supplementary material 1 — CANL 127960, Acarospora peliscypha (Sokoloff 286) [file biodiversity_data_journal-4-e8176-s001.jpg]

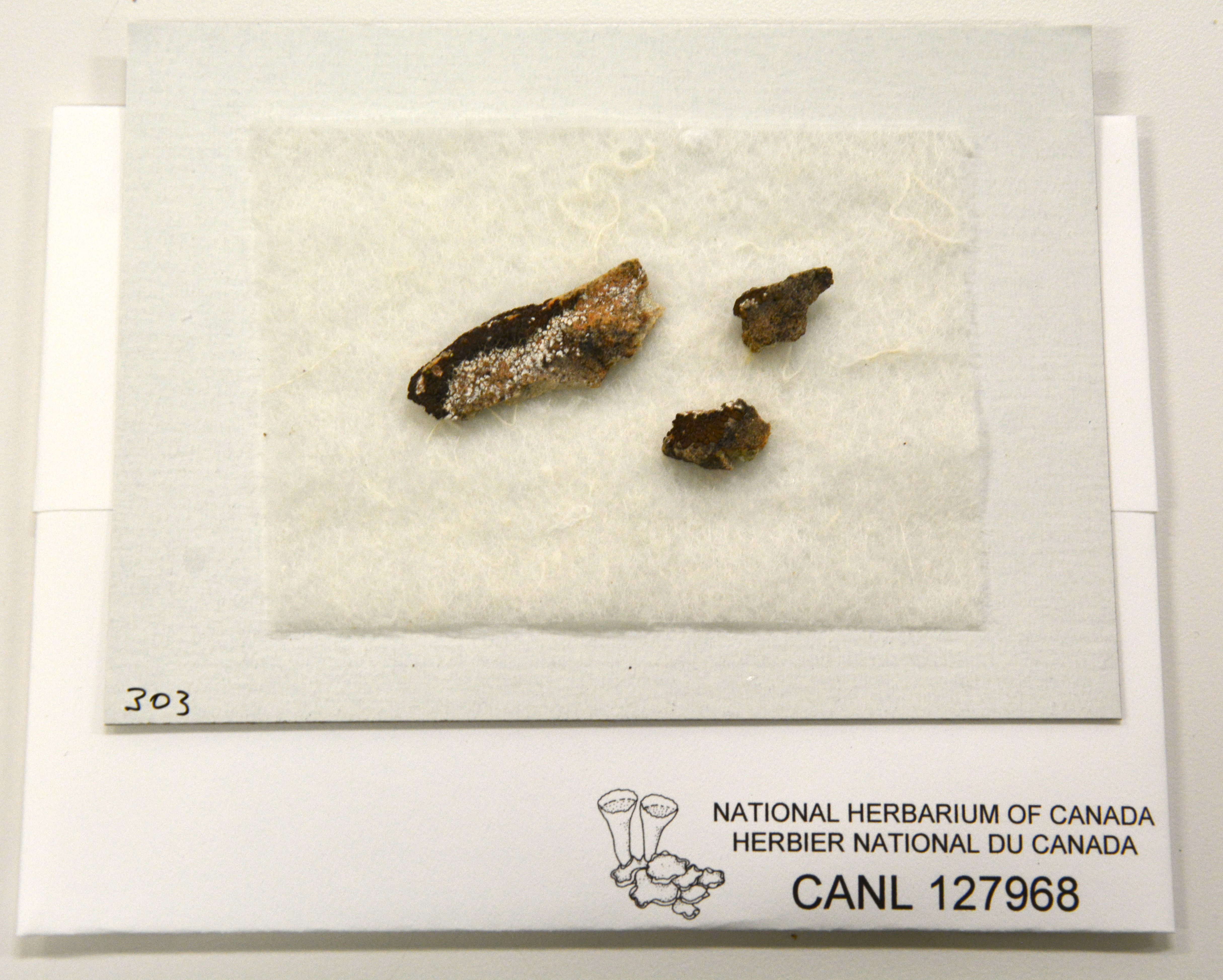

Supplement: Supplementary material 2 — CANL 127968, Acarospora rosulata (Sokoloff 303) [file biodiversity_data_journal-4-e8176-s002.jpg]

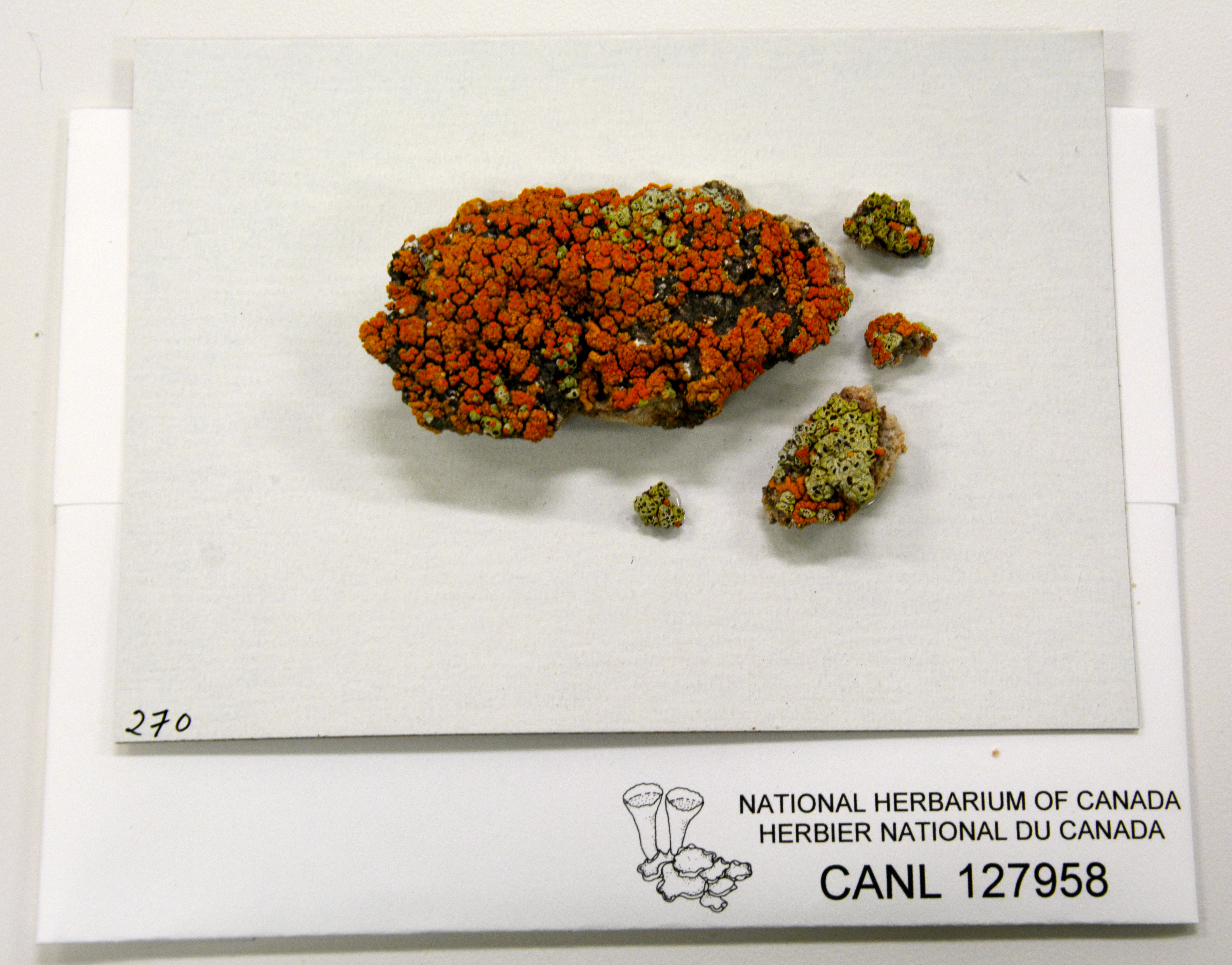

Supplement: Supplementary material 3 — CANL 127958, Acarospora stapfiana (Sokoloff 270) [file biodiversity_data_journal-4-e8176-s003.jpg]

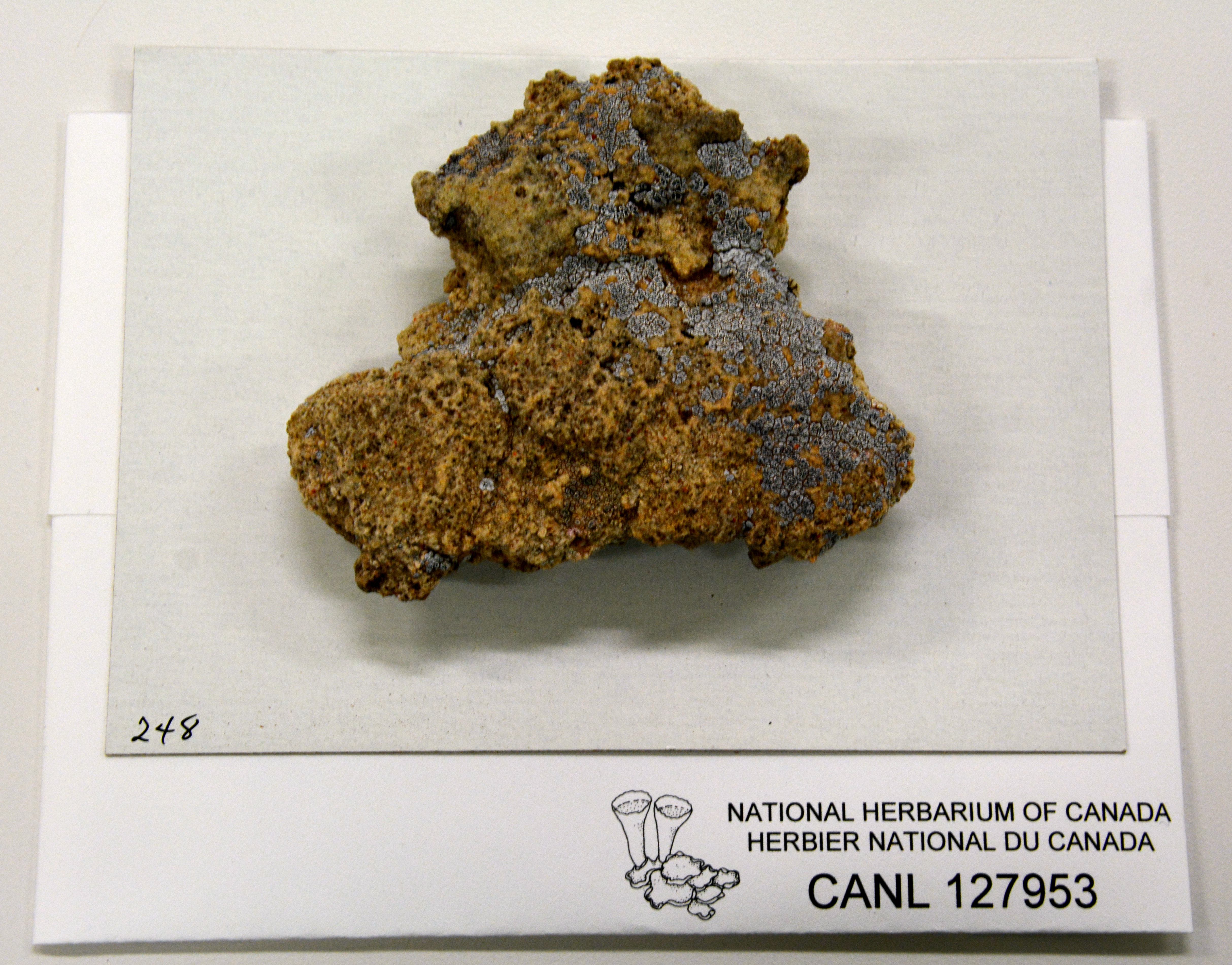

Supplement: Supplementary material 4 — CANL 127953, Acarospora strigata (Sokoloff 248) [file biodiversity_data_journal-4-e8176-s004.jpg]

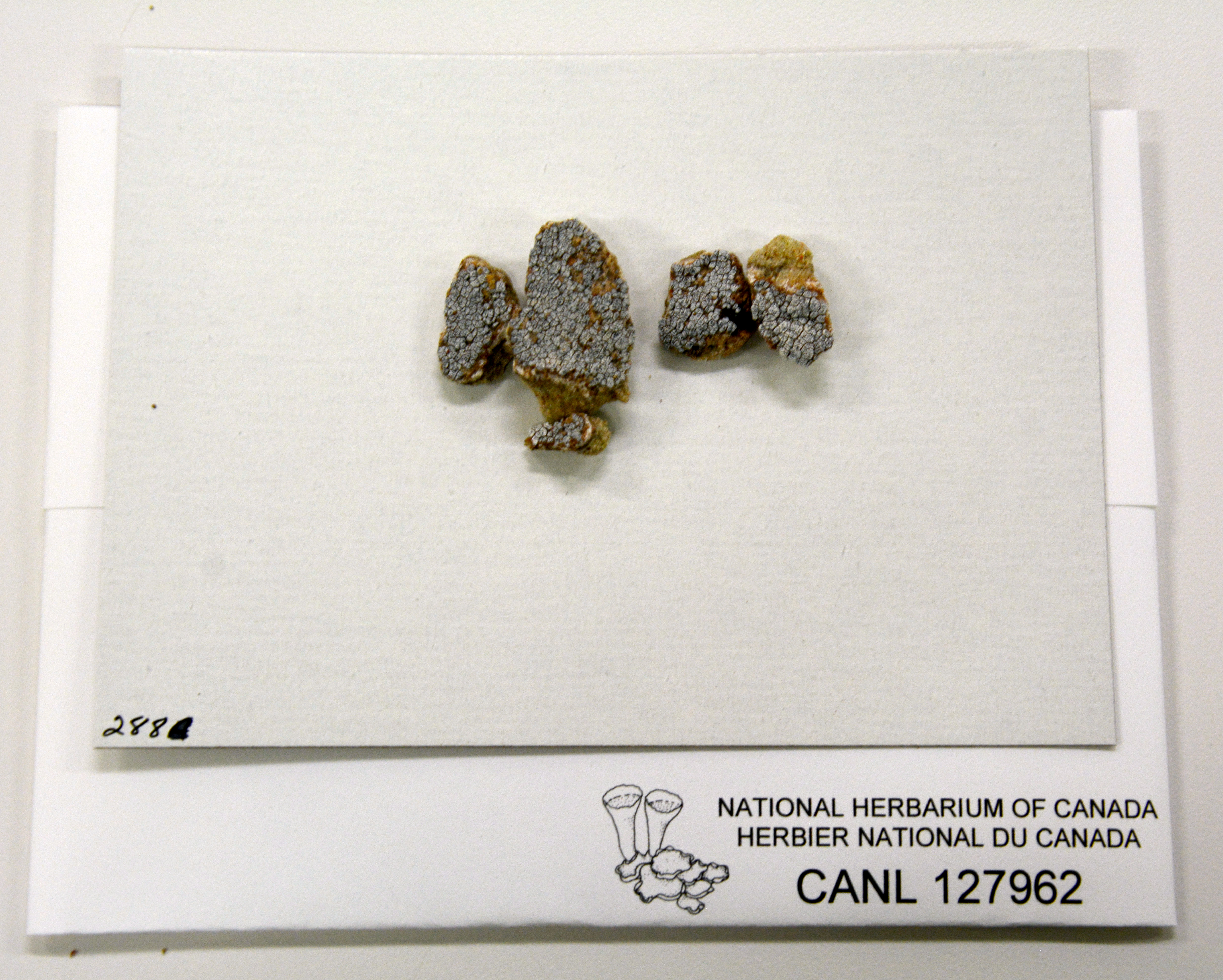

Supplement: Supplementary material 5 — CANL 127962, Acarospora strigata (Sokoloff 288) [file biodiversity_data_journal-4-e8176-s005.jpg]

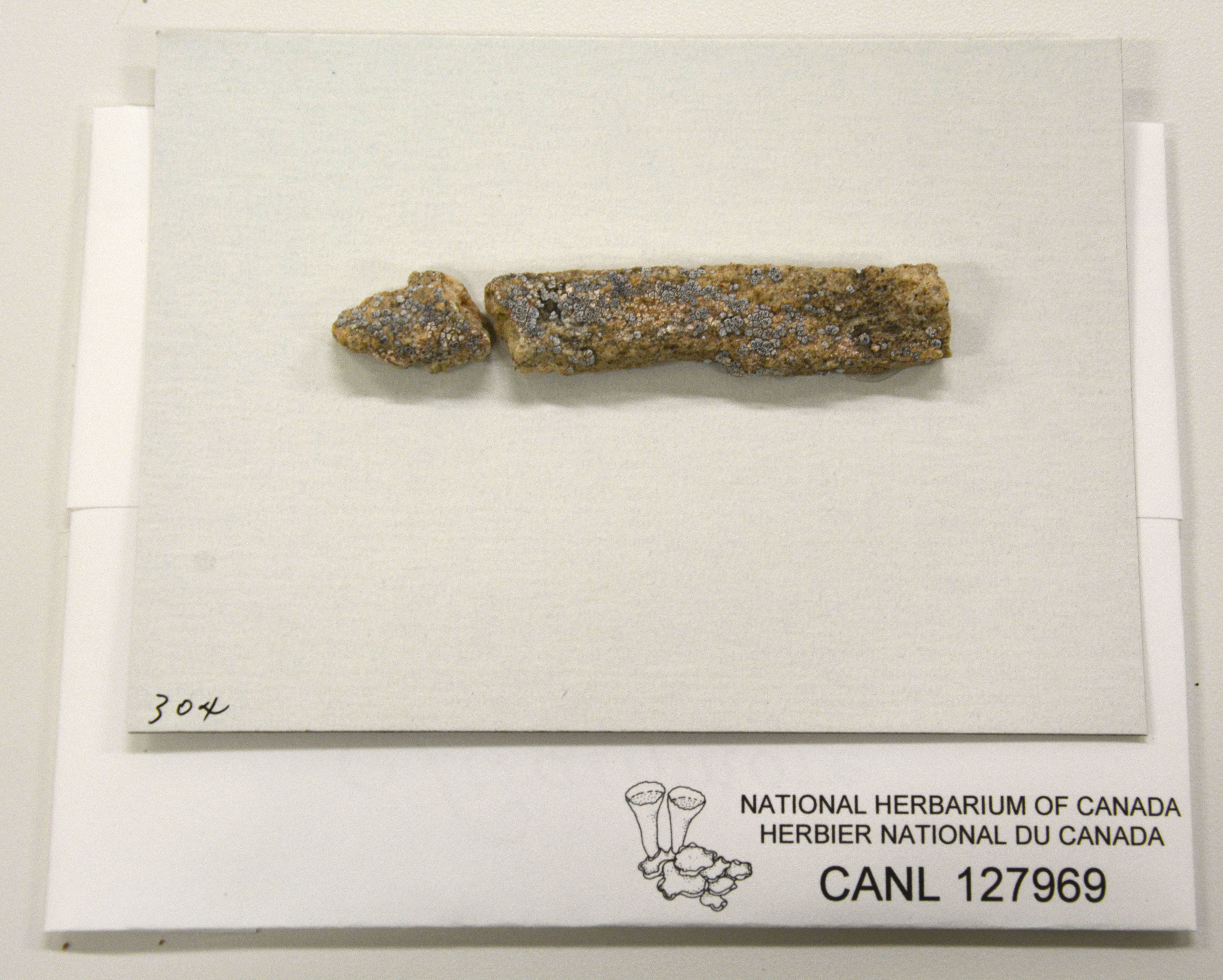

Supplement: Supplementary material 6 — CANL 127969, Acarospora strigata (Sokoloff 304) [file biodiversity_data_journal-4-e8176-s006.jpg]

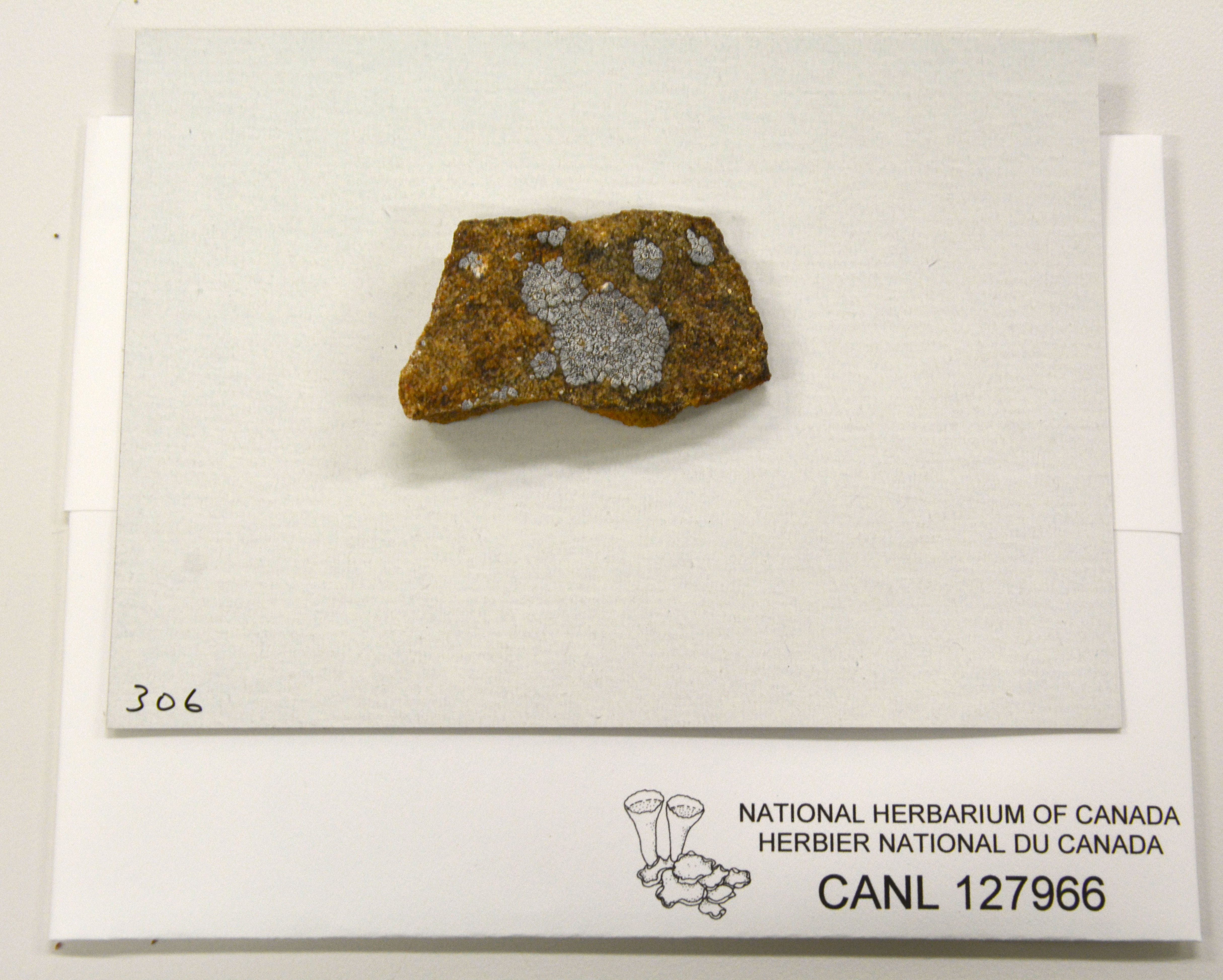

Supplement: Supplementary material 7 — CANL 127966, Acarospora strigata (Sokoloff 306) [file biodiversity_data_journal-4-e8176-s007.jpg]

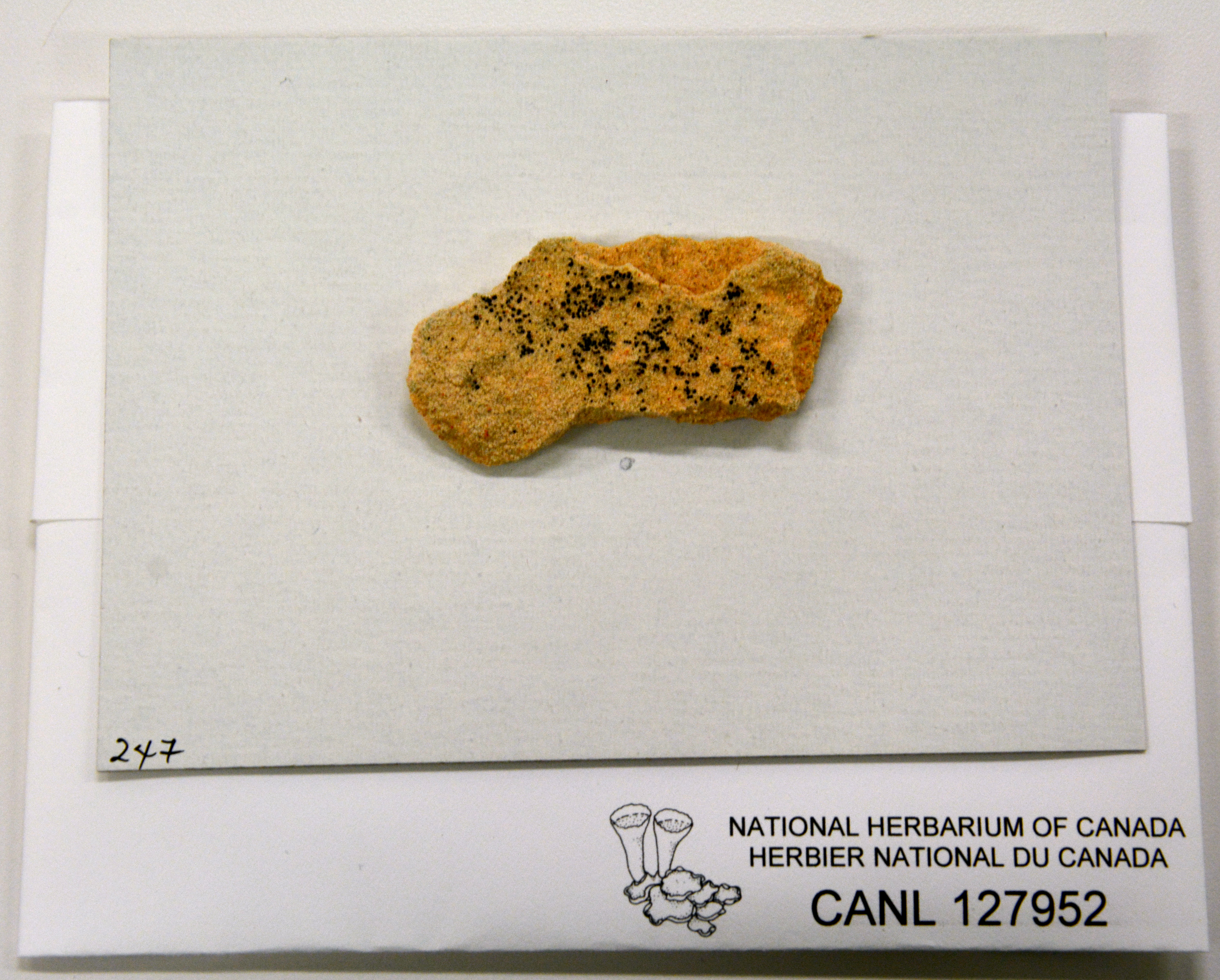

Supplement: Supplementary material 8 — CANL 127952, Polysporina gyrocarpa (Sokoloff 247) [file biodiversity_data_journal-4-e8176-s008.jpg]

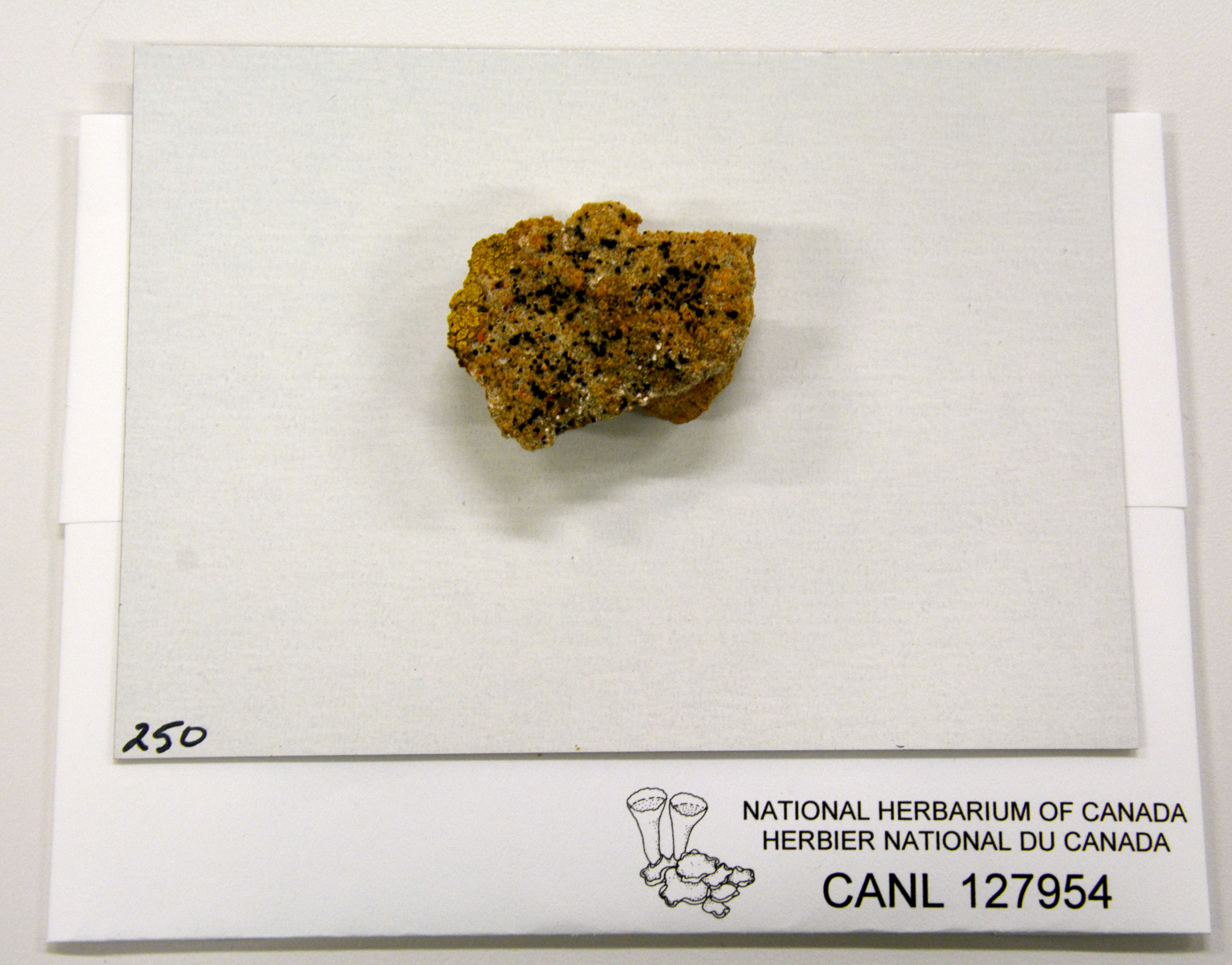

Supplement: Supplementary material 9 — CANL 127954, Polysporina gyrocarpa (Sokoloff 250) [file biodiversity_data_journal-4-e8176-s009.jpg]

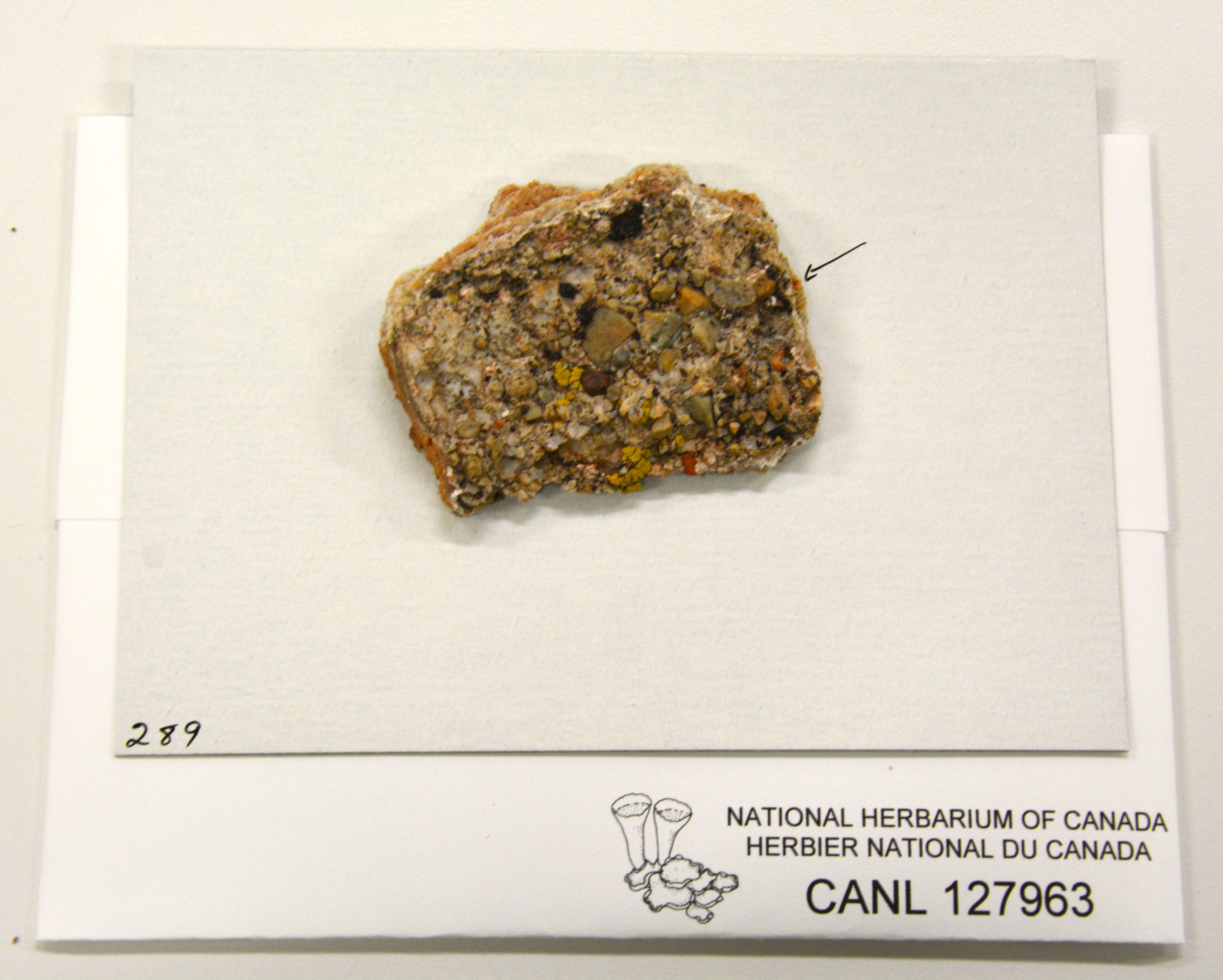

Supplement: Supplementary material 10 — CANL 127963, Polysporina gyrocarpa (Sokoloff 289) [file biodiversity_data_journal-4-e8176-s010.jpg]

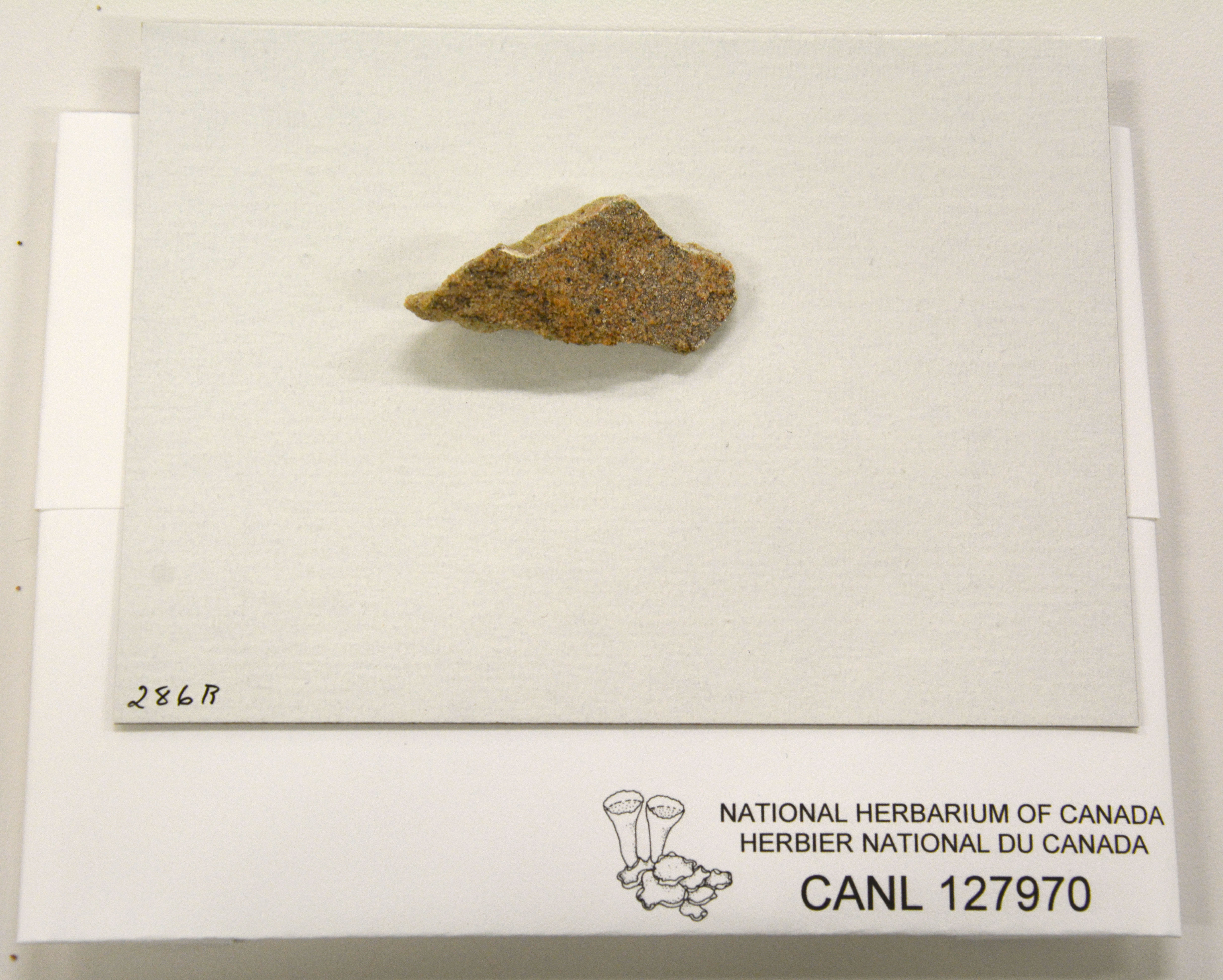

Supplement: Supplementary material 11 — CANL 127970, Polysporina gyrocarpa (Sokoloff 286b) [file biodiversity_data_journal-4-e8176-s011.jpg]

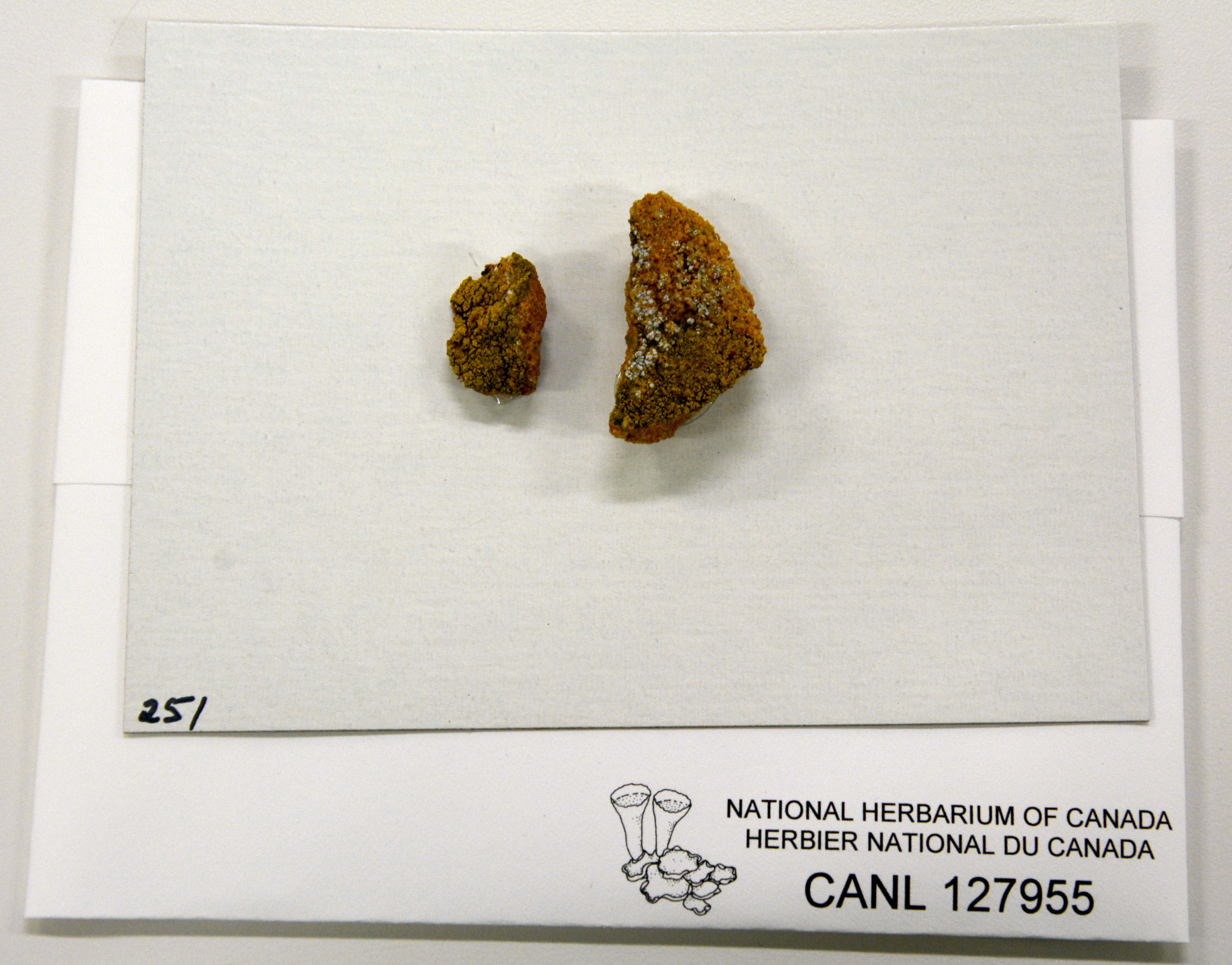

Supplement: Supplementary material 12 — CANL 127955, Candelariella cf. rosulans (Sokoloff 251) [file biodiversity_data_journal-4-e8176-s012.jpg]

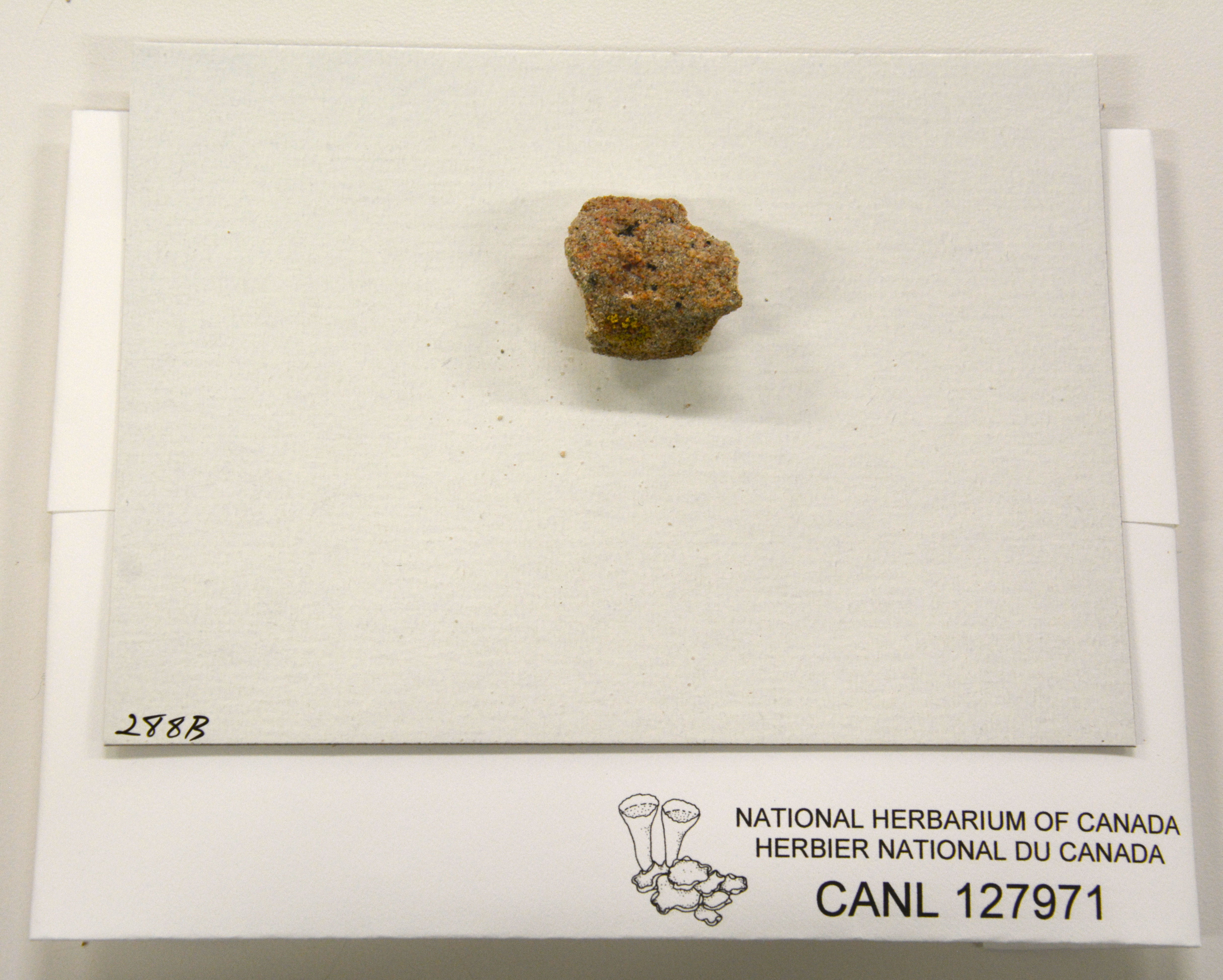

Supplement: Supplementary material 13 — CANL 127971, Candelariella rosulans (Sokoloff 288b) [file biodiversity_data_journal-4-e8176-s013.jpg]

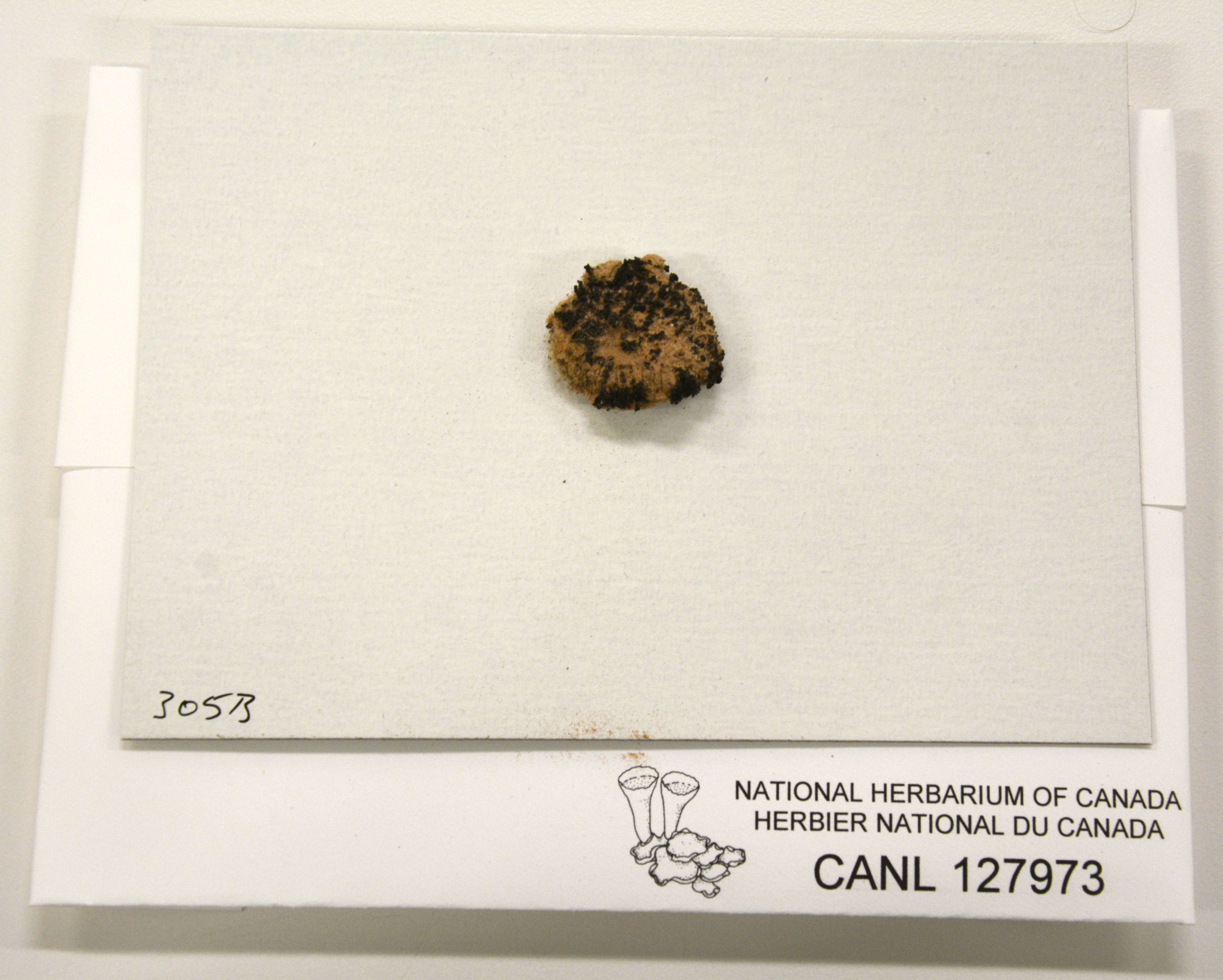

Supplement: Supplementary material 14 — CANL 127973, Enchylium tenax (Sokoloff 305b) [file biodiversity_data_journal-4-e8176-s014.jpg]

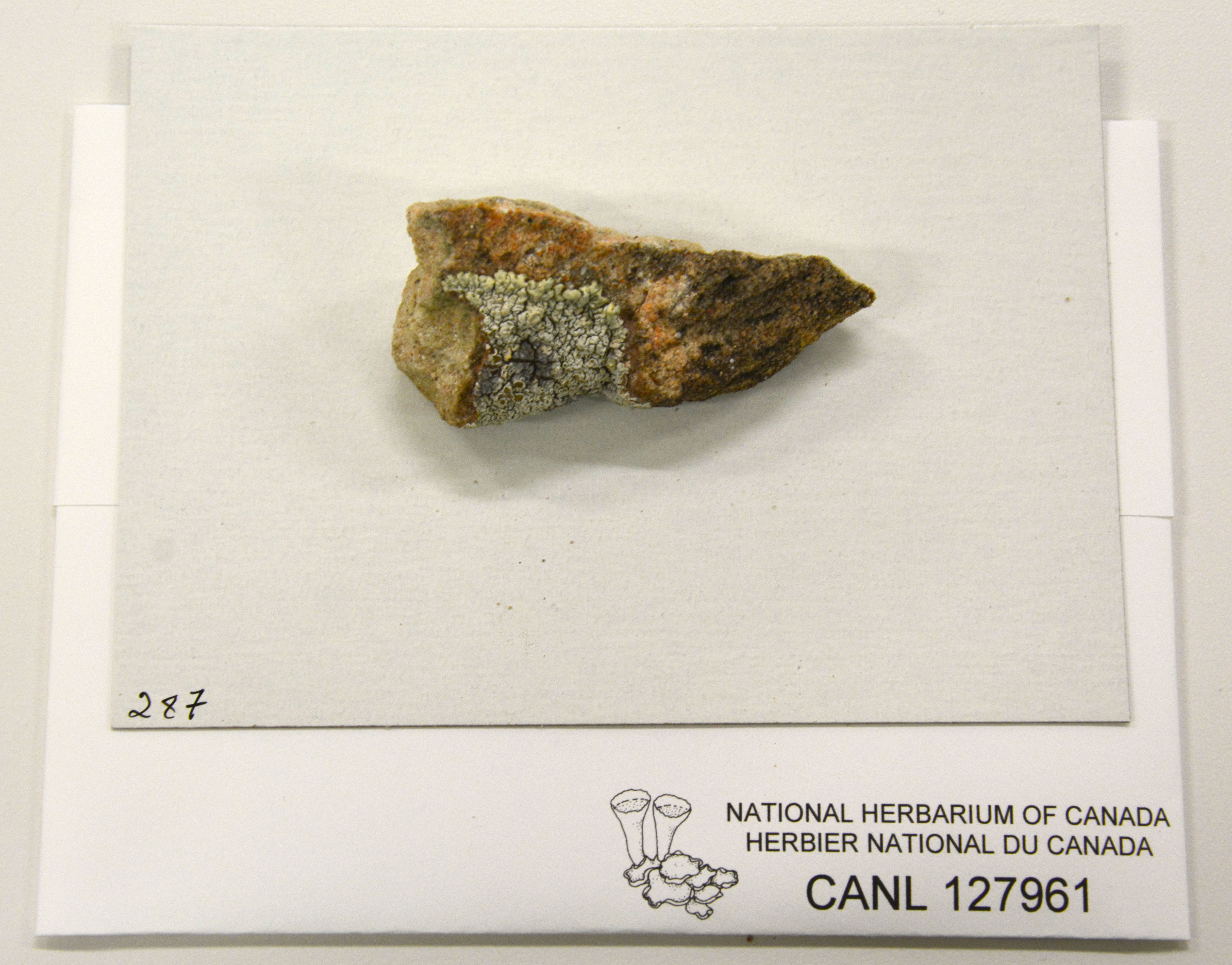

Supplement: Supplementary material 15 — CANL 127961, Lecanora garovaglii (Sokoloff 287) [file biodiversity_data_journal-4-e8176-s015.jpg]

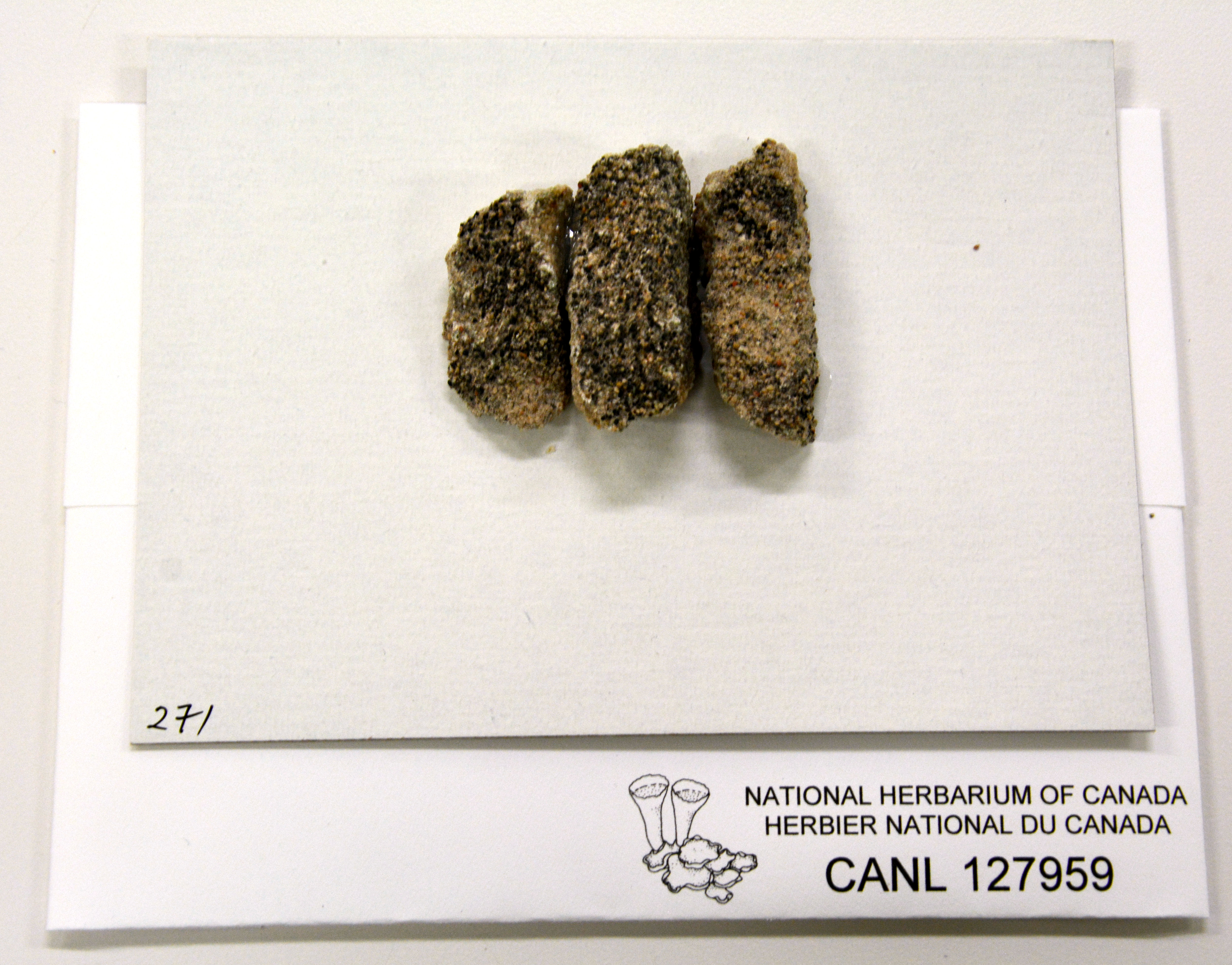

Supplement: Supplementary material 16 — CANL 127959, Buellia abstracta (Sokoloff 271) [file biodiversity_data_journal-4-e8176-s016.jpg]

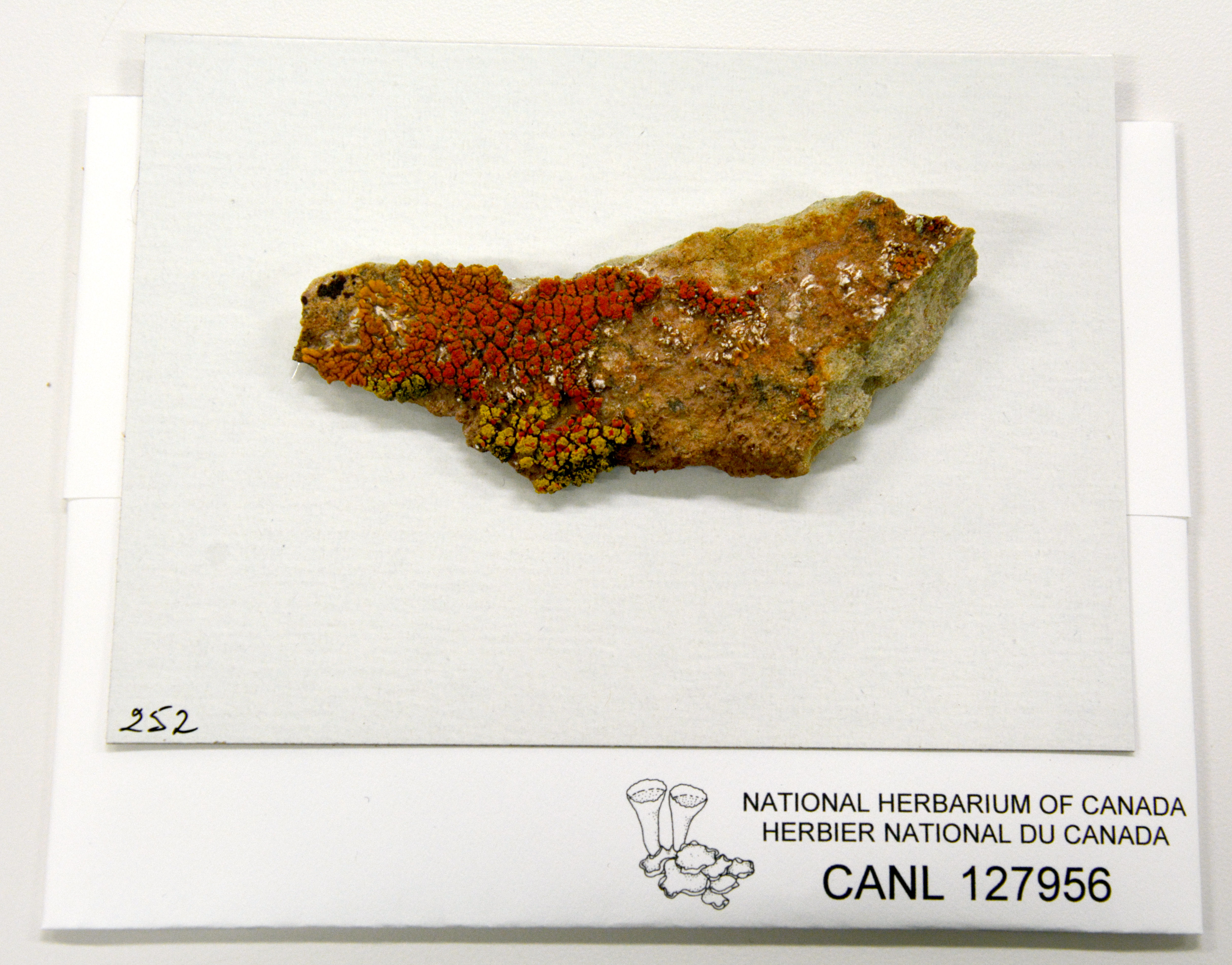

Supplement: Supplementary material 17 — CANL 127956, Caloplaca trachyphylla (Sokoloff 252) [file biodiversity_data_journal-4-e8176-s017.jpg]

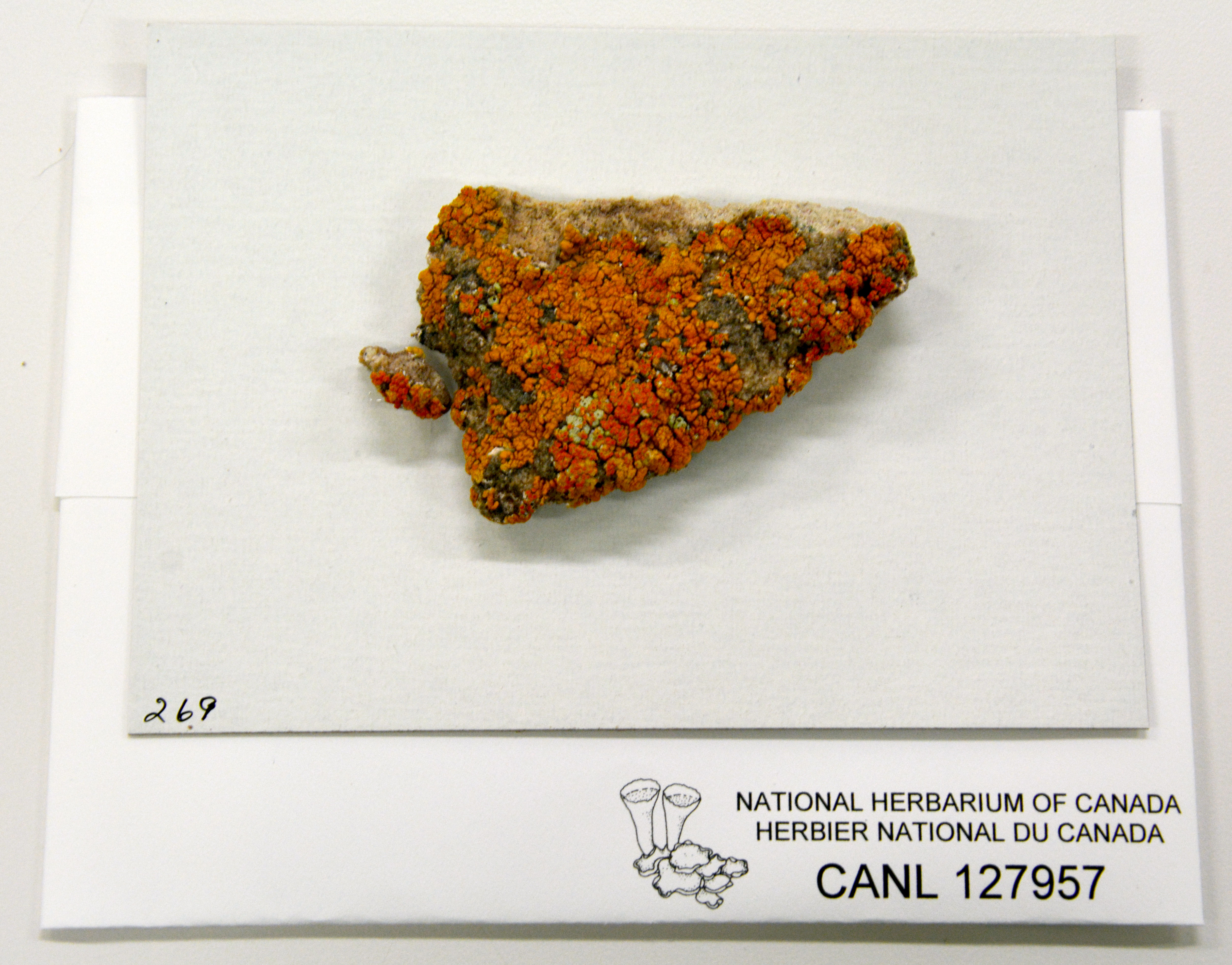

Supplement: Supplementary material 18 — CANL 127957, Caloplaca trachyphylla (Sokoloff 269) [file biodiversity_data_journal-4-e8176-s018.jpg]

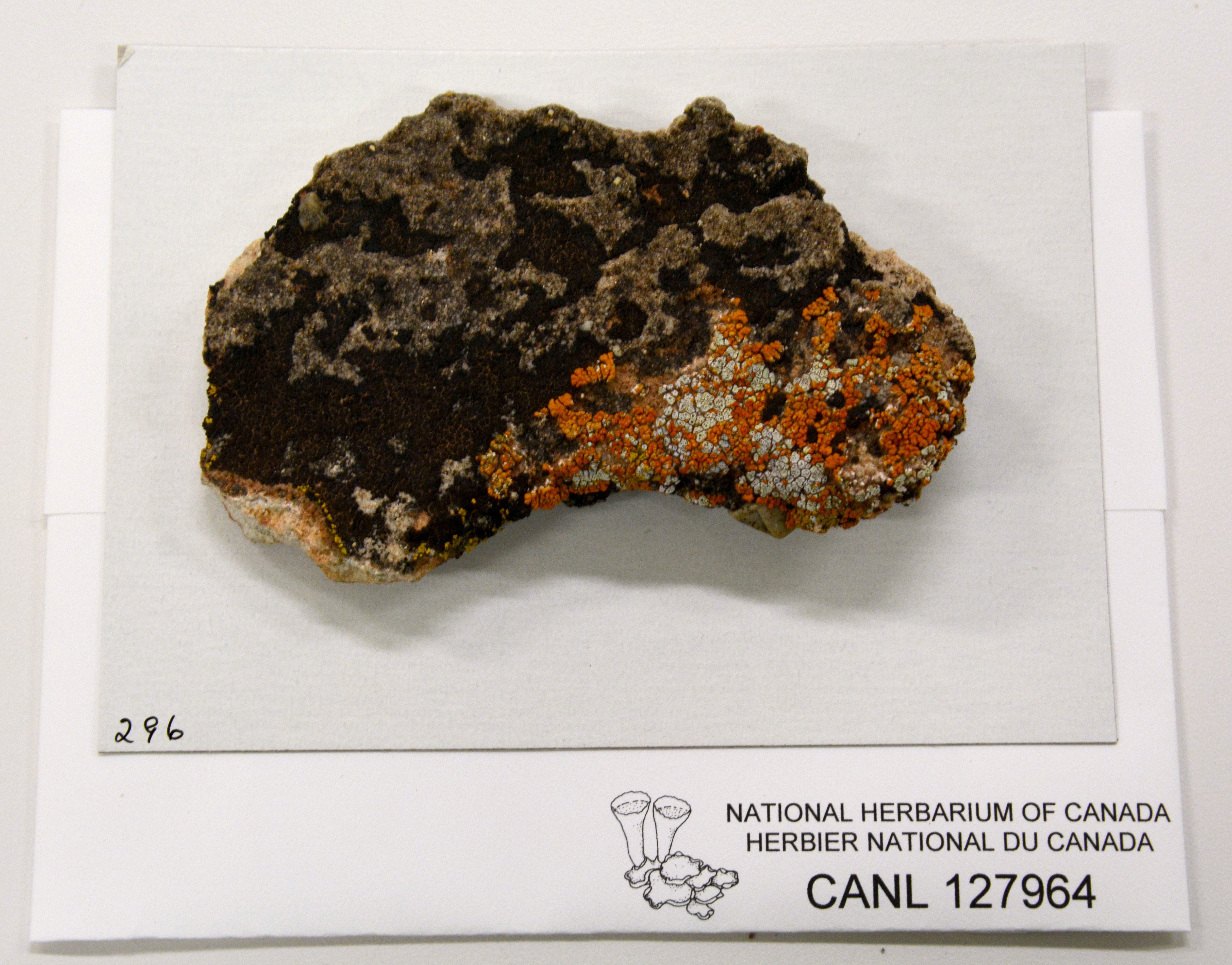

Supplement: Supplementary material 19 — CANL 127964, Heteroplacidium compactum (Sokoloff 296) [file biodiversity_data_journal-4-e8176-s019.jpg]

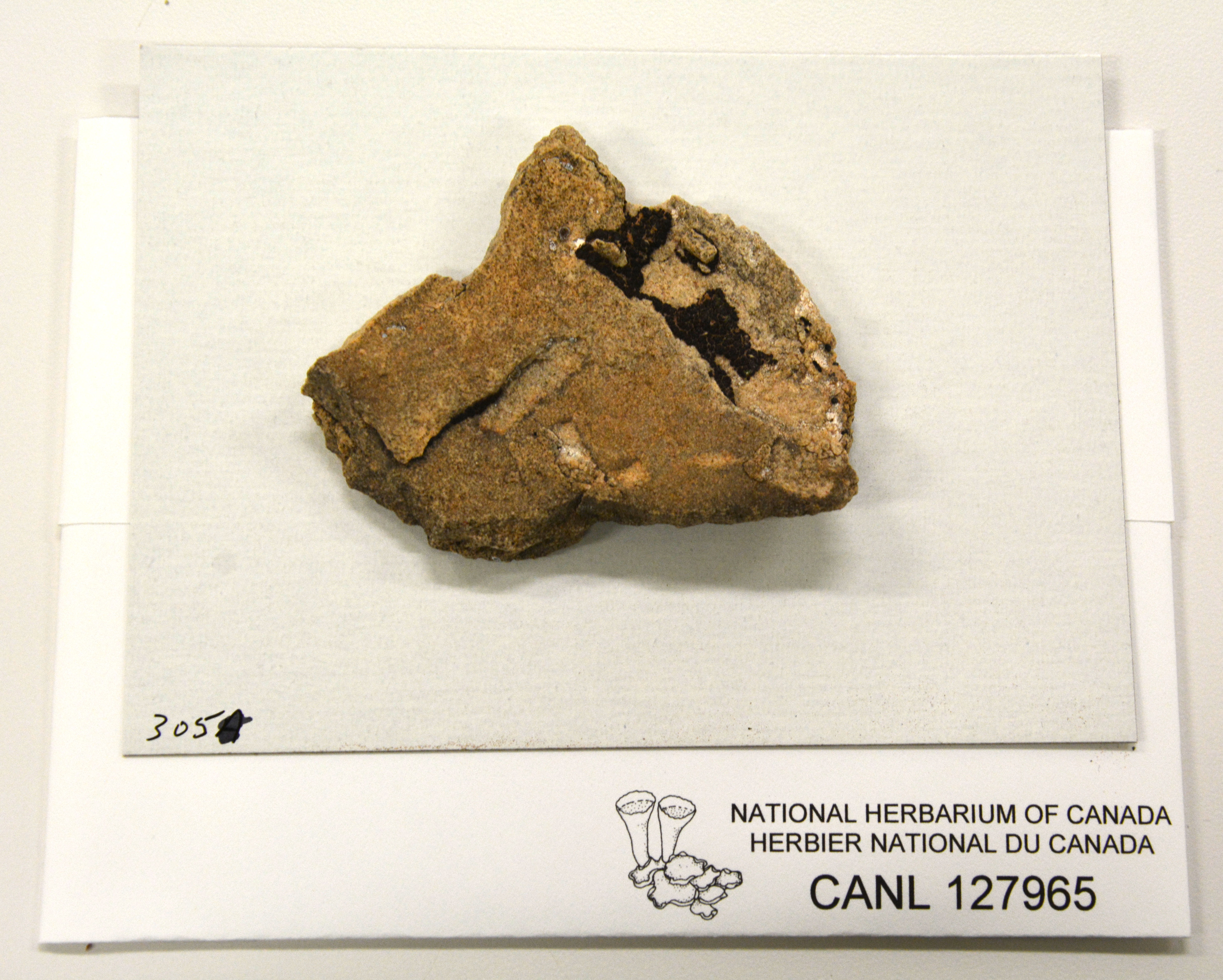

Supplement: Supplementary material 20 — CANL 127965, Placidium acarosporoides (Sokoloff 305) [file biodiversity_data_journal-4-e8176-s020.jpg]

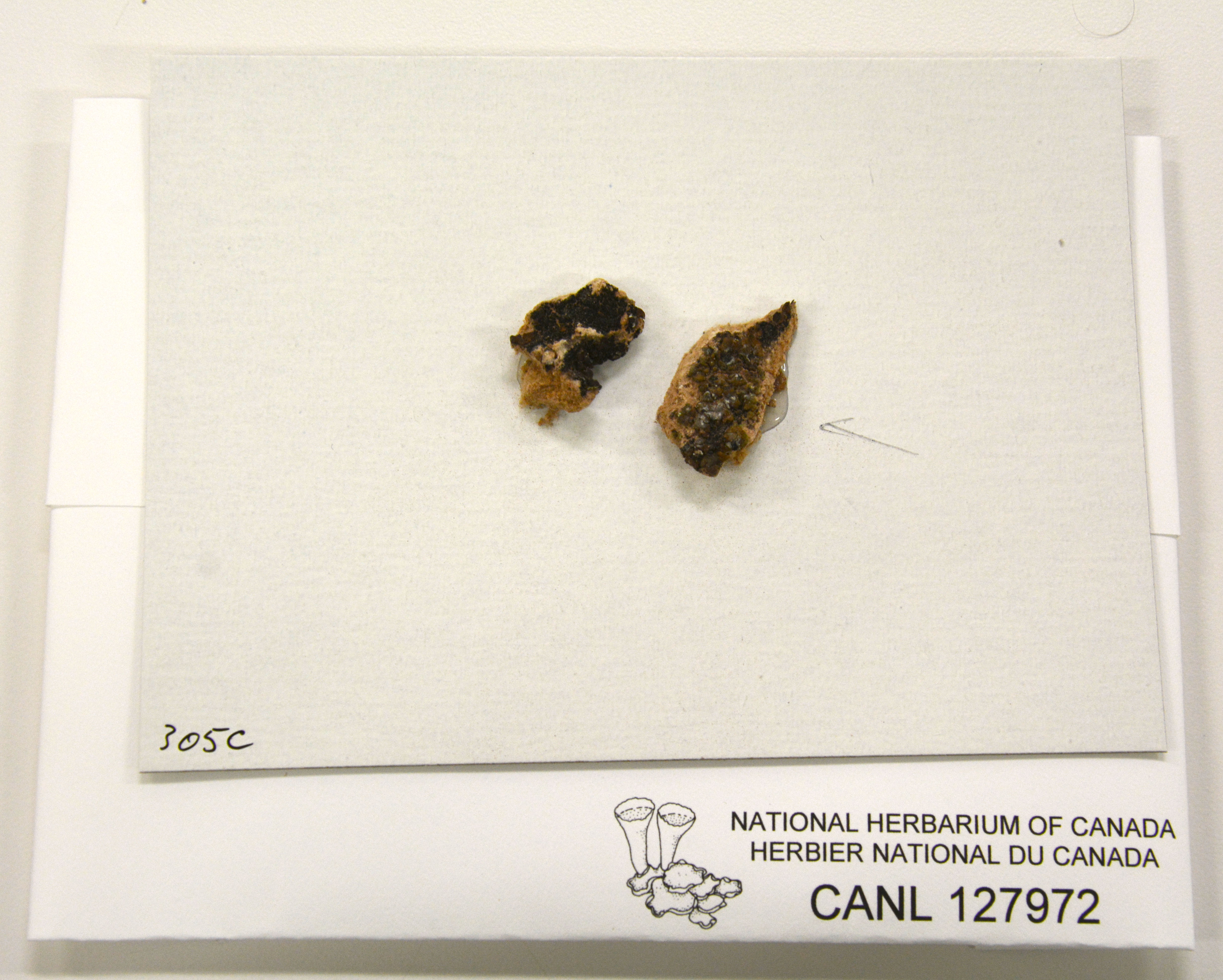

Supplement: Supplementary material 21 — CANL 127972, Placidium lachneum (Sokoloff 305c) [file biodiversity_data_journal-4-e8176-s021.jpg]

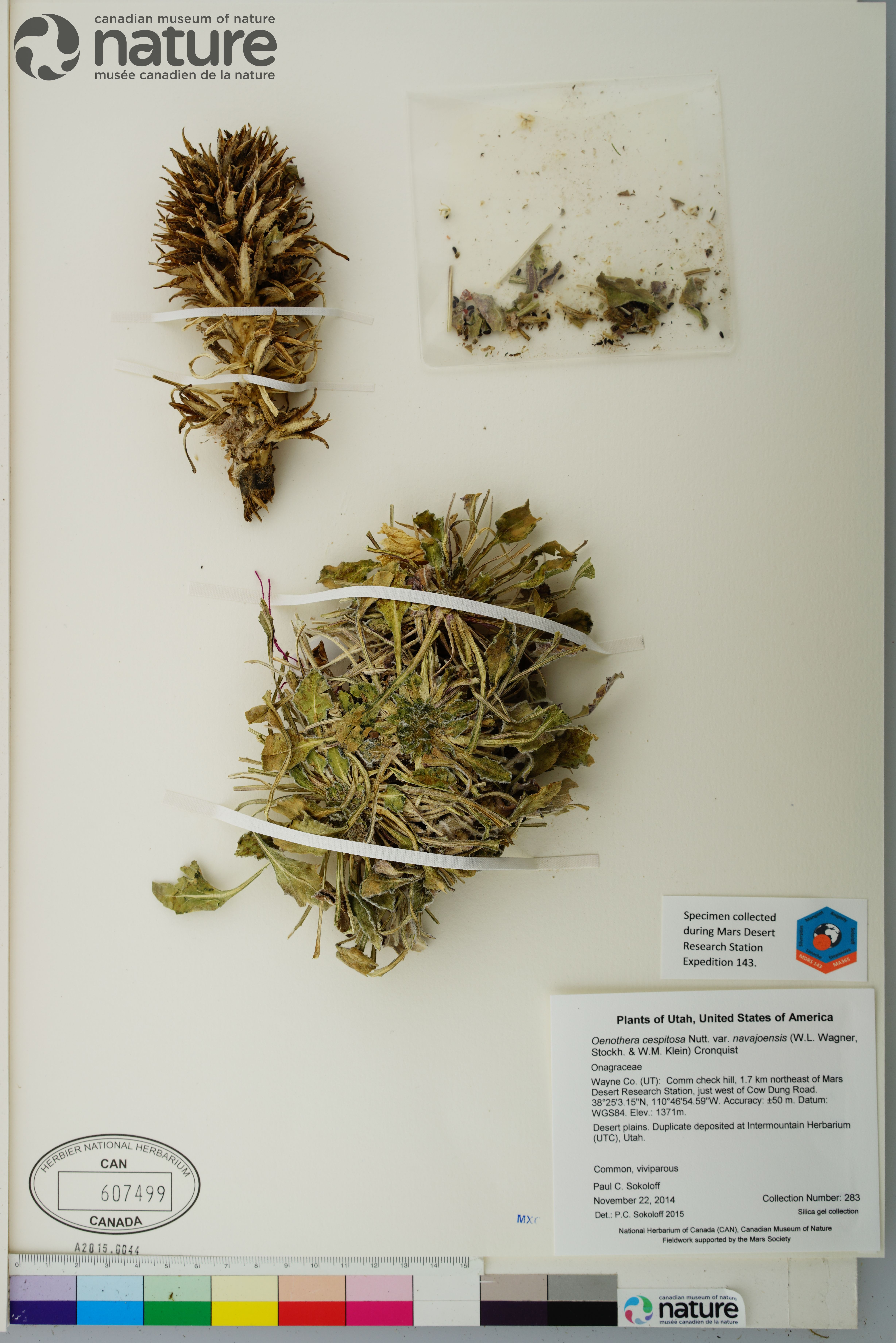

Supplement: Supplementary material 56 — CAN 607499, Oenothera cespitosa var. navajoensis (Sokoloff 283) [file biodiversity_data_journal-4-e8176-s056.jpg]
